# Orthogonal Syntheses of 3.2.0 Bicycles from Enallenes Promoted by Visible Light

**DOI:** 10.1021/acs.orglett.0c02193

**Published:** 2020-08-05

**Authors:** Andrea Serafino, Davide Balestri, Luciano Marchiò, Max Malacria, Etienne Derat, Giovanni Maestri

**Affiliations:** † Department of Chemistry, Life Sciences and Environmental Sustainability, Università di Parma, Parco Area delle Scienze 17/A, 43124 Parma, Italy; ‡ Faculty of Science and Engineering, CNRS, Institut Parisien de Chimie Moléculaire (UMR CNRS 8232), 4 place Jussieu, Paris 75252 Cedex 05, France

## Abstract

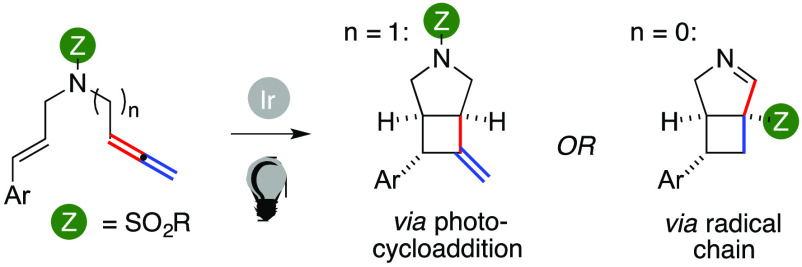

Enallenes can be readily converted into two families of 3.2.0 (hetero)bicycles with high diastereoselectivities through the combination of visible light with a suitable Ir(III) complex (1 mol %). Two complementary pathways, namely, a photocycloaddition versus a radical chain, can then take place. Both manifolds grant complete regiocontrol of the allene difunctionalization. This is accompanied by an original 1,3-group shift using sulfonyl allenamides that deliver a congested tetrasubstituted headbridging carbon in the corresponding product.

The 3.2.0 bicyclic unit is found in a myriad of natural products and functional bioactive molecules.^1^ Several of these examples feature an alkene motif that suggests sealing the four-membered ring via an intramolecular [2 + 2] cycloaddition from an enallene.^2^ However, the allene functionalization presents regiochemical challenges (Scheme 1). This issue is shown in the synthesis of Bielschowskysin, in which a mixture of isomers was observed in the key annulation promoted by UV-C light.^3^

**Scheme 1:**
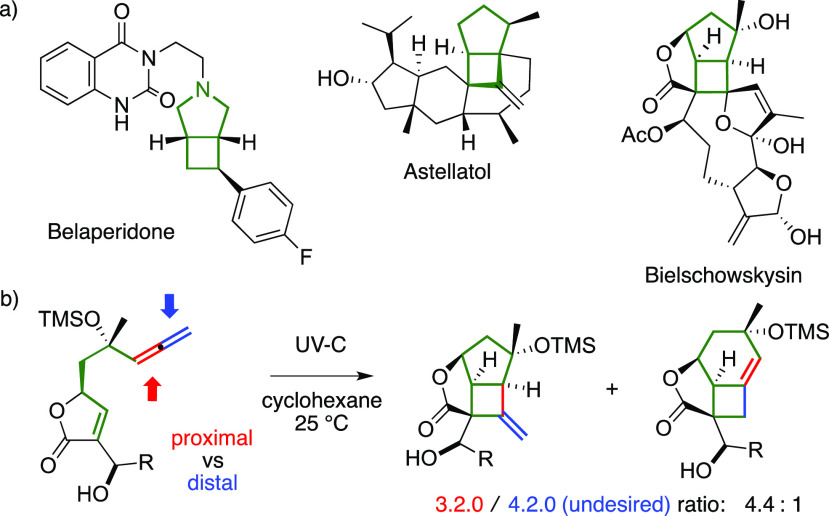
3.2.0 Unit in Functional Molecules and Regiochemical Issues in [2 + 2] Photocycloaddition of Enallenes

Complete regiochemical control has been achieved in a few cases (Scheme 2). The thermal activation of 1,7-enallenes leads to the corresponding 4.2.0 bicycles with an endocyclic C–C double bond.^4^ However, this approach requires heating to 150 °C. Cationic gold(I) complexes can smoothly activate analogous substrates at room temperature, leading to 3.2.0 products instead. However, a selective reaction requires that the gold catalyst could discriminate between the two cumulated double bonds, limiting the method to trisubstituted allenes.^5^

**Scheme 2:**
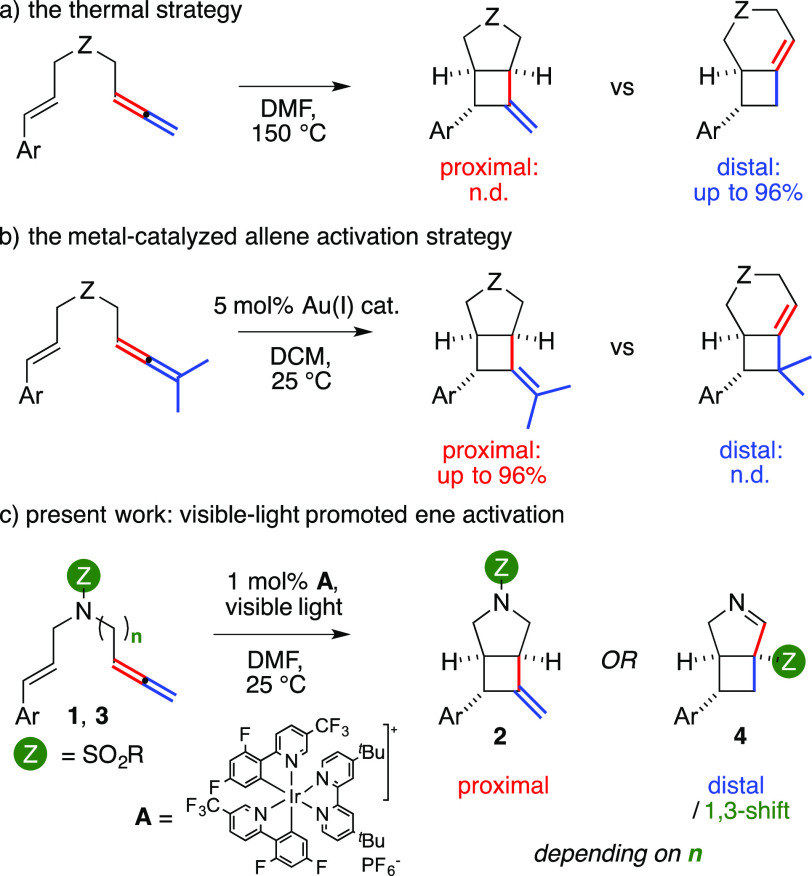
Regiocontrol in [2 + 2] Cycloadditions of Enallenes

We report here the first general visible-light-promoted cyclization method using enallenes. This approach uses an Ir(III) photoactive complex to activate the alkene arm,^6^ pivoting on the seminal work of Yoon on the activation of styryl fragments, regardless of their electronic demands,^2e^ via energy transfer (eT)^6a,6b^ to trigger intramolecular [2 + 2] cycloadditions with a tethered alkene partner. This strategy is complementary to thermal and organometallic strategies for the cyclization of enallenes. The present process allows us to control the functionalization of *either* the proximal *or* the distal position of the allenyl fragment.

The reaction of 1,7-enallenes **1** delivers 3.2.0 bicycles **2**, which have three contiguous stereocenters, with high regio- and diastereocontrol. A different tether between the two unsaturated partners reverts the outcome under otherwise identical conditions. Indeed, the use of 1,6-enallenes **3** leads to products **4**. Furthermore, a formal 1,3-sigmatropic rearrangement of sulfonyl groups occurs,^7^ creating a sterically congested quaternary carbon at the headbridge position and an imine function. This synthetically challenging architecture likely forms through the propagation of a radical chain, in striking contrast with the catalytic photocycloaddition that gives product **2**.

We turned our attention to the reactivity of 1,7-enallene **1a** as part of an ongoing interest in the cyclization sequences of polyunsaturated substrates that allow complete atom economy.^8^ We found that **1a** could smoothly deliver **2a** in 82% isolated yield in the presence of 1 mol % of Ir(III) complex **A** (Figure 1) upon a preliminary screening of the reaction conditions. Reactions were carried out on 0.2 mmol scale under N_2_ in degassed dimethylformamide (DMF). The mixtures were placed in nuclear magnetic resonance (NMR) tubes, kept at 25 °C without stirring, and irradiated for 16 h with an light-emitting diode (LED) strip.^9^ The structure of **2a** was confirmed by X-ray analysis, which confirmed the relative configuration of the three stereocenters initially assigned by correlation NMR experiments.

**Figure 1:**
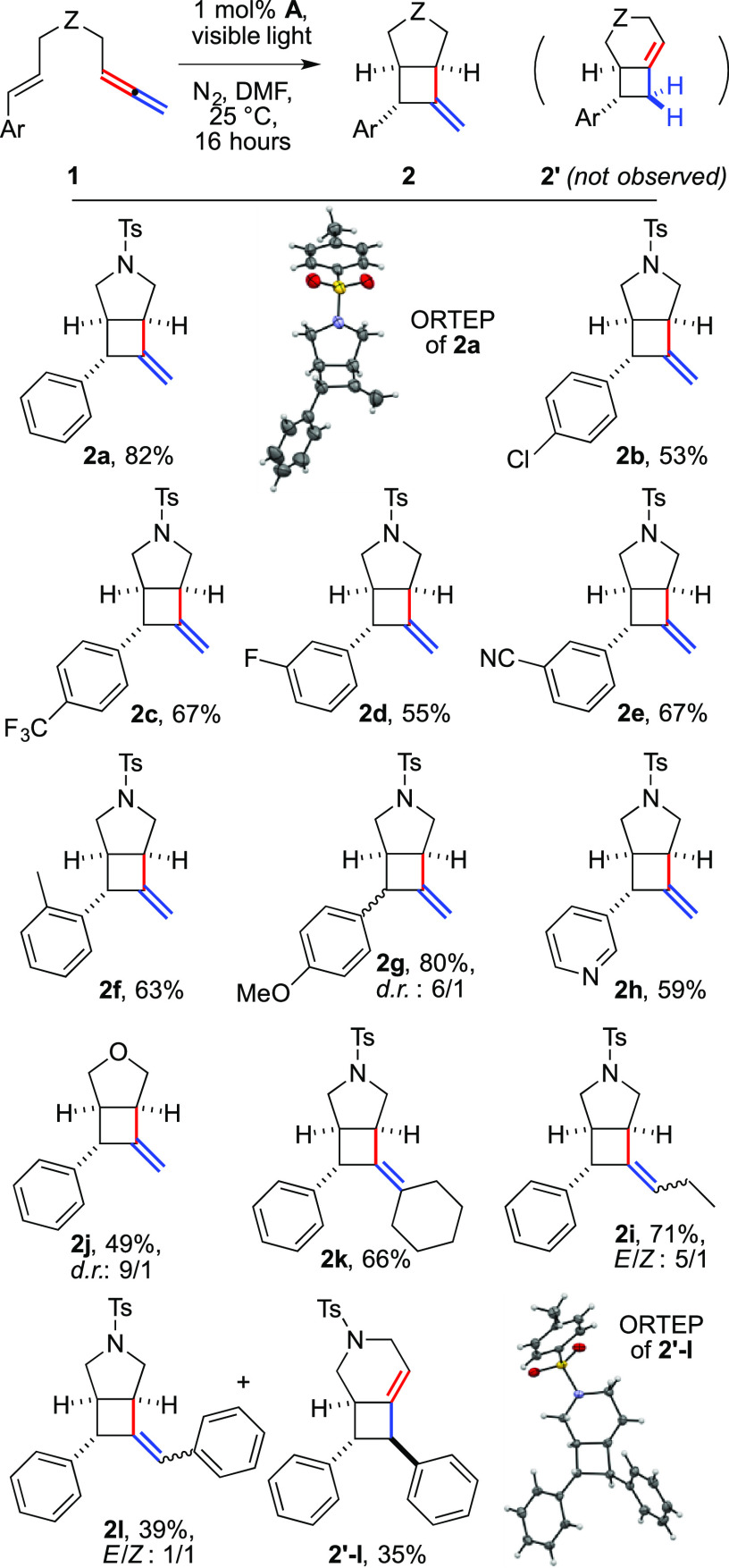
Synthesis of vinylidencyclobutanes **2**.

Encouraged by this result, we prepared a library of 1,7-enallenes through homologation of the corresponding 1,6-enynes.^10^ The styryl arm could be decorated by electron-withdrawing groups, including halides and cyano and trifluoromethyl groups, and the corresponding products were retrieved in moderate to good yields (**2b**–**e**, 53–67%). Similarly, the presence of donating substituents was well tolerated (**2f**,**g**, 63 and 80%, respectively), although the diastereocontrol was lower using reagent **1g** (dr = 6:1). Heteroaromatics could be employed, as witnessed by **2h**, which had a 3-pyridyl group (59%). The presence of an ethereal tether led to **2j** (49%, dr = 9:1). Complete control of the functionalization of the allene proximal double bond was observed using a trisubstituted allene (**2k**, 66%). A disubstituted one followed suit (**2i**, 71%), although the product was retrieved as mixture of E/Z isomers (5:1). The substrate **1l**, which had a styryl arm and a phenylallene arm, afforded two products. Together with **2l** (39%, 1:1 E/Z mixture), we isolated **2′**-**l** in 35% yield and clarified its structure by X-ray analysis. It had a 4.2.0 bicyclic unit with three contiguous stereocenters, and its two phenyl rings were anti with respect to each other. The structure of **2′**-**l** showed that difunctionalization of the distal double bond of the allene might have been possible under the present conditions.

We thus thought to elicit a distal-selective variant of the reaction by preparing 1,6-enallenes, reasoning that the formation of a 3.2.0 product **4′** would have been favored over that of the corresponding 2.2.0 product (**4′′**, Figure 2). Substrates were synthesized by isomerization of the corresponding 1,6-enynes. Gratifyingly, the reaction of **3a** indeed gave a 3.2.0 bicycle. However, crystallization and subsequent X-ray analysis revealed that its structure was different from the expected one, namely, **4′**. Surprisingly, sulfonamide activation and formal 1,3-sigmatropic rearrangement of the sulfonyl group took place,^7^ eventually delivering bicyclic imine **4a** (59%) as a single diastereoisomer. To the best of our knowledge, this reactivity has not been previously reported in sequences promoted by visible light.^11^ We therefore tested its generality.

**Figure 2:**
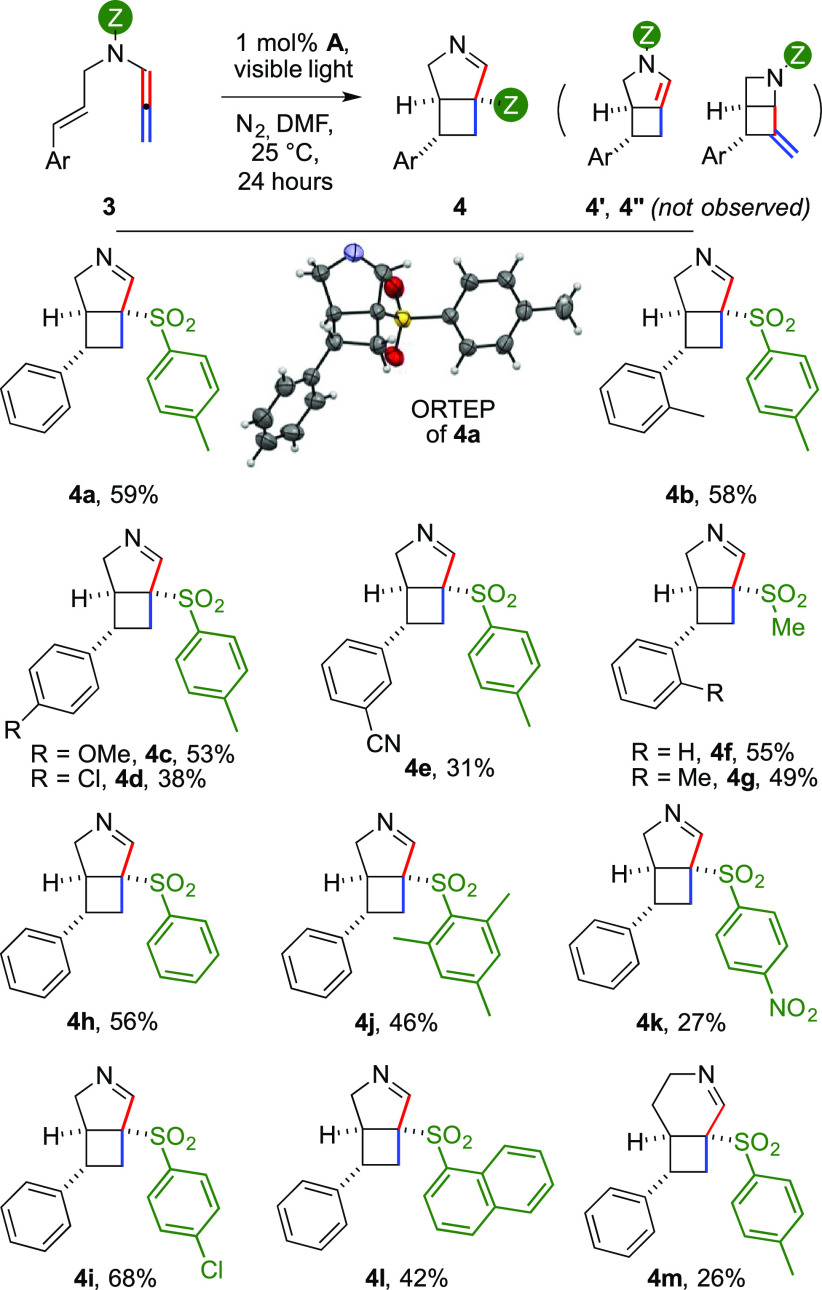
Synthesis of 3.2.0 heterobicycles **4**.

The method could be extended to different styryl partners, delivering the corresponding products **4b**–**e** in moderate to good yields. Electron-rich aromatics, both ortho- and para-substituted (**4b,c**, 53–58%), performed better than those with withdrawing groups (**4d,e**, 31–38%). The tosyl group could be replaced by a mesyl group (**4f,g**, 49–55%). Similarly, various arylsulfonyl groups were employed (**4h–l**), including bulky mesitylene and naphthalene units. The variation was well tolerated by the reaction (46–68%), with the partial exception of the nosyl derivative, which gave the corresponding product in 27% yield. A longer tether enabled us to access a tetrahydropyridine unit, albeit with a moderate yield (**4m**, 26%).

We then tried to prove whether the sulfonyl fragmentation/recombination occurred through either a uni- or a multimolecular pathway. A 1:1 mixture of enallenes **3b** and **3f** was thus reacted under optimized conditions. We analyzed the crude product by NMR and isolated nearly equimolar amounts of four products, in which aryls and sulfonyl groups were evenly scrambled (Scheme 3). This result shows that the rearrangement is a multimolecular process.

**Scheme 3:**
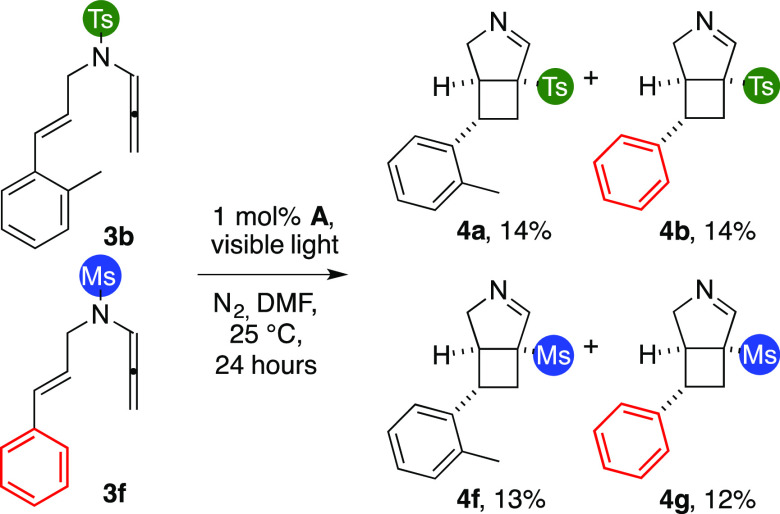
Double Tagging Experiment

On the basis of literature studies and experimental/computational studies, we propose the rationale presented in Figure 3 to account for the present complementary cyclization of enallenes.

**Figure 3:**
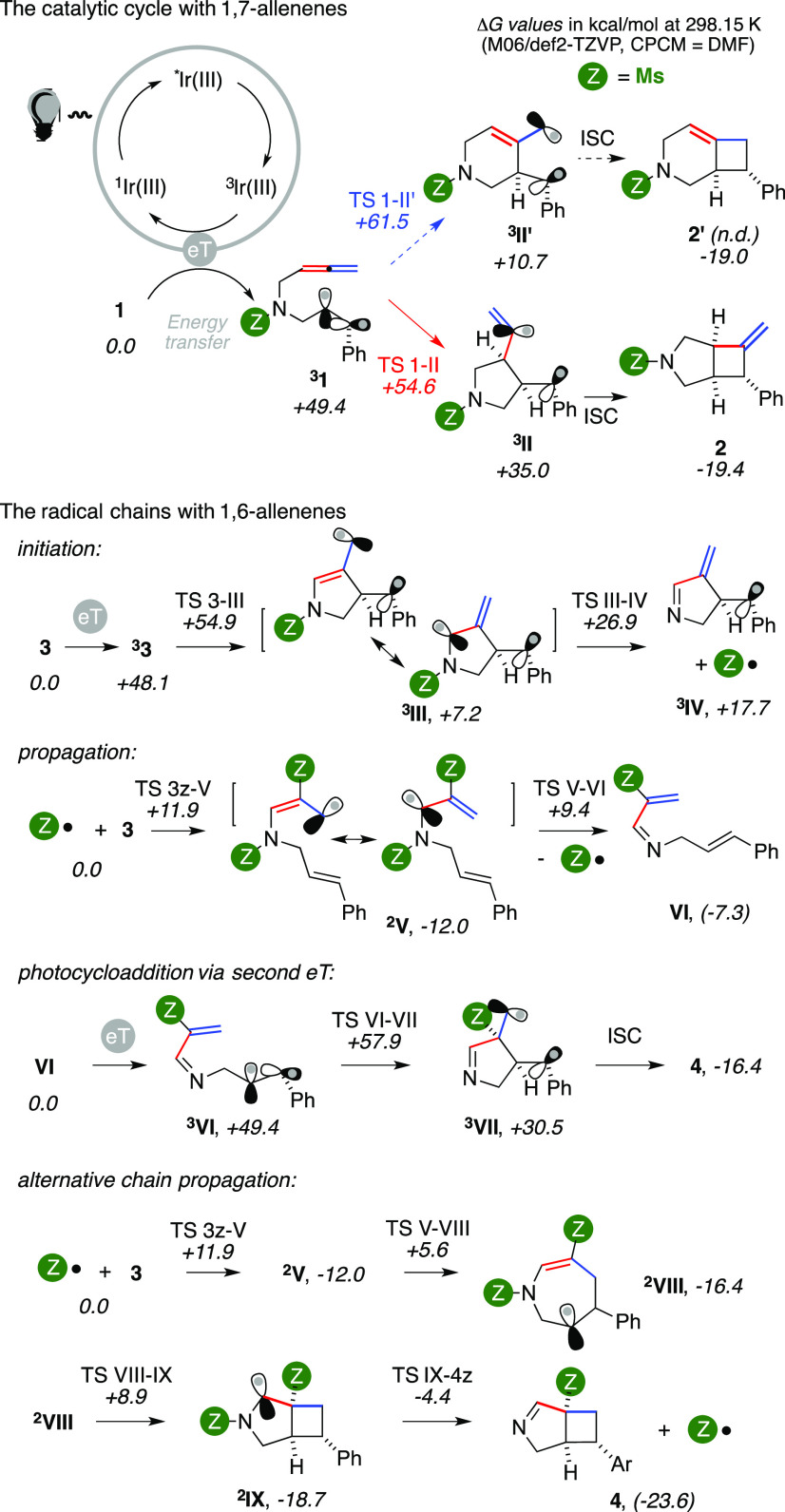
Proposed reaction mechanism.

Monosubstituted allenes have triplet energies that do not match that of the iridium complex. Their redox potentials are beyond those accessible using the present catalyst and visible light.^6^ The oxidation of styryl fragments, especially those with electron-withdrawing substituents, follows suit.^6a,8a^ On the contrary, the triplet state of the iridium catalyst could activate the vinylarene arm of substrates through an energy transfer (eT) process.^12^ This correlates with the absence of reactivity observed by replacing the photocatalyst with species that have triplet energies unable to activate β-styryl units, such as the popular Ru(bpy)_3_^2+^ complex. Upon eT, intermediate **^3^1** can then evolve through two different pathways, forming either a six- or a five-membered ring (^**3**^**II′** and ^**3**^**II**, respectively). The latter cyclization prevails, enabling the formation of vinylidenecyclobutane **2** upon intersystem crossing (ISC). According to density functional theory (DFT) modeling, this stems from both an easier transition state (TS) and the least stable exoergonic intermediate (ΔΔ*G* of −6.9 and +24.3 kcal/mol, respectively, at the M06/def2-TZVP level using DMF as an implicit solvent).

Analogous activation of the 1,6-enallene gives triplet ^**3**^**3**, for which steric factors disfavor the 4-exo cyclization that would have led to a 2.2.0 bicycle. The alternative formation of a five-membered ring delivers ^**3**^**III**. The cyclization can smoothly take place through a low barrier (Δ*G* of +6.8 kcal/mol) and thus parallels the results observed with 1,7-allenenes. In ^**3**^**III** the spin density on the allyl group is evenly spread, with a slight preference for the internal carbon atom. The two mono-occupied molecular orbitals are nearly perpendicular in ^**3**^**III**. This likely explains its reduced prowess toward the formation of the expected unobserved [2 + 2] cycloaddition product **4′**. However, the N–S bond of ^**3**^**III** is slightly longer than that of ^**3**^**3** (by +0.008 and +0.0005 Å using def2-SVP and def2-TZVP basis sets, respectively). This makes possible a relatively easy β-fragmentation, which provides ^**3**^**IV** by homolytic N–S bond cleavage. A significant basis set effect on calculated energies was observed for this TS (Δ*G* of +14.3 and +19.7 kcal/mol using def2-SVP and def2-TZVP, respectively; see the SI for a comparison of the basis sets and the Hartree–Fock contribution to functionals for this step).^13^ Nonetheless, even the least favorable calculated barrier is still viable for a homolytic bond cleavage.

The fate of the two radical fragments of ^**3**^**IV** posed several hurdles (see the SI for additional, less favorable pathways), until we considered a chain reaction.^14^ Substrate activation, cyclization, and fragmentation comprise the overall initiation of the process. The propagation involves the selective addition of a sulfonyl radical on the C(sp) carbon of a substrate molecule,^15^ affording allyl radical ^**2**^**V** (Δ*G* = −12.2 kcal/mol) through a low-lying TS (barrier of +11.9 kcal/mol in Δ*G*). The β-fragmentation of ^**2**^**V** occurs through a barrier of +21.4 kcal/mol in Δ*G*, and it gives intermediate **VI**, regenerating the sulfonyl radical. The former could then undergo a second eT, providing triplet ^**3**^**VI**, by analogy to the activation of **1** and **3** that is nearly isoenergetic. Triplet ^**3**^**VI** can smoothly evolve into ^**3**^**VII** via 5-exo-trig cyclization (barrier of +8.5 kcal/mol in Δ*G*). Product **4** eventually forms by intersystem crossing (ISC).

An alternative scenario involves a propagation leading directly to bicycle **4**. In this case, allyl radical ^**2**^**V** undergoes a 7-endo-trig cyclization that provides ^**2**^**VIII** upon a relative barrier of +17.6 kcal/mol in Δ*G*. The subsequent 4-exo-trig/5-endo-trig cyclization is the most energy-demanding step of the pathway (barrier of +24.9 kcal/mol in Δ*G*), and it gives bicyclic radical ^**2**^**IX**. The latter could then undergo β-fragmentation to provide product **4** and regenerate the sulfonyl radical. This step has a lower barrier compared with the analogous N–S bond cleavage taking place on ^**3**^**III** (ΔΔ*G* = −3.4 kcal/mol). Overall, the propagation of this chain reaction has a largely negative Δ*G* (−23.6 kcal/mol). All of its steps are exoergonic and, moreover, progressively more so. This should further increase the easiness of the propagation.

Starting from ^**2**^**V**, the highest energy TS of this manifold is just 0.5 kcal/mol lower in energy than that of the joint radical/photochemical mechanism, suggesting that the competition might be too close to call. The chain generating **VI** has a less favorable Δ*G* (−7.3 kcal/mol) than that directly affording **4** (−23.6 kcal/mol). This gap, coupled to the requirement of a second excited ^**3**^**Ir** species to afford the product,^8a,16^ seems to favor the odds of an entirely free-radical pathway.

In sharp contrast with the β-fragmentation of the C–S bond that is a routine tool in radical sequences, it is worth noting that that of α-sulfonamidoyl radicals has very few precedents.^17^ These are limited at the present to processes promoted by tin hydrides, which, furthermore, do not allow reincorporation of the sulfonyl fragment in the final product.

In conclusion, we reported the synthesis of two complementary families of 3.2.0 bicycles from enallenes. Both reactions show remarkable regio- and diastereocontrol, affording complex scaffolds under mild conditions. Mechanistic studies point out two orthogonal pathways that took place under identical conditions, namely, a [2 + 2] photocycloaddition versus a radical chain indirectly initiated by a photoexcited iridium complex. Although catalytic pathways and innate chains often compete in visible-light-promoted processes,^18^ we are unaware of synthetic methods in which they are similarly interconnected.

## Supporting Information Available

Experimental procedures, synthesis of reagents, characterization of products, modeling data, X-ray crystallography data, and copies of NMR spectra (PDF)

## Accession Codes

CCDC 2009276–2009278 contain the supplementary crystallographic data for this paper. These data can be obtained free of charge via www.ccdc.cam.ac.uk/data_request/cif, or by emailing data_request@ccdc.cam.ac.uk, or by contacting The Cambridge Crystallographic Data Centre, 12 Union Road, Cambridge CB2 1EZ, UK; fax: +44 1223 336033.
