# Supplementary material for: Orthogonal Syntheses of 3.2.0 Bicycles from Enallenes Promoted by Visible Light
Source: Org Lett. 2020 Aug 5;22(16):6354–9. doi: 10.1021/acs.orglett.0c02193 (PMC8010793; doi:10.1021/acs.orglett.0c02193)
Supplement: Supplementary file 1 — ol0c02193_si_001.pdf [file ol0c02193_si_001.pdf]

Supporting Information for

Orthogonal syntheses of 3.2.0 bicycles from enallenes promoted by visible light

Andrea Serafino,[a] Davide Balestri,[a] Luciano Marchiò,[a]
Max Malacria,[b] Etienne Derat,[b] Giovanni Maestri[a]

a Università di Parma, Department of Chemistry, Life Sciences and Environmental Sustainability, Parco Area delle Scienze 17/A, 43124 Parma, Italy

b Sorbonne Université, Faculty of Science and Engineering, CNRS, Institut Parisien de Chimie Moléculaire (UMR CNRS 8232), 4 place Jussieu, 75252 Paris Cedex 05, France

Table of contents

General remarks	3
Additional computational results	5
Selected optimization entries	7
Synthesis of substrates	9
Characterization of products	22
X-Ray crystallography data	33
Copies of NMR spectra	38
XYZ coordinates	94
Comprehensive table in AU	116
References	118

General remarks

All chemicals those syntheses are not reported hereafter were purchased from commercial sources and used as received. Solvents were dried passing through alumina columns using an Inert® system and were stored under nitrogen. Present visible-light promoted reactions did not required the use of dry solvents but presence of molecular oxygen exerts a negative effect on their rate. Chromatographic purifications were performed under gradient using a Combiflash® system and prepacked disposable silica cartridges or through isocratic flash chromatography using commercial 60 Å silica gel. All reactions that required heating were performed with the use of high-vacuum grade silicon oil. Reactions promoted by visible light were carried out in standard 4mm NMR tubes, surrounded by a commercial strip of 300 RGB household leds (12V) that were put a distance of ca 10 cm and switched on irradiating white light (model SMD5050-300 ip65). The tubes were inside an oil bath fitted with a thermometer to monitor the temperature of the solution. To avoid reproducibility issues, cooling is ensured by two fans recovered by outdated PCs. During summer time, solutions are kept at RT through the addition, inside the silicon oil bath, of a rubber spire linked to a chiller, which keeps pumping a cooled ethanol solution to maintain the desired temperature.

^{1}H and ^{13}C NMR spectra were recorded at 300 K on a Bruker 300 MHz, a Bruker 400 MHz or a Jeol 600 MHz spectrometers using residual non-deuterated solvents as internal standards (7.26 ppm for ^{1}H NMR and 77.00 ppm for ^{13}C-NMR for $CDCl_3$, 2.05 ppm for ^{1}H NMR and 29.84 ppm for ^{13}C NMR for acetone-d_6). ^{19}F-NMR spectra were recorded in $CDCl_3$ at 298 K on a Jeol 600 spectrometer fitted with a BBFO probehead at 564 MHz. The terms m, s, d, t, q and quint represent multiplet, singlet, doublet, triplet, quadruplet and quintuplet respectively, and the term br means a broad signal. Mass analyses were recorded on an Infusion Water Acquity Ultra Performance LC HO6UPS-823M instrument equipped with a SQ detector (Electrospray source); high-resolution mass analyses were recorded on a LTQ ORBITRAP XL Thermo Mass Spectrometer (Electrospray source).

CCDC 2009276, 2009277 and 2009278 contain the supplementary crystallographic data for compounds **2a**, **2'-l** and **4a**, respectively. Single crystal Data were collected with a Bruker D8 diffractometer equipped with PhotonII area detector, using a CuKα or a MoKα microfocus

radiation source. The data collection strategy covered the sphere of reciprocal space. Absorption corrections were applied using the program SADABS. The structure was solved with the SHELXT code. Fourier analysis and refinement were performed by the full-matrix least-squares methods based on F2 using SHELXL-2014 as implemented in Olex2. All the non-H atoms were refined with anisotropic displacement parameters.

Calculations were performed with Gaussian 09 at the DFT level.[1] The geometries of all species were optimized without any constraints at the generalized gradient approximation using the M06 hybrid functional of Zhao and Truhlar.[2] On key intermediates and TSs, reoptimization with the M06-2X functional were carried out to assess the influence of different Hartree-Fock contributions to calculated energies and geometries. Optimizations were carried out using the LACVP(d) basis set, which consists of the standard 6-31G(d) basis set for lighter atoms (H, C, N, O), and with the Def2-SVP(d) set defined by Weigand and Ahlrichs to check potential biases due to basis set effects.[3] On Def2-SVP(d) optimized geometries, complete re-optimization was performed employing a more refined triple-ζ basis set, namely the def2-TZVP(d) set.[3] These gas-phase structures were then freely optimized using DMF as an implicit solvent as implemented in Gaussian 09 through the CPCM approach.[4] The starting approximate geometries for transition states (TS) were obtained through scans of the relative reaction coordinate. Transition states were identified by the presence of a single imaginary frequency in the Hessian matrix, corresponding to the vibration along the reaction coordinate linking the reactant and the product. Conversely, stable intermediates were identified by the absence of imaginary frequencies in their Hessian matrix. Additional modeled pathways that proved less feasible are briefly presented hereafter. XYZ coordinates and a comprehensive table in atomic units including imaginary IR frequencies for transition states are in the dedicated section of present documents.

Additional computational results

- Less favorable reaction pathways

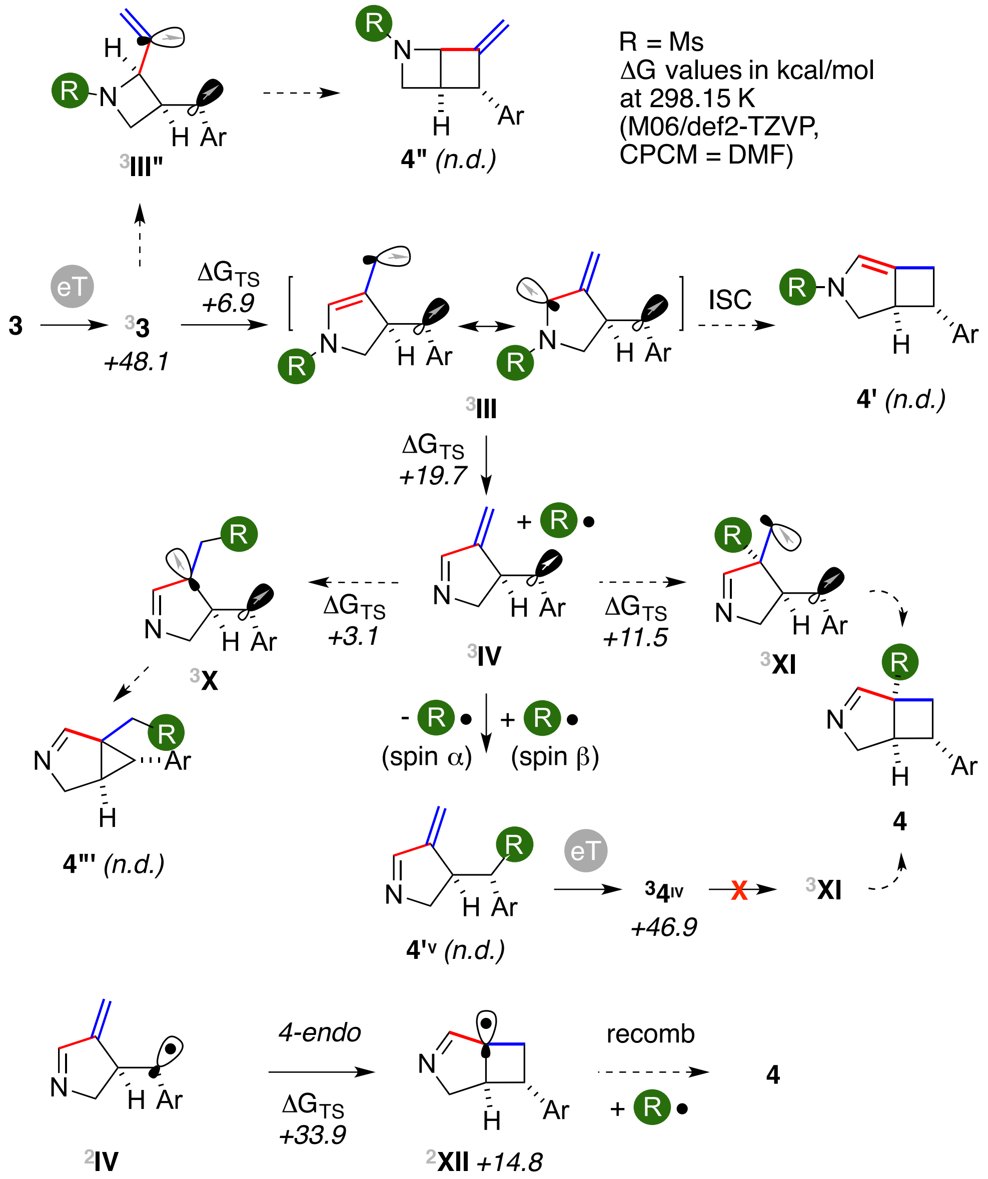

Radical addition on the C-C double bond of ^{3}IV can occur from its least hindered face. The two competing pathways have both low barriers (+3.1 and +11.5 kcal/mol, respectively), but that on the terminal carbon atom results way more favorable (by -8.2 kcal/mol in ΔΔG at the M06/Def2-tzvp level). This is due to both a reduced hindrance and a higher stabilization of the resulting α-imino tertiary radical in ^{3}X. Although this feature is consistent with ample literature precedents, it is starkly at odds with the recovery of product 4 and the absence of the 3.1.0 bicycle 4’”. None of them can either undergo further activation from PC1 via eT according to modeling. Recombination of the two fragments of triplet ^{3}III is forbidden if both have the same

spin. It is however conceivable, on the basis of the experimental findings revealing a multimolecular mechanism for the formal fragmentation/addition of the sulfonyl group, that formation of 4^{IV} could occur via recombination of two radicals with different spin. A second excitation of this putative intermediate could lead to the corresponding triplet $^{3}4^{IV}$. This energy gap is similar to those calculated for the reagent itself and seems therefore accessible under present condition (+46.9 kcal/mol in ΔG). Try as we might, we were however unable to find any accessible transition state for the subsequent 1,3-sulfonyl migration from the benzylic position to carbon atom that occupies the headbridge position in the final product 4. A similar reaction would have led to triplet ^{3}XI, which could have been delivered the final product upon ISC. Finally, an intramolecular *4-endo-trig* cyclyzation on radical ^{2}IV has been modeled. The calculated barrier is significantly high in energy (ΔG = +33.9 kcal/mol) and the resulting intermediate ^{2}XII is less stable than the entry channel (by +14.8 kcal/mol in ΔG). Although radical recombination of this species with a sulfonyl should be energetically favorable, modelled values for this *4-endo-trig* are significantly higher than those presented in the main article. This reduces the odds that a similar pathway takes place in present reactions.

- Basis set and Hartree-Fock contribution effects on N-S fragmentation

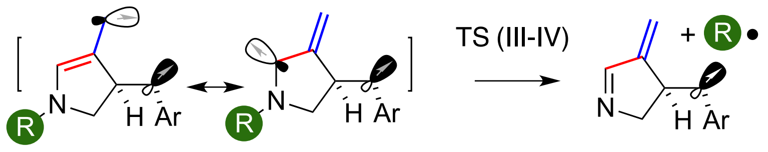

^{3}**III** ^{3}**IV**

The fragmentation of ^{3}III and the corresponding TS (III-IV) have been modeled using three different basis sets (def2-SVP, def-TZVP and lacvp(d)) and two different functionals with different Hartree-Fock contributions (M06 and M06-2X). In contrast to the other steps of present reactions, a higher than usual difference (up to 8 kcal/mol in ΔG) emerged among these TSs. This seems to correlate with the calculated N-S bond length in intermediate ^{3}III. Although variation ranges 0.01-0.03 angstroms, a slightly longer bond in the intermediate leads to lower calculated barrier for the fragmentation. The following table sum up calculated data for these different systems.

CPCM (DMF)							
M06/def2-SVP	H (Hartrees)	S (cal/K*mol)	Imaginary freq (1/cm)	ΔH	ΔS	ΔG	Bond lenght (Ang)
^{3}III	-1106.243898	134.23					NS = 1.67917
TS (III-IV)	-1106.220223	135.973	-313.6115	14.86	1.743	14.34	
M062X/def2-SVP							
^{3}III	-1106.416324	131.045					NS = 1.66719
TS (III-IV)	-1106.389236	136.275	-383.6849	17.00	5.230	15.44	
M06/def2-TZVP							
^{3}III	-1107.217777	133.285					NS = 1.64974
TS (III-IV)	-1107.185694	134.697	-280.4895	20.13	1.412	19.71	
M062X/def2-TZVP							
^{3}III	-1107.409185	130.902					NS = 1.64303
TS (III-IV)	-1107.372658	134.440	-291.8977	22.92	2.538	22.16	
M06/lacvp(d)							
^{3}III	-1106.875924	134.063					NS = 1.67725
TS (III-IV)	-1106.856009	135.658	-286.159	12.50	1.595	12.02	
M062X/lacvp(d)							
^{3}III	-1107.050056	130.835					NS = 1.66768
TS (III-IV)	-1107.028059	134.555	-365.0586	13.80	3.720	12.69	

Selected optimization entries

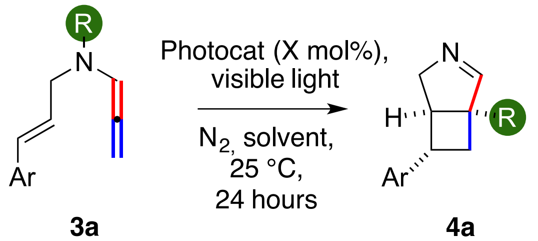

3a **4a**

entry	Photocat (mol%)	Dry solvent (M of **3a**)	Yield of **4**[b]
1	Complex **A** (2.5%)	DMF (0.2)	41%
2[a]	Complex **A** (2.5%)	DMF (0.2)	40%
3	Complex **A** (1%)	DMF (0.2)	43%
4	Complex **A** (0.5%)	DMF (0.2)	37%
5	$Ir(ppy)_3$ (1%)	DMF (0.2)	36% (28%)
6	$Ru(bpy)_3Cl_2$ (2.5%)	DMF (0.2)	--
7	Complex **A** (1%)	Toluene/DCM 3:1 (0.20)	52%
8	Complex **A** (1%)	Toluene/DCM 3:1 (0.05)	62%
9	Complex **A** (1%)	DMF (0.05)	68 % (59%)
10	Ir(p-F-ppy)$_3$ (1%)	Toluene (0.05)	54%

Reaction conditions: 0.1 mmol of **3a**, photocatalyst (X mol%), solvent (0.05-0.2 M), in a 5 mm NMR tube at 25 °C for 24 hours without stirring; [a] at 45° C; [b] ^{1}H NMR yield using trimethoxybenzene as internal standard, isolated yields in parentheses.

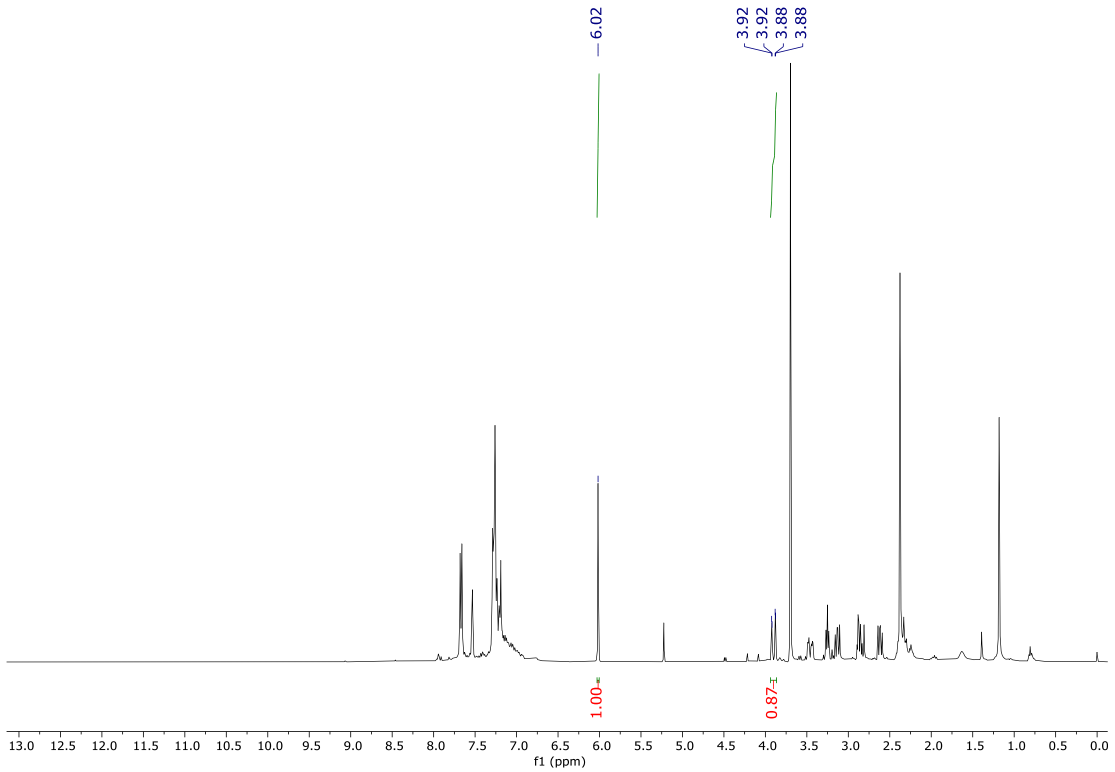

Copy of the ^{1}H NMR spectrum of the crude reaction mixture from entry 9. Trimethoxybenzene (2.36 mg, 0.026 mmol, left peak) vs product (right peak). Beside **4a**, we were unable to identify any other well-defined byproduct of the reaction, although partial broadening of the aromatic region suggests that partial oligo-/polymerization of the enallene occurred.

Synthesis of substrates

Synthesis of 1,6-enynes

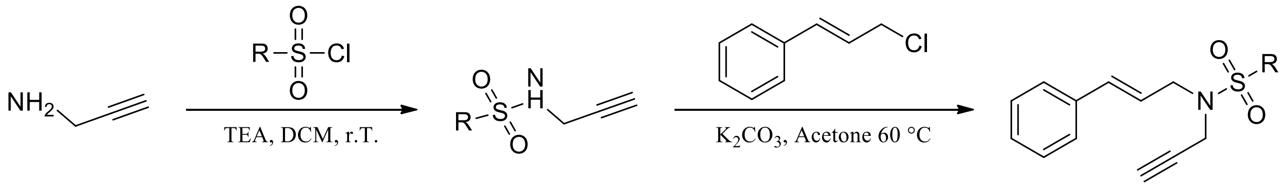

DCM (0.4 M), TEA (1.1 eq) and propargyl amine (1 equiv.) were added to a 100 mL round bottom flask equipped with magnetic stirring bar. The resulting mixture was stirred at 0 °C and sulfonyl halide (1.05 eq) was added slowly. The reaction mixture was stirred at room temperature for 12 hours, quenched with sat NH_4Cl, diluted with DCM and washed with water. The organic layer was dried over Na_2SO_4, filtered and concentred under reduced pressure. Whitout isolation, propargyl tosylamide was transferred in a Schlenk tube equipped with a magnetic stirring bar and K_2CO_3 (1.5 equiv.). Acetone (0.33 M) and cinnamyl chloride (1.3 equiv.) were then added. The reaction mixture was stirred at 60 °C overnight. The mixture was cooled to room temperature, diluted with water and extracted with EtOAc (3 x 20 mL). The combined organic layers were dried over Na_2SO_4, filtered and concentred under reduced pressure. The resulting crude was purified by chromatography on silica gel (*n-hexane*/EtOAc 9:1).

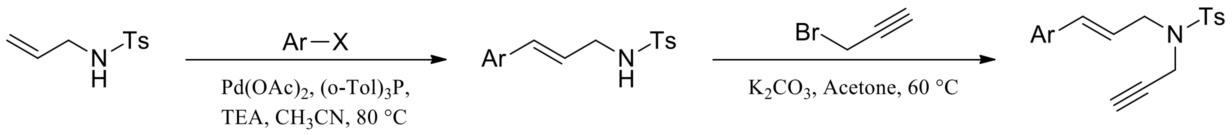

X=I, Br

Ar=4-chlorophenyl
4-(trifluoromethyl)phenyl
3-fluorophenyl
2-methylphenyl
4-methoxyphenyl

N-allyl-4-methylbenzenesulfonamide (1 equiv.), (o-Tol)$_3$P (0.1 equiv.) and Pd(OAc)$_2$ (0.05 equiv.) were sequentially added to a Schlenk tube equipped with magnetic stirring bar. CH_3CN (0.41 M), TEA (2 equiv.) and the desired aryl halide (1 equiv.) were added under N_2 atmosphere and the mixture was stirred at 80 °C for 3 hours. A second batch of the desired aryl halide (0.42 equiv.), Pd(OAc)$_2$ (0.026 equiv.) and (o-Tol)$_3$P (0.05 equiv.) were then added. The mixture was stirred at 80 °C for further 6 hours, allowed to cool to room temperature, diluted with water and extracted with EtOAc (3 x 30 mL). The combined organic layers were dried over Na_2SO_4, filtered and concentred under reduced pressure. The resulting crude was purified by chromatography on silica gel (*n-hexane*/EtOAc 9:1). The substituted cinnamyl tosylamide (1 equiv.) was dissolved in acetone (0.2 M). K_2CO_3 (3 equiv.) and propargyl bromide (85% in toluene, 1.5 equiv.) were then added. The mixture was subsequently placed in a pre-heated oil bath at 60 °C and stirred overnight. After consumption of the starting material, the reaction mixture was cooled down to room temperature and water was added. The mixture was extracted with EtOAc (3 x 30 mL). The combined organic fractions were dried over Na_2SO_4

and concentrated under reduced pressure. The resulting crude was purified by chromatography on silica gel (*n-hexane*/EtOAc 8:2).

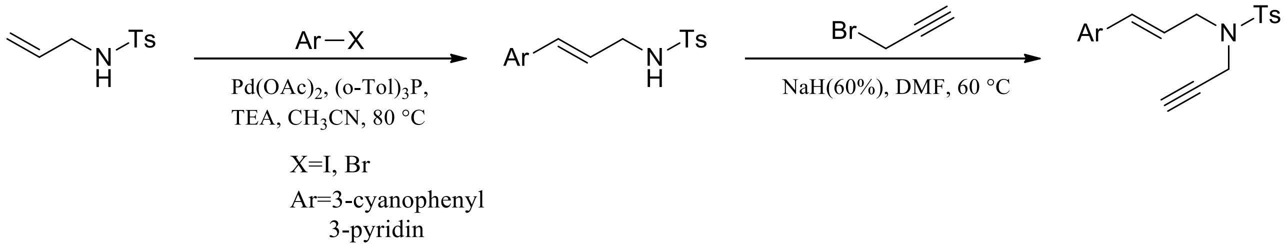

The Heck-type coupling was performed according to the above-mentioned procedure.
Then, the resulting tosylamide (1 equiv.) was dissolved in DMF (0.2 M). NaH (60% in mineral oil, 1.3 equiv.) was added slowly at 0° C under vigorous stirring. The resulting mixture was stirred for 1 hour at room temperature prior to the addition of a propargyl bromide solution (85% in toluene, 1.5 equiv.). The resulting mixture was then heated at 60° C for 2 hours. After completion, the reaction mixture was cooled down to room temperature and water was added. The mixture was extracted with EtOAc (3 x 30 mL) and the organic layers washed with a sat. LiCl solution. The combined organic fractions were dried over Na_2SO_4 and concentrated under reduced pressure. The resulting crude was purified by chromatography on silica gel (*n-hexane*/EtOAc 8:2).

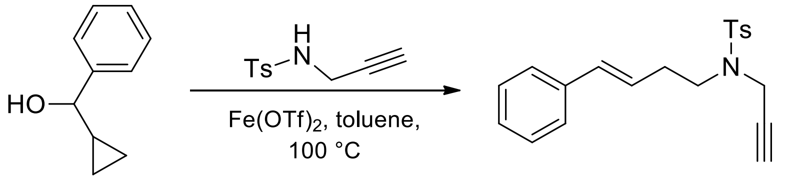

$Fe(OTf)_2$ (0.1 equiv.) and toluene (0.17 M) were added to a Schlenk tube equipped with magnetic stirring bar. The resulting mixture was stirred at room temperature for 10 minutes prior to the addition of a solution of cyclopropyl(phenyl)methanol (1 equiv.) and 4-methyl-N-(prop-2-yn-1-yl)benzenesulfonamide (4 equiv.) in toluene (0.17 M). The mixture was stirred at 100 °C and conversion was monitoring by TLC. The reaction mixture was then cooled and the solvent removed under reduced pressure. The resulting crude was purified by chromatography on silica gel (*n-hexane*/EtOAc 9:1).

Synthesis of 1,7-enallenes

General procedure 1a (GP-1a)

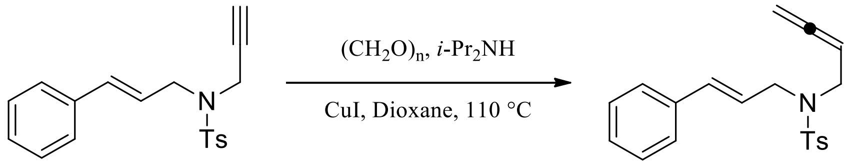

CuI (0.1 equiv.), paraformaldehyde (1.6 equiv.), the desired 1,6-enyne (1 equiv.) and dioxane (1.5 M) were sequentially added to a 50 mL two-necked round-bottom flask equipped with a magnetic stirring. The resulting mixture was stirred at room temperature for 10 minutes prior to the addition of *i*Pr_2NH (1.4 equiv.). The mixture was then heated to 110 °C for 12 hours

under air and then cooled down to room temperature. A sat. NH_4Cl solution was added. The mixture was diluted with water and extracted with EtOAc (3 x 20 mL). The combined organic layers were dried over Na_2SO_4, filtered and concentred under reduced pressure. The resulting crude was purified by chromatography on silica gel (*n-hexane*/EtOAc 95:5).

General procedure 1b (GP-1b)

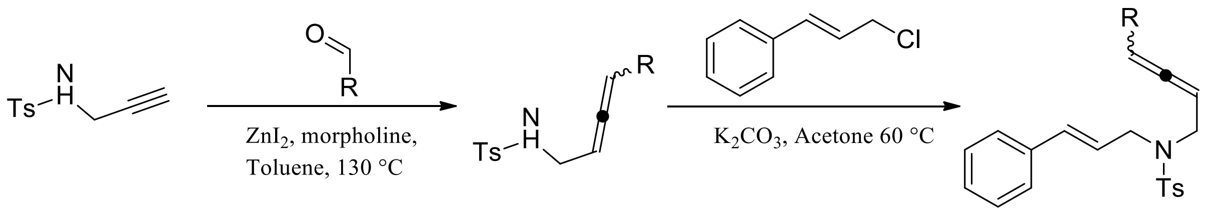

R= Et, Ar

ZnI_2 (0.8 equiv.), the desired aldehyde (1.8 equiv.), 4-methyl-N-(prop-2-yn-1-yl)benzenesulfonamide (1 equiv.) and toluene (0.33 M) were sequentially added to a 50 mL two-necked round-bottom flask equipped with a magnetic stirring bar. The resulting mixture was stirred at room temperature for 10 minutes prior to the addition of morpholine (1.4 equiv.). The mixture was then heated to 110 °C for 12 hours under air and then cooled down to room temperature. A saturated NH_4Cl solution was added. The mixture was diluted with water and extracted with EtOAc (3 x 20 mL). The combined organic layer was dried over Na_2SO_4, filtered and concentred under reduced pressure. The resulting crude was purified by chromatography on silica gel (*n-hexane*/EtOAc 8:2). The intermediate was dissolved in acetone (0.33 M). K_2CO_3 (1.5 equiv.) and cinnamylchloride (1.3 equiv.) were sequentially added. The mixture was stirred at 60 °C monitoring the conversion by TLC. Upon full conversion, the mixture was diluted with EtOAc (20 mL) and washed with water. The combined organic layers were dried over Na_2SO_4, filtered and concentred under reduced pressure. The resulting crude was purified by chromatography on silica gel (*n-hexane*/EtOAc 95:5).[5]

Synthesis of 1,6-enallenes

General procedure 2 (GP-2)

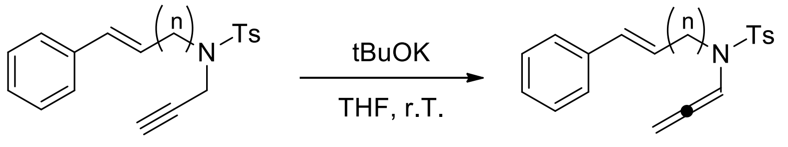

n = 1,2

The desired alkyne (1 equiv.) and THF (0.17 M) were sequentially added to a Schlenk tube equipped with a magnetic stirring bar. the resulting mixture was stirred at room temperature for 10 minutes prior to the addition of tBuOK (0.3 equiv.). After complete conversion as monitored by TLC, a saturated NH_4Cl solution (15 ml) was added. The mixture was extracted with EtOAc (3 x 15 mL), the organic layers separated and dried over Na_2SO_4. The solution was concentrated under reduced pressure and the crude purified by chromatography on silica gel (*n-hexane*/EtOAc 95:5).

N-(buta-2,3-dien-1-yl)-N-cinnamyl-4-methylbenzenesulfonamide (1a)

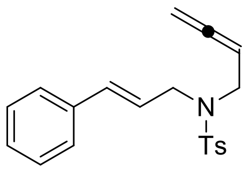

The general procedure (**GP-1a**) was followed using the correspondent enyne (600mg, 1.85 mmol). Purification by chromatography on silica gel yielded **1a** (182.7 mg, 29%) as a white solid. **^{1}H NMR (400 MHz, $CDCl_3$)** δ = 7.71 (d, *J* = 8.3 Hz, 2H), 7.50 – 6.73 (m, 7H), 6.43 (d, *J* = 15.8 Hz, 1H), 5.96 (dt, *J* = 15.8, 6.8 Hz, 1H), 4.91 (p, *J* = 6.9 Hz, 1H), 4.67 (dt, *J* = 6.6, 2.5 Hz, 2H), 3.98 (d, *J* = 6.8, Hz, 2H), 3.87 (dt, *J* = 7.2, 2.5 Hz, 2H), 2.40 (s, 3H). **^{13}C NMR (101 MHz, $CDCl_3$)** δ = 209.6 (Cq), 143.4 (Cq), 137.5 (Cq), 136.3 (Cq), 134.2 (CH), 129.8 (CH), 128.6 (CH), 127.9 (CH), 127.3 (CH), 126.5 (CH), 123.7 (CH), 85.8 (CH), 76.3 (CH_2), 48.9 (CH_2), 45.8 (CH_2), 21.6 (CH_3). **(ESI)-HRMS** calcd for $C_{20}H_{22}NO_2S$ $[M+H]^+$ 340.1366, found 340.1374.

(E)-N-(buta-2,3-dien-1-yl)-N-(3-(4-chlorophenyl)allyl)-4-methylbenzenesulfonamide (1b)

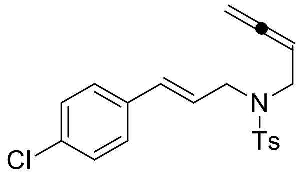

The general procedure (**GP-1a**) was followed using the correspondent enyne (312 mg, 0.87 moml). Purification by chromatography on silica gel yielded **1b** (197 mg, 61%) as a white solid. **^{1}H NMR (300 MHz, acetone)** δ = 7.83 – 7.73 (m, 2H), 7.48 – 7.27 (m, 6H), 6.56 (dt, *J* = 15.8, 1.5 Hz, 1H), 6.10 (dt, *J* = 15.9, 6.7 Hz, 1H), 4.99 (p, *J* = 6.9 Hz, 1H), 4.75 (dt, *J* = 6.6, 2.5 Hz, 2H), 4.02 (d, *J* = 6.6 Hz, 2H), 3.90 (dt, *J* = 7.1, 2.6 Hz, 2H), 2.42 (s, 3H). **^{13}C NMR (75 MHz, acetone)** δ = 209.5 (Cq), 143.4 (Cq), 137.8 (Cq), 135.5 (Cq), 132.9 (Cq), 132.4 (CH), 129.8 (CH), 128.6 (CH), 128.0 (CH), 127.3 (CH), 125.1 (CH), 85.6 (CH), 75.6 (CH_2), 48.7 (CH_2), 45.9 (CH_2), 20.6 (CH_3). **(ESI)-HRMS** calcd for $C_{20}H_{20}ClNNaO_2S$ $[M+Na]^+$ 396.0795, found 396.0791.

(E)-N-(buta-2,3-dien-1-yl)-4-methyl-N-(3-(4-(trifluoromethyl)phenyl)allyl)benzenesulfonamide (1c)

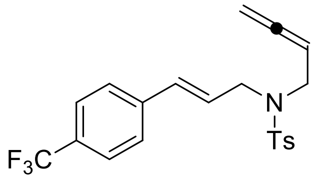

The general procedure (**GP-1a**) was followed using the correspondent enyne (310 mg, 0.82 mmol). Purification by chromatography on silica gel yielded **1c** (212 mg, 66%) as a white solid. **^{1}H NMR (400 MHz, $CDCl_3$)** δ = 7.76 – 7.70 (m, 2H), 7.55 (d, *J* = 8.1 Hz, 2H), 7.37 (d, *J* = 8.1 Hz, 2H), 7.30 (d, *J* = 8.0 Hz, 2H), 6.50 (d, *J* = 15.9 Hz, 1H), 6.12 (dt, *J* = 15.9, 6.6 Hz, 1H), 4.96 (p, *J* = 6.8 Hz, 1H), 4.69 (dt, *J* = 6.5, 2.5 Hz, 2H), 4.02 (d, *J* = 6.6 Hz, 2H), 3.89 (dt, *J* = 7.1, 2.5 Hz, 2H), 2.42 (s, 3H). **^{13}C NMR (101 MHz, $CDCl_3$)** δ = 209.7 (Cq), 143.5 (Cq), 139.8 (Cq), 137.3 (Cq), 132.4 (CH), 129.8 (CH), 129.6 (Cq, *J* = 32.1 Hz), 127.2 (CH), 126.8 (CH), 126.6 (CH), 125.5 (Cq, *J* = 4.1 Hz), 124,1 (Cq, *J* = 271.5 Hz), 85.7 (CH), 76.3 (CH_2), 48.7 (CH_2), 46.2 (CH_2), 21.5 (CH_3). **^{19}F NMR**

(564 MHz, $CDCl_3$) δ = -62.44. **(ESI)-HRMS** calcd for $C_{21}H_{20}F_3NNaO_2S$ $[M+Na]^+$ 430.1059, found 430.1048.

(E)-N-(buta-2,3-dien-1-yl)-N-(3-(3-fluorophenyl)allyl)-4-methylbenzenesulfonamide (1d)

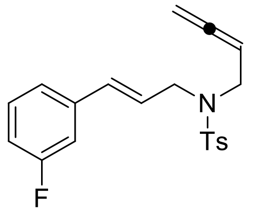

The general procedure (**GP-1a**) was followed using the correspondent enyne (240 mg, 0.7 mmol). Purification by chromatography on silica gel yielded **1d** (102 mg, 41%) as a white solid. **^{1}H NMR (400 MHz, $CDCl_3$)** δ = 7.75 (d, *J* = 8.2 Hz, 2H), 7.37 – 7.23 (m, 3H), 7.06 (d, *J* = 7.7 Hz, 1H), 7.00 – 6.90 (m, 2H), 6.44 (d, *J* = 15.8 Hz, 1H), 6.01 (dt, *J* = 15.8, 6.6 Hz, 1H), 4.97 (p, *J* = 6.8 Hz, 1H), 4.72 (dt, *J* = 6.5, 2.5 Hz, 1H), 4.72 (dt, *J* = 6.5, 2.5 Hz, 2H), 4.03 (d, *J* = 6.7 Hz, 1H), 3.94 – 3.88 (m, 2H), 2.45 (s, 3H). **^{13}C NMR (101 MHz, $CDCl_3$)** δ = 209.8 (Cq), 163.0 (Cq, d, *J* = 245.4 Hz), 143.4 (Cq), 138.6 (Cq, d, *J* = 7.9 Hz), 137.5 (Cq), 132.8 (d, *J* = 2.3 Hz, CH), 130.0 (d, *J* = 8.5 Hz, CH), 129.8 (CH), 127.24 (CH), 125.3 (CH), 122.3 (d, *J* = 2.9 Hz, CH), 114.7 (d, *J* = 21.3 Hz, CH), 112.9 (d, *J* = 22.0 Hz, CH), 85.8 (CH), 76.2 (CH_2), 48.7 (CH_2), 46.0 (CH_2), 21.5 (CH_3). **^{19}F NMR (564 MHz, $CDCl_3$)** δ = -113.24. **(ESI)-HRMS** calcd for $C_{20}H_{20}FNNaO_2S$ $[M+Na]^+$ 380.1091, found 380.1100.

(E)-N-(buta-2,3-dien-1-yl)-N-(3-(3-cyanophenyl)allyl)-4-methylbenzenesulfonamide (1e)

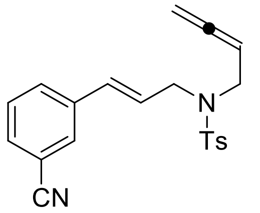

The general procedure (**GP-1a**) was followed using the correspondent enyne (245 mg, 0.7 mmol). Purification by chromatography on silica gel yielded **1e** (100 mg, 39%) as a white solid. **^{1}H NMR (400 MHz, $CDCl_3$)** δ = 7.72 (d, *J* = 7.9 Hz, 2H), 7.51 (d, *J* = 8.3 Hz, 3H), 7.45 – 7.36 (m, 1H), 7.31 (d, *J* = 7.9 Hz, 2H), 6.44 (d, *J* = 15.9 Hz, 1H), 6.13 – 6.01 (m, 1H), 4.94 (p, *J* = 6.8 Hz, 1H), 4.74 – 4.66 (m, 2H), 4.01 (d, *J* = 6.5 Hz, 2H), 3.88 (dd, *J* = 6.6, 3.1 Hz, 2H), 2.43 (s, 3H). **^{13}C NMR (101 MHz, $CDCl_3$)** δ = 209.7 (Cq), 143.6 (Cq), 137.6 (Cq), 137.4 (Cq), 131.5 (CH), 131.1 (CH), 130.5 (CH), 129.9 (CH), 129.8 (CH), 129.4 (CH), 127.2 (CH), 127.1 (CH), 118.6 (Cq), 112.9 (Cq), 87.2 (CH), 76.3 (CH_2), 48.6 (CH_2), 46.3 (CH_2), 21.5 (CH_3). **(ESI)-HRMS** calcd for $C_{21}H_{20}N_2NaO_2S$ $[M+Na]^+$ 387.1138 found 387.1131.

(E)-N-(buta-2,3-dien-1-yl)-4-methyl-N-(3-(m-tolyl)allyl)benzenesulfonamide (1f)

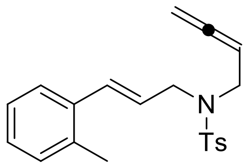

The general procedure (**GP-1a**) was followed using the correspondent enyne (66.3 mg, 0.19 mmol). Purification by chromatography on silica gel yielded **1f** (41.5 mg, 63%) as a white solid. **^{1}H NMR (400 MHz, $CDCl_3$)** δ = 7.74 (d, *J* = 8.3 Hz, 2H), 7.34 – 7.24 (m, 3H), 7.21 – 7.09 (m, 3H), 6.68 (d, *J* = 15.7 Hz, 1H), 5.86 (dt, *J* = 15.7, 6.8 Hz, 1H), 5.01 – 4.90 (m, 1H), 4.71 (dt, *J* = 6.7, 2.5 Hz, 2H), 4.03 (d, *J* = 6.9 Hz, 2H), 3.91 (dt, *J* = 7.2, 2.5 Hz, 2H), 2.43 (s, 3H), 2.29 (s, 3H). **^{13}C NMR (101 MHz, $CDCl_3$)** δ = 207.8 (Cq), 143.4 (Cq), 137.6 (Cq), 135.4 (Cq), 135.4 (CH) 132.2 (CH), 130.3 (Cq), 129.8 (CH), 127.8 (CH), 127.2 (CH), 126.1 (CH), 125.8 (CH), 125.0 (CH), 85.8 (CH), 75.4 (CH_2), 49.0 (CH_2), 45.6 (CH_2), 21.5 (CH_3), 19.8 (CH_3). **(ESI)-HRMS** calcd for $C_{21}H_{23}NKO_2S$ $[M+K]^+$ 392.1081, found 392.1074.

(E)-N-(buta-2,3-dien-1-yl)-N-(3-(4-methoxyphenyl)allyl)-4-methylbenzenesulfonamide (1g)

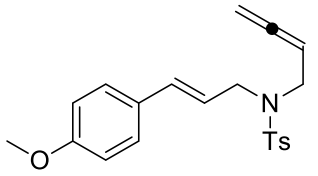

The general procedure (**GP-1a**) was followed using the correspondent enyne (169 mg, 0.47 mmol). Purification by chromatography on silica gel yielded **1g** (127.5 mg, 73%) as a white solid **^{1}H NMR (400 MHz, $CDCl_3$)** δ = 7.73 (d, *J* = 8.3 Hz, 2H), 7.30 (d, *J* = 7.7 Hz, 2H), 7.25 – 7.17 (m, 2H), 6.89 – 6.78 (m, 2H), 6.40 (d, *J* = 15.8 Hz, 1H), 5.84 (dt, *J* = 15.8, 6.8 Hz, 1H), 4.93 (p, *J* = 6.9 Hz, 1H), 4.69 (dt, *J* = 6.6, 2.5 Hz, 2H), 3.98 (d, *J* = 6.8 Hz, 2H), 3.89 (dt, *J* = 7.1, 2.5 Hz, 2H), 3.80 (s, 3H), 2.42 (s, 3H). **^{13}C NMR (101 MHz, $CDCl_3$)** δ = 209.6 (Cq), 159.4 (Cq), 143.3 (Cq), 137.6 (Cq), 133.7 (CH), 129.7 (CH), 129.0 (Cq), 127.7 (CH), 127.2 (CH), 121.3 (CH), 114.0 (CH), 85.8 (CH_2), 76.2 (CH), 55.3 (CH_3), 49.0 (CH_2), 45.6 (CH_2), 21.6 (CH_3). **(ESI)-HRMS** calcd for $C_{21}H_{23}KNO_3S$ $[M+K]^+$ 408.1030, found 408.1042.

(E)-N-(buta-2,3-dien-1-yl)-4-methyl-N-(3-(pyridin-3-yl)allyl)benzenesulfonamide (1h)

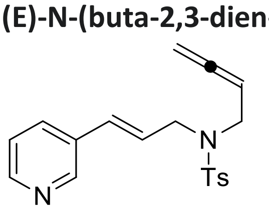

The general procedure (**GP-1a**) was followed using the correspondent enyne (200 mg, 0.61 mmol). Purification by chromatography on silica gel yielded **1h** (140 mg, 67 %) as a white solid. **^{1}H NMR (400 MHz, $CDCl_3$)** δ = 8.02 – 7.54 (m, 3H), 7.30 (d, *J* = 8.0 Hz, 2H), 6.54 (d, *J* = 15.5 Hz, 1H), 6.14 – 6.03 (m, 1H), 4.93 (p, *J* = 6.9 Hz, 1H), 4.69 (dt, *J* = 6.6, 2.4 Hz, 2H), 4.03 (d, *J* = 6.1 Hz, 2H), 3.89 (dt, *J* = 7.2, 2.4 Hz, 2H), 2.42 (s, 3H). **^{13}C NMR (101 MHz, $CDCl_3$)** δ = 209.6 (Cq), 143.5 (Cq), 137.3 (Cq), 129.8 (CH), 127.2 (CH), 126.9 (CH), 85.7 (CH), 76.4 (CH_2), 48.8 (CH_2), 46.1 (CH_2), 21.6 (CH_3). Resonances of nuclei from the 3-pyridyl unit were almost undetectable

even with long relaxation times. **(ESI)-HRMS** calcd for $C_{19}H_{21}N_2O_2S$ $[M+H]^+$ 341.1324, found 341.1328.

(E)-(3-(buta-2,3-dien-1-yloxy)prop-1-en-1-yl)benzene (1j)

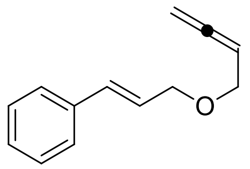

CuBr (103.8 mg, 0.72 mmol), paraformaldehyde (109 mg, 3.62 mmol), the 1,6-enyne (250 mg, 1.45 mmol) and dioxane (5.3 mL) were sequentially added to a 50 mL two-necked round-bottom flask equipped with a magnetic stirring. The resulting mixture was stirred at room temperature for 10 minutes prior to the before of *i*Pr_2NH (410 µL, 2.90 mmol). The mixture was then warmed to 110 °C under air for 12 hours. The reaction mixture was cooled to room temperature and a saturated NH_4Cl solution was added. The mixture was diluted with water and extracted with dietyl ether (3 x 10 mL). The combined organic layers were dried over Na_2SO_4, filtered and concentred under reduced pressure. The resulting crude was purified by chromatography on silica gel (*n-hexane*/EtOAc 97:3), which afforded **1j** (120.1 mg, 44%) as a yellow liquid. Spectroscopic data correspond to the literature.[6]
^{1}H NMR (400 MHz, CDCl$_3$) δ = 7.50 – 7.20 (m, 5H), 6.66 (d, *J* = 15.9 Hz, 1H), 6.34 (dt, *J* = 15.9, 6.1 Hz, 1H), 5.33 (p, *J* = 6.8 Hz, 1H), 4.86 (dt, *J* = 6.6, 2.4 Hz, 2H), 4.22 (dd, *J* = 6.1, 1.5 Hz, 2H), 4.12 (dt, *J* = 7.0, 2.5 Hz, 2H). **^{13}C NMR (101 MHz, CDCl$_3$)** δ = 209.4 (Cq), 136.7 (Cq), 132.7 (CH), 128.6 (CH), 127.7 (CH), 126.5 (CH), 125.8 (CH), 87.8 (CH), 75.8 (CH_2), 70.5 (CH_2), 67.9 (CH_2). **HRMS** calcd for $C_{19}H_{14}NaO$ $[M+Na]^+$ 209.0937, found 209.0926.

N-cinnamyl-N-(3-cyclohexylideneallyl)-4-methylbenzenesulfonamide (1k)

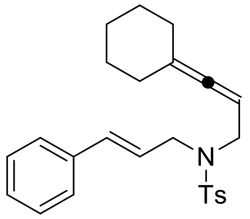

CuBr (28.6 mg, 0.2 mmol), toluene (2 mL), 4-methyl-N-(prop-2-yn-1-yl)benzenesulfonamide (420 mg, 2.0 mmol) and cyclohexanone (239 mg, 2.2 mmol) were sequentially added to a Schlenk tube kept under a nitrogen atmosphere. The resulting mixture was stirred at room temperature for 10 minutes prior to the addition of pyrrolidine (182 µL, 2.2 mmol), and then heated at 100° C. After 3 hours, the reaction mixture was cooled and filtered through a short pad of silica gel, eluting with acetone (70 mL). The crude product was then dissolved in toluene (6 mL) and transferred to a Schlenk tube containing ZnI_2 (191.4 mg, 1.2 mmol). The tube was placed in a pre-heated oil bath at 120° C and kept under stirring for 7 hours. Upon complete conversion monitored by TLC, the crude reaction mixture was filtrated through a short pad of silica gel, eluting with acetone (50 mL). The resulting crude was purified by chromatography on silica gel (*n-hexane*/EtOAc 9:1), which afforded N-(3-cyclohexylideneallyl)-4-methylbenzenesulfonamide (148.0 mg, 25 %) as a white solid.[7] N-(3-cyclohexylideneallyl)-4-methylbenzenesulfonamide was transferred in a Schlenk tube equipped with a magnetic

stirring bar and K_2CO_3 (105 mg, 0.76 mmol), acetone (1.5 mL) and cinnamyl chloride (103 µL, 0.66 mmol) were then added sequentially. The reaction mixture was stirred at 60 °C overnight. The mixture was cooled to room temperature, diluted with water and extracted with EtOAc (3 x 10 mL). The combined organic layers were dried over Na_2SO_4, filtered and concentred under reduced pressure. The resulting crude was purified by chromatography on silica gel (*n-hexane*/EtOAc 9:1), which afforded **1k** (152 mg, 70%) as a white solid. **^{1}H NMR (400 MHz, $CDCl_3$)** δ = 7.74 (d, *J* = 8.2 Hz, 2H), 7.34 – 7.20 (m, 7H), 6.44 (d, *J* = 15.8 Hz, 1H), 5.98 (dt, *J* = 15.8, 6.7 Hz, 1H), 4.75 (tt, *J* = 6.8, 2.0 Hz, 1H), 4.03 (dd, *J* = 6.5, 1.3 Hz, 2H), 3.84 (d, *J* = 6.9 Hz, 2H), 2.42 (s, 3H), 2.15 – 1.99 (m, 4H), 1.60 – 1.46 (m, 6H). **^{13}C NMR (101 MHz, $CDCl_3$)** δ = 200.2 (Cq), 143.2 (Cq), 137.8 (Cq), 136.3 (Cq), 133.9 (CH), 129.7 (CH), 128.6 (CH), 127.9 (CH), 127.2 (CH), 126.4 (CH), 123.9 (CH), 103.9 (Cq), 84.0 (CH), 48.3 (CH_2), 46.8 (CH_2), 31.3 (CH_2), 27.2 (CH_2), 25.9 (CH_2), 21.6 (CH_3). **(ESI)-HRMS** calcd for $C_{25}H_{30}NO_2S$ $[M+H]^+$ 408.1992, found 408.2001.

N-cinnamyl-N-(hexa-2,3-dien-1-yl)-4-methylbenzenesulfonamide (1i)

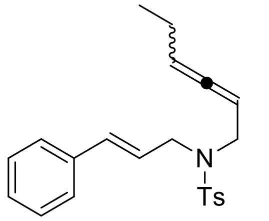

The general procedure (**GP-1b**) was followed using propionaldehyde. Purification by chromatography on silica gel yielded **1i** (36.7mg, 5%) as a viscous oil. **^{1}H NMR (400 MHz, acetone)** δ = 7.80 (d, *J* = 8.3 Hz, 2H), 7.44 (dd, *J* = 8.6, 0.8 Hz, 2H), 7.45 – 7.22 (m, 5H), 6.56 (d, *J* = 15.9 Hz, 1H), 6.08 (dt, *J* = 15.9, 6.6 Hz, 1H), 5.31 – 5.21 (m, 1H), 5.05 – 4.94 (m, 1H), 4.05 (d, *J* = 6.9 Hz, 2H), 3.89 (dd, *J* = 6.9, 2.3 Hz, 2H), 2.45 (s, 3H), 2.04 – 1.92 (m, 2H), 0.96 (t, *J* = 7.4 Hz, 3H). **^{13}C NMR (101 MHz, acetone)** δ = 205.6 (Cq), 144.2 (Cq), 138.9 (Cq), 137.4 (Cq), 134.5 (CH), 130.6 (CH), 129.4 (CH), 128.6 (CH), 128.1 (CH), 127.3 (CH), 124.9 (CH), 94.8 (CH), 87.9 (CH_2), 49.5 (CH_2), 47.3 (CH_2), 22.3 (CH_2), 21.4 (CH_3), 13.8 (CH_3). **(ESI)-HRMS** calcd for $C_{22}H_{25}NNaO_2S$ $[M+Na]^+$ 390.1498, found 390.1503.

N-cinnamyl-4-methyl-N-(4-phenylbuta-2,3-dien-1-yl)benzenesulfonamide (1l)

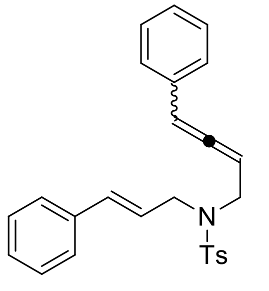

The general procedure (**GP-1b**) was followed using benzaldehyde. Purification by chromatography on silica gel yielded **1l** (91.0 mg, 22%) as a white solid. **^{1}H NMR (400 MHz, $CDCl_3$)** δ = 7.78 (d, *J* = 8.3 Hz, 2H), 7.41 – 7.07 (m, 12H), 6.39 (d, *J* = 15.9 Hz, 1H), 6.15 (dt, *J* = 6.4, 2.3 Hz, 1H), 5.99 (dt, *J* = 15.8, 6.8 Hz, 1H), 5.42 (q, *J* = 6.7 Hz, 1H), 4.20 – 3.92 (m, 4H), 2.45 (s, 3H). **^{13}C NMR (101 MHz, $CDCl_3$)** δ = 206.4 (Cq), 143.4 (Cq), 137.5 (Cq), 136.1 (Cq), 134.3 (CH), 133.5 (Cq), 129.8 (CH), 128.7 (CH), 128.5 (CH), 127.9 (CH), 127.4 (CH), 127.3 (CH), 126.9

(CH), 126.4 (CH), 123.4 (CH), 96.0 (CH), 90.6 (CH), 49.0 (CH_2), 45.8 (CH_2), 21.5 (CH_3). **(ESI)-HRMS** calcd for $C_{26}H_{25}KNO_2S$ $[M+K]^+$ 454.1238, found 453.1223.

N-cinnamyl-4-methyl-N-(propa-1,2-dien-1-yl)benzenesulfonamide (3a)

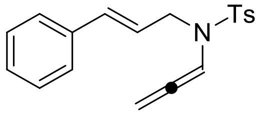

The general procedure (**GP-2**) was followed using the correspondent enyne (828 mg, 2.54 mmol). Purification by chromatography on silica gel yielded **3a** (560 mg, 67%) as a white solid. **^{1}H NMR (400 MHz, $CDCl_3$)** δ = 7.72 (d, *J* = 8.2 Hz, 2H), 7.38 – 7.20 (m, 7H), 6.89 (t, *J* = 6.2 Hz, 1H), 6.45 (d, *J* = 15.9 Hz, 1H), 5.98 (dt, *J* = 15.9, 6.2 Hz, 1H), 5.31 (d, *J* = 6.2 Hz, 2H), 3.98 (dd, *J* = 6.2, 1.4 Hz, 2H), 2.40 (s, 3H).**^{13}C NMR (101 MHz, $CDCl_3$)** δ = 201.9 (Cq), 143.8 (Cq), 136.5 (Cq), 135.7 (Cq), 133.6 (CH), 129.8 (CH), 128.5 (CH), 127.8 (CH), 127.3 (CH), 126.4 (CH), 123.5 (CH), 100.1 (CH), 88.0 (CH_2), 48.6 (CH_2), 21.6 (CH_3). **(ESI)-HRMS** calcd for $C_{19}H_{19}NNaO_2S$ $[M+Na]^+$ 348.1029, found 347.1017.

(E)-4-methyl-N-(propa-1,2-dien-1-yl)-N-(3-(o-tolyl)allyl)benzenesulfonamide (3b)

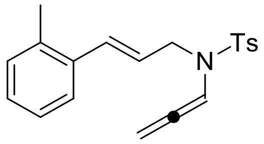

The general procedure (**GP-2**) was followed using the correspondent enyne (140.0 mg, 0.47 mmol). Purification by chromatography on silica gel yielded **3b** (78.7 mg, 56 %) as a viscous clear oil. **^{1}H NMR (400 MHz, acetone)** δ = 7.80 (d, *J* = 8.2 Hz, 2H), 7.45 (d, *J* = 8.1 Hz, 2H), 7.35 – 7.27 (m, 1H), 7.18 – 7.09 (m, 3H), 6.92 (t, *J* = 6.3 Hz, 1H), 6.78 (d, *J* = 15.8 Hz, 1H), 5.92 (dt, *J* = 15.8, 6.1 Hz, 1H), 5.41 (d, *J* = 6.3 Hz, 2H), 4.05 (dd, *J* = 6.1, 1.5 Hz, 2H), 2.44 (s, 3H), 2.30 (s, 3H). **^{13}C NMR (101 MHz, acetone)** δ = 201.9 (Cq), 144.0 (Cq), 136.1 (Cq), 135.6 (Cq), 135.2 (Cq), 131.1 (CH), 130.1 (CH), 129.8 (CH), 127.5 (CH), 127.3 (CH), 126.02 (CH), 125.6 (CH), 125.0 (CH), 99.8 (CH), 87.3 (CH_2), 48.6 (CH_2), 20.5 (CH_3), 18.8 (CH_3). **(ESI)-HRMS** calcd for $C_{20}H_{22}NO_2S$ $[M+H]^+$ 340.1366, found 340.1378.

(E)-N-(3-(4-methoxyphenyl)allyl)-4-methyl-N-(propa-1,2-dien-1-yl)benzenesulfonamide (3c)

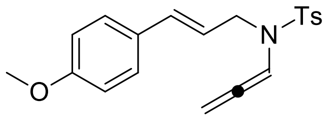

The general procedure (**GP-2**) was followed using the correspondent enyne (78.6 mg, 0.22 mmol). Purification by chromatography on silica gel yielded **3c** (38.6 mg, 49%) as a viscous oil. **^{1}H NMR (400 MHz, Acetone)** δ = 7.78 (d, *J* = 8.3 Hz, 2H), 7.49 – 7.40 (m, 2H), 7.29 (d, *J* = 8.7 Hz, 2H), 6.95 – 6.83 (m, 3H), 6.47 (d, *J* = 15.9 Hz, 1H), 5.91 (dt, *J* = 15.9, 6.3 Hz, 1H), 5.38 (d, *J* = 6.3 Hz, 2H), 3.98 (d, *J* = 6.3 Hz, 2H), 3.80 (s, 3H), 2.43 (s, 3H). **^{13}C NMR (101 MHz, $CDCl_3$)** δ = 207.1 (Cq), 164.8 (Cq), 149.2 (Cq), 141.2 (Cq), 138.3 (CH), 135.0 (CH), 134.4 (Cq), 132.8 (CH), 132.5 (CH), 126.2 (CH), 119.1 (CH), 104.9 (CH), 92.6 (CH_2), 59.9 (CH_2), 53.7 (CH_3), 25.8 (CH_3). **(ESI)-HRMS** calcd for $C_{20}H_{21}KNO_3S$ $[M+K]^+$ 394.0874 found 394.0881.

(E)-N-(3-(4-chlorophenyl)allyl)-4-methyl-N-(propa-1,2-dien-1-yl)benzenesulfonamide (3d)

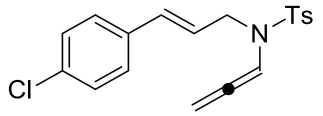

The general procedure (**GP-2**) was followed using the correspondent enyne (300.0 mg, 0.83 mmol). Purification by chromatography on silica gel yielded **3d** (111.2 mg, 37%) as a white solid. **^{1}H NMR (300 MHz, CDCl$_3$)** δ = 7.71 (d, *J* = 8.3 Hz, 2H), 7.45 – 7.02 (m, 6H), 6.88 (t, *J* = 6.2 Hz, 1H), 6.40 (d, *J* = 16.0 Hz, 1H), 5.95 (dt, *J* = 15.9, 6.1 Hz, 1H), 5.30 (d, *J* = 6.2 Hz, 2H), 3.95 (d, *J* = 6.5 Hz, 2H), 2.41 (s, 3H). **^{13}C NMR (75 MHz, CDCl$_3$)** δ = 201.8 (Cq), 143.8 (Cq), 135.7 (Cq), 135.0 (Cq), 133.4 (Cq), 132.1 (CH), 129.7 (CH), 128.6 (CH), 127.5 (CH), 127.3 (CH), 124.2 (CH), 100.1 (CH), 87.9 (CH$_2$), 48.4 (CH$_2$), 21.5 (CH$_3$). **(ESI)-HRMS** calcd for $C_{19}H_{19}ClNO_2S$ [M+H]$^+$ 360.0820, found 360.0811.

(E)-N-(3-(3-cyanophenyl)allyl)-4-methyl-N-(propa-1,2-dien-1-yl)benzenesulfonamide (3e)

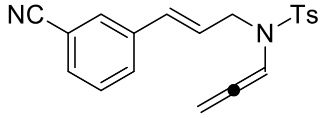

The general procedure (**GP-2**) was followed using the correspondent enyne (200.0 mg, 0.57 mmol). Purification by chromatography on silica gel yielded **3e** (54.3 mg, 27%) as a viscous yellow oil. **^{1}H NMR (400 MHz, acetone)** δ = 7.79 (d, *J* = 8.0 Hz, 2H), 7.73 – 7.61 (m, 3H), 7.54 (t, *J* = 7.7 Hz, 1H), 7.43 (d, *J* = 8.0 Hz, 2H), 6.93 (t, *J* = 6.3 Hz, 1H), 6.58 (d, *J* = 16.0 Hz, 1H), 6.23 (dt, *J* = 16.1, 6.0 Hz, 1H), 5.40 (d, *J* = 6.2 Hz, 2H), 4.06 (d, *J* = 5.7 Hz, 2H), 2.42 (s, 3H). **^{13}C NMR (101 MHz, acetone)** δ = 201.8 (Cq), 144.1 (Cq), 138.0 (Cq), 136.0 (Cq), 131.0 (CH), 130.9 (CH), 130.6 (CH), 129.8 (CH), 129.7 (CH), 129.7 (CH), 127.3 (CH), 126.7 (CH), 118.3 (Cq), 112.7 (Cq), 99.8 (CH), 87.6 (CH$_2$), 48.1 (CH$_2$), 20.6 (CH$_3$). **(ESI)-HRMS** calcd for $C_{20}H_{18}N_2NaO_2S$ [M+Na]$^+$ 373.0981, found 373.0992.

N-cinnamyl-N-(propa-1,2-dien-1-yl)methanesulfonamide (3f)

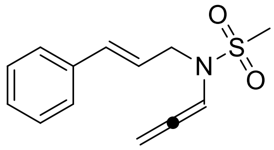

The general procedure (**GP-2**) was followed using the correspondent enyne (250 mg, 1.00 mmol). Purification by chromatography on silica gel yielded **3f** (124.5 mg, 50%) as a white solid. **^{1}H NMR (400 MHz, CDCl$_3$)** δ = 7.40 – 7.22 (m, 5H), 6.73 (tt, *J* = 6.2, 0.7 Hz, 1H), 6.63 (d, *J* = 15.8 Hz, 1H), 6.18 (dt, *J* = 15.9, 6.5 Hz, 1H), 5.44 (d, *J* = 6.3 Hz, 2H), 4.15 (ddd, *J* = 6.4, 1.4, 0.7 Hz, 2H), 2.93 (s, 3H). **^{13}C NMR (101 MHz, CDCl$_3$)** δ = 201.3 (Cq), 136.2 (Cq), 134.3 (CH), 128.7 (CH), 128.1 (CH), 126.5 (CH), 123.0 (CH), 99.7 (CH), 88.2 (CH$_2$), 48.1 (CH$_2$), 39.1 (CH$_3$). **(ESI)-HRMS** calcd for $C_{13}H_{16}NO_2S$ [M+H]$^+$ 250.0896, found 250.0910.

(E)-N-(propa-1,2-dien-1-yl)-N-(3-(o-tolyl)allyl)methanesulfonamide (3g)

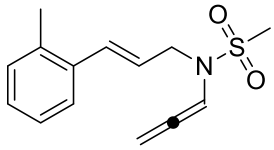

The general procedure (**GP-2**) was followed using the correspondent enyne (175 mg, 0.67 mmol). Purification by chromatography on silica gel yielded **3g** (43.7 mg, 25.0%) as a pale yellow solid. **^{1}H NMR (400 MHz, acetone)** δ 7.51 – 7.43 (m, 1H), 7.20 – 7.11 (m, 3H), 6.88 (dd, *J* = 15.8, 1.6 Hz, 1H), 6.76 (t, *J* = 6.3 Hz, 1H), 6.13 (dt, *J* = 15.7, 6.2 Hz, 1H), 5.49 (d, *J* = 6.3 Hz, 2H), 4.18 (d, *J* = 6.2 Hz, 2H), 3.04 (s, 3H), 2.33 (s, 3H). **^{13}C NMR (101 MHz, acetone)** δ = 201.4 (Cq), 135.6 (Cq), 135.3 (Cq), 131.4 (CH), 130.2 (CH), 127.6 (CH), 126.1 (CH), 125.6 (CH), 125.3 (CH), 99.6 (CH), 87.4 (CH_2), 48.5 (CH_2), 38.0 (CH_3), 18.9 (CH_3). **(ESI)-HRMS** calcd for $C_{14}H_{17}NNaO_2S$ $[M+Na]^+$ 286.0872, found 286.0864.

N-cinnamyl-N-(propa-1,2-dien-1-yl)benzenesulfonamide (3h)

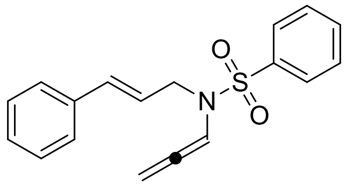

The general procedure (**GP-2**) was followed using the correspondent enyne (370 mg, 1.19 mmol). Purification by chromatography on silica gel yielded **3h** (252.2 mg, 68%) as a pale white solid. **^{1}H NMR (400 MHz, acetone)** δ = 7.93 (d, *J* = 7.6 Hz, 2H), 7.82 – 7.56 (m, 3H), 7.40 – 7.22 (m, 5H), 6.91 (t, *J* = 6.3 Hz, 1H), 6.55 (d, *J* = 15.9 Hz, 1H), 6.09 (dt, *J* = 15.9, 6.1 Hz, 1H), 5.39 (d, *J* = 6.2 Hz, 2H), 4.04 (d, *J* = 6.1 Hz, 2H). **^{13}C NMR (101 MHz, acetone)** δ = 201.9 (Cq), 138.8 (Cq), 136.6 (Cq), 133.4 (CH), 133.1 (CH), 129.3 (CH), 128.5 (CH), 127.7 (CH), 127.2 (CH), 126.3 (CH), 123.6 (CH), 99.7 (CH), 87.4 (CH_2), 48.5 (CH_2). **(ESI)-HRMS** calcd for $C_{18}H_{18}NO_2S$ $[M+H]^+$ 312.1053, found 312.1040.

N-cinnamyl-2,4,6-trimethyl-N-(propa-1,2-dien-1-yl)benzenesulfonamide (3j)

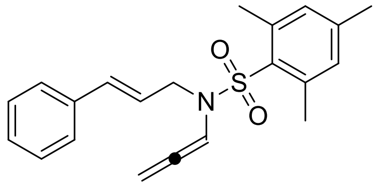

The general procedure (**GP-2**) was followed using the correspondent enyne (270 mg, 0.76 mmol). Purification by chromatography on silica gel yielded **3j** (108.2 mg, 40%) as a viscous liquid. **^{1}H NMR (300 MHz, $CDCl_3$)** δ = 7.34 – 7.16 (m, 5H), 6.94 (s, 2H), 6.89 (t, *J* = 6.3 Hz, 1H), 6.30 (d, *J* = 15.9 Hz, 1H), 5.94 (dt, *J* = 15.9, 6.4 Hz, 1H), 5.36 (d, *J* = 6.3 Hz, 2H), 3.96 (d, *J* = 6.3 Hz, 2H), 2.64 (s, 6H), 2.26 (s, 3H).**^{13}C NMR (75 MHz, acetone)** δ = 200.7 (Cq), 143.2 (Cq), 139.9 (CH), 136.6 (Cq), 133.4 (CH), 133.2 (Cq), 132.1 (CH), 128.5 (CH), 127.6 (CH), 126.2 (CH), 123.2 (CH), 99.5 (CH), 87.7 (CH_2), 47.4 (CH_2), 22.3 (CH_3), 20.0 (CH_3). **(ESI)-HRMS** calcd for $C_{21}H_{23}NKO_2S$ $[M+H]^+$ 392.1081, found 392.1094.

N-cinnamyl-4-nitro-N-(propa-1,2-dien-1-yl)benzenesulfonamide (3k)

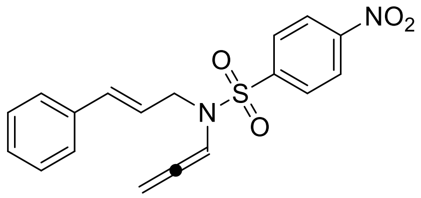

The general procedure (**GP-2**) was followed using the correspondent enyne (357.0 mg, 1.00 mmol). Purification by chromatography on silica gel yielded **3k** (89.6 mg. 25%) as a viscous yellow oil. **^{1}H NMR (300 MHz, acetone) δ =** 8.33 (d, *J* = 8.8 Hz, 2H), 8.02 (d, *J* = 8.8 Hz, 2H), 7.40 – 7.22 (m, 5H), 6.88 (t, *J* = 6.2 Hz, 1H), 6.49 (d, *J* = 15.9 Hz, 1H), 5.95 (dt, *J* = 15.8, 6.3 Hz, 1H), 5.40 (d, *J* = 6.2 Hz, 2H), 4.07 (d, *J* = 6.4 Hz, 2H). **^{13}C NMR (75 MHz, $CDCl_3$)** δ = 201.8 (Cq), 150.1 (Cq), 144.4 (Cq), 135.9 (Cq), 134.5 (CH), 128.7 (CH), 128.5 (CH), 128.2 (CH), 126.3 (CH), 124.3 (CH), 122.3 (CH), 99.4 (CH) ,88.5 (CH_2), 49.1 (CH_2). **(ESI)-HRMS** calcd for $C_{18}H_{17}N_2O_4S$ $[M+H]^+$ 357.0904, found 357.0894.

4-chloro-N-cinnamyl-N-(propa-1,2-dien-1-yl)benzenesulfonamide (3i)

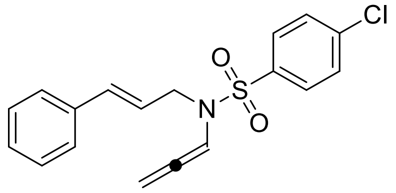

The general procedure (**GP-2**) was followed using the correspondent enyne (250 mg, 0.72 mmol). Purification by chromatography on silica gel yielded **3i** (168 mg, 67%) as a white solid. **^{1}H NMR (300 MHz, acetone)** δ = 7.91 (d, *J* = 8.6 Hz, 2H), 7.65 (d, *J* = 8.6 Hz, 2H), 7.48 – 7.18 (m, 5H), 6.89 (t, *J* = 6.3 Hz, 1H), 6.54 (d, *J* = 16.0 Hz, 1H), 6.07 (dt, *J* = 15.9, 6.2 Hz, 1H), 5.41 (d, *J* = 6.3 Hz, 2H), 4.04 (d, *J* = 6.2 Hz, 2H).**^{13}C NMR (75 MHz, acetone)** δ = 202.0 (Cq), 138.9 (Cq), 137.5 (Cq), 136.5 (Cq), 133.6 (CH), 129.5 (CH), 129.1 (CH), 128.5 (CH), 127.8 (CH), 126.3 (CH), 123.2 (CH), 99.4 (CH_2), 87.6 (CH), 48.6 (CH_2). **(ESI)-HRMS** calcd for $C_{18}H_{17}ClNO_2S$ $[M+H]^+$ 346.0663, found 346.0670.

N-cinnamyl-N-(propa-1,2-dien-1-yl)naphthalene-1-sulfonamide (3l)

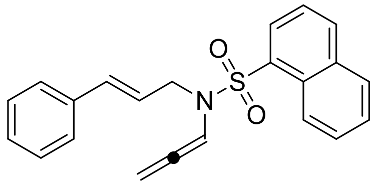

The general procedure (**GP-2**) was followed using the correspondent enyne (135 mg, 0.37 mmol). Purification by chromatography on silica gel yielded **3l** (106 mg, 78%) as a viscous liquid. **^{1}H NMR (300 MHz, acetone) δ =** 8.57 – 8.51 (m, 1H), 8.17 – 8.08 (m, 2H), 8.07 – 8.00 (m, 1H), 7.90 (dd, *J* = 8.7, 2.0 Hz, 1H), 7.77 – 7.72 (m, 2H), 7.33 – 7.14 (m, 5H), 6.98 (t, *J* = 6.3 Hz, 1H), 6.54 (dt, *J* = 15.8, 1.5 Hz, 1H), 6.07 (dt, *J* = 15.9, 6.2 Hz, 1H), 5.36 (d, *J* = 6.3 Hz, 2H), 4.08 (d, *J* = 6.2 Hz, 2H). **^{13}C NMR (75 MHz, acetone)** δ = 201.9 (Cq), 136.6 (Cq), 135.9 (Cq), 135.0 (Cq), 133.4 (CH), 132.3 (Cq), 129.5 (CH), 129.4 (CH), 129.0 (CH), 128.7 (CH), 128.5 (CH), 127.9 (CH), 127.7 (CH), 127.6 (CH), 126.3 (CH), 123.5 (CH), 122.5 (CH), 99.7 (CH_2), 87.5 (CH), 48.5 (CH_2). **(ESI)-HRMS** calcd for $C_{22}H_{20}NO_2S$ $[M+H]^+$ 362.1209, found 362.1206.

(E)-4-methyl-N-(4-phenylbut-3-en-1-yl)-N-(propa-1,2-dien-1-yl)benzenesulfonamide (3m)

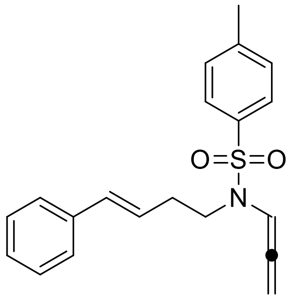

The general procedure (**GP-2**) was followed using the correspondent enyne (140 mg, 0.41 mmol). Purification by chromatography on silica gel yielded **3m** (55.8 mg, 40%) as a viscous liquid. **^{1}H NMR (400 MHz, acetone)** δ = 7.76 (d, *J* = 8.4 Hz, 2H), 7.47 – 7.41 (m, 2H), 7.40 – 7.34 (m, 2H), 7.34 – 7.26 (m, 2H), 7.25 – 7.16 (m, 1H), 6.87 (t, *J* = 6.3 Hz, 1H), 6.46 (d, *J* = 15.9 Hz, 1H), 6.22 (dt, *J* = 15.9, 7.1 Hz, 1H), 5.42 (d, *J* = 6.3 Hz, 2H), 3.29 (dd, *J* = 8.2, 6.3 Hz, 2H), 2.57 – 2.26 (m, 5H). **^{13}C NMR (101 MHz, acetone)** δ = 200.6 (Cq), 144.0 (Cq), 137.5 (Cq), 135.7 (Cq), 131.9 (CH), 129.9 (CH), 128.5 (CH), 127.2 (CH), 127.1 (CH), 126.3 (CH), 126.0 (CH), 99.8 (CH), 87.3 (CH_2), 46.1 (CH_2), 31.6 (CH_2), 20.6 (CH_3). **(ESI)-HRMS** calcd for $C_{20}H_{21}NNaO_2S$ $[M+Na]^+$ 362.1185, found 361.1192.

Characterization of products

General procedure 3 (GP-3)

To a vial charged with substrate **1** or **3** (1 equiv.) and complex **A** (1 mol%), dry and degassed DMF (0.05 M) was added through a syringe. The solution was transferred in two NMR tubes capped with a rubber septum and it was then degassed through three freeze-pump cycles. The homogeneous solution was placed in an oil bath kept at 25 °C and irradiated with LED stripes for 6 hours (for 1,7-enallenes) or 24 hours (for 1,6-enallenes). Conversion was monitored by TLC and the mixture was then concentrated in vacuo. The residue was eventually purified by chromatography on silica gel.

(1R,5R,7R)-6-methylene-7-phenyl-3-tosyl-3-azabicyclo[3.2.0]heptane (2a)

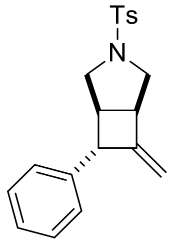

The general procedure (**GP-3**) was followed using **1a** (35mg, 0.1 mmol). Purification by chromatography on silica gel (*n*-hexanes/EtOAc 90:10) yielded **2a** (28.9 mg, 82%) as a white solid. **^{1}H NMR (400 MHz, CDCl$_3$)** δ = 7.72 (d, *J* = 8.1 Hz, 2H), 7.39 – 7.27 (m, 4H), 7.26 – 7.17 (m, 3H), 5.05 (t, *J* = 2.4 Hz, 1H), 4.88 (t, *J* = 2.5 Hz, 1H), 3.95 (br, 1H), 3.69 (dd, *J* = 9.7, 4.5 Hz, 2H), 3.42 (br, 1H), 2.89 – 2.65 (m, 3H), 2.44 (s, 3H). **^{13}C NMR (101 MHz, CDCl$_3$)** δ = 151.5 (Cq), 143.7 (Cq), 142.3 (Cq), 131.9 (Cq), 129.6 (CH), 128.6 (CH), 128.1 (CH), 127.2 (CH), 126.6 (CH), 110.1 (CH$_2$), 53.8 (2CH$_2$), 53.0 (CH), 45.0 (CH), 43.9 (CH), 21.6 (CH$_3$). **(ESI)-HRMS** calcd for $C_{20}H_{21}NNaO_2S$ [M+Na]$^+$ 362.1185, found 362.1179.

(1R,5R,6R)-6-(4-chlorophenyl)-7-methylene-3-tosyl-3-azabicyclo[3.2.0]heptane (2b)

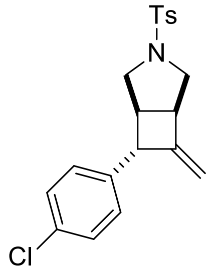

The general procedure (**GP-3**) was followed using **1b** (75 mg, 0.2 mmol). Purification by chromatography on silica gel (*n*-hexanes/EtOAc 90:10) yielded **2b** (40.1mg, 53%) as a white solid. **^{1}H NMR (300 MHz, CDCl$_3$)** δ = 7.72 (d, *J* = 8.0 Hz, 2H), 7.39 – 7.23 (m, 4H), 7.16 (d, *J* = 8.2 Hz, 2H), 5.06 (s, 1H), 4.87 (s, 1H), 3.93 (br, 1H), 3.68 (dd, *J* = 9.7, 4.6 Hz, 2H), 3.43 (br, 1H), 2.85 – 2.62 (m, 3H), 2.45 (s, 3H). **^{13}C NMR (75 MHz, CDCl$_3$)** δ = 151.1 (Cq), 143.79 (Cq), 140.8 (Cq), 132.3 (Cq), 131.8 (Cq), 129.6 (CH), 128.7 (CH), 128.6 (CH), 128.1 (CH), 110.3 (CH$_2$), 53.7 (CH$_2$), 53.7 (CH$_2$), 52.3 (CH), 45.0 (CH), 43.8 (CH), 21.6 (CH$_3$). **(ESI)-HRMS** calcd for $C_{20}H_{20}ClNNaO_2S$ [M+Na]$^+$ 396.0795, found 396.0805.

(1R,5R,7R)-6-methylene-3-tosyl-7-(4-(trifluoromethyl)phenyl)-3-azabicyclo[3.2.0]heptane (2c)

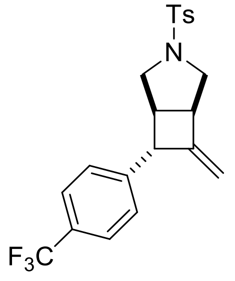

The general procedure (**GP-3**) was followed using **1c** (80.9 mg, 0.2 mmol). Purification by chromatography on silica gel (*n*-hexanes/EtOAc 90:10) yielded **2c** (54.5 mg, 67%) as a white solid. **^{1}H NMR (400 MHz, CDCl$_3$)** δ = 7.75 (d, *J* = 8.0 Hz, 2H), 7.58 (d, *J* = 8.0 Hz, 2H), 7.37 (dd, *J* = 8.2, 4.0 Hz, 4H), 5.11 (s, 1H), 4.90 (s, 1H), 4.04 (br, 1H) 3.72 (dd, *J* = 9.8, 3.7 Hz, 2H), 3.47 (br, 1H), 2.88 – 2.67 (m, 3H), 2.46 (s, 3H). **^{13}C NMR (101 MHz, CDCl$_3$)** δ = 150.6 (Cq), 146.3 (q, *J* = 1.5 Hz, Cq), 143.9 (Cq), 131.6 (Cq), 129.7 (CH), 128.8 (q, *J* = 32.2 Hz, Cq), 128.1 (CH), 127.6 (CH), 125.6 (q, *J* = 3.6 Hz, CH), 124.2 (q, *J* = 271.7 Hz, Cq) 110.8 (CH$_2$), 53.75 (CH$_2$), 53.72 (CH$_2$), 53.6 (CH), 45.0 (CH), 43.7 (CH), 21.6 (CH$_3$). **^{19}F NMR (564 MHz, CDCl$_3$) δ = -62.28. (ESI)-HRMS** calcd for $C_{21}H_{20}F_3NNaO_2S$ [M+Na]$^+$ 430.1059, found 429.1070.

(1R,5R,6R)-6-(3-fluorophenyl)-7-methylene-3-tosyl-3-azabicyclo[3.2.0]heptane (2d)

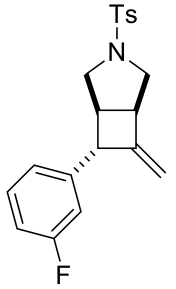

The general procedure (**GP-3**) was followed using **1d** (71.0 mg, 02 mmol). Purification by chromatography on silica gel (*n*-hexanes/EtOAc 90:10) yielded **2d** (39.0 mg, 55%) as a white solid. **^{1}H NMR (600 MHz, CDCl$_3$)** δ = 7.71 (d, *J* = 8.3 Hz, 2H), 7.33 (d, *J* = 8.0 Hz, 2H), 7.28 – 7.21 (m, 1H), 6.97 (d, *J* = 7.9 Hz, 1H), 6.95 – 6.85 (m, 2H), 5.07 – 5.03 (m, 1H), 4.90 – 4.86 (m, 1H), 3.95 – 3.90 (m, 1H), 3.70 – 3.60 (m, 2H), 3.41 (br, 1H), 2.82 – 2.72 (m, 2H), 2.69 (dd, *J* = 10.0, 5.9 Hz, 1H), 2.43 (s, 3H). **^{13}C NMR (151 MHz, CDCl$_3$)** δ = 163.1 (d, *J* = 246.0 Hz, Cq), 150.8 (Cq), 144.9 (d, *J* = 6.7 Hz, Cq), 143.9 (Cq), 131.99 (Cq), 130.14 (d, *J* = 8.2 Hz, CH), 129.7 (CH), 128.2 (CH), 123.0 (d, *J* = 2.8 Hz, CH), 114.0 (d, *J* = 21.4 Hz, CH), 113.5 (d, *J* = 21.0 Hz, CH), 110.6 (CH$_2$), 53.80 (CH$_2$), 53.76 (CH$_2$), 52.6 (CH), 45.0 (CH), 43.8 (CH), 21.6 (CH$_3$). **^{19}F NMR (564 MHz, CDCl$_3$)** δ = -112.87 (q, *J* = 8.7 Hz). **(ESI)-HRMS** calcd for $C_{20}H_{20}FNNaO_2S$ [M+Na]$^+$ 380.1091, found 380.1079.

3-((1R,5R,6R)-7-methylene-3-tosyl-3-azabicyclo[3.2.0]heptan-6-yl)benzonitrile (2e)

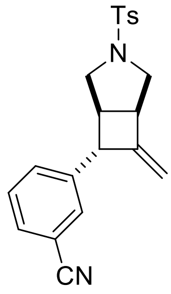

The general procedure (**GP-3**) was followed using **1e** (72.6 mg, 0.20 mmol). Purification by chromatography on silica gel (*n*-hexanes/EtOAc 65:35) yielded **2e** (49.0 mg, 67%) as a white solid. **^{1}H NMR (400 MHz, CDCl$_3$)** δ = 7.73 (d, *J* = 8.3 Hz, 2H), 7.60 – 7.50 (m, 2H), 7.51 – 7.29 (m, 4H), 5.13 (br, 1H), 4.90 (br, 1H), 4.04 – 3.96 (m, 1H), 3.71 (dd, *J* = 9.8, 4.2 Hz, 2H), 3.51 – 3.42 (m, 1H), 2.86 – 2.63 (m, 3H), 2.46 (s, 3H). **^{13}C NMR (101 MHz, CDCl$_3$)** δ = 150.1 (Cq), 143.9 (Cq), 143.7 (Cq), 132.0 (CH), 131.6 (Cq), 130.7 (CH), 130.3 (CH), 129.7 (CH), 129.5 (CH), 128.1 (CH), 118.9 (Cq), 112.7 (Cq), 111.1 (CH$_2$), 53.7 (CH$_2$), 53.6 (CH$_2$), 52.2 (CH), 45.0 (CH), 43.7 (CH), 21.6 (CH$_3$). **(ESI)-HRMS** calcd for $C_{21}H_{20}N_2NaO_2S$ [M+Na]$^+$ 387.1138, found 387.1146.

(1R,5R,7R)-6-methylene-7-(o-tolyl)-3-tosyl-3-azabicyclo[3.2.0]heptane (2f)

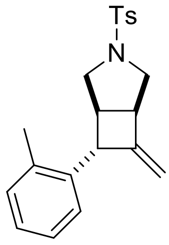

The general procedure (**GP-3**) was followed using **1f** (66.3 mg, 0.19 mmol). Purification by chromatography on silica gel (*n*-hexanes/EtOAc 90:10) yielded **2f** (41.5 mg, 63%) as a white solid. **^{1}H NMR (400 MHz, CDCl$_3$)** δ = 7.73 (d, *J* = 8.3 Hz, 2H), 7.45 – 7.29 (m, 3H), 7.23 – 7.07 (m, 3H), 5.16 (br, 1H), 4.95 (br, 1H), 4.16 – 4.07 (m, 1H), 3.73 (t, *J* = 10.0 Hz, 2H), 3.43 (br, 1H), 2.81 – 2.71 (m, 2H), 2.71 – 2.63 (m, 1H), 2.44 (s, 3H), 2.27 (s, 3H). **^{13}C NMR (101 MHz, CDCl$_3$)** δ = 150.5 (Cq), 143.7 (Cq), 140.2 (Cq), 135.9 (Cq), 131.8 (Cq), 130.4 (CH), 129.6 (CH), 128.1 (CH), 126.5 (CH), 126.1 (CH), 126.0 (CH), 111.0 (CH$_2$), 53.9 (2CH$_2$), 50.0 (CH), 44.6 (CH), 43.5 (CH), 21.6 (CH$_3$), 20.0 (CH$_3$). **(ESI)-HRMS** calcd for $C_{21}H_{23}NNaO_2S$ [M+Na]$^+$ 376.1342, found 376.1351.

(1R,5R,6R)-6-(4-methoxyphenyl)-7-methylene-3-tosyl-3-azabicyclo[3.2.0]heptane (2g)

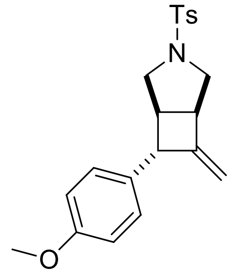

The general procedure (**GP-3**) was followed using **1g** (73.9 mg, 0.20 mmol). Purification by chromatography on silica gel (*n*-hexanes/EtOAc 90:10) yielded **2g** (58.8 mg, 80%, 6:1 mixture of diastereoisomers that are not separable by chromatography) as a white solid. Mayor diastereoisomer: **^{1}H NMR (400 MHz, $CDCl_3$)** δ = 7.72 (d, *J* = 8.3 Hz, 2H), 7.43 – 7.31 (m, 2H), 7.18 – 7.11 (m, 2H), 6.90 – 6.77 (m, 2H), 5.05 – 4.99 (m, 1H), 4.88 – 4.82 (m, 1H), 3.89 (dq, *J* = 5.7, 2.9 Hz, 1H), 3.78 (s, 3H), 3.67 (dd, *J* = 9.7, 5.8 Hz, 2H), 3.46 – 3.36 (m, 1H), 2.82 – 2.72 (m, 2H), 2.68 (dd, *J* = 9.8, 5.8 Hz, 1H), 2.44 (s, 3H). **^{13}C NMR (101 MHz, $CDCl_3$)** δ = 158.3 (Cq), 152.1 (Cq), 143.8 (Cq), 134.5 (Cq), 131.7 (Cq), 129.6 (CH), 128.3 (CH), 128.1 (CH), 114.0 (CH), 109.8 (CH_2), 55.3 (CH_3), 53.8 ($2CH_2$), 52.3 (CH), 44.9 (CH), 44.1 (CH), 21.6 (CH_3). **(ESI)-HRMS** calcd for $C_{21}H_{23}KNO_3S$ $[M+K]^+$ 408.1030, found 408.1033.

(1R,5R,7R)-6-methylene-7-(pyridin-3-yl)-3-tosyl-3-azabicyclo[3.2.0]heptane. (2h)

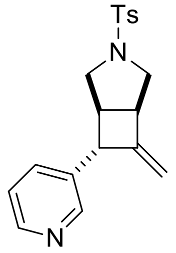

The general procedure (**GP-3**) was followed using **1h** (68.2 mg, 0.20 mmol). Purification by chromatography on silica gel (DCM/MeOH 98:2) yielded **2h** (40.7 mg, 59%) as a blue/green solid. **^{1}H NMR (400 MHz, $CDCl_3$)** δ = 7.71 (d, *J* = 8.3 Hz, 2H), 7.35 (d, *J* = 7.9 Hz, 2H), 5.09 (s, 1H), 4.87 (s, 1H), 4.14 (br, 1H), 3.69 (dd, *J* = 9.8, 6.2 Hz, 2H), 3.52 – 3.41 (m, 1H), 2.97 – 2.67 (m, 3H), 2.44 (s, 3H). **^{13}C NMR (101 MHz, $CDCl_3$)** δ = 150.6 (Cq), 143.9 (Cq), 131.7 (Cq), 129.7 (CH), 128.1 (CH), 110.9 (CH_2), 53.7 ($2CH_2$), 45.2 (CH), 43.9 (CH), 21.6 (CH_3). Resonances of the 3-pyridyl unit were barely observed even with prolonged relaxation times. **(ESI)-HRMS** calcd for $C_{19}H_{21}N_2O_2S$ $[M+H]^+$ 341.1324, found 341.1322.

(1R,5R,7R)-6-methylene-7-phenyl-3-oxabicyclo[3.2.0]heptane (2j)

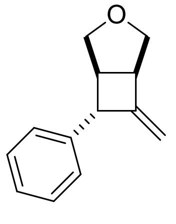

The general procedure (**GP-3**) was followed using **1j** (55.0 mg, 0.29 mmol). Purification by chromatography on silica gel (*n*-hexanes/EtOAc 95:5) yielded **2j** (27.0 mg, 49%, 9:1 mixture of

diastereoisomers that are not separable by chromatography) as a yellow oil. Major diastereoisomer: **^{1}H NMR (400 MHz, CDCl$_3$)** δ = 7.40 – 7.20 (m, 5H), 5.04 (dd, *J* = 2.9, 1.9 Hz, 1H), 4.86 (t, *J* = 2.4 Hz, 1H), 4.14 (dd, *J* = 9.1, 7.5 Hz, 2H), 3.87 – 3.79 (m, 1H), 3.66 (dd, *J* = 8.9, 6.2 Hz, 1H), 3.60 (dd, *J* = 9.4, 4.8 Hz, 2H), 3.01 (dt, *J* = 7.5, 5.0 Hz, 1H). **^{13}C NMR (101 MHz, CDCl$_3$)** δ = 153.1 (Cq), 142.9 (Cq), 128.6 (CH), 127.3 (CH), 126.4 (CH), 108.7 (CH$_2$), 74.0 (CH$_2$), 73.6 (CH$_2$), 52.8 (CH), 46.8 (CH), 45.4 (CH). Minor diastereoisomer: **^{1}H NMR (400 MHz, CDCl$_3$)** δ = 7.43 – 7.20 (m, 5H), 5.13 (dd, *J* = 3.2, 1.8 Hz, 1H), 4.97 (t, *J* = 2.5 Hz, 1H), 4.36 (dt, *J* = 9.3, 3.0 Hz, 2H), 3.72 – 3.51 (m, 2H), 3.41 (dd, *J* = 9.9, 6.1 Hz, 1H), 3.31 – 3.18 (m, 2H). **^{13}C NMR (101 MHz, CDCl$_3$)** δ = 153.5 (Cq), 138.2 (Cq), 128.8 (CH), 128.3 (CH), 126.6 (CH), 108.2 (CH$_2$), 73.7 (CH$_2$), 68.6 (CH$_2$), 49.2 (CH), 47.0 (CH), 42.4 (CH). **(ESI)-HRMS** calcd for $C_{13}H_{14}NaO$ [M+Na]$^+$ 209.0937, found 209.0945.

(1R,5R,7S)-6-cyclohexylidene-7-phenyl-3-tosyl-3-azabicyclo[3.2.0]heptane (2k)

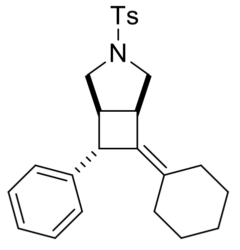

The general procedure (**GP-3**) was followed using **1k** (81.5mg, 0.20 mmol). Purification by chromatography on silica gel (*n*-hexanes/EtOAc 90:10) yielded **2k** (54.2 mg, 66%) as a white solid. **^{1}H NMR (400 MHz, CDCl$_3$)** δ = 7.74 (d, *J* = 8.2 Hz, 2H), 7.51 – 7.09 (m, 7H), 3.88 (br, 1H), 3.70 (d, *J* = 9.8 Hz, 1H), 3.63 (d, *J* = 9.3 Hz, 1H), 3.54 – 3.41 (m, 1H), 2.69 (ddd, *J* = 15.5, 9.5, 6.4 Hz, 2H), 2.62 – 2.53 (m, 1H), 2.46 (s, 3H), 2.13 – 1.96 (m, 2H), 1.81 – 1.17 (m, 8H). **^{13}C NMR (101 MHz, CDCl$_3$)** δ = 144.1 (Cq), 143.6 (Cq), 136.3 (Cq), 131.8 (Cq), 129.6 (CH), 129.2 (Cq), 128.5 (CH), 128.1 (CH), 127.1 (CH), 126.2 (CH), 53.9 (CH$_2$), 53.7 (CH$_2$), 51.5 (CH), 44.0 (CH), 42.4 (CH), 29.3 (CH$_2$), 29.3 (CH$_2$), 27.7 (CH$_2$), 26.8 (CH$_2$), 26.3 (CH$_2$), 21.6 (CH$_3$). **(ESI)-HRMS** calcd for $C_{25}H_{29}NNaO_2S$ [M+Na]$^+$ 430.1811, found 430.1804.

(1R,5R,6S)-6-phenyl-7-propylidene-3-tosyl-3-azabicyclo[3.2.0]heptane (2i)

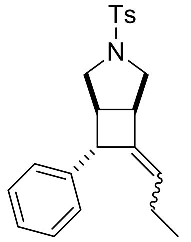

The general procedure (**GP-3**) was followed using **1i** (36mg, 0.10 mmol). Purification by chromatography on silica gel (*n*-hexanes/EtOAc 90:10) yielded **2i** (25.6 mg, 71%, 5:1 mixture of *E*/*Z* isomers that are not separable by chromatography) as a white solid. Mayor isomer: **^{1}H NMR (400 MHz, acetone)** δ = 7.71 (d, *J* = 8.3 Hz, 2H), 7.46 (d, *J* = 8.0 Hz, 2H), 7.33 – 7.25 (m, 4H), 7.24 – 7.13 (m, 1H), 5.38 (tt, *J* = 7.3, 2.3 Hz, 1H), 3.85 (br, 1H), 3.68 – 3.51 (m, 2H), 3.49 (br, 1H), 2.88 – 2.62 (m, 3H), 2.44 (s, 3H), 1.74 – 1.58 (m, 2H), 0.71 (t, *J* = 7.5 Hz, 3H). **^{13}C NMR (101 MHz, acetone)** δ = 143.7 (Cq), 143.6 (Cq), 141.3 (Cq), 132.1 (Cq), 129.6 (CH), 128.5 (CH), 128.1 (CH), 127.1 (CH), 127.0 (CH), 126.2 (CH), 54.4 (CH$_2$), 53.8 (CH$_2$), 51.9 (CH), 44.4 (CH), 44.1

(CH), 21.0 (CH_2), 20.6 (CH_3), 12.9 (CH_3). **(ESI)-HRMS** calcd for $C_{22}H_{25}KNO_2S$ $[M+K]^+$ 406.1238, found 406.1234.

(1R,5R,7S)-6-benzylidene-7-phenyl-3-tosyl-3-azabicyclo[3.2.0]heptane (2l)

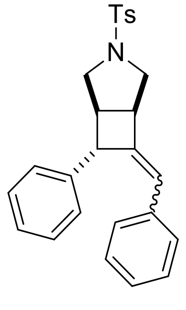

The general procedure (**GP-3**) was followed using **1l** (75mg, 0.18 mmol). Purification by chromatography on silica gel (gradient from *n*-hexanes/EtOAc 96:4 to 90:10) yielded **2l** (29.3 mg, 39%, 1:1 mixture of E/Z isomers, which were separated by chromatography) as a white solid. Isomer A: **^{1}H NMR (400 MHz, CDCl$_3$)** δ = 7.74 (d, *J* = 8.1 Hz, 2H), 7.42 – 7.15 (m, 12H), 6.07 (t, *J* = 2.3 Hz, 1H), 4.12 – 4.05 (m, 1H), 3.92 – 3.80 (m, 2H), 3.73 (d, *J* = 10.0 Hz, 1H), 2.95 (td, *J* = 7.7, 2.1 Hz, 2H), 2.77 (dd, *J* = 9.9, 5.7 Hz, 1H), 2.45 (s, 3H). **^{13}C NMR (101 MHz, CDCl$_3$)** δ = 144.2 (Cq), 143.8 (Cq), 142.5 (Cq), 136.4 (Cq), 131.8 (Cq), 129.7 (CH), 128.7 (CH), 128.6 (CH), 128.2 (CH), 127.7 (CH), 127.5 (CH), 126.8 (2CH), 125.2 (CH), 54.0 (CH_2), 53.7 (CH_2), 52.2 (CH), 45.4 (CH), 45.2 (CH), 21.6 (CH_3). **(ESI)-MS** calcd for $C_{26}H_{25}KNO_2S$ $[M+K]^+$ 454.12 found 454.35. Isomer B: **^{1}H NMR (400 MHz, CDCl$_3$)** δ = 7.72 (d, *J* = 8.3 Hz, 2H), 7.33 – 6.96 (m, 12H), 6.36 (t, *J* = 2.3 Hz, 1H), 4.24 – 4.21 (m, 1H), 3.83 – 3.72 (m, 2H), 3.65 – 3.54 (m, 1H), 2.87 – 2.73 (m, 3H), 2.40 (s, 3H). **^{13}C NMR (101 MHz, CDCl$_3$)** δ = 143.80 (Cq), 142.8 (Cq), 141.5 (Cq), 135.9 (Cq), 132.1 (Cq), 129.7 (CH), 128.9 (CH), 128.3 (CH), 128.2 (CH), 128.2 (CH), 127.1 (CH), 126.8 (CH), 126.7 (CH), 126.3 (CH), 54.6 (CH_2), 54.1 (CH_2), 53.9 (CH), 46.1 (CH), 45.7 (CH), 21.7 (CH_3). **(ESI)-HRMS** calcd for $C_{26}H_{25}KNO_2S$ $[M+K]^+$ 454.1238, found 454.1239.

(1R,7R,8S)-7,8-diphenyl-3-tosyl-3-azabicyclo[4.2.0]oct-5-ene (2'-l)

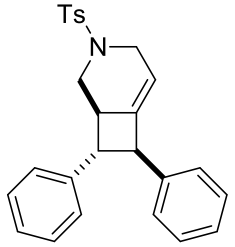

The general procedure (**GP-3**) was followed using **1l** (75mg, 0.18 mmol). Purification by chromatography on silica gel (gradient from *n*-hexanes/EtOAc 96:4 to 90:10) yielded **2'-l** (26.2 mg, 35%) as a white solid. **^{1}H NMR (600 MHz, CDCl$_3$)** δ = 7.71 (d, *J* = 8.3 Hz, 2H), 7.36 – 7.28 (m, 6H), 7.25 – 7.16 (m, 6H), 5.29 (s, 1H), 4.44 – 4.27 (m, 2H), 4.23 – 4.18 (m, 1H), 3.35 – 3.26 (m, 2H), 3.24 (t, *J* = 8.4 Hz, 1H), 2.50 – 2.36 (m, 4H). **^{13}C NMR (151 MHz, CDCl$_3$)** δ = 143.6 (Cq), 141.8 (Cq), 141.0 (Cq), 139.5 (Cq), 134.4 (Cq), 129.8 (CH), 128.7 (CH), 128.6 (CH), 127.5 (CH), 127.1 (CH), 126.9 (CH), 126.6 (CH), 109.8 (CH), 57.5 (CH), 51.0 (CH), 47.7 (CH_2), 45.0 (CH_2), 44.9 (CH), 21.6 (CH_3). **(ESI)-HRMS** calcd for $C_{26}H_{25}KNO_2S$ $[M+K]^+$ 438.1498, found 438.1510.

(1R,5S,6S)-6-phenyl-1-tosyl-3-azabicyclo[3.2.0]hept-2-ene (4a)

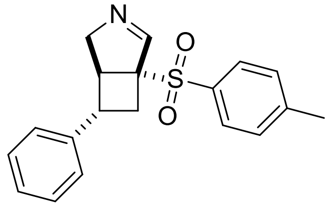

The general procedure (**GP-3**) was followed using **3a** (32.7mg, 0.10mmol). Purification by chromatography on silica gel (*n*-hexanes/EtOAc/DCM 50:40:10) yielded **4a** (19.3mg, 59%) as a pale yellow solid. ^{1}**H NMR (400 MHz, CDCl$_3$)** δ = 7.74 (d, *J* = 7.5 Hz, 2H), 7.60 (s, 1H), 7.43 – 7.20 (m, 7H), 3.97 (d, *J* = 17.0 Hz, 1H), 3.53 (ddd, *J* = 16.9, 6.4, 2.9 Hz, 1H), 3.32 (t, *J* = 6.5 Hz, 1H), 3.20 (dd, *J* = 12.2, 8.9 Hz, 1H), 2.93 (q, *J* = 8.2 Hz, 1H), 2.73 – 2.62 (m, 1H), 2.45 (s, 3H). ^{13}**C NMR (101 MHz, CDCl$_3$)** δ = 161.4 (CH), 145.5 (Cq), 142.2 (Cq), 133.5 (Cq), 130.1 (CH), 128.9 (CH), 128.8 (CH), 127.2 (CH), 127.0 (CH), 76.0 (Cq), 68.0 (CH$_2$), 47.2 (CH), 40.4 (CH), 33.5 (CH$_2$), 21.7 (CH$_3$). **(ESI)-HRMS** calcd for $C_{19}H_{20}NO_2S$ [M+H]$^+$ 326.1209, found 326.1220.

(1R,5S,6S)-6-(o-tolyl)-1-tosyl-3-azabicyclo[3.2.0]hept-2-ene (4b)

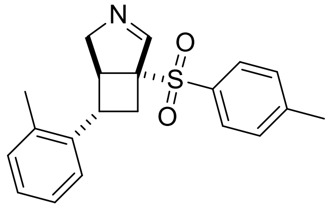

The general procedure (**GP-3**) was followed using **3b** (64.0 mg, 0.20 mmol). Purification by chromatography on silica gel (*n*-hexanes/EtOAc/DCM 50:40:10) yielded **4b** (37.3 mg, 58%) as a white solid. ^{1}**H NMR (400 MHz, CDCl$_3$)** δ = 7.74 (d, *J* = 8.0 Hz, 2H), 7.64 (s, 1H), 7.49 (d, *J* = 7.8 Hz, 1H), 7.35 (d, *J* = 8.0 Hz, 2H), 7.31 – 7.24 (m, 1H), 7.22 – 7.08 (m, 2H), 3.98 (d, *J* = 17.0 Hz, 1H), 3.58 (ddd, *J* = 17.0, 6.4, 2.9 Hz, 1H), 3.39 (t, *J* = 6.3 Hz, 1H), 3.24 – 3.15 (m, 2H), 2.74 – 2.61 (m, 1H), 2.45 (s, 3H), 2.21 (s, 3H). ^{13}**C NMR (101 MHz, CDCl$_3$)** δ = 160.6 (CH), 145.5 (Cq), 139.9 (Cq), 135.3 (Cq), 133.4 (Cq), 130.3 (CH), 130.1 (CH), 128.9 (CH), 126.9 (CH), 126.8 (CH), 126.1 (CH), 76.0 (Cq), 68.2 (CH$_2$), 45.9 (CH), 36.3 (CH), 33.0 (CH$_2$), 21.7 (CH$_3$), 20.1 (CH$_3$). **(ESI)-HRMS** calcd for $C_{20}H_{22}NO_2S$ [M+H]$^+$ 340.1366, found 340.1372.

1R,5S,6S)-6-(4-methoxyphenyl)-1-tosyl-3-azabicyclo[3.2.0]hept-2-ene (4c)

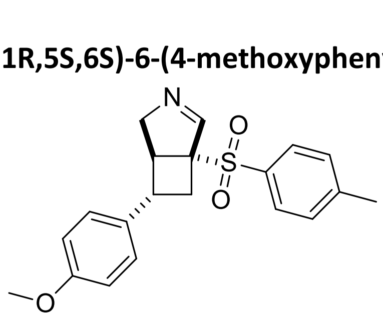

The general procedure (**GP-3**) was followed using **3c** (36.6 mg, 0.10 mmol). Purification by chromatography on silica gel (*n*-hexanes/EtOAc/DCM 50:40:10) yielded **4c** (18.3 mg, 53%) as a yellow solid. ^{1}**H NMR (300 MHz, CDCl$_3$)** δ = 7.74 (d, *J* = 8.3 Hz, 2H), 7.60 (s, 1H), 7.35 (d, *J* = 8.0 Hz, 2H), 7.29 – 7.17 (m, 2H), 6.88 (d, *J* = 8.6 Hz, 2H), 3.96 (d, *J* = 16.7 Hz, 1H), 3.80 (s, 3H),

3.53 (ddd, *J* = 16.8, 6.3, 2.5 Hz, 1H), 3.27 (t, *J* = 6.4 Hz, 1H), 3.15 (dd, *J* = 12.1, 8.7 Hz, 1H), 2.88 (td, *J* = 8.6, 6.6 Hz, 1H), 2.66 (dd, *J* = 12.1, 8.6 Hz, 1H), 2.45 (s, 3H). **^{13}C NMR (75 MHz, CDCl$_3$)** δ = 158.8 (Cq, CH), 145.5 (Cq), 134.3 (Cq), 133.5 (Cq), 130.0 (CH),129.2 (Cq), 128.9 (CH), 128.1 (CH), 114.1 (CH), 67.9 (Cq), 55.3 (CH3), 39.8 (CH), 33.8 (CH), 29.7 (CH), 21.7 (CH3). **(ESI)-HRMS** calcd for $C_{20}H_{21}NKO_3S$ [M+K]$^+$ 394.0874, found 394.0867.

(1R,5S,6S)-6-(4-chlorophenyl)-1-tosyl-3-azabicyclo[3.2.0]hept-2-ene (4d)

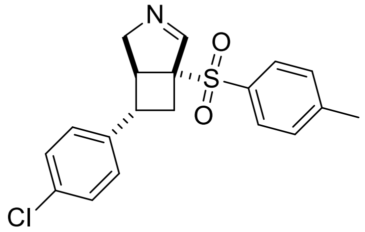

The general procedure (**GP-3**) was followed using **3d** (71.0 mg, 0.20 mmol). Purification by chromatography on silica gel (*n*-hexanes/EtOAc/DCM 50:40:10) yielded **4d** (27.0 mg, 38%) as a white solid. **^{1}H NMR (400 MHz, CDCl$_3$)** δ = 7.73 (d, *J* = 8.0 Hz, 2H), 7.58 (s, 1H), 7.46 – 7.06 (m, 6H), 3.96 (d, *J* = 17.1 Hz, 1H), 3.60 – 3.48 (m, 1H), 3.27 (t, *J* = 6.5 Hz, 1H), 3.14 (dd, *J* = 12.3, 8.8 Hz, 1H), 2.91 (q, *J* = 8.5 Hz, 1H), 2.68 (dd, *J* = 12.3, 8.6 Hz, 1H), 2.45 (s, 3H). **^{13}C NMR (101 MHz, CDCl$_3$)** δ = 161.3 (CH), 145.6 (Cq), 140.7 (Cq), 134.0 (Cq), 133.0 (Cq), 130.1 (CH), 128.9 (CH), 128.9 (CH), 128.4 (CH), 76.0 (Cq), 67.9 (CH$_2$), 47.1 (CH), 39.8 (CH), 33.5 (CH$_2$), 20.7 (CH$_3$). **(ESI)-HRMS** calcd for $C_{19}H_{19}ClNO_2S$ [M+H]$^+$ 360.0820, found 360.0811.

3-((1R,5S,6S)-1-tosyl-3-azabicyclo[3.2.0]hept-2-en-6-yl)benzonitrile (4e)

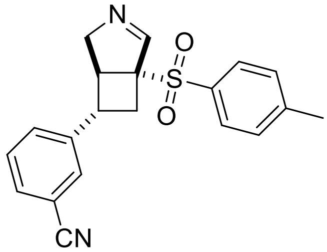

The general procedure (**GP-3**) was followed using **3e** (45.0 mg, 0.13 mmol). Purification by chromatography on silica gel (*n*-hexanes/EtOAc/DCM 50:40:10) yielded **4e** (14.0 mg, 31%) as a yellow solid. **^{1}H NMR (400 MHz, CDCl$_3$)** δ = 7.74 (d, *J* = 8.3 Hz, 2H), 7.66 – 7.58 (m, 2H), 7.56 (dt, *J* = 7.7, 1.4 Hz, 1H), 7.52 – 7.43 (m, 2H), 7.37 (d, *J* = 8.0 Hz, 2H), 3.99 (d, *J* = 17.1 Hz, 1H), 3.61 (ddd, *J* = 17.0, 6.4, 2.9 Hz, 1H), 3.30 (t, *J* = 6.4 Hz, 1H), 3.14 (dd, *J* = 12.3, 8.7 Hz, 1H), 2.98 (td, *J* = 8.6, 6.5 Hz, 1H), 2.73 (dd, *J* = 12.5, 8.8 Hz, 1H), 2.46 (s, 3H).**^{13}C NMR (101 MHz, CDCl$_3$)** δ = 161.1 (CH), 145.9 (Cq), 143.6 (Cq), 133.1 (Cq), 131.5 (CH), 131.0 (CH), 130.6 (CH), 130.2 (CH), 129.8 (CH), 128.9 (CH), 118.6 (Cq), 112.8 (Cq), 76.0 (Cq), 68.0 (CH$_2$), 46.8 (CH), 39.8 (CH), 33.3 (CH$_2$), 21.8 (CH$_3$). **(ESI)-HRMS** calcd for $C_{20}H_{19}N_2O_2S$ [M+H]$^+$ 351.1162, found 350.1149.

(1R,5S,6S)-1-(methylsulfonyl)-6-phenyl-3-azabicyclo[3.2.0]hept-2-ene (4f)

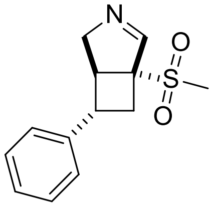

The general procedure (**GP-3**) was followed using **3f** (26.4 mg, 0.1 mmol). Purification by preparative TLC on silica gel (*n*-hexanes/EtOAc/DCM 50:40:10) yielded **4f** (14.5 mg, 55%) as a pale brown solid. **^{1}H NMR (400 MHz, $CDCl_3$)** δ = 7.80 (t, *J* = 2.6 Hz, 1H), 7.40 – 7.14 (m, 5H), 4.29 – 4.09 (m, 2H), 3.41 (t, *J* = 6.2 Hz, 1H), 3.18 – 3.00 (m, 2H), 2.80 – 2.69 (m, 4H). **^{13}C NMR (101 MHz, $CDCl_3$)** δ = 161.4 (CH), 141.0 (Cq), 129.0 (CH), 127.5 (CH), 127.1 (CH), 75.2 (Cq), 68.4 (CH_2), 46.7 (CH), 41.0 (CH), 37.3 (CH_3), 32.7 (CH_2). **(ESI)-HRMS** calcd for $C_{13}H_{16}NO_2S$ $[M+H]^+$ 250.0896, found 249.0905.

(1R,5S,6S)-1-(methylsulfonyl)-6-(o-tolyl)-3-azabicyclo[3.2.0]hept-2-ene (4g)

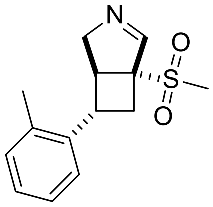

The general procedure (**GP-3**) was followed using **3g** (43.7 mg, 1.7 mmol). Purification by preparative TLC on silica gel (*n*-hexanes/EtOAc/DCM 50:40:10) yielded **4g** (21.3 mg, 49%) as a viscous yellow oil. **^{1}H NMR (400 MHz, $CDCl_3$)** δ = 7.83 (s, 1H), 7.50 (dd, *J* = 7.9, 1.3 Hz, 1H), 7.34 – 7.23 (m, 1H), 7.23 – 7.09 (m, 2H), 4.30 – 4.14 (m, 2H), 3.53 – 3.44 (m, 1H), 3.30 (td, *J* = 8.8, 6.8 Hz, 1H), 3.08 (dd, *J* = 12.3, 9.0 Hz, 1H), 2.81 – 2.71 (m, 4H), 2.25 (s, 3H). **^{13}C NMR (101 MHz, $CDCl_3$)** δ = 161.4 (CH), 139.6 (Cq), 135.2 (Cq), 130.3 (CH), 127.1 (CH), 126.8 (CH), 126.0 (CH), 75.1 (Cq), 68.5 (CH_2), 45.2 (CH), 37.2 (CH_3), 36.9 (CH), 32.2 (CH_2), 20.15 (CH_3). **(ESI)-HRMS** calcd for $C_{14}H_{17}NNaO_2S$ $[M+Na]^+$ 286.0872, found 286.0861.

(1R,5S,6S)-6-phenyl-1-(phenylsulfonyl)-3-azabicyclo[3.2.0]hept-2-ene (4h)

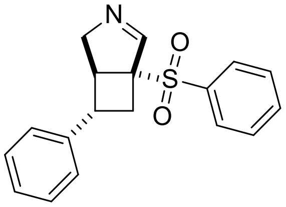

The general procedure (**GP-3**) was followed using **3h** (32 mg, 0.10 mmol). Purification by chromatography on silica gel (*n*-hexanes/EtOAc/DCM 50:40:10) yielded **4h** (17,9 mg, 56%) as a pale yellow solid. **^{1}H NMR (300 MHz, $CDCl_3$)** δ = 7.97 – 7.84 (m, 2H), 7.79 – 7.53 (m, 4H), 7.45 – 7.20 (m, 5H), 4.00 (d, *J* = 17.0 Hz, 1H), 3.53 (ddd, *J* = 17.0, 6.3, 2.9 Hz, 1H), 3.36 (t, *J* = 6.4 Hz, 1H), 3.24 (dd, *J* = 12.1, 8.8 Hz, 1H), 2.97 (td, *J* = 8.6, 6.6 Hz, 1H), 2.72 (dd, *J* = 12.1, 8.5 Hz, 1H). **^{13}C NMR (75 MHz, $CDCl_3$)** δ = 161.3 (CH), 142.1 (Cq), 136.4 (Cq), 134.3 (CH), 129.4 (CH), 128.9 (CH), 128.8 (CH), 127.3 (CH), 127.0 (CH), 76.0 (Cq), 67.9 (CH_2), 47.2 (CH), 40.4 (CH), 33.4 (CH_2). **(ESI)-HRMS** calcd for $C_{18}H_{18}NO_2S$ $[M+H]^+$ 312.1053, found 312.1060.

(1R,5S,6S)-1-(mesitylsulfonyl)-6-phenyl-3-azabicyclo[3.2.0]hept-2-ene (4j)

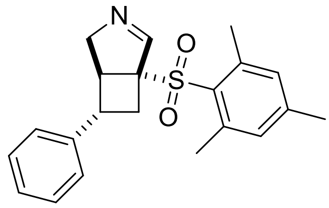

The general procedure (**GP-3**) was followed using **3j** (34.6mg, 0.10 mmol). Purification by chromatography on silica gel (*n*-hexanes/EtOAc/DCM 50:40:10) yielded **4j** (15.8 mg, 46%) as a dark yellow solid. **^{1}H NMR (400 MHz, CDCl$_3$)** δ = 7.47 – 7.19 (m, 6H), 7.01 (s, 2H), 4.14 – 4.05 (m, 1H), 4.01 – 3.90 (m, 1H), 3.53 (t, *J* = 6.2 Hz, 1H), 3.20 (dd, *J* = 12.0, 8.9 Hz, 1H), 2.97 – 2.87 (m, 1H), 2.73 (dd, *J* = 12.0, 8.5 Hz, 1H), 2.65 (s, 6H), 2.34 (s, 3H). **^{13}C NMR (101 MHz, CDCl$_3$)** δ = 161.3 (CH), 144.0 (Cq), 142.3 (Cq), 140.8 (Cq), 132.8 (CH$_2$), 131.3 (Cq), 128.8 (CH), 127.1 (CH), 126.9 (CH), 77.9 (Cq), 68.2 (CH$_2$), 48.0 (CH), 40.0 (CH), 35.44 (CH$_2$), 23.70 (CH$_3$), 21.05 (CH$_3$). **(ESI)-HRMS** calcd for $C_{21}H_{24}NO_2S$ [M+H]$^+$ 354.1522, found 354.1532.

(1R,5S,6S)-1-((4-nitrophenyl)sulfonyl)-6-phenyl-3-azabicyclo[3.2.0]hept-2-ene (4k)

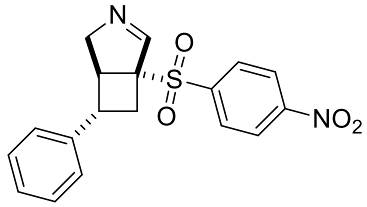

The general procedure (**GP-3**) was followed using **3k** (36.6 mg, 0.10 mmol). Purification by chromatography on silica gel (*n*-hexanes/EtOAc/DCM 50:40:10) yielded **4k** (10.0 mg, 27%) as a yellow solid. **^{1}H NMR (400 MHz, CDCl$_3$)** δ = 8.41 (d, *J* = 8.8 Hz, 2H), 8.08 (d, *J* = 8.8 Hz, 2H), 7.62 (d, *J* = 2.7 Hz, 1H), 7.44 – 7.22 (m, 5H), 4.04 (d, *J* = 17.2 Hz, 1H), 3.56 (ddd, *J* = 17.3, 6.4, 2.9 Hz, 1H), 3.38 (t, *J* = 6.5 Hz, 1H), 3.25 (dd, *J* = 12.3, 8.9 Hz, 1H), 3.00 (td, *J* = 8.7, 6.6 Hz, 1H), 2.74 (dd, *J* = 12.3, 8.6 Hz, 1H). **^{13}C NMR (75 MHz, acetone)** δ = 159.8 (CH), 151.4 (Cq), 142.7 (Cq), 142.3 (Cq), 130.7 (CH), 128.6 (CH), 126.9 (CH), 126.8 (CH), 124.6 (CH), 76.1 (Cq), 67.7 (CH$_2$), 46.8 (CH), 40.1 (CH), 32.9 (CH$_2$). **(ESI)-HRMS** calcd for $C_{18}H_{17}N_2O_4S$ [M+H]$^+$ 357.0904, found 357.0896.

(1R,5S,6S)-1-((4-chlorophenyl)sulfonyl)-6-phenyl-3-azabicyclo[3.2.0]hept-2-ene (4i)

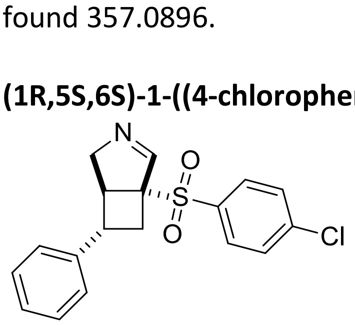

The general procedure (**GP-3**) was followed using **3i** (34.1 mg, 0.10 mmol). Purification by chromatography on silica gel (*n*-hexanes/EtOAc/DCM 50:40:10) yielded **4i** (23.5 mg, 68%) as a white solid. **^{1}H NMR (400 MHz, CDCl$_3$)** δ = 7.80 (d, *J* = 8.6 Hz, 2H), 7.61 (s, 1H), 7.54 (d, *J* = 8.5 Hz, 2H), 7.43 – 7.20 (m, 5H), 4.01 (d, *J* = 17.1 Hz, 1H), 3.56 (ddd, *J* = 17.2, 6.3, 2.9 Hz, 1H), 3.33 (t, *J* = 6.4 Hz, 1H), 3.21 (dd, *J* = 12.2, 8.8 Hz, 1H), 2.96 (td, *J* = 8.7, 6.6 Hz, 1H), 2.71 (dd, *J* = 12.2, 8.6 Hz, 1H).**^{13}C NMR (75 MHz, CDCl$_3$)** δ = 161.0 (CH), 141.9 (Cq), 141.3 (Cq), 134.9 (Cq), 130.3

(CH), 129.8 (CH), 128.8 (CH), 127.3 (CH), 126.9 (CH), 76.1 (Cq), 68.0 (CH_2), 47.2 (CH), 40.4 (CH), 33.4 (CH_2). **(ESI)-HRMS** calcd for $C_{18}H_{17}ClNO_2S$ $[M+H]^+$ 346.0663, found 346.0653.

(1R,5S,6S)-1-(naphthalen-1-ylsulfonyl)-6-phenyl-3-azabicyclo[3.2.0]hept-2-ene (4l)

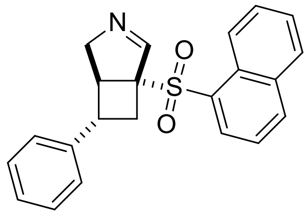

The general procedure (**GP-3**) was followed using **3l** (72.3 mg, 0.20 mmol). Purification by chromatography on silica gel (*n*-hexanes/EtOAc/DCM 50:40:10) yielded **4l** (30.3 mg,42%) as a pale brown solid. **^{1}H NMR (300 MHz, $CDCl_3$)** δ = 8.45 (s, 1H), 8.07 – 7.88 (m, 3H), 7.83 (dd, *J* = 8.6, 1.9 Hz, 1H), 7.76 – 7.56 (m, 3H), 7.40 – 7.20 (m, 5H), 3.97 (d, *J* = 16.9 Hz, 1H), 3.54 (ddd, *J* = 16.8, 6.3, 2.8 Hz, 1H), 3.43 (t, *J* = 6.4 Hz, 1H), 3.28 (dd, *J* = 12.2, 8.8 Hz, 1H), 2.96 (td, *J* = 8.7, 6.5 Hz, 1H), 2.72 (dd, *J* = 12.2, 8.6 Hz, 1H). **^{13}C NMR (75 MHz, $CDCl_3$)** δ = 161.3 (CH), 142.2 (Cq), 135.5 (Cq), 133.5 (Cq), 132.1 (Cq), 130.9 (CH), 129.7 (CH), 129.7 (CH), 129.5 (CH), 128.8 (CH), 128.0 (CH), 127.9 (CH), 127.3 (CH), 127.0 (CH), 123.1 (CH), 77.3 (Cq), 68.0 (CH_2), 47.3 (CH), 40.5 (CH), 33.6 (CH_2). **(ESI)-HRMS** calcd for $C_{22}H_{20}NO_2S$ $[M+H]^+$ 362.1209, found 361.1213.

(1R,6R,7S)-7-phenyl-1-tosyl-3-azabicyclo[4.2.0]oct-2-ene (4m)

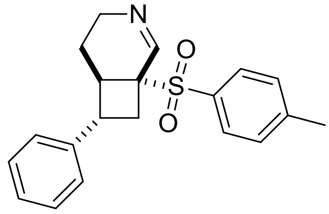

The general procedure (**GP-3**) was followed using **3m** (46 mg, 0.13mmol). Purification by chromatography on silica gel (gradient from *n*-hexanes/EtOAc/DCM 60:30:10 to 50:40:10) yielded **4m** (12.1 mg, 26%) as a white solid. **^{1}H NMR (400 MHz, $CDCl_3$)** δ = 8.21 (s, 1H), 7.75 (d, *J* = 8.3 Hz, 2H), 7.50 – 7.11 (m, 8H), 3.79 (dd, *J* = 18.3, 5.6 Hz, 1H), 3.67 – 3.53 (m, 1H), 3.32 (t, *J* = 4.4 Hz, 1H), 3.21 – 3.06 (m, 2H), 2.61 – 2.48 (m, 1H), 2.45 (s, 3H), 1.55 – 1.44 (m, 1H), 0.95 – 0.80 (m, 1H). **^{13}C NMR (101 MHz, $CDCl_3$)** δ = 157.7 (CH), 145.5 (Cq), 142.0 (Cq), 133.4 (Cq), 130.1 (CH), 129.2 (CH), 128.8 (CH), 127.2 (CH), 127.1 (CH) (CH), 56.9 (Cq), 45.1 (CH_2), 40.1 (CH), 36.3 (CH), 34.0 (CH_2), 21.8 (CH_3), 19.8 (CH_2). **(ESI)-HRMS** calcd for $C_{20}H_{21}NNaO_2S$ $[M+H]^+$ 362.1185, found 362.1191.

X-Ray crystallography data

Single crystal Data for **2a**, **2**′-**I**, and **4a** were collected with a Bruker D8 diffractometer equipped with PhotonII area detector, using a CuKα or a MoKα microfocus radiation source. Crystals were grown by vapor diffusion from hexane/acetone (**2a** and **4a**) and hexane/THF (**2'-I**) and mounted on a cryoloop and the data collection strategy covered the sphere of reciprocal space. Absorption corrections were applied using the program SADABS.[8] The structure was solved with the SHELXT code.[9] Fourier analysis and refinement were performed by the full-matrix least-squares methods based on F2 using SHELXL-2014[10], implemented in in Olex2[11]. All the non-H atoms were refined with anisotropic displacement parameters.

Crystal data and structure refinement for **2a**, **2'**-I, and **4a**

	**2a**	**2'**-I	**4a**
Empirical formula	$C_{20}H_{21}NO_2S$	$C_{26}H_{25}NO_2S$	$C_{19}H_{19}NO_2S$
Formula weight	339.44	415.53	192.25
Temperature/K	190	110	200
Crystal system	triclinic	monoclinic	monoclinic
Space group	P-1	$P2_1/n$	$P2_1/n$
a/Å	6.0300(11)	12.4094(10)	5.4523(5)
b/Å	14.762(3)	8.9350(8)	10.5750(10)
c/Å	29.542(7)	19.7043(17)	29.125(3)
α/°	96.731(5)	90	90
β/°	93.220(5)	105.200(3)	95.261(3)
γ/°	91.700(6)	90	90
Volume/$Å^3$	2605.6(10)	2108.3(3)	1672.2(3)
Z	6	4	4
$\rho_{calc}g/cm^3$	1.298	1.309	0.191
μ/mm^{-1}	0.198	0.177	0.380
F(000)	1080.0	880.0	101.0
Crystal size/mm^3	0.25 × 0.19 × 0.11	0.12 × 0.08 × 0.02	0.24 × 0.23 × 0.12
Radiation	MoKα (λ = 0.71073)	MoKα (λ = 0.71073)	CuKα (λ = 1.54184)
2Θ range for data coll./°	5.562 to 52.04	4.47 to 51.566	6.094 to 149.08
Index ranges	-7 ≤ h ≤ 7, -18 ≤ k ≤ 18, -36 ≤ l ≤ 36	-15 ≤ h ≤ 13, -10 ≤ k ≤ 10, -24 ≤ l ≤ 24	-6 ≤ h ≤ 6, -13 ≤ k ≤ 13, -36 ≤ l ≤ 33
Reflections collected	69975	33876	50378
Independent reflections	10235 [R_{int} = 0.0629, R_{sigma} = 0.0352]	4027 [R_{int} = 0.1284, R_{sigma} = 0.0674]	3380 [R_{int} = 0.0511, R_{sigma} = 0.0202]
Data/restraints/parameters	10235/0/652	4027/0/276	3380/0/209
Goodness-of-fit on F^2	1.034	1.084	1.011
Final R indexes [I>=2σ (I)]	R_1 = 0.0438, wR_2 = 0.1008	R_1 = 0.0588, wR_2 = 0.1319	R_1 = 0.0512, wR_2 = 0.1487
Largest diff. peak/hole / e $Å^{-3}$	0.23/-0.38	0.43/-0.39	0.18/-0.39

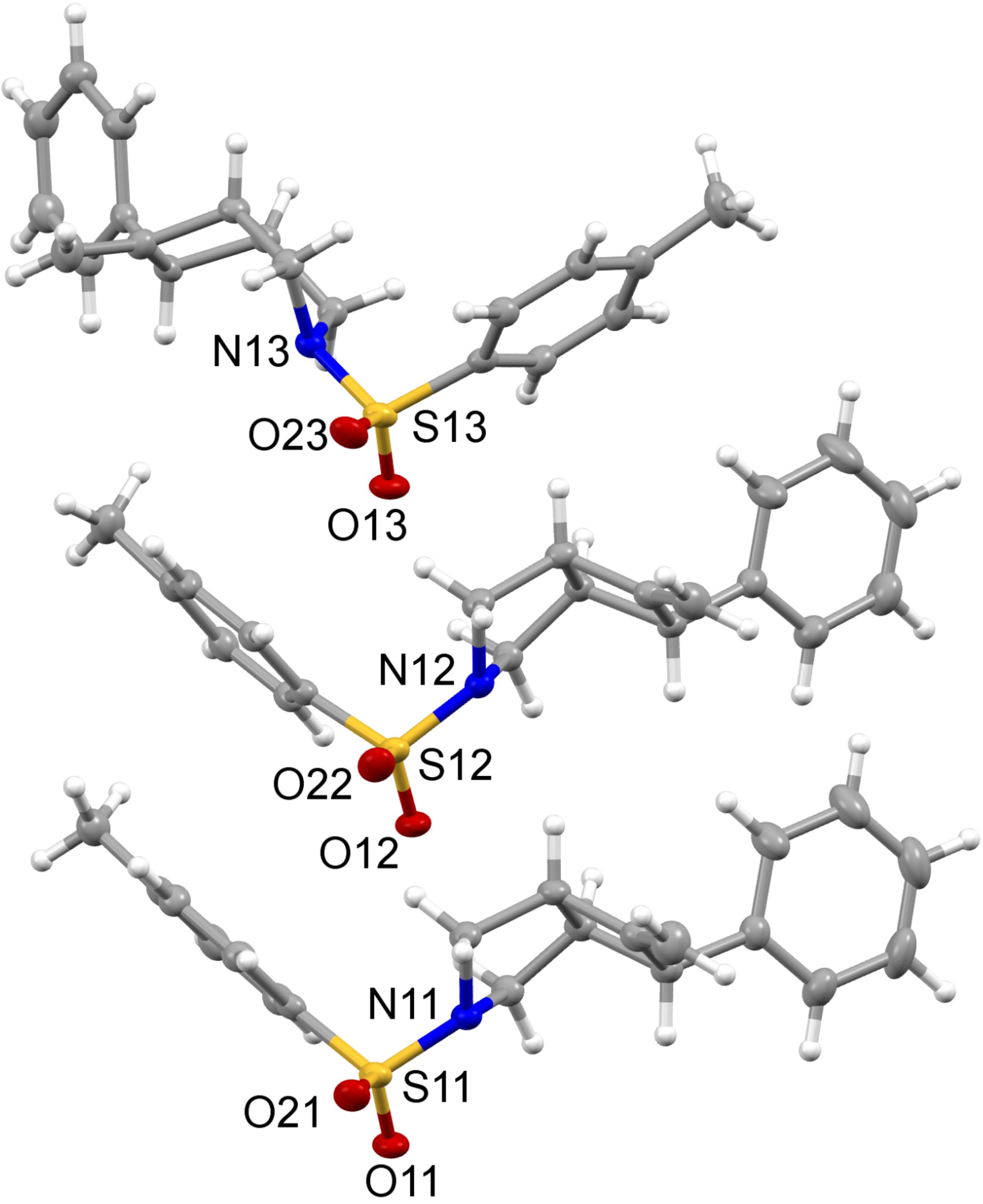

Figure S1. Molecular structure of **2a** with thermal ellipsoids depicted at the 30% probability level. The asymmetric unit comprises three molecular entities.

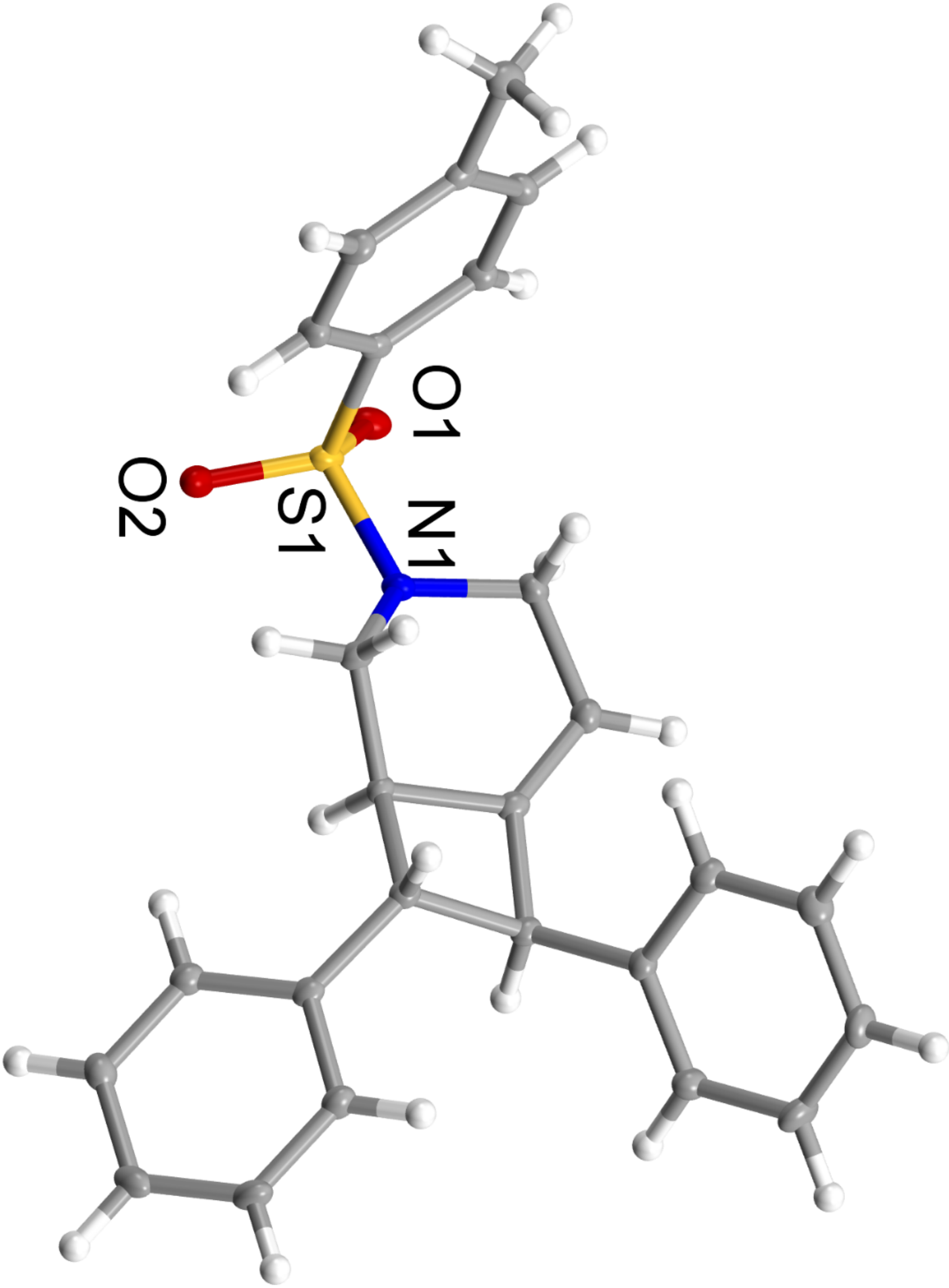

Figure S2. Molecular structure of **2**’-l with thermal ellipsoids depicted at the 30% probability level.

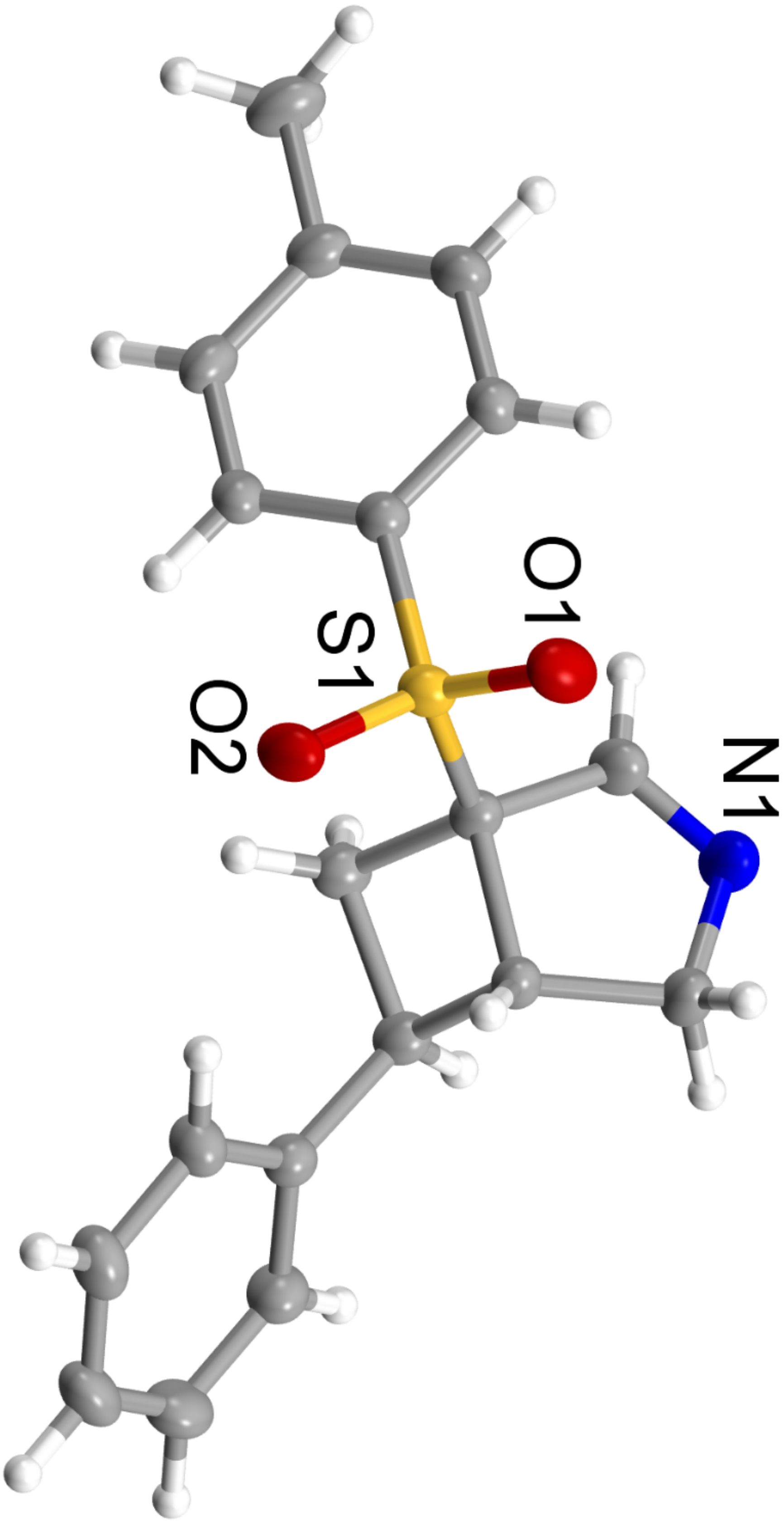

Figure S3. Molecular structure of **4a** with thermal ellipsoids depicted at the 30% probability level.

Copies of NMR spectra

^{1}H NMR (400 MHz, $CDCl_3$)

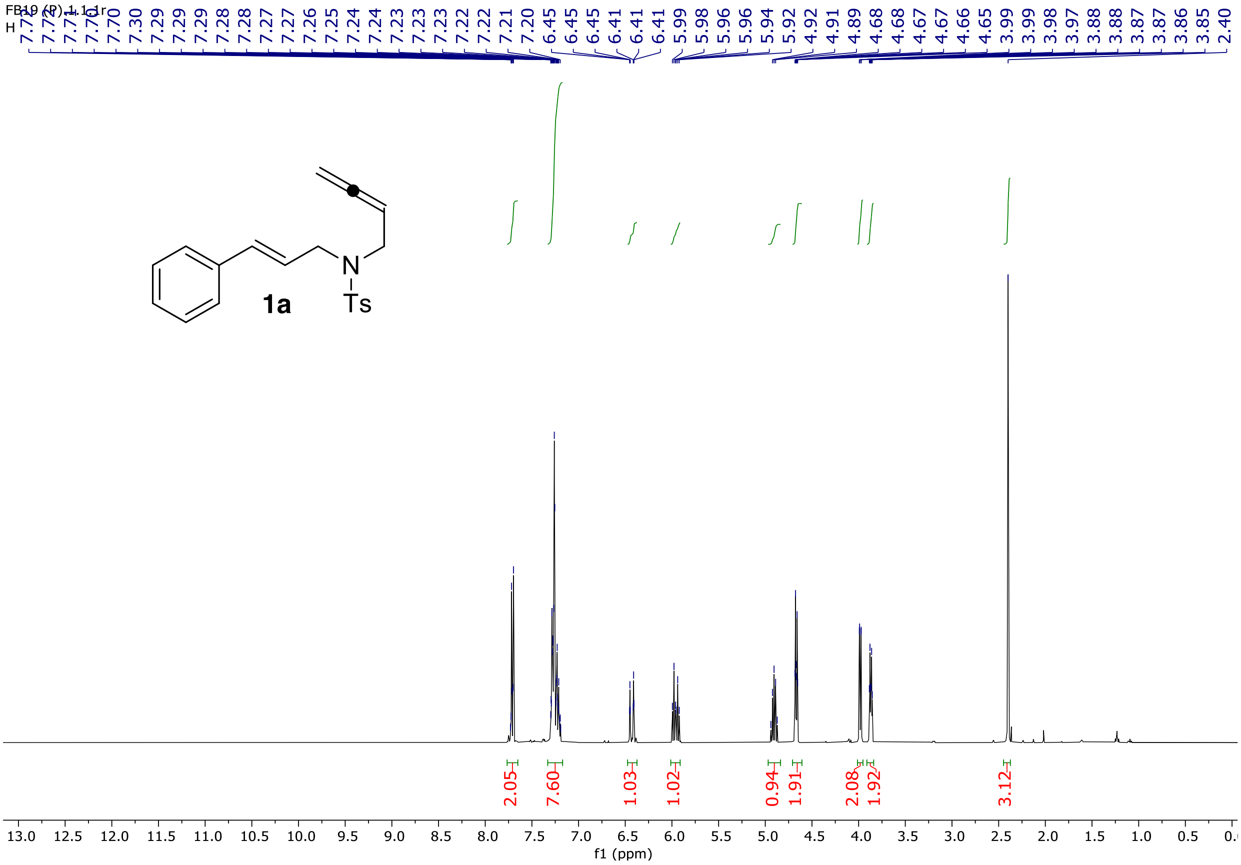

^{13}C NMR (101 MHz, $CDCl_3$)

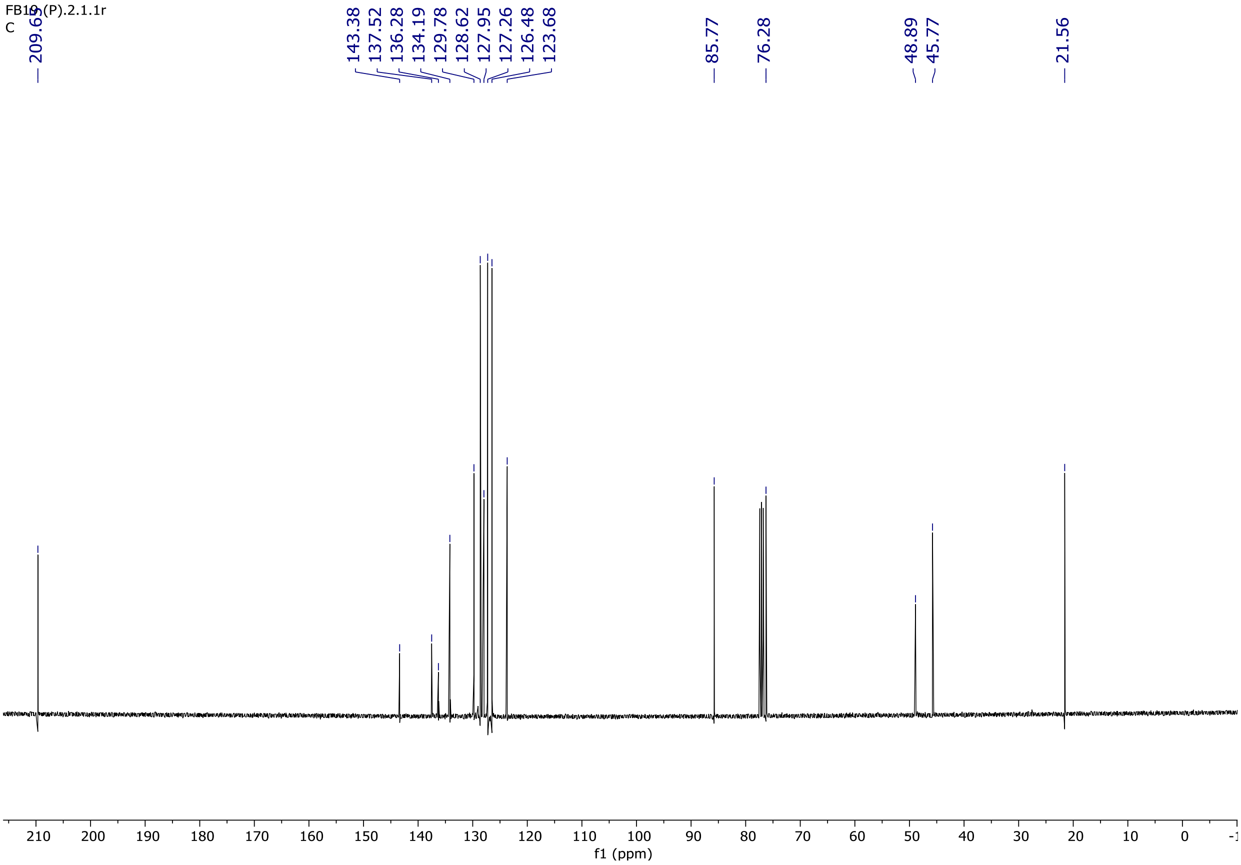

^{1}H NMR (300 MHz, acetone)

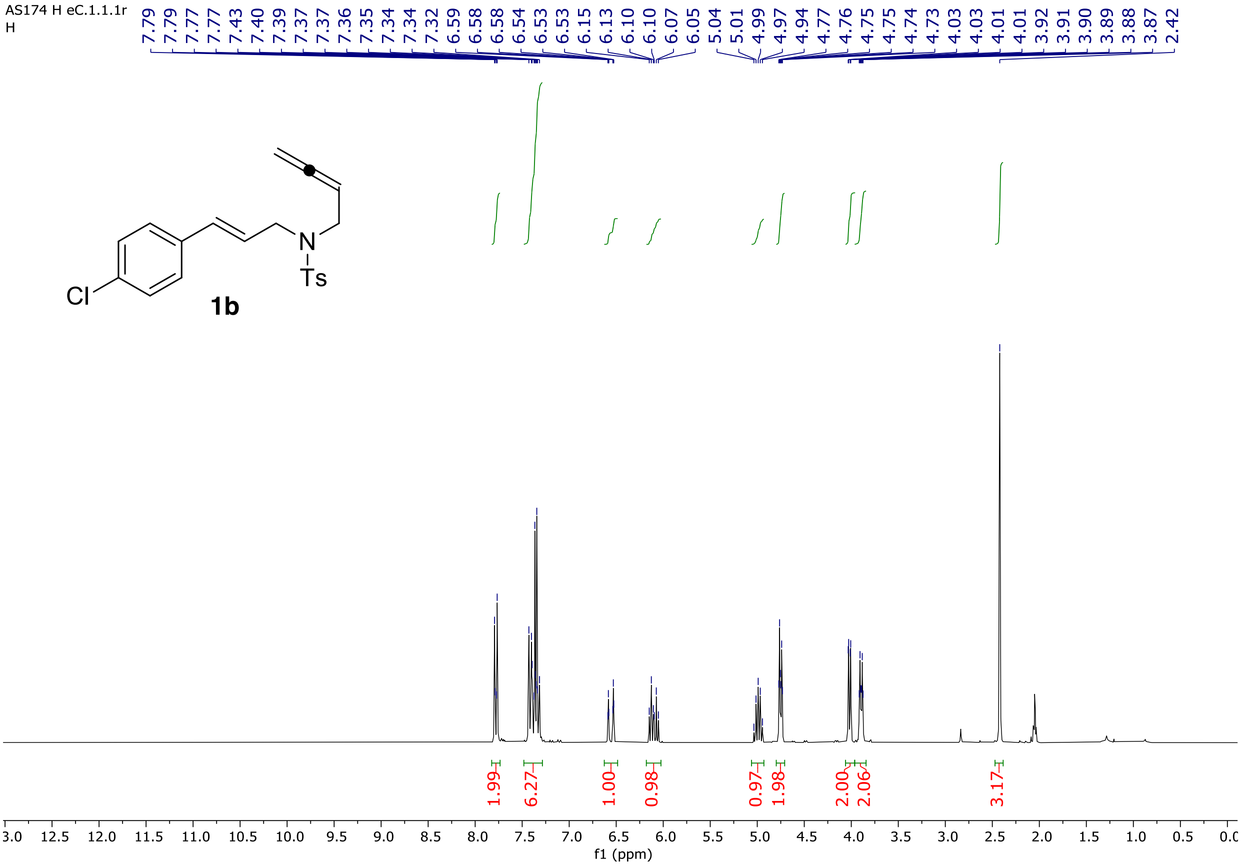

^{13}C NMR (75 MHz, acetone)

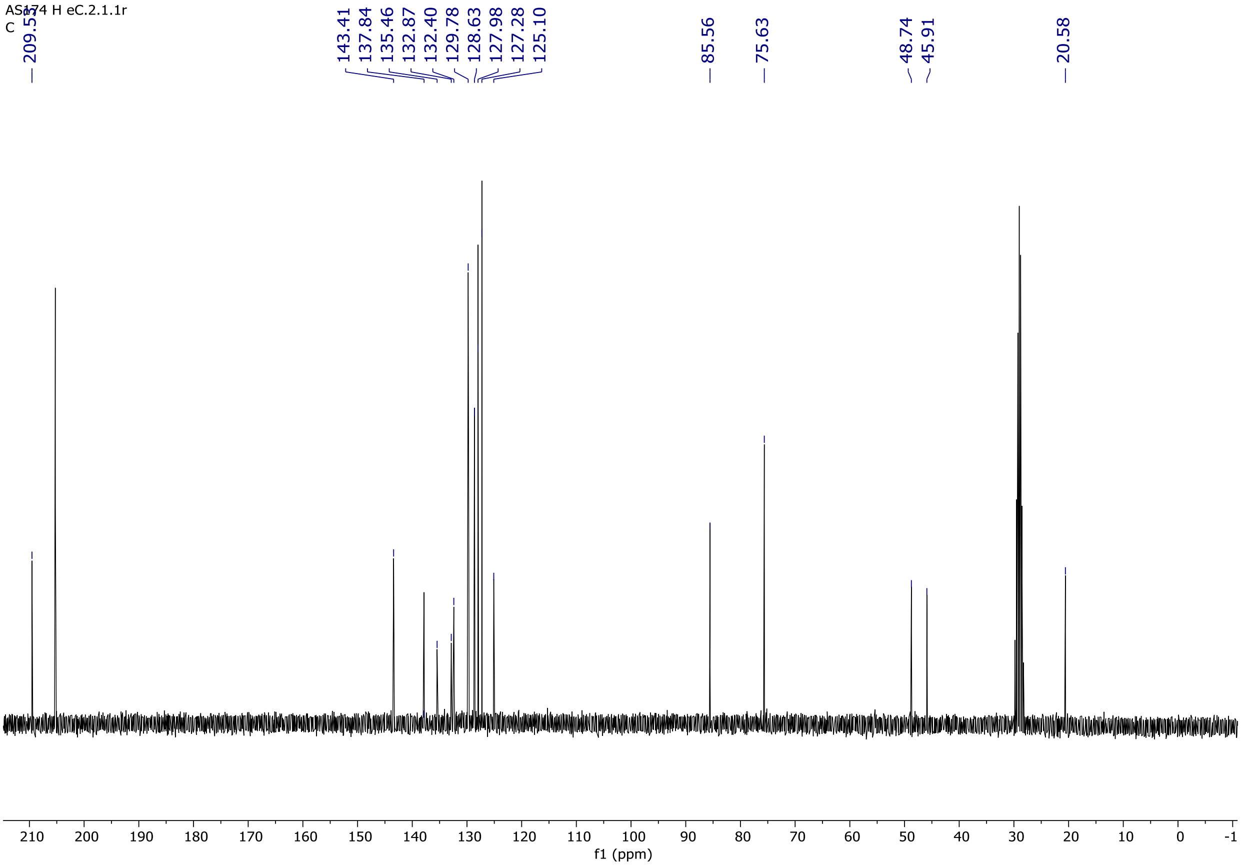

^{1}H NMR (400 MHz, $CDCl_3$)

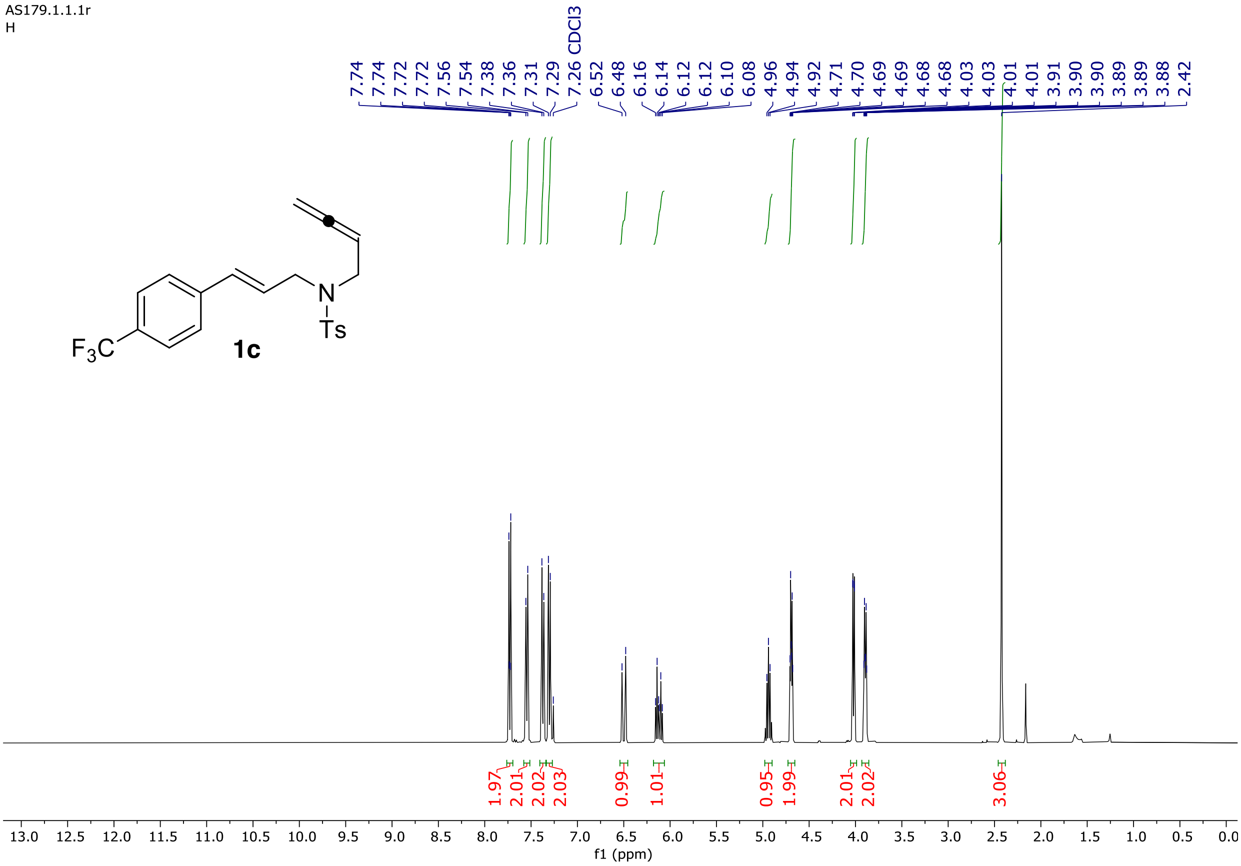

^{13}C NMR (101 MHz, $CDCl_3$)

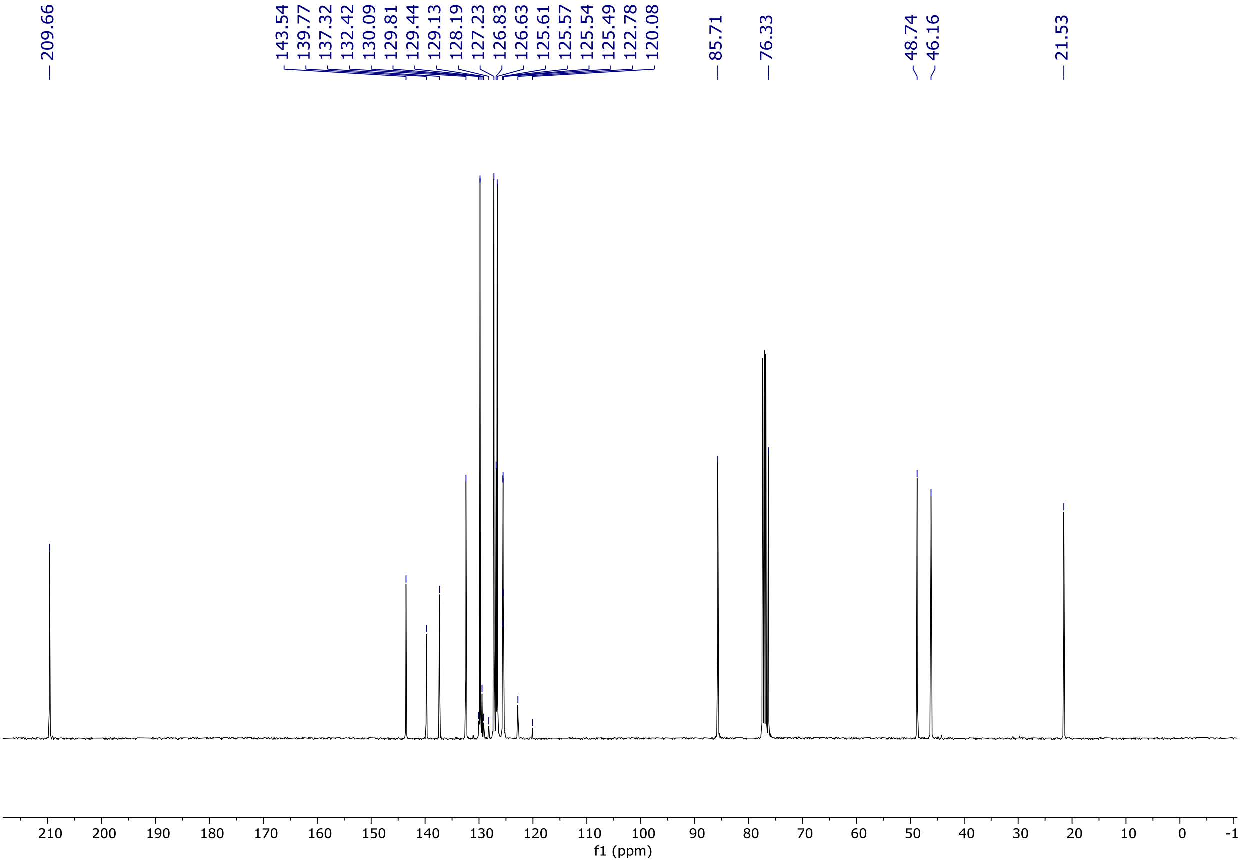

^{19}F NMR (564 MHz, CDCl$_3$)

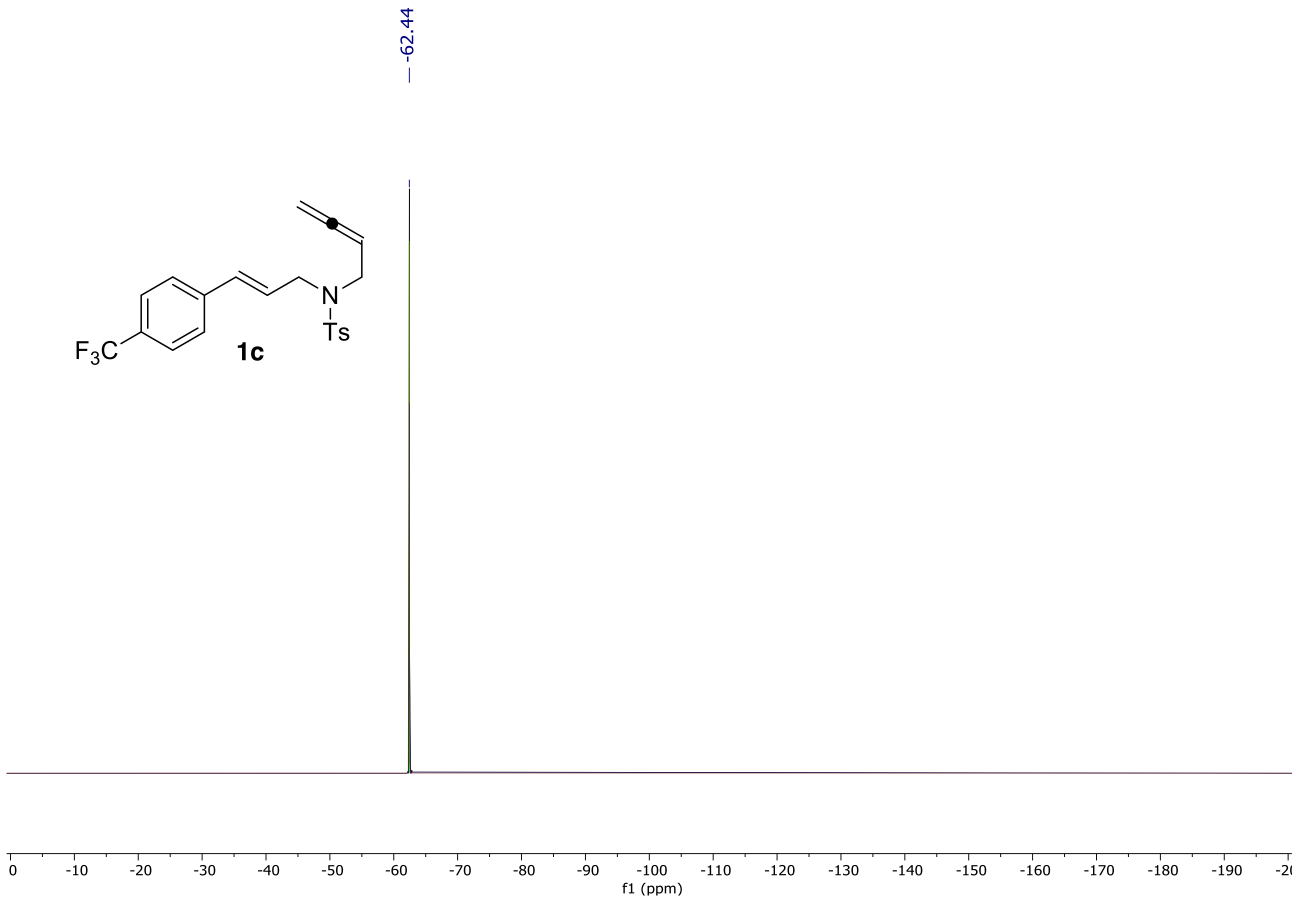

^{1}H NMR (400 MHz, $CDCl_3$)

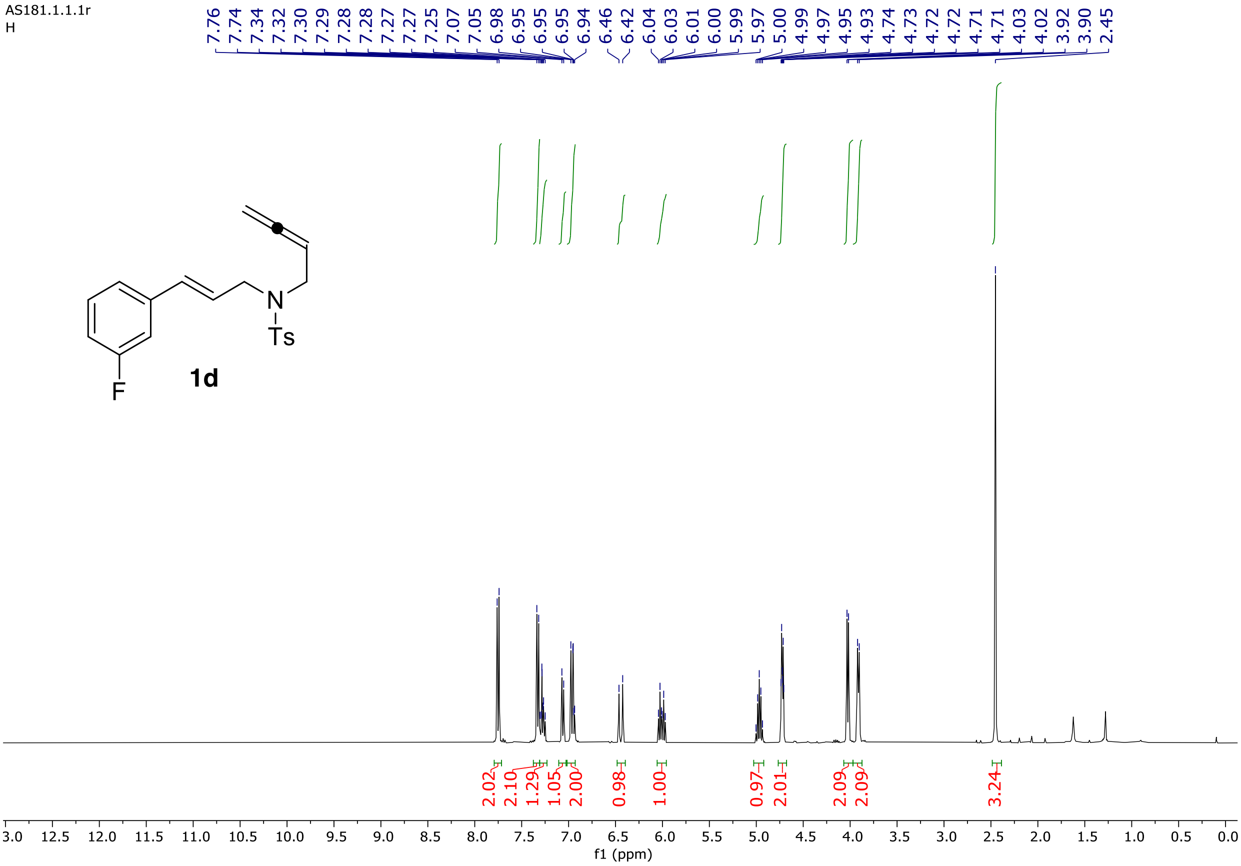

1d

^{13}C NMR (101 MHz, $CDCl_3$)

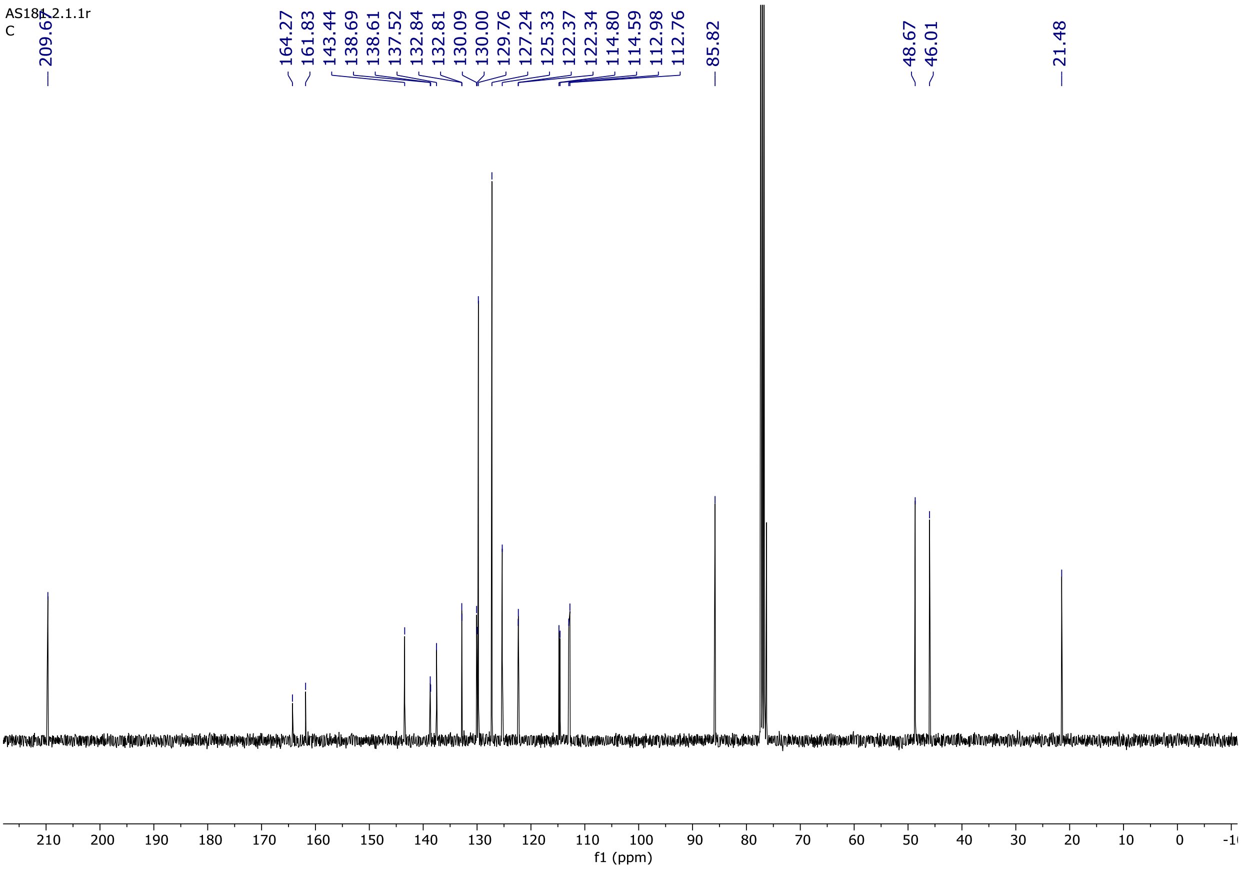

^{19}F NMR (564 MHz, $CDCl_3$)

AS181
single_pulse

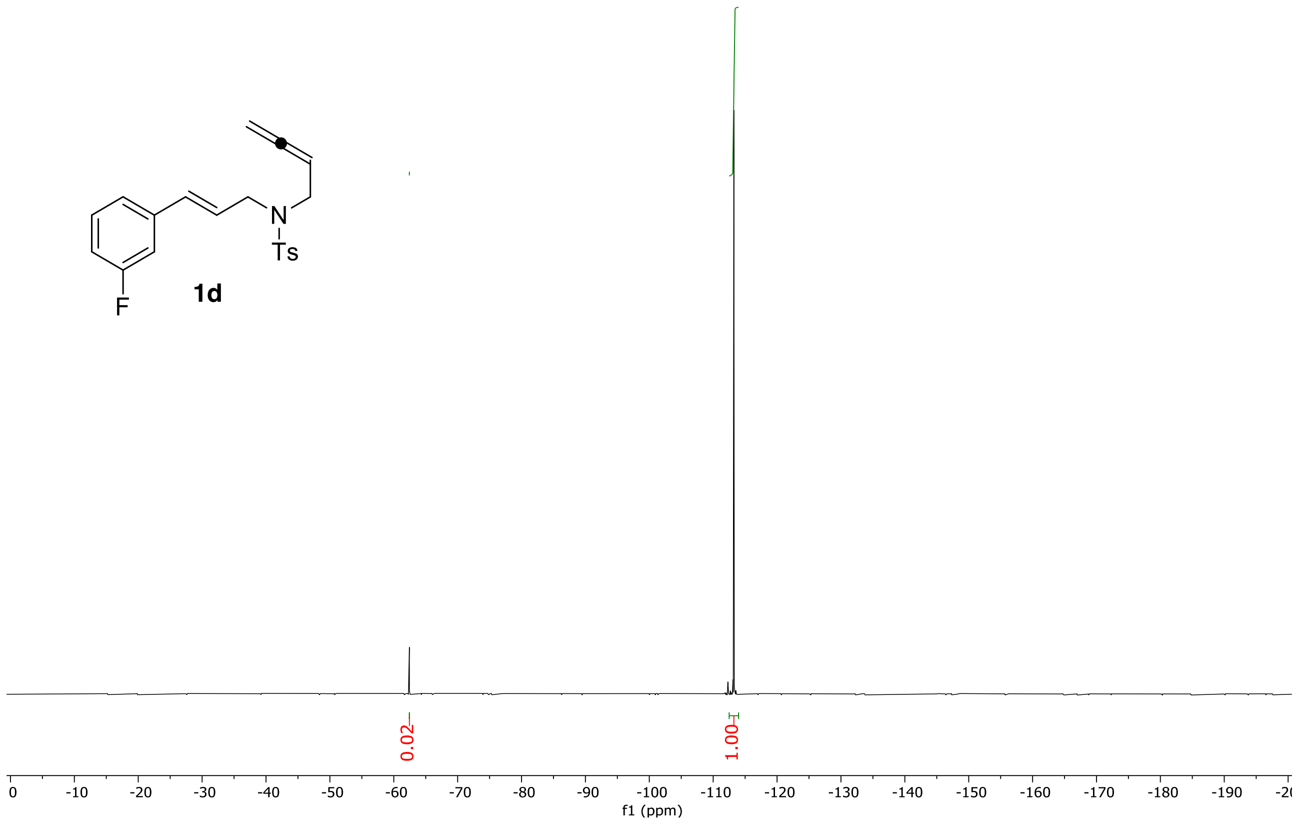

^{1}H NMR (400 MHz, $CDCl_3$)

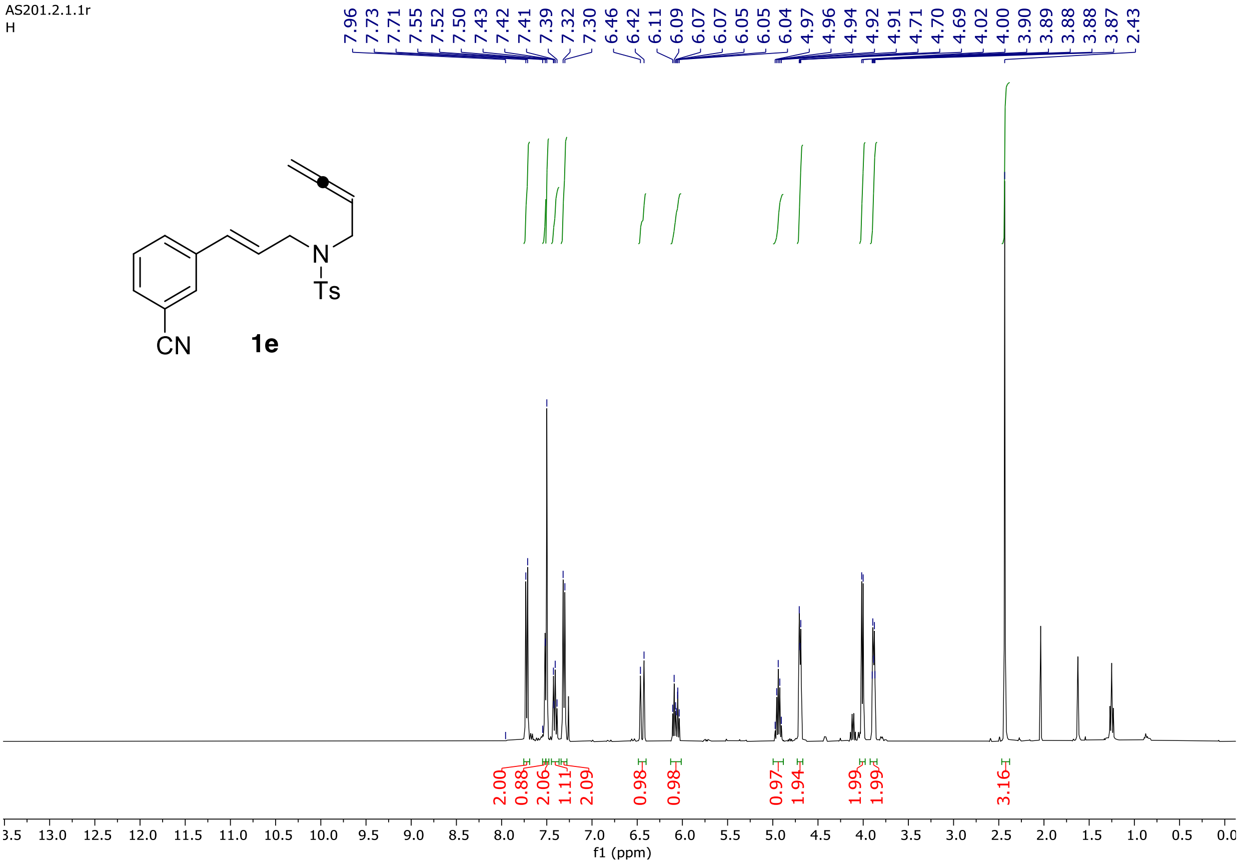

^{13}C NMR (101 MHz, $CDCl_3$)

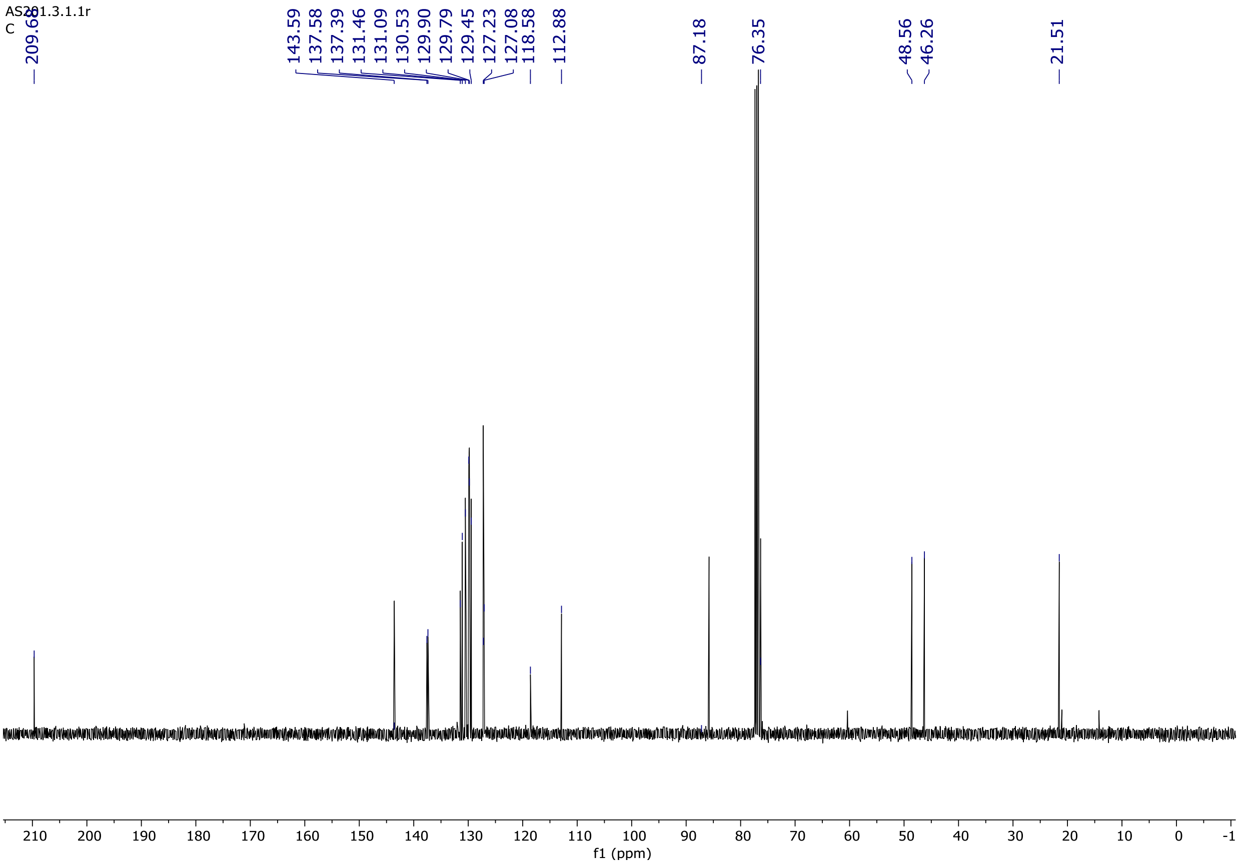

^{1}H NMR (400 MHz, $CDCl_3$)

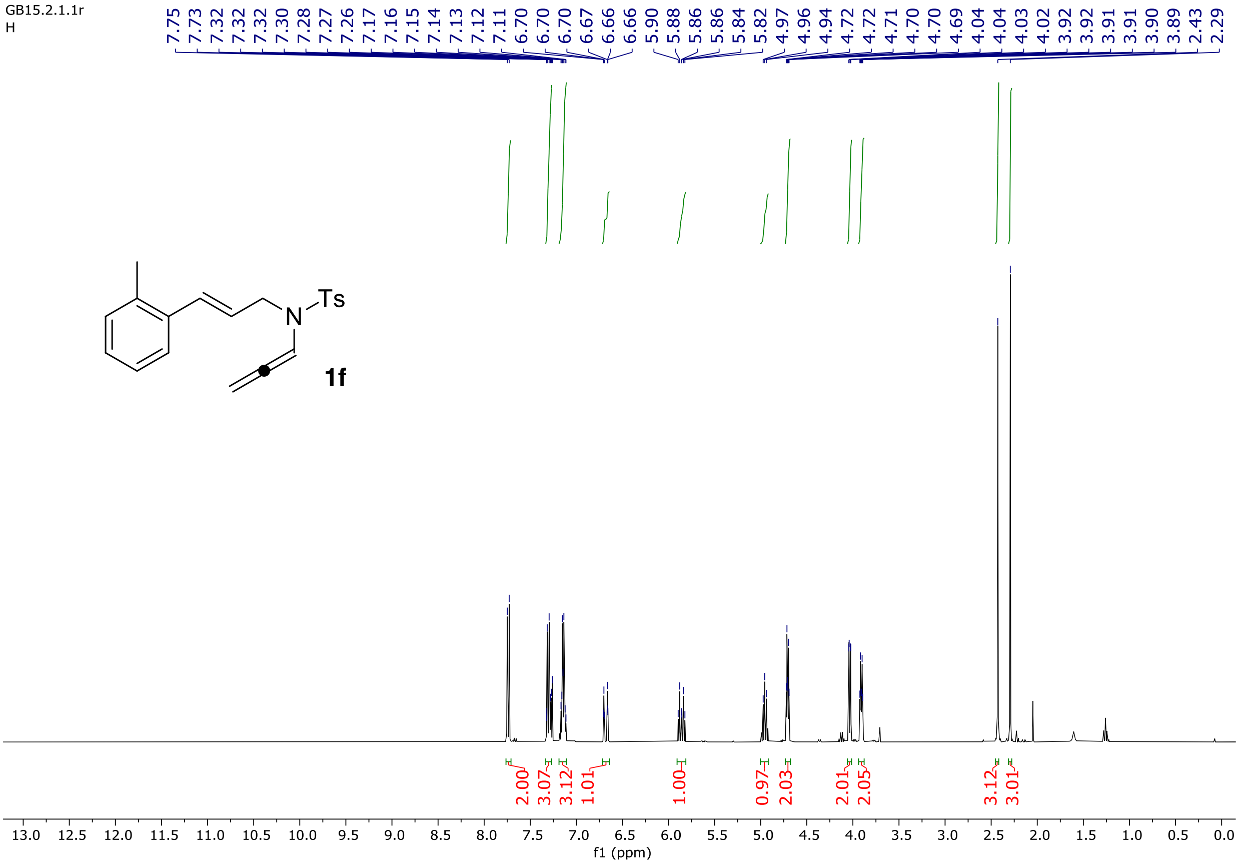

^{13}C NMR (101 MHz, $CDCl_3$)

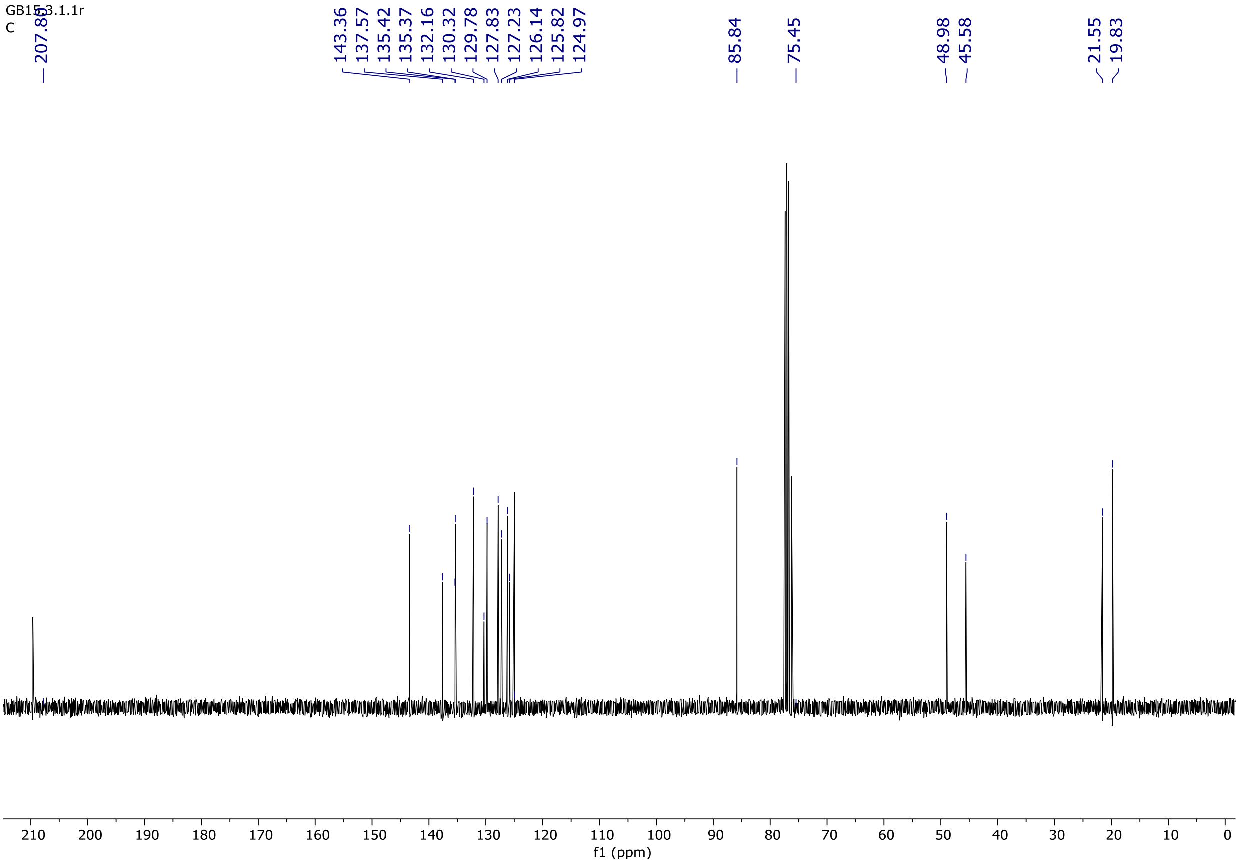

^{1}H NMR (400 MHz, $CDCl_3$)

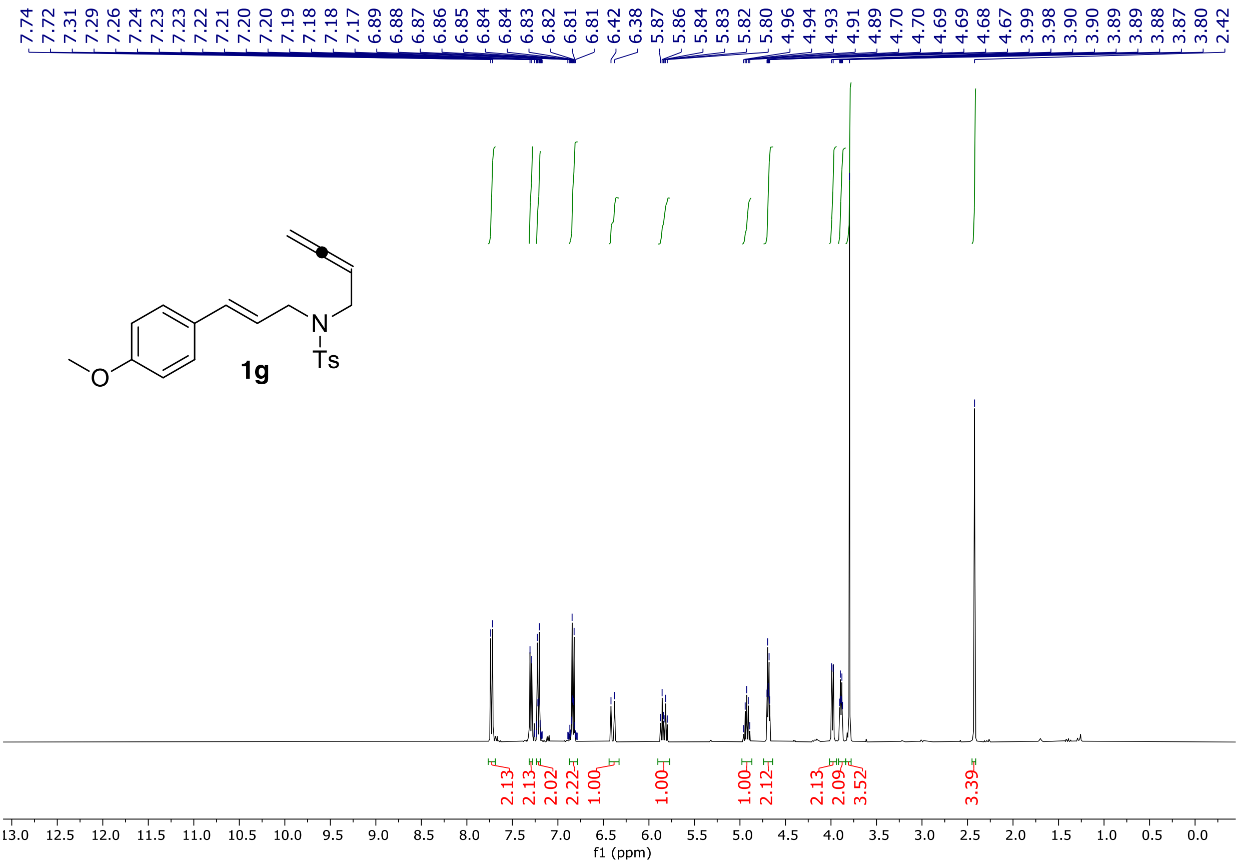

^{13}C NMR (101 MHz, $CDCl_3$)

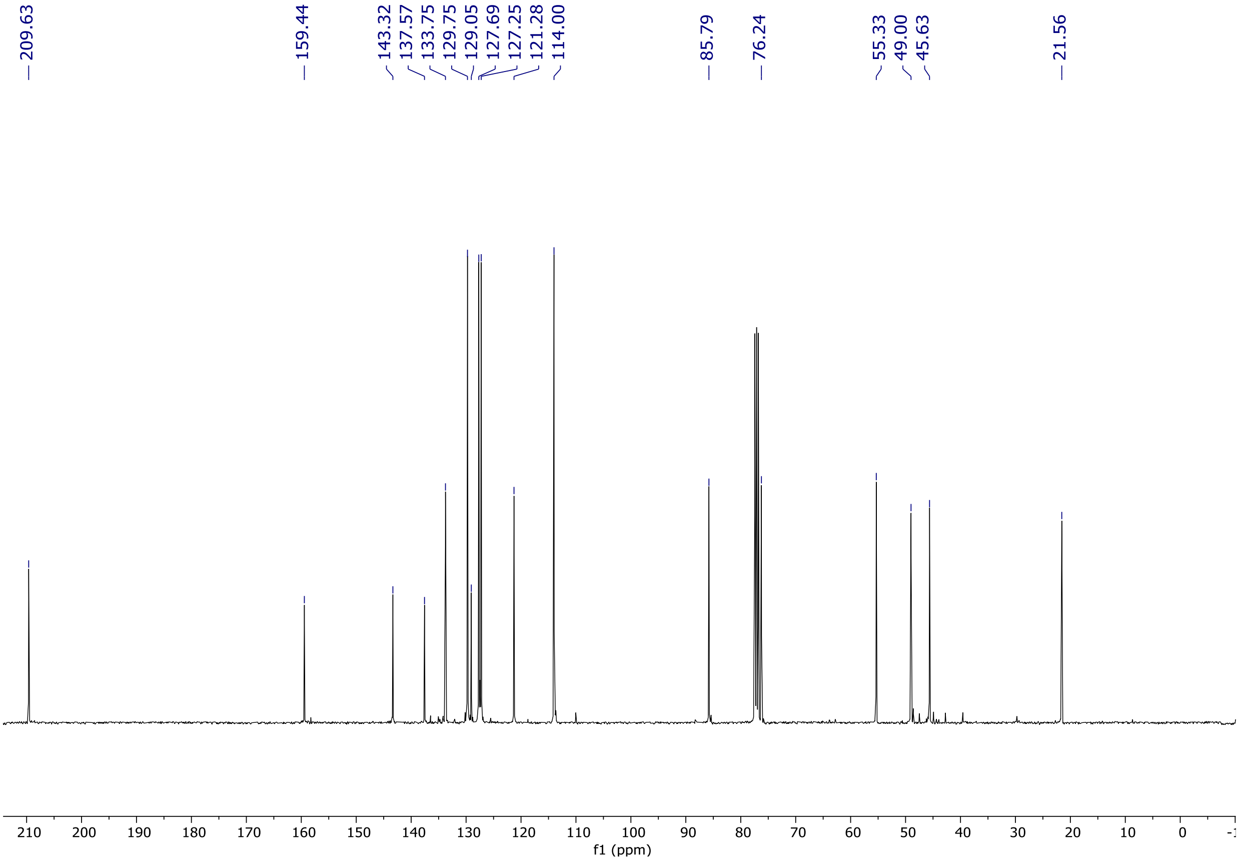

^{1}H NMR (400 MHz, $CDCl_3$)

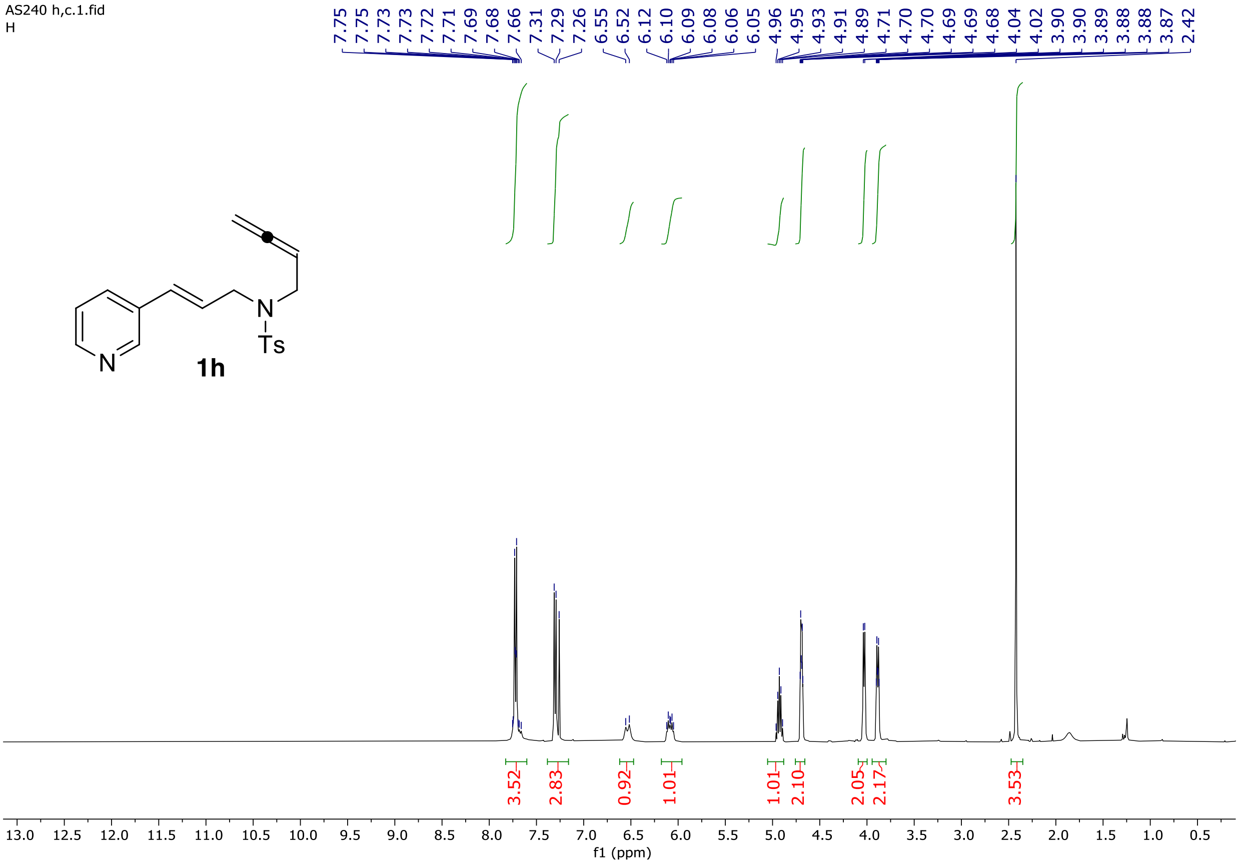

^{13}C NMR (101 MHz, $CDCl_3$)

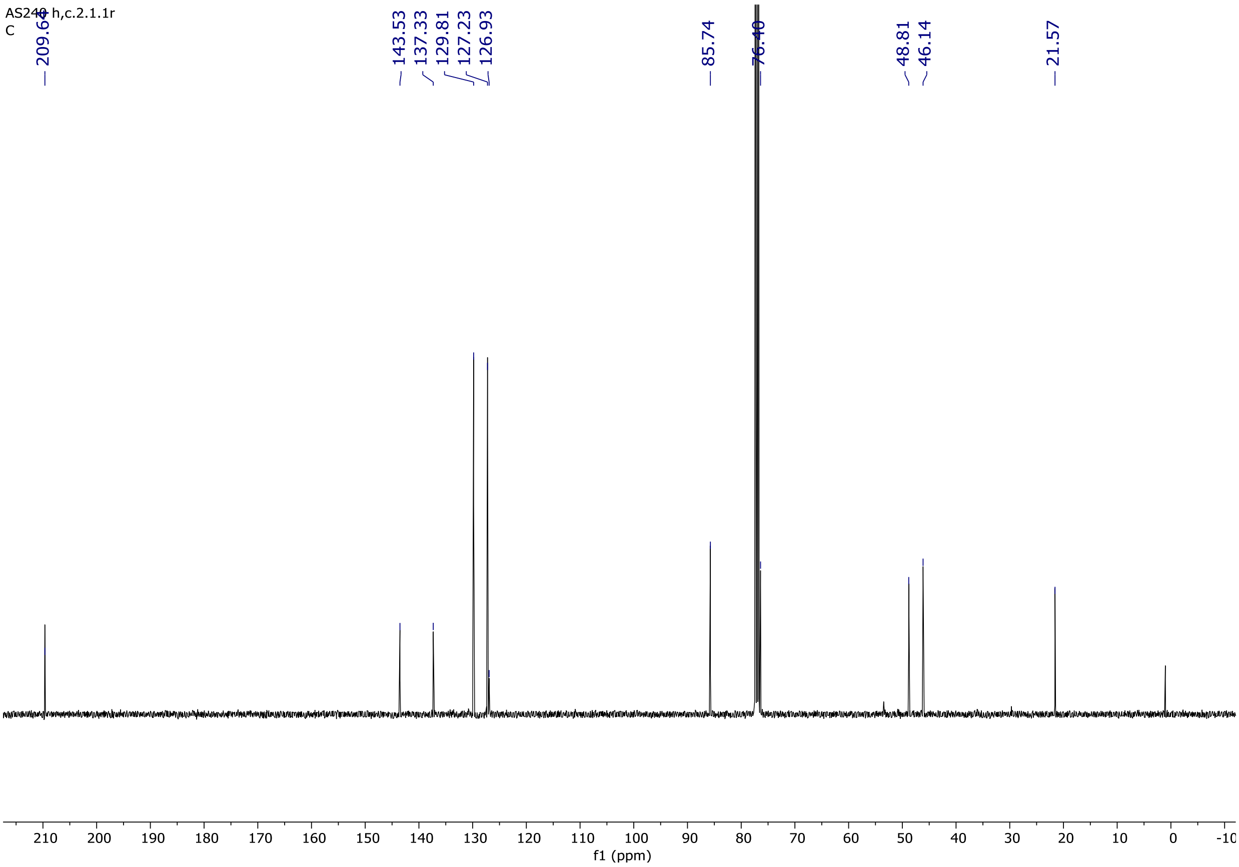

^{1}H NMR (400 MHz, $CDCl_3$)

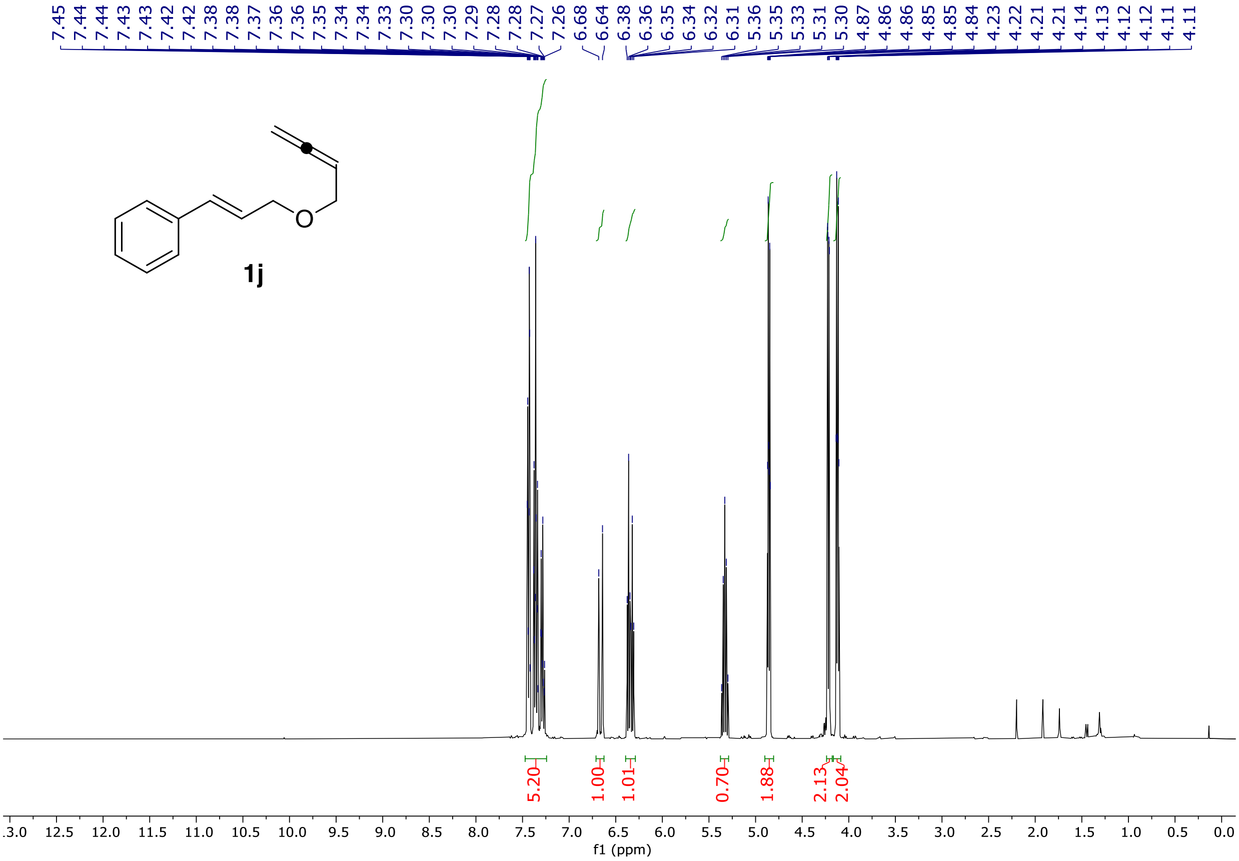

^{13}C NMR (101 MHz, $CDCl_3$)

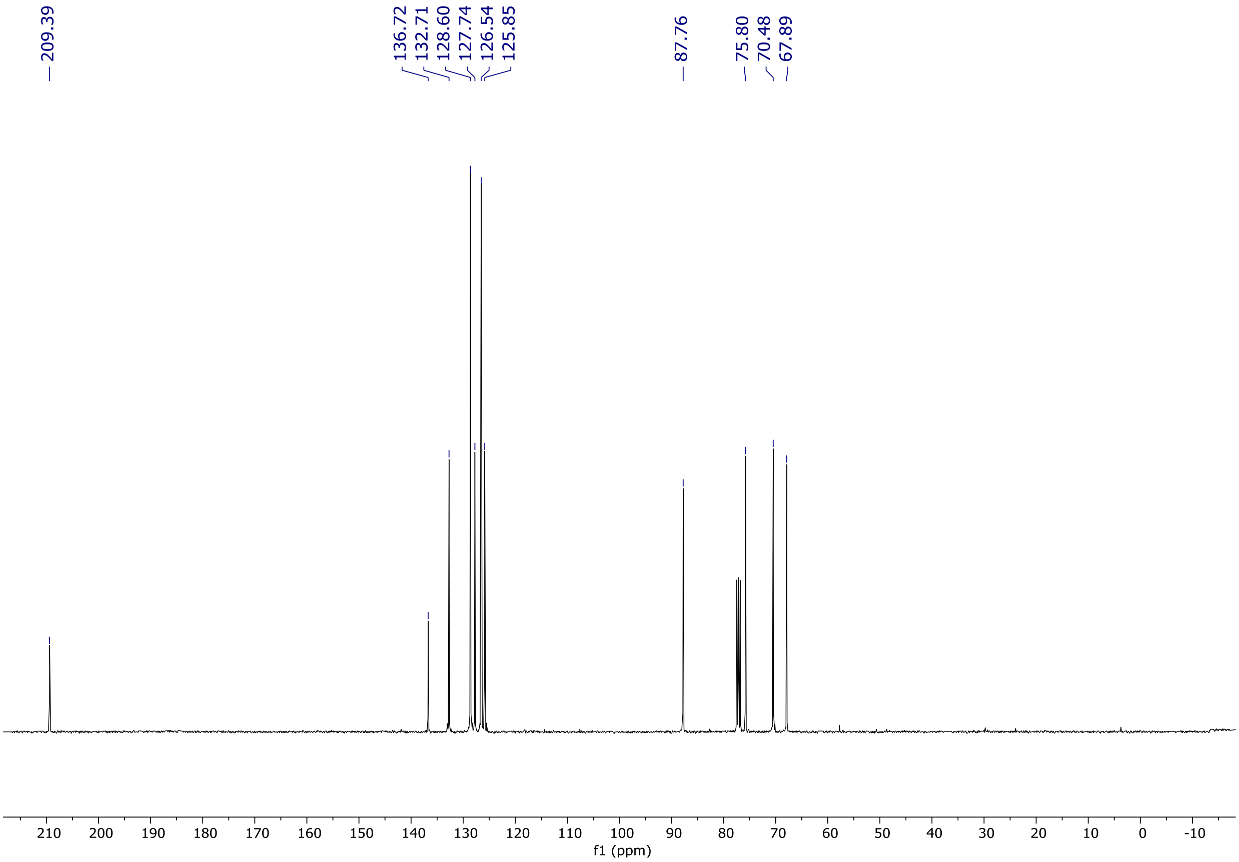

^{1}H NMR (400 MHz, $CDCl_3$)

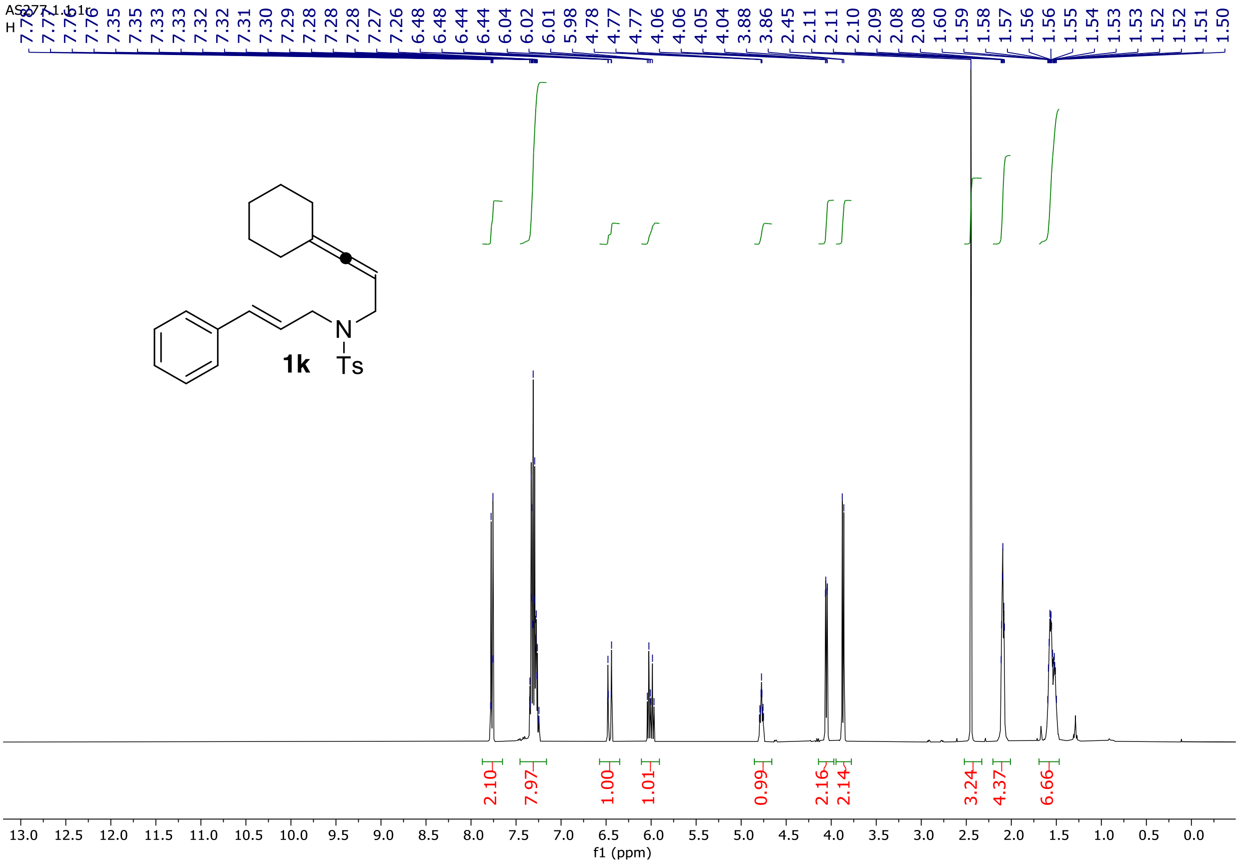

^{13}C NMR (101 MHz, $CDCl_3$)

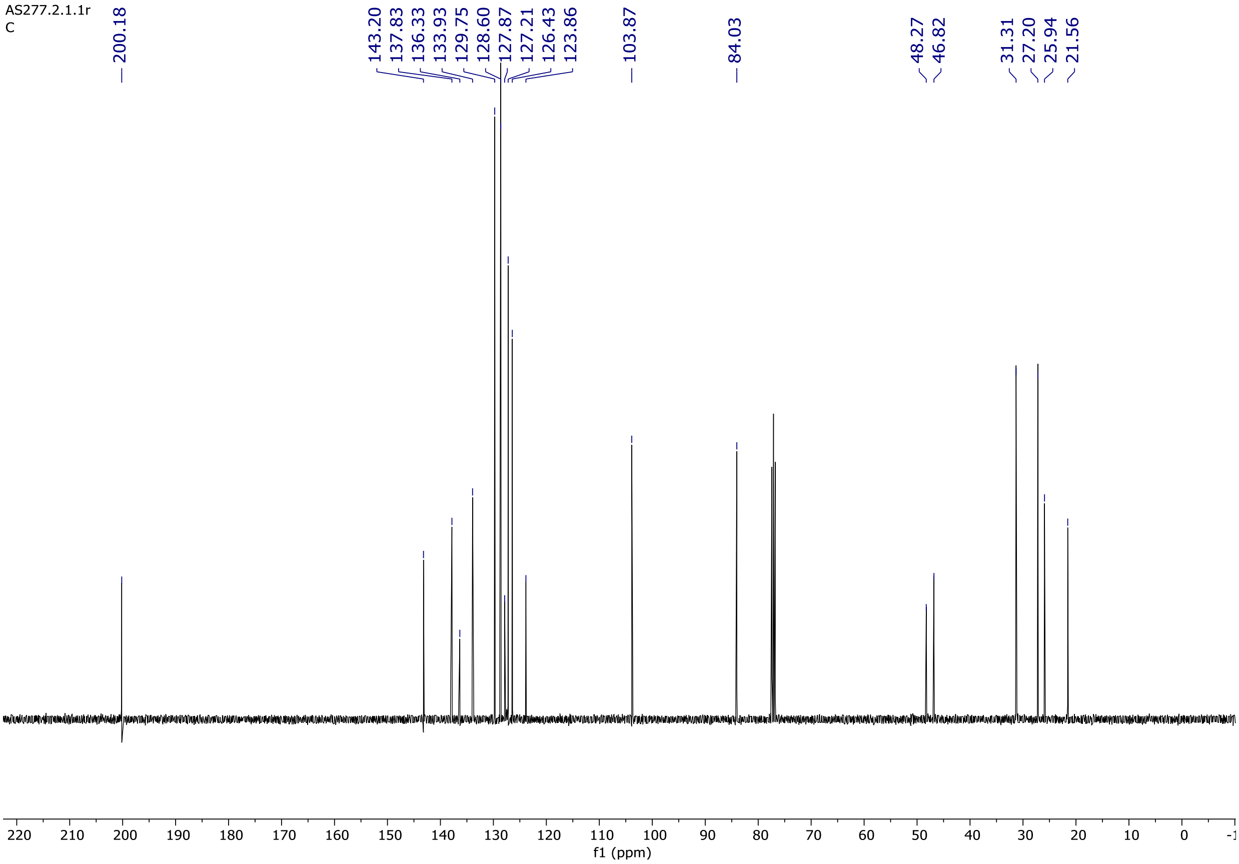

^{1}H NMR (400 MHz, acetone)

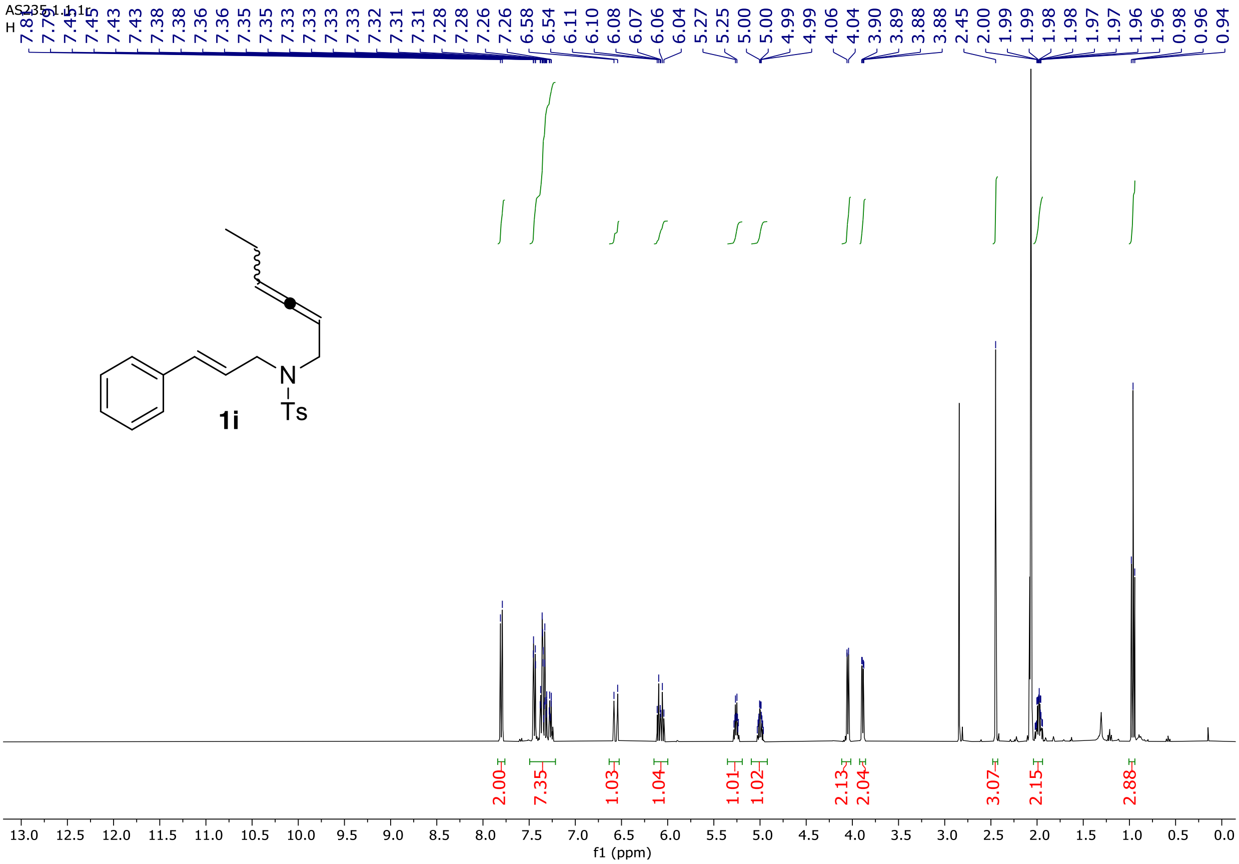

^{13}C NMR (101 MHz, acetone)

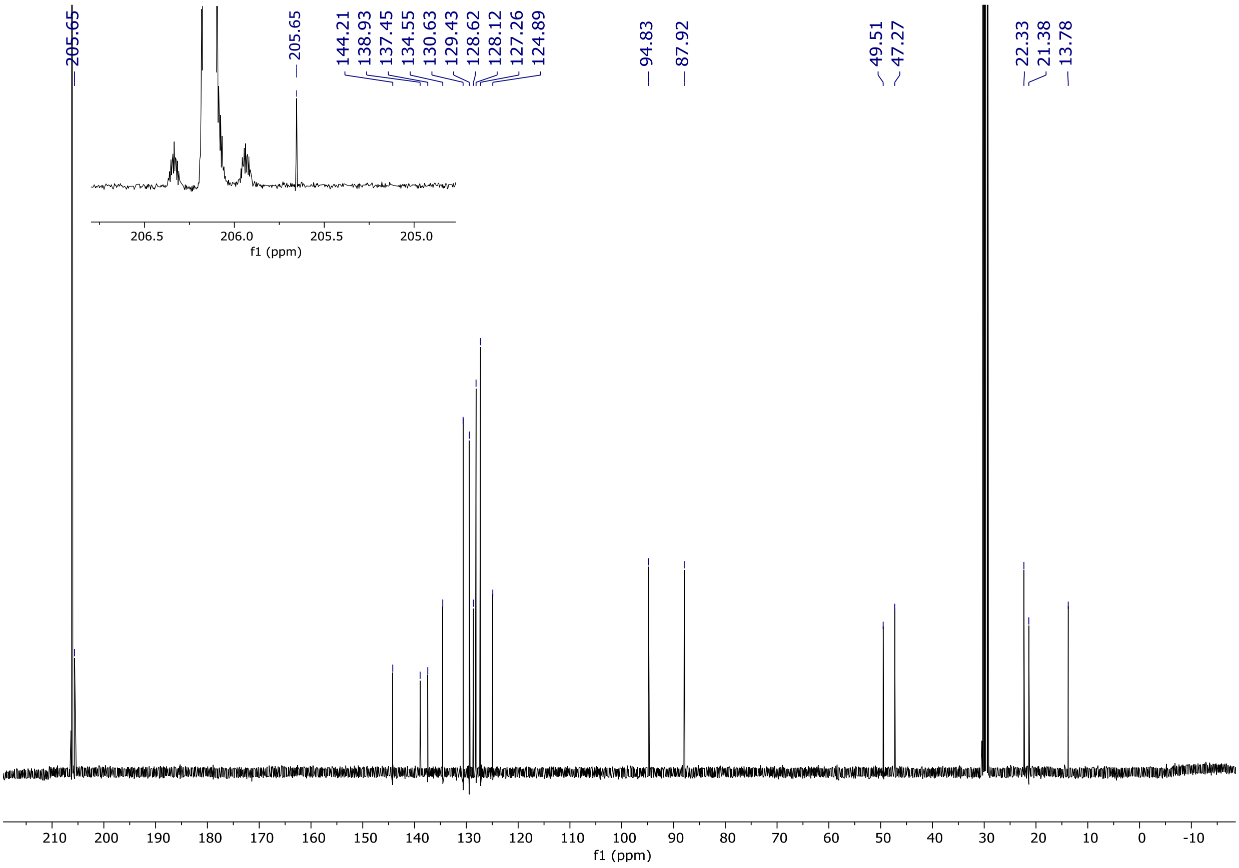

^{1}H NMR (400 MHz, $CDCl_3$)

1l

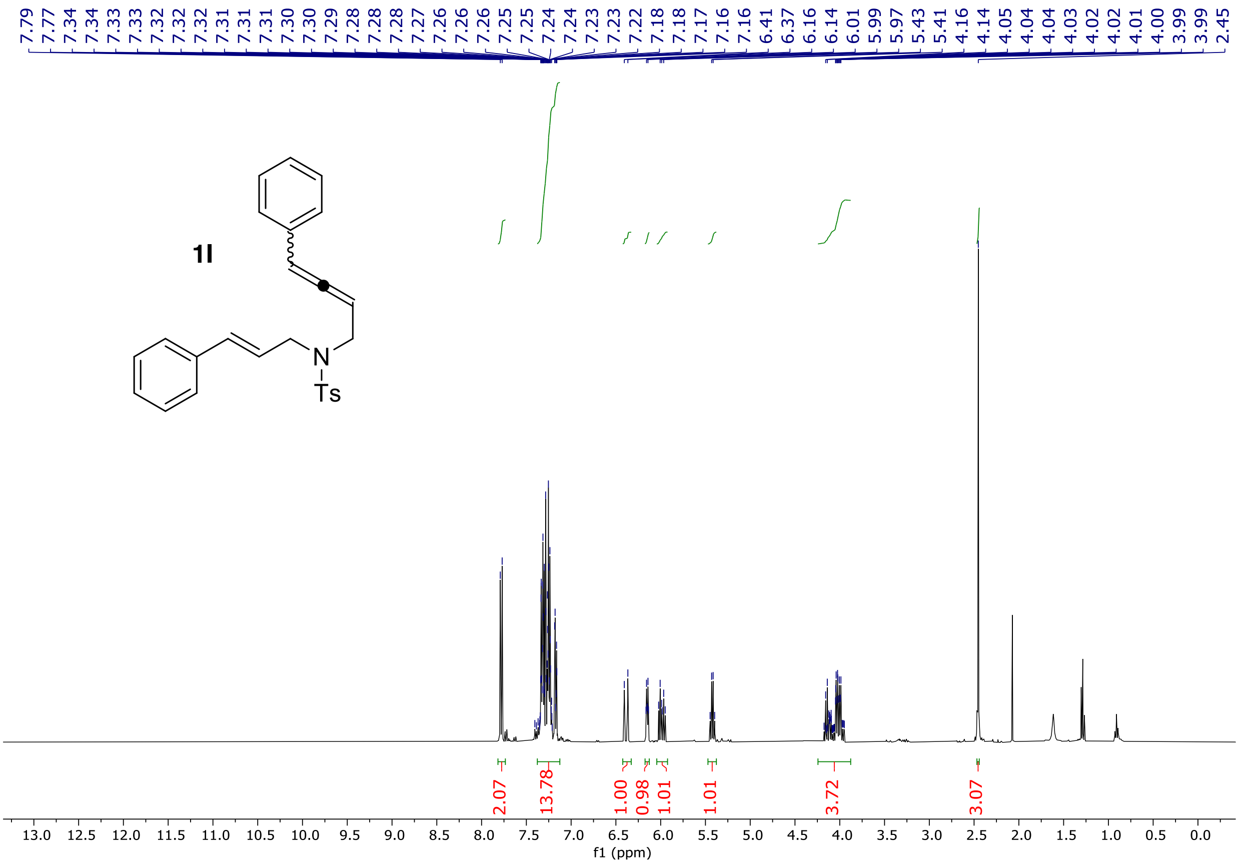

^{13}C NMR (101 MHz, $CDCl_3$)

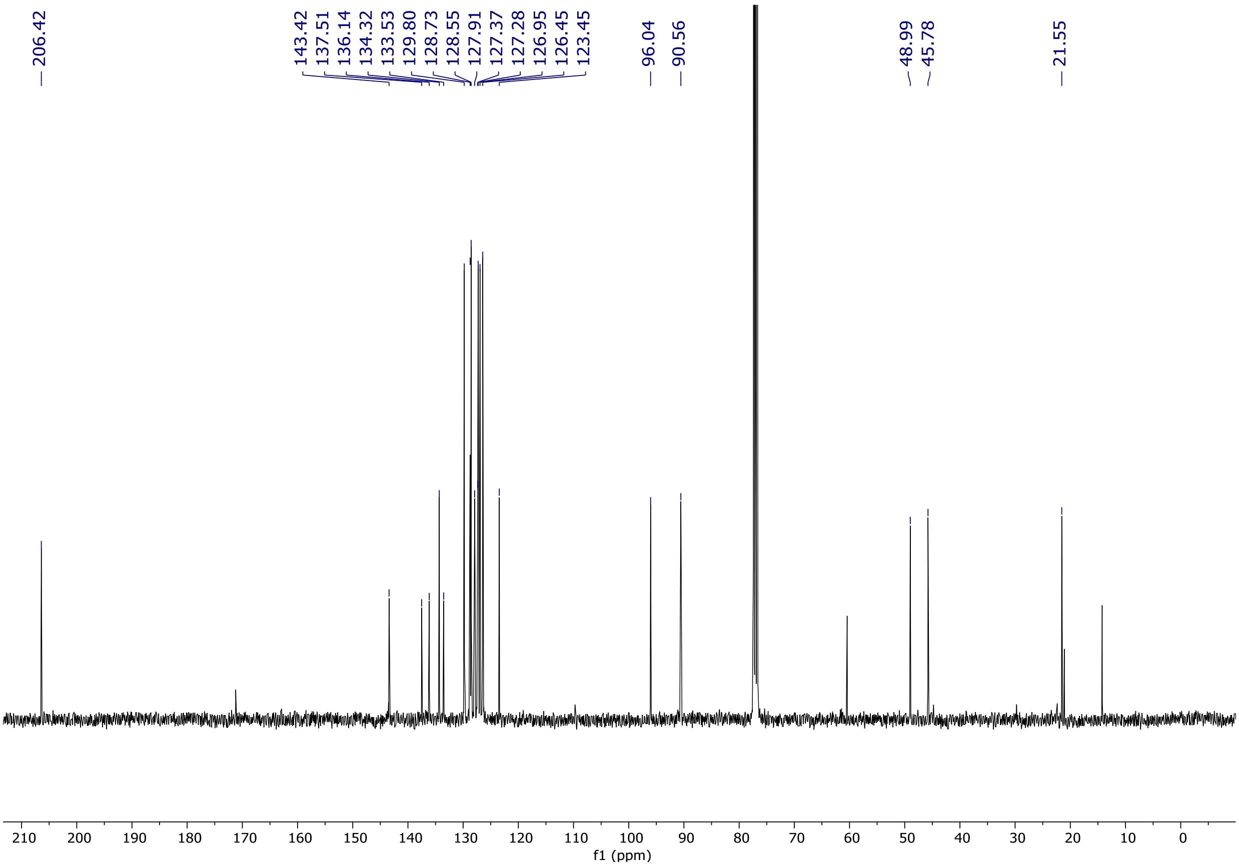

^{1}H NMR (400 MHz, $CDCl_3$)

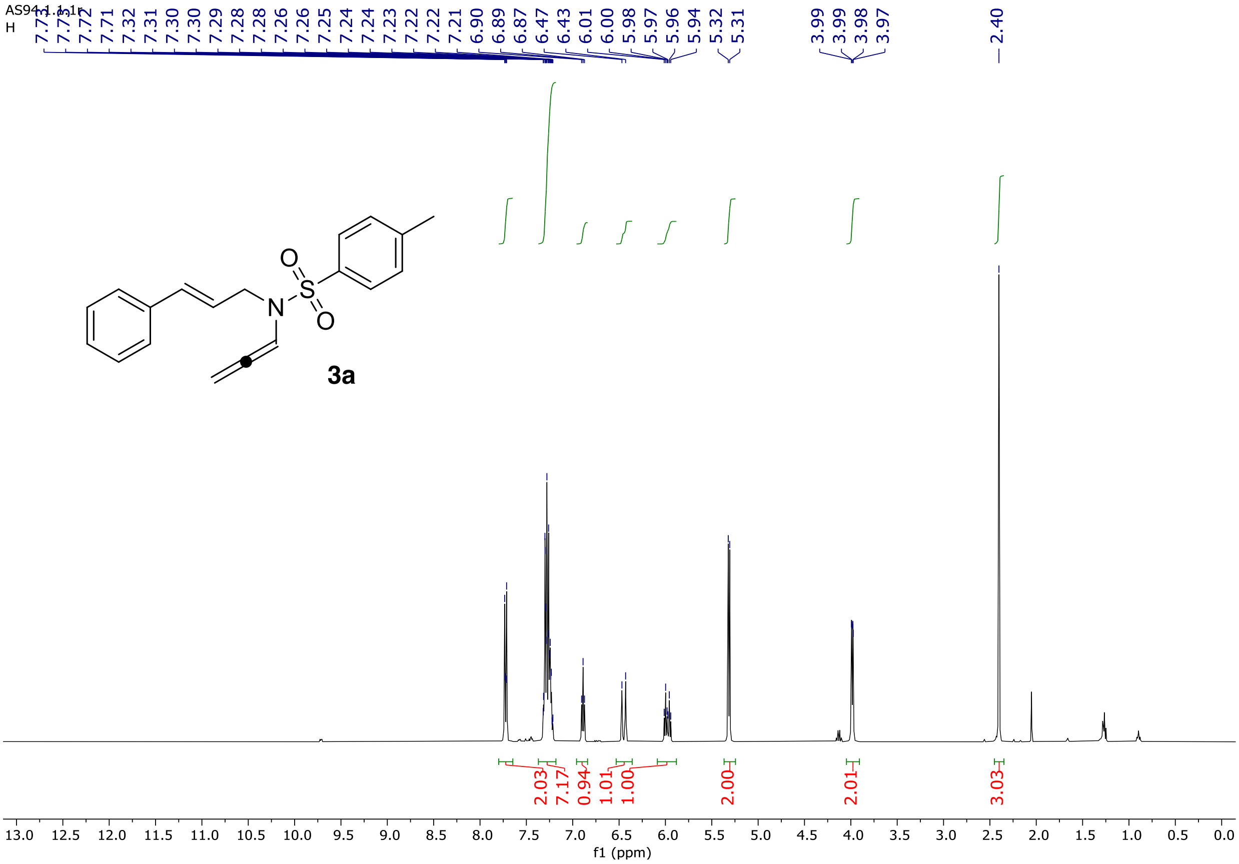

^{13}C NMR (101 MHz, $CDCl_3$)

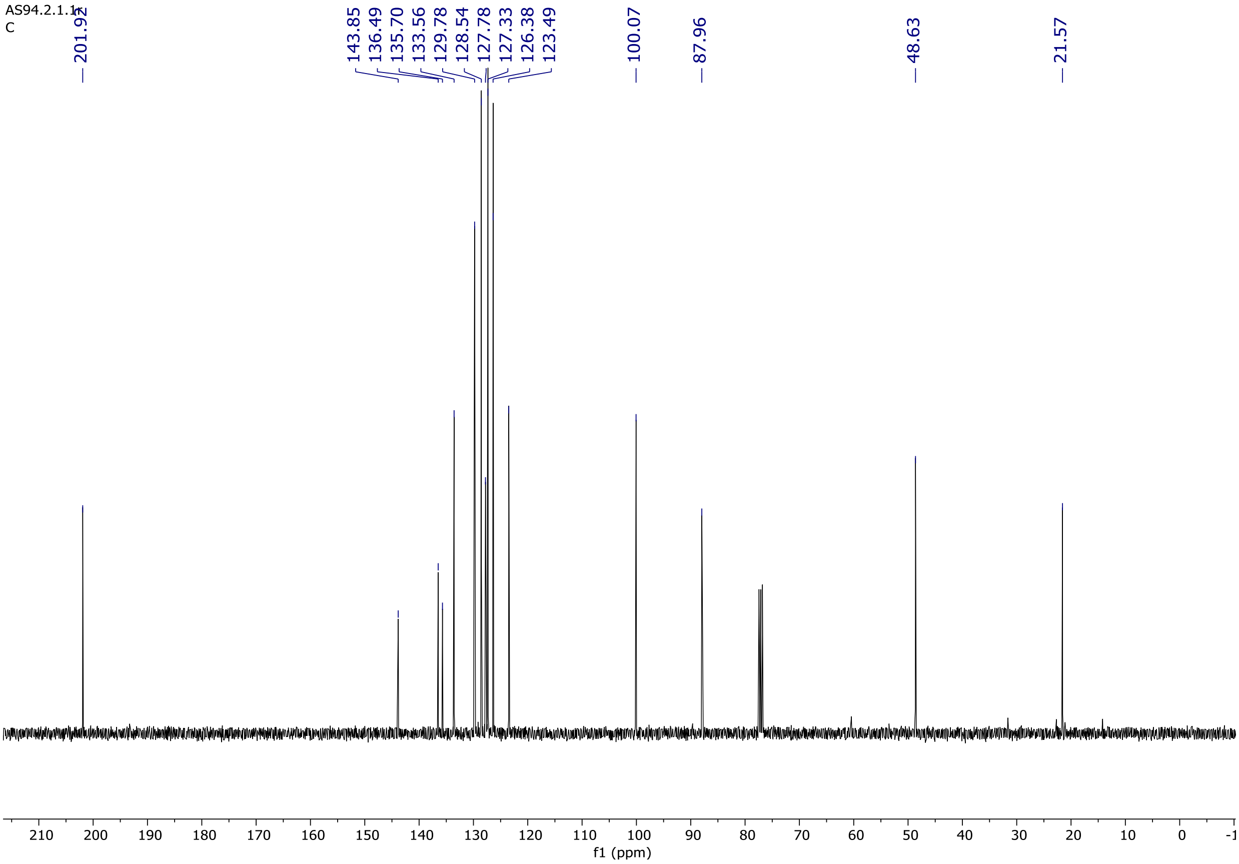

^{1}H NMR (400 MHz, acetone)

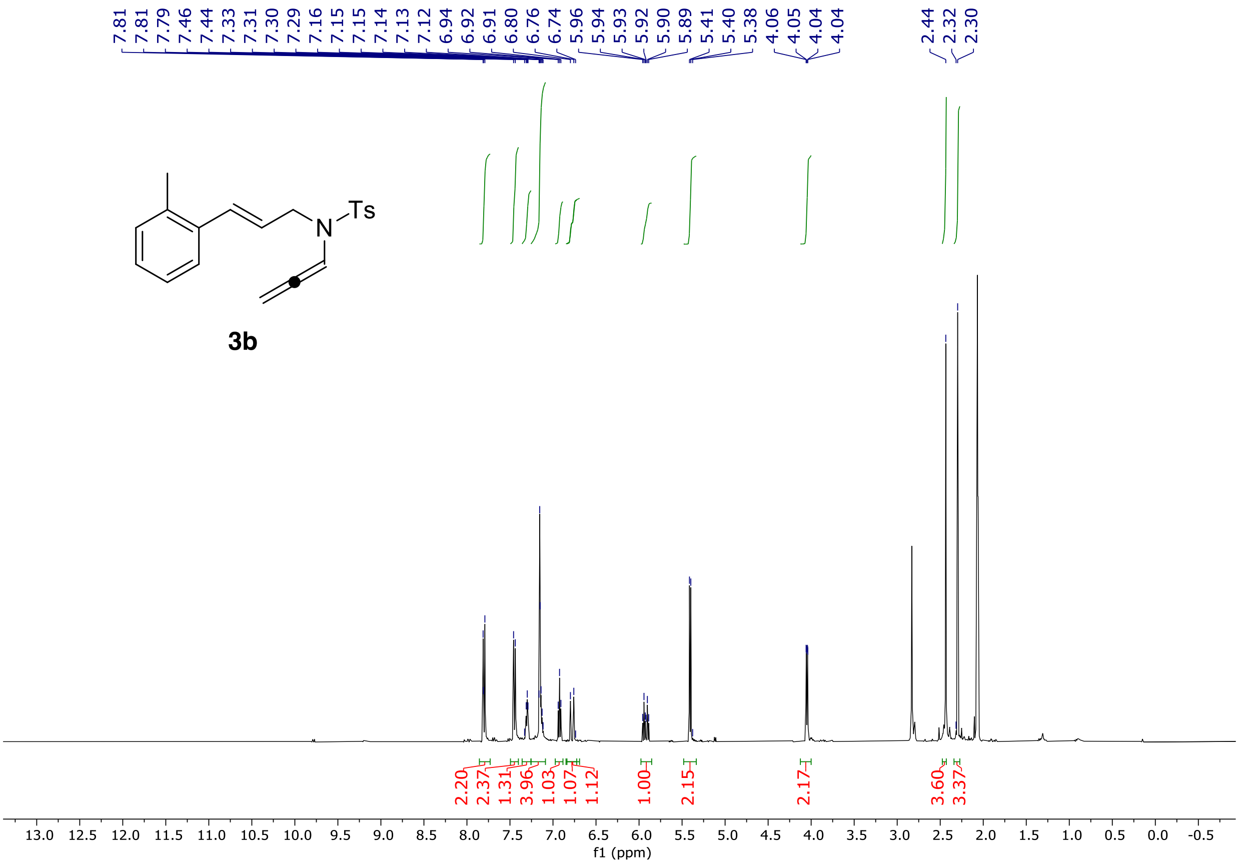

^{13}C NMR (101 MHz, acetone)

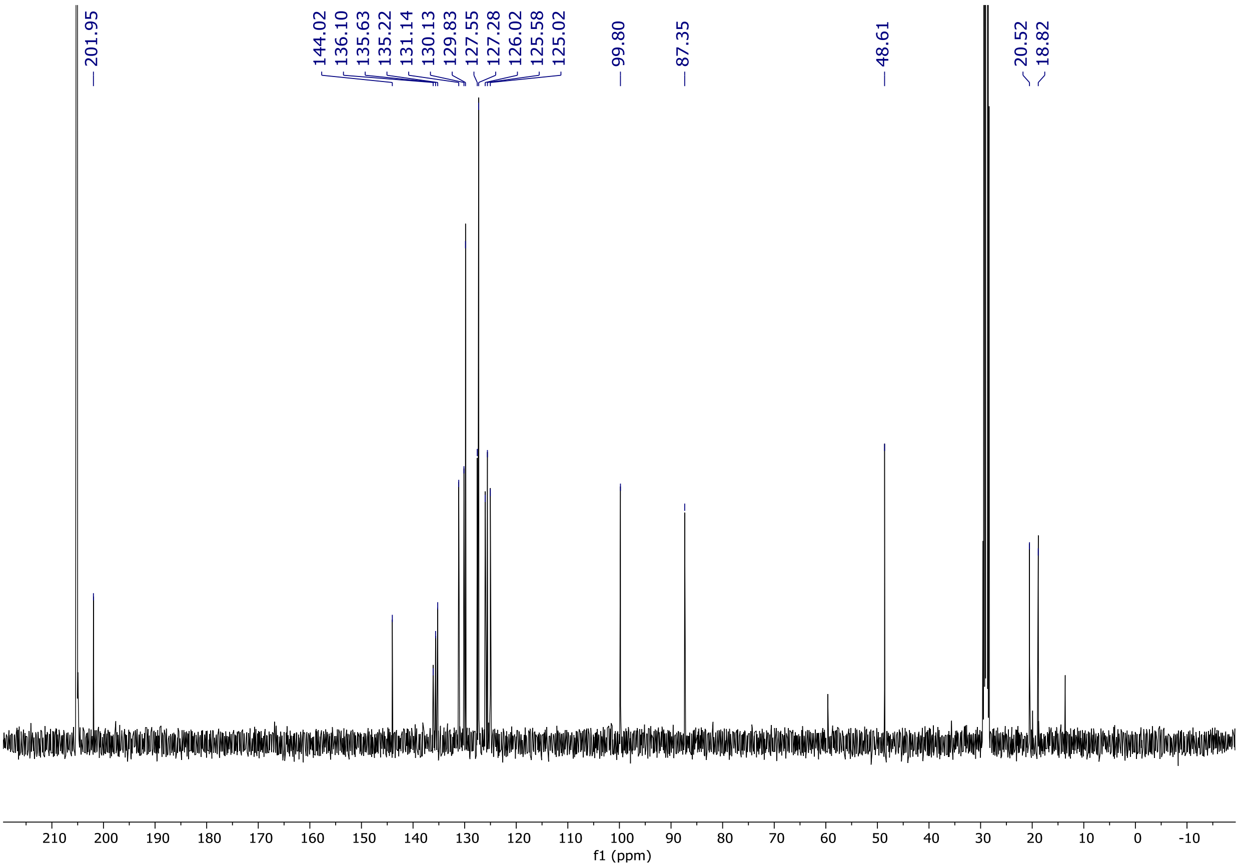

^{1}H NMR (400 MHz, acetone)

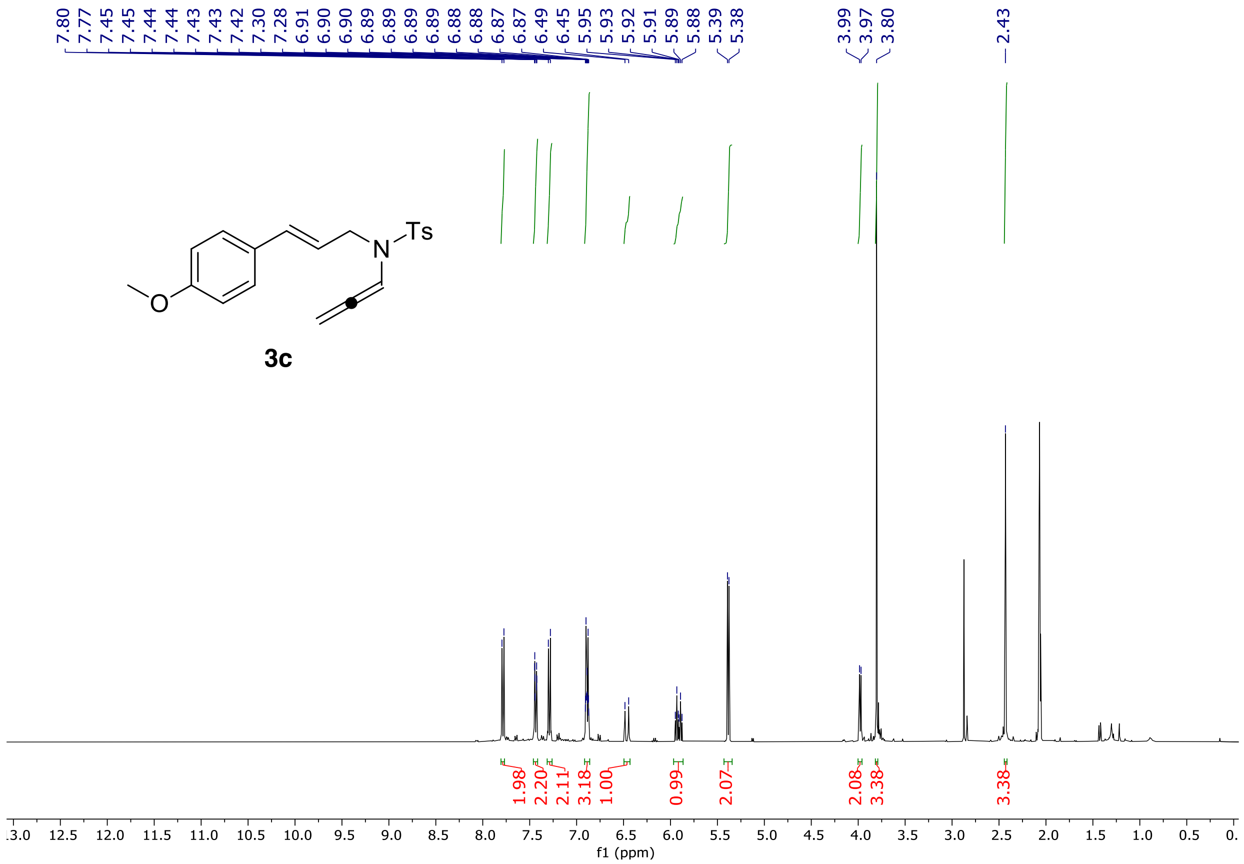

^{13}C NMR (101 MHz, acetone)

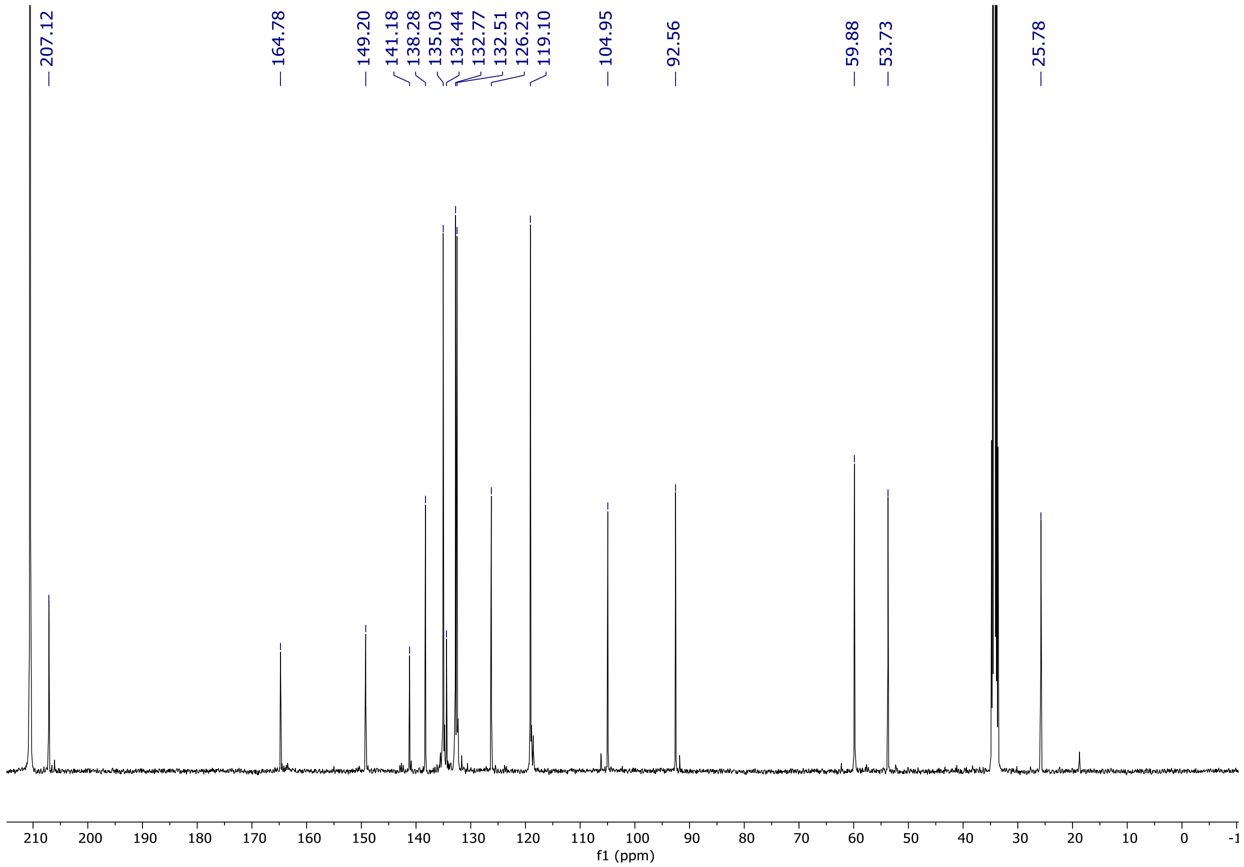

^{1}H NMR (400 MHz, $CDCl_3$)

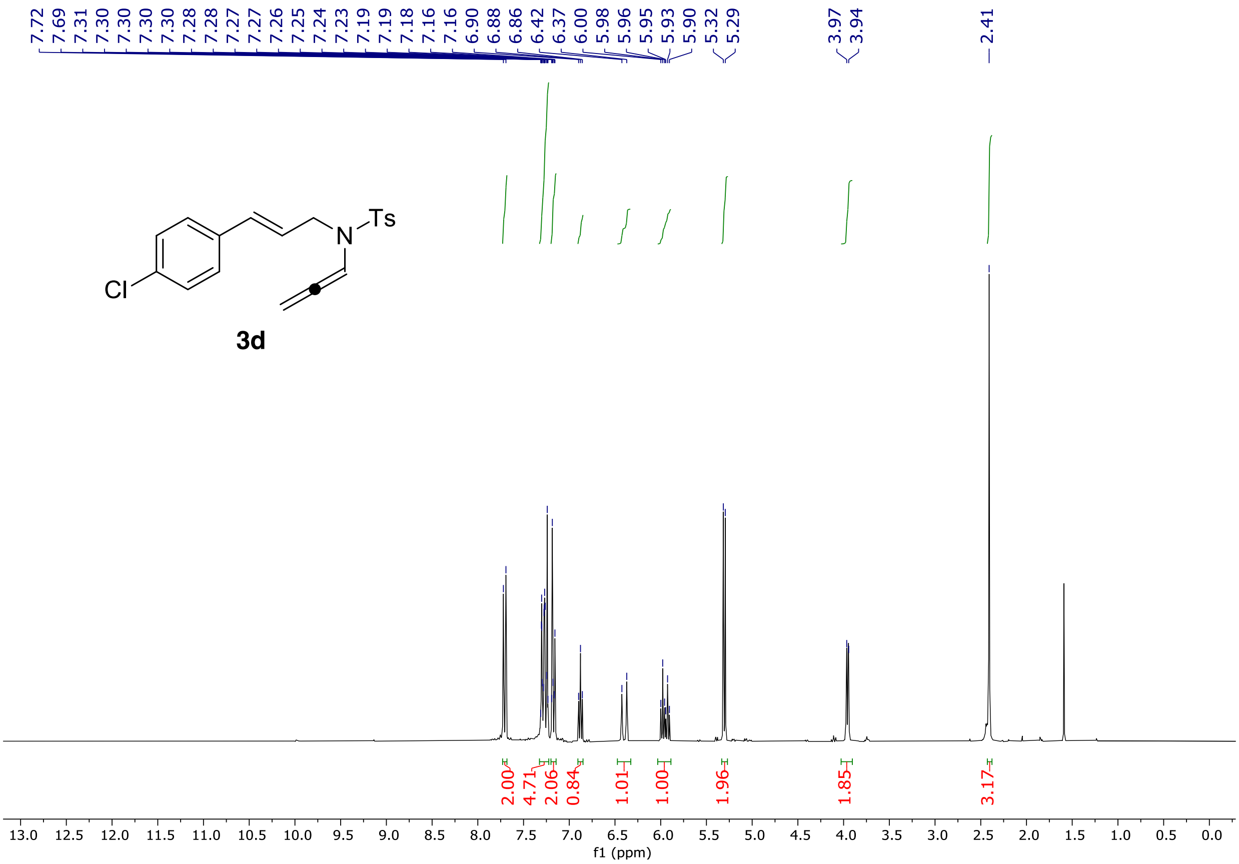

3d

^{13}C NMR (101 MHz, $CDCl_3$)

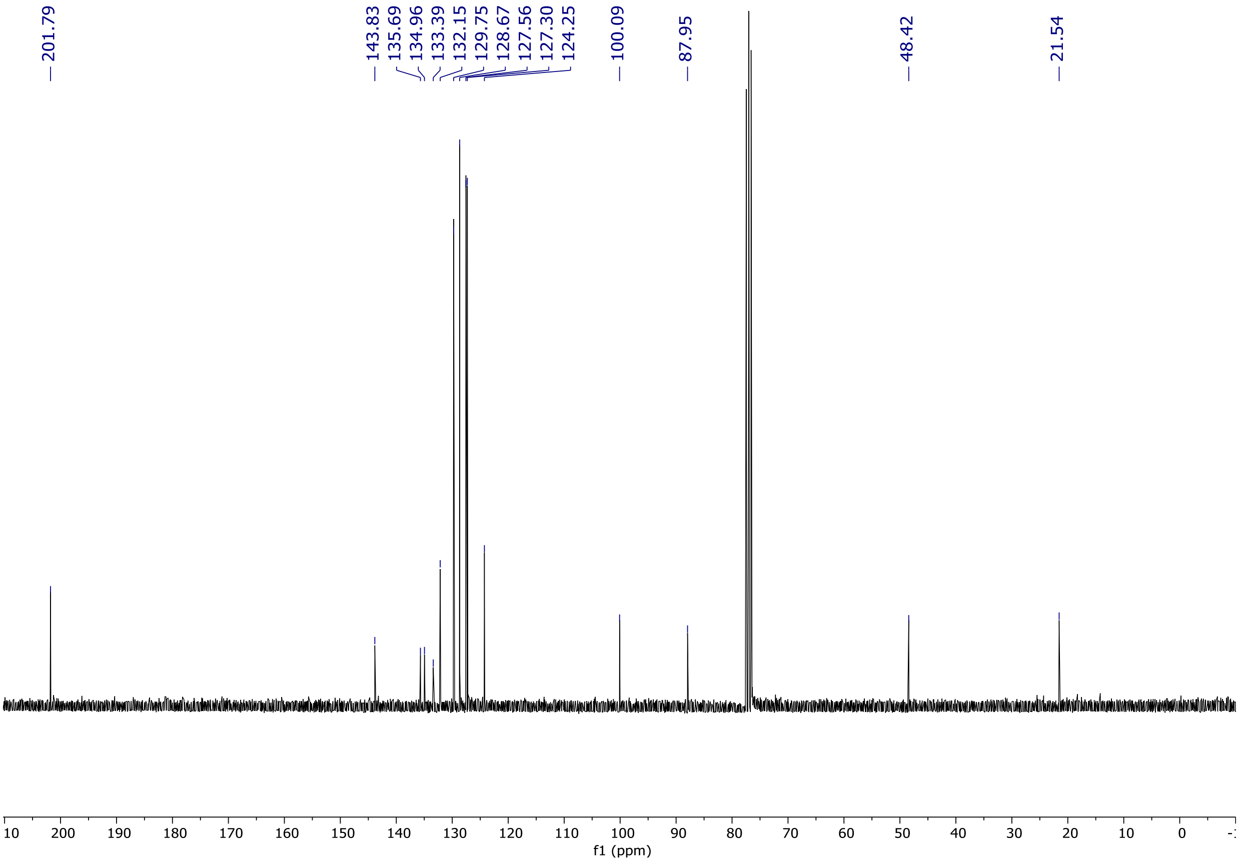

^{1}H NMR (400 MHz, acetone)

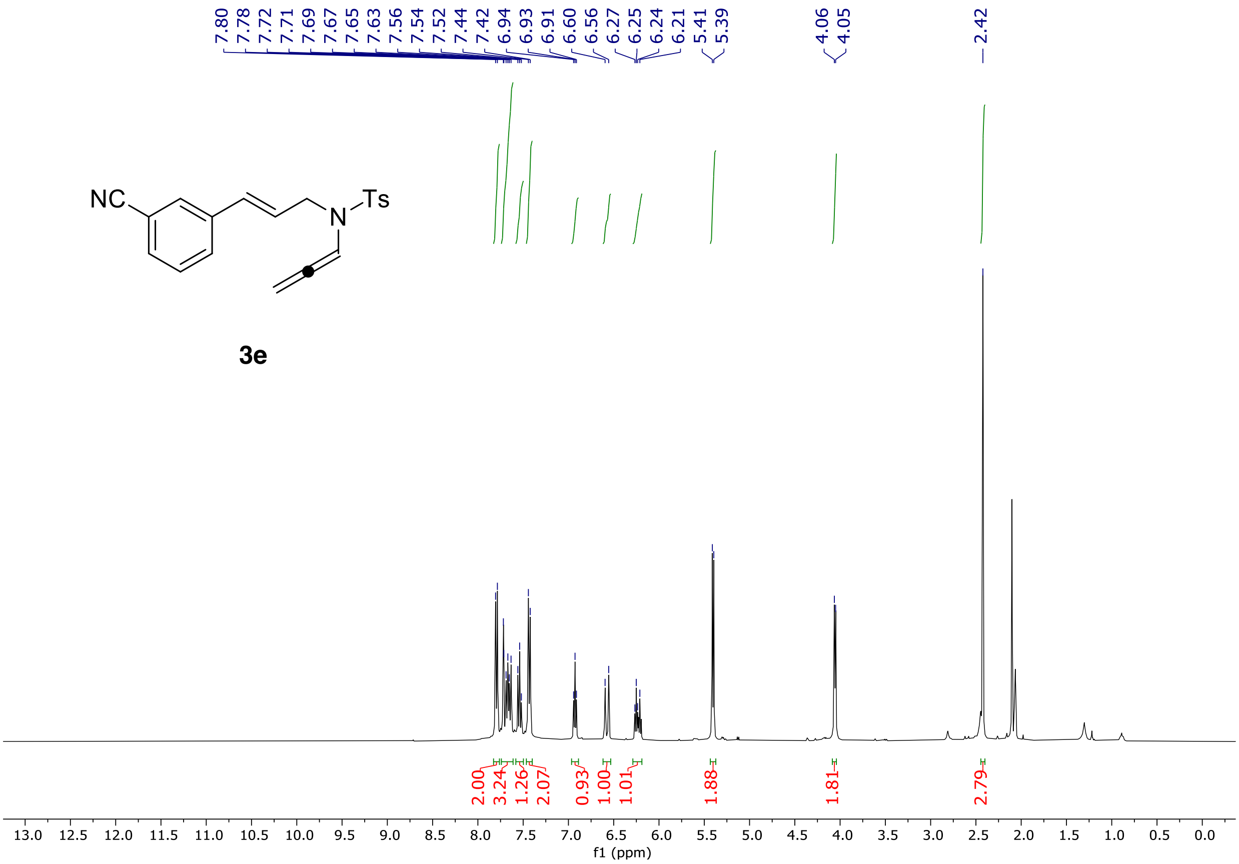

^{13}C NMR (101 MHz, acetone)

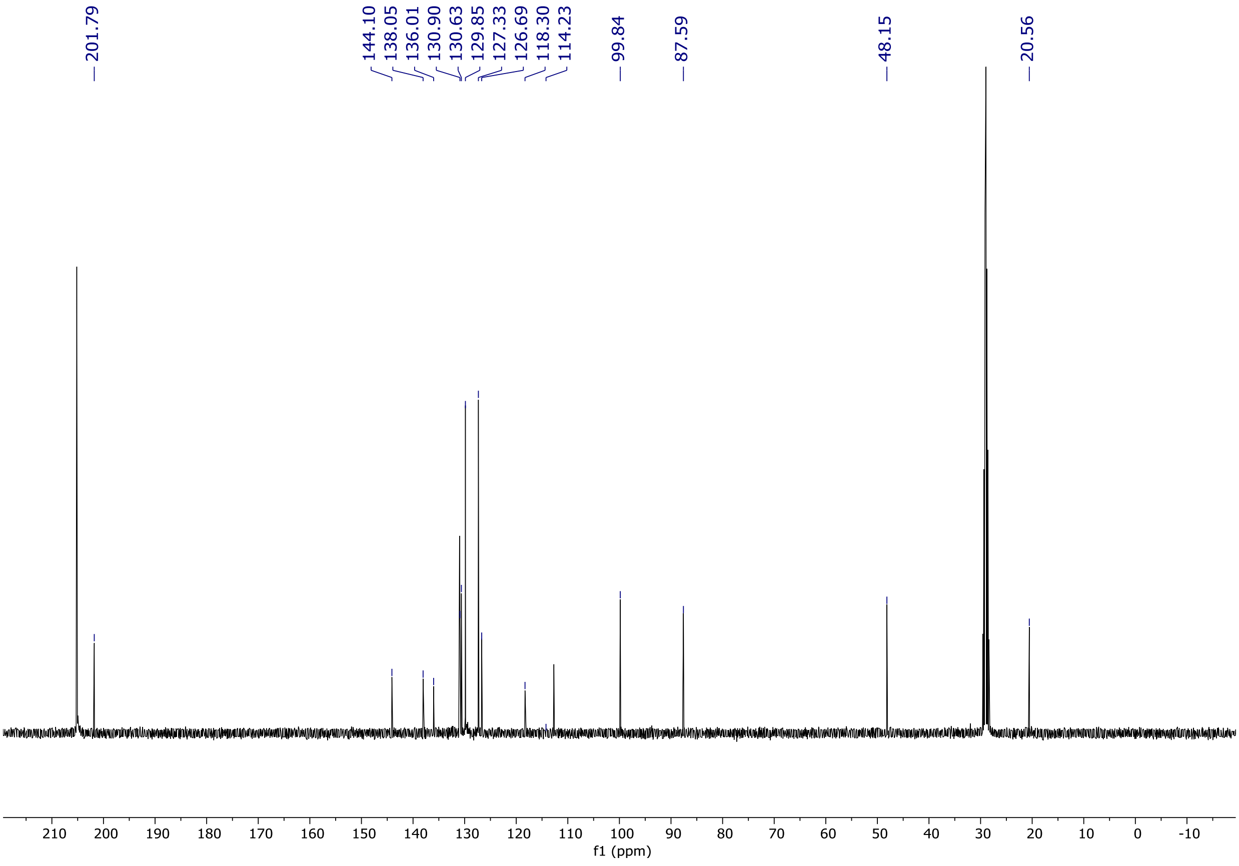

^{1}H NMR (400 MHz, $CDCl_3$)

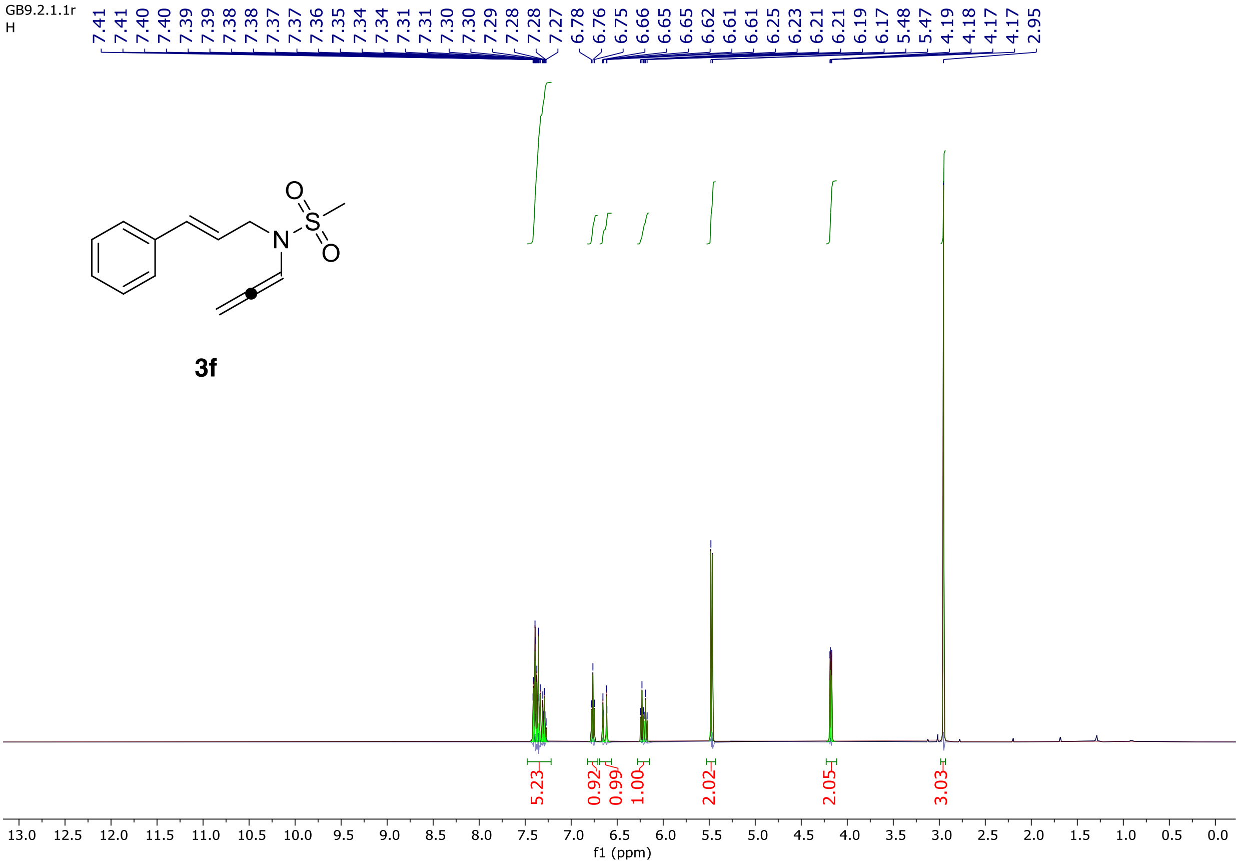

3f

^{13}C NMR (101 MHz, $CDCl_3$)

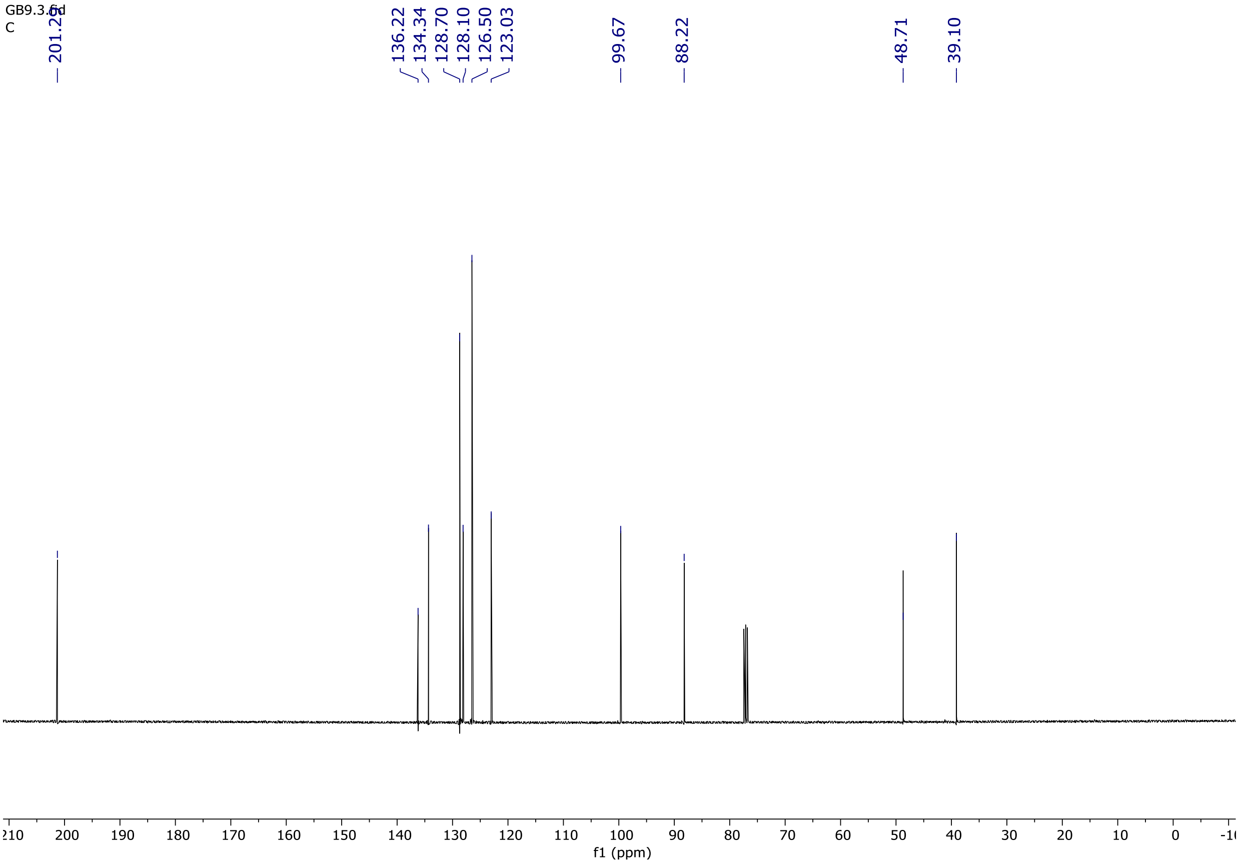

^{1}H NMR (400 MHz, acetone)

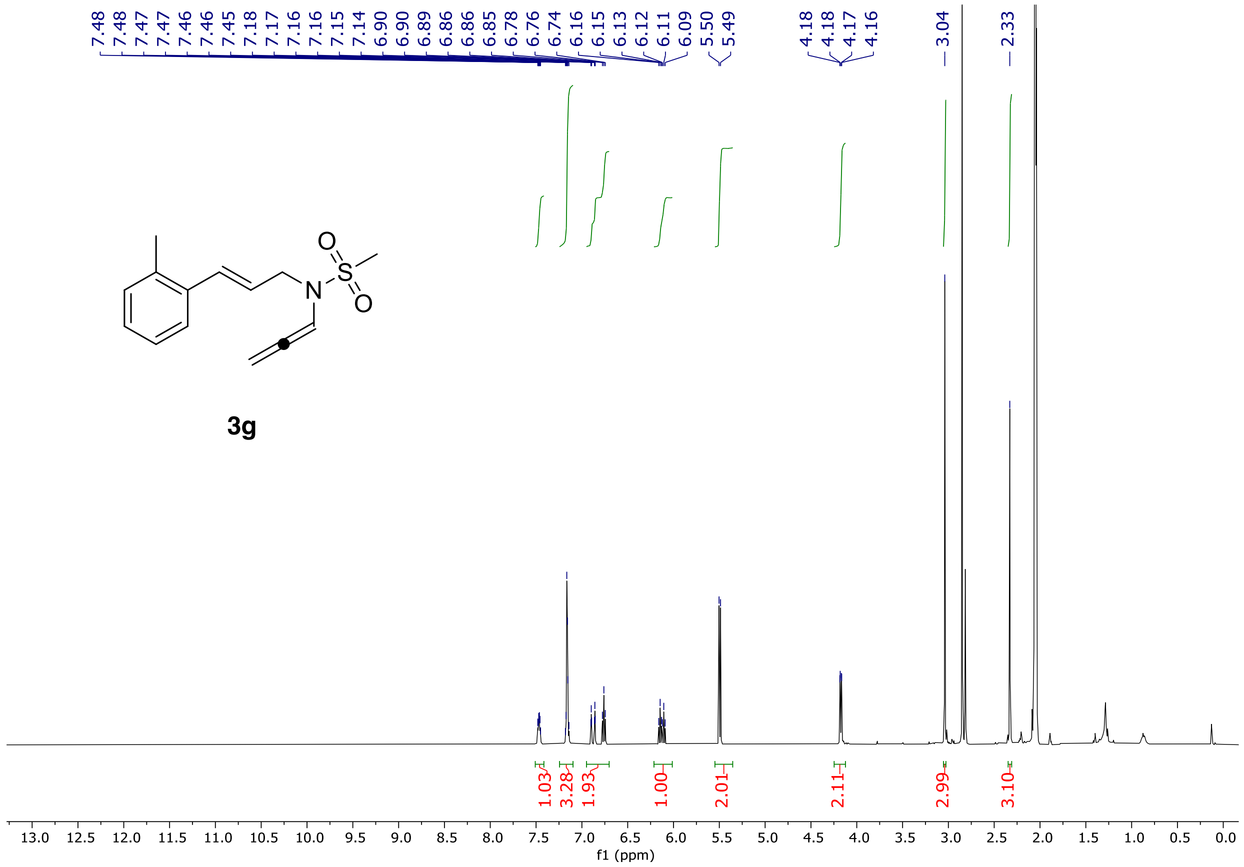

^{13}C NMR (101 MHz, acetone)

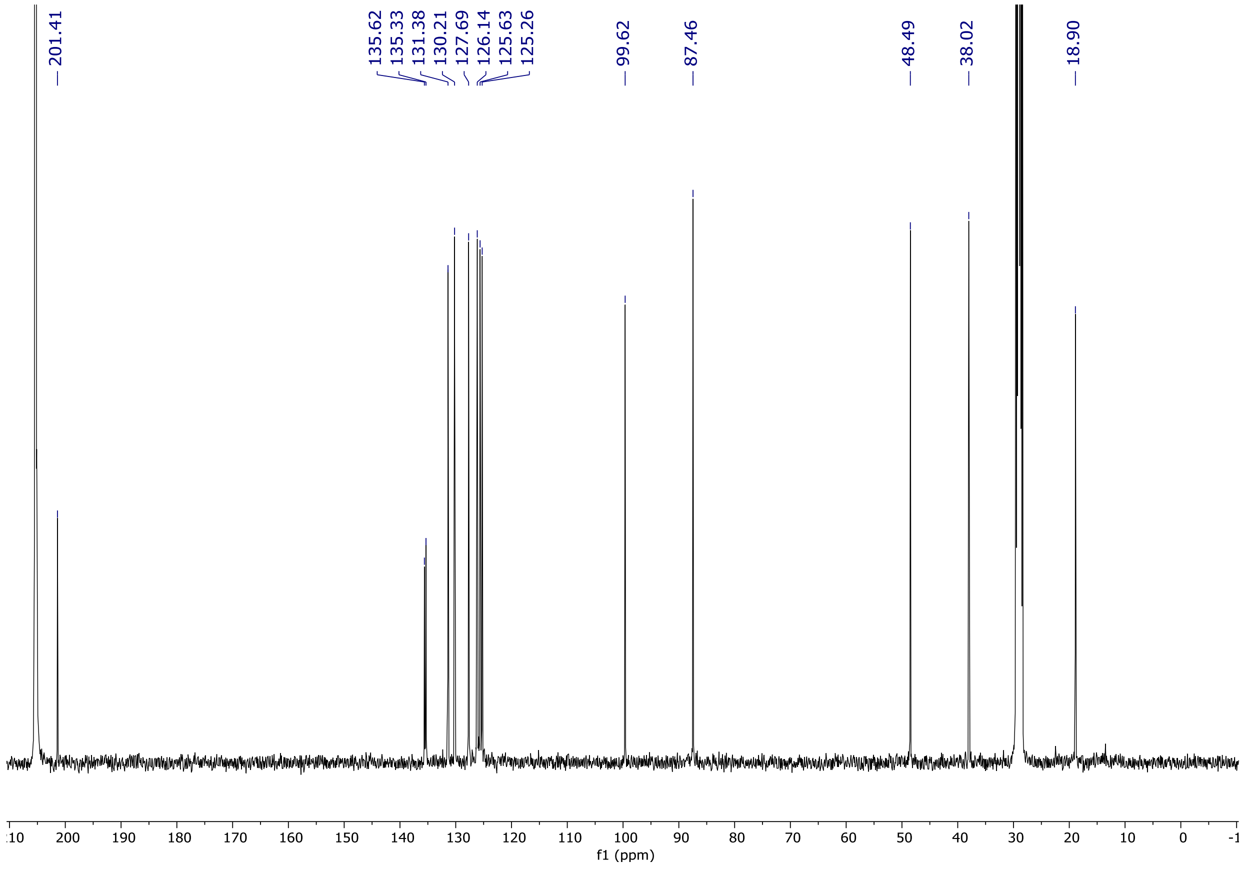

^{1}H NMR (400 MHz, acetone)

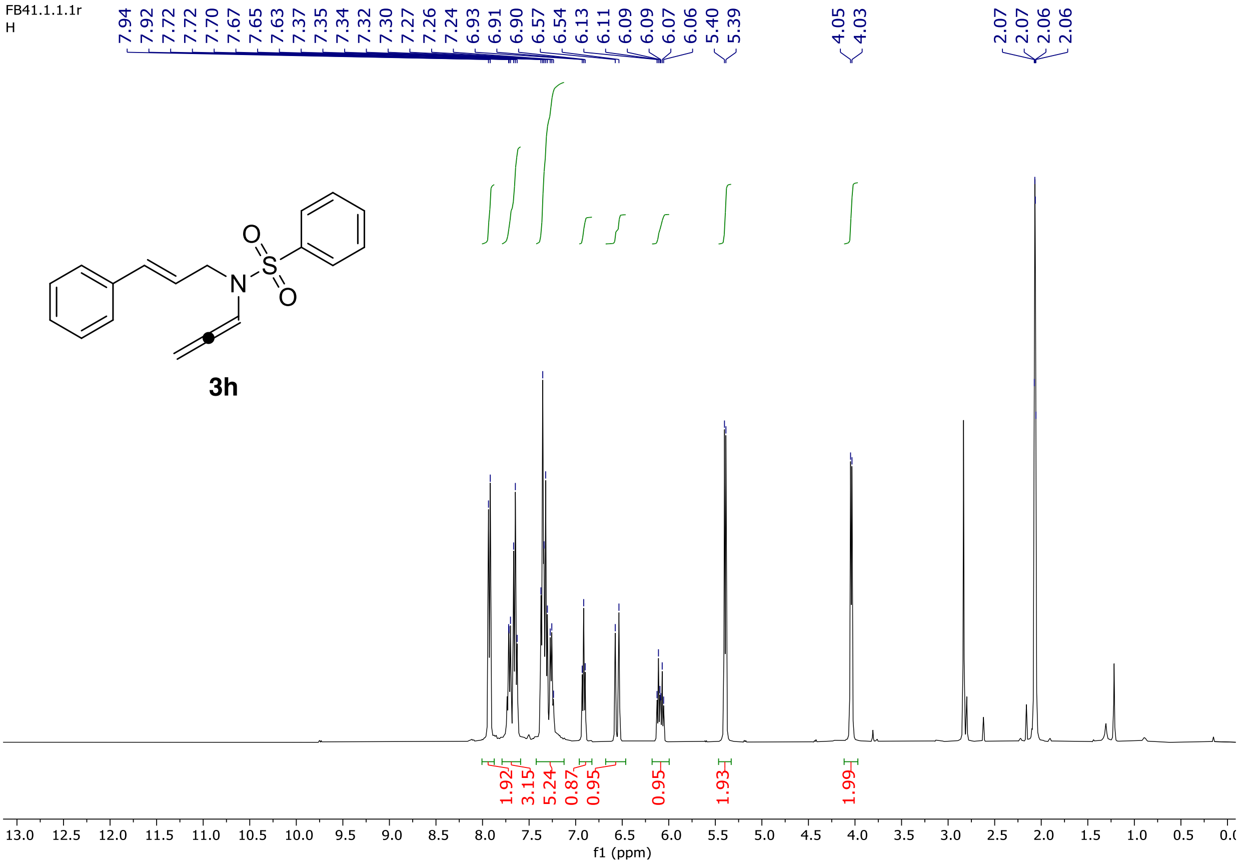

^{13}C NMR (101 MHz, acetone)

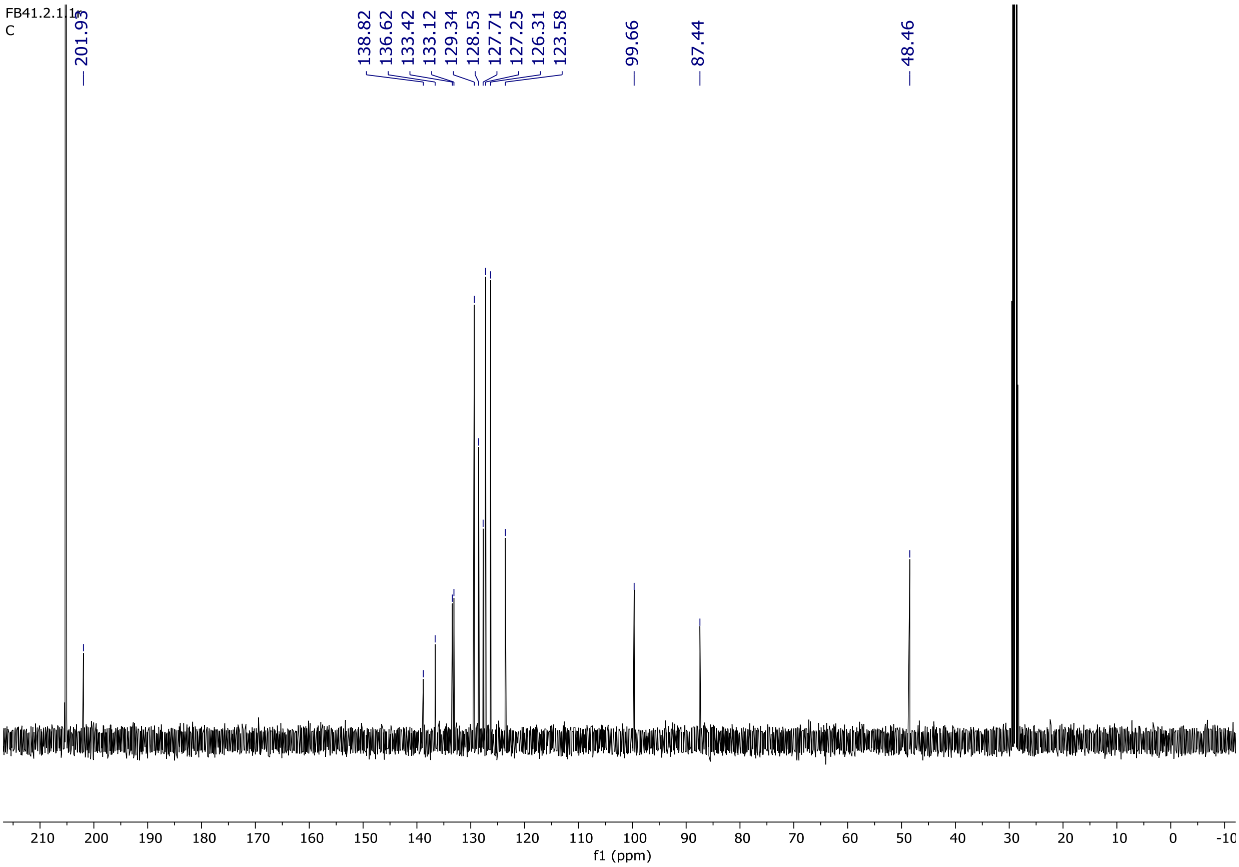

^{1}H NMR (300 MHz, acetone)

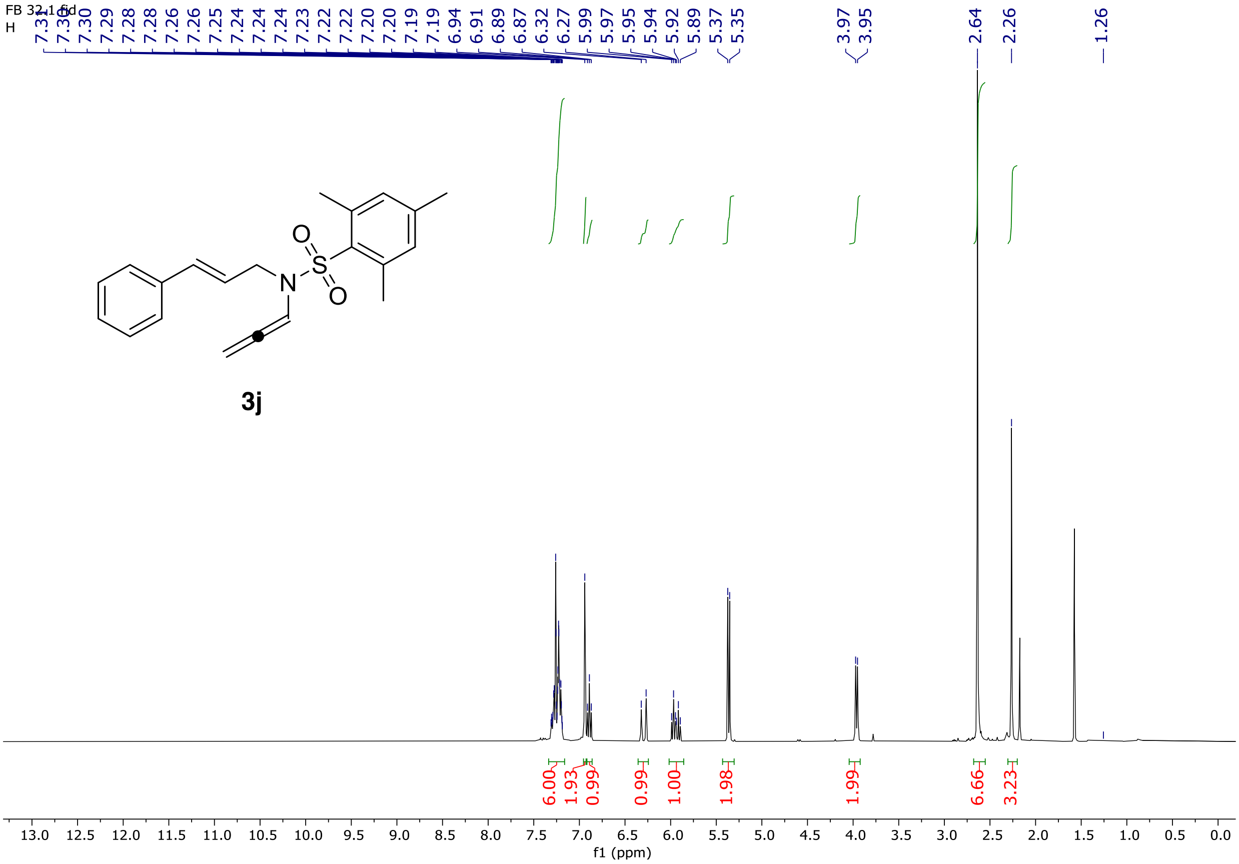

^{13}C NMR (75 MHz, acetone)

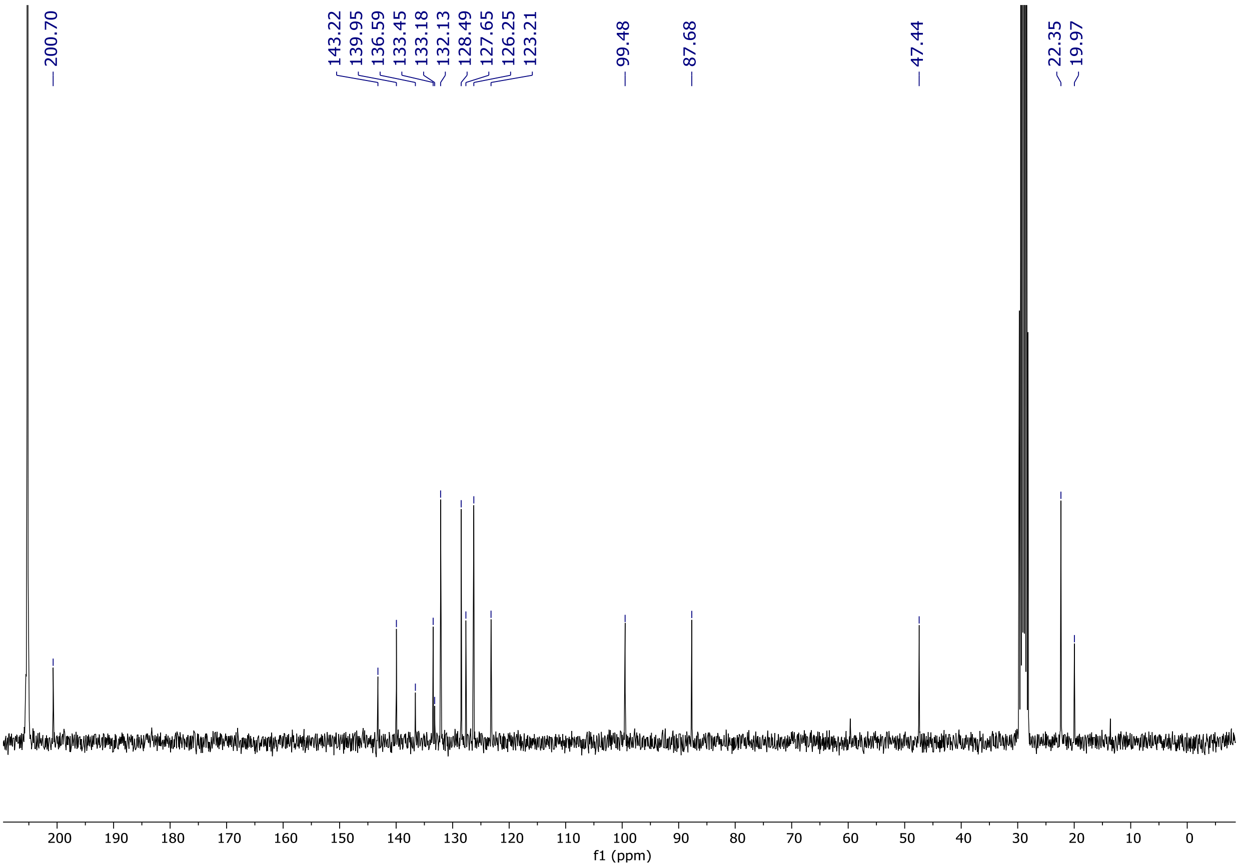

^{1}H NMR (300 MHz, $CDCl_3$)

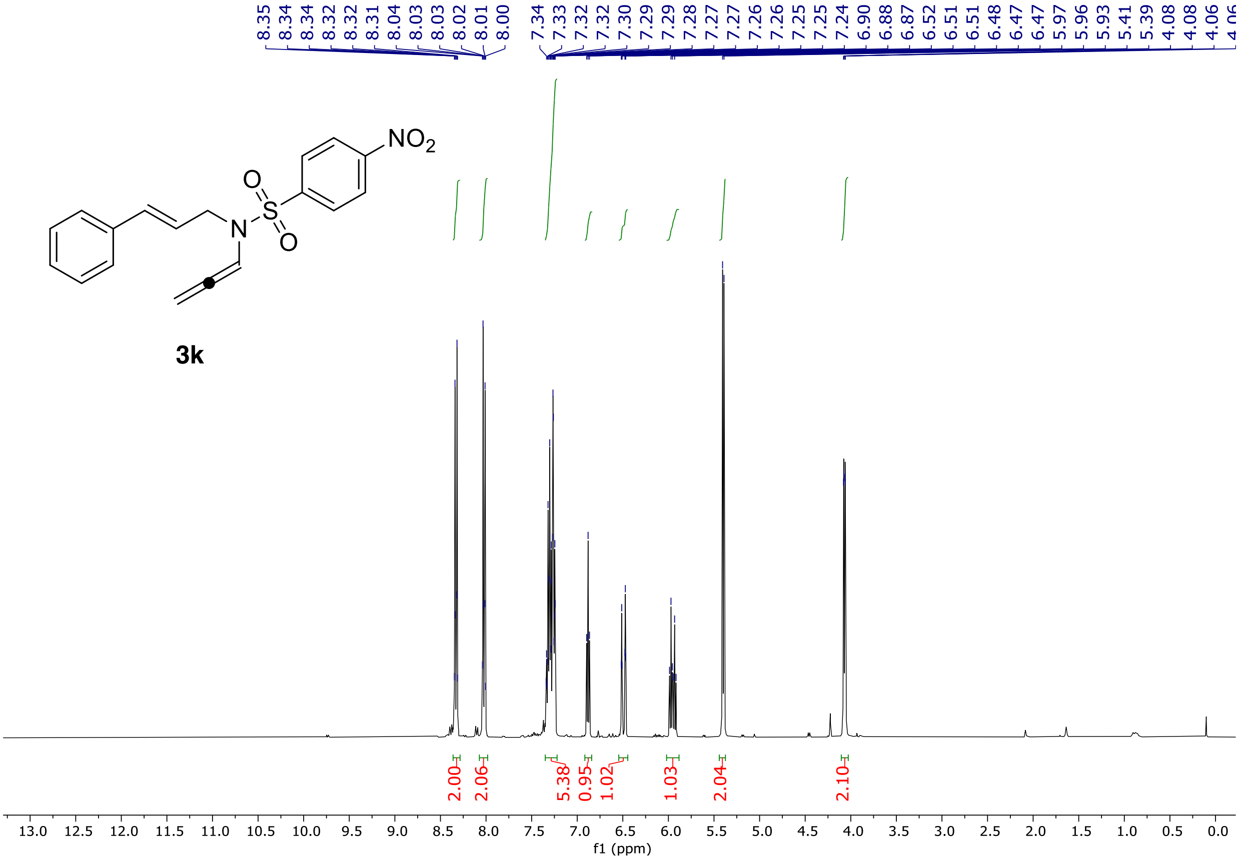

^{13}C NMR (75 MHz, $CDCl_3$)

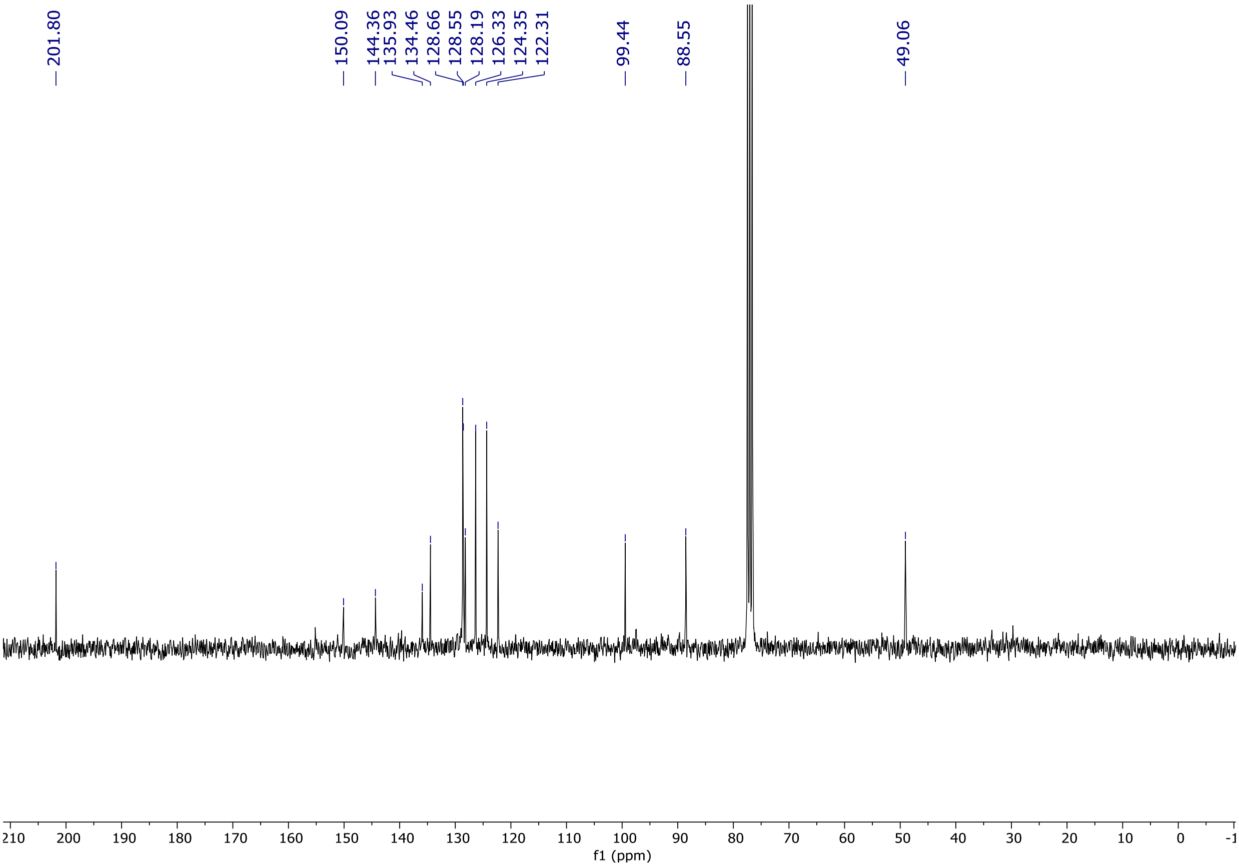

^{1}H NMR (300 MHz, acetone)

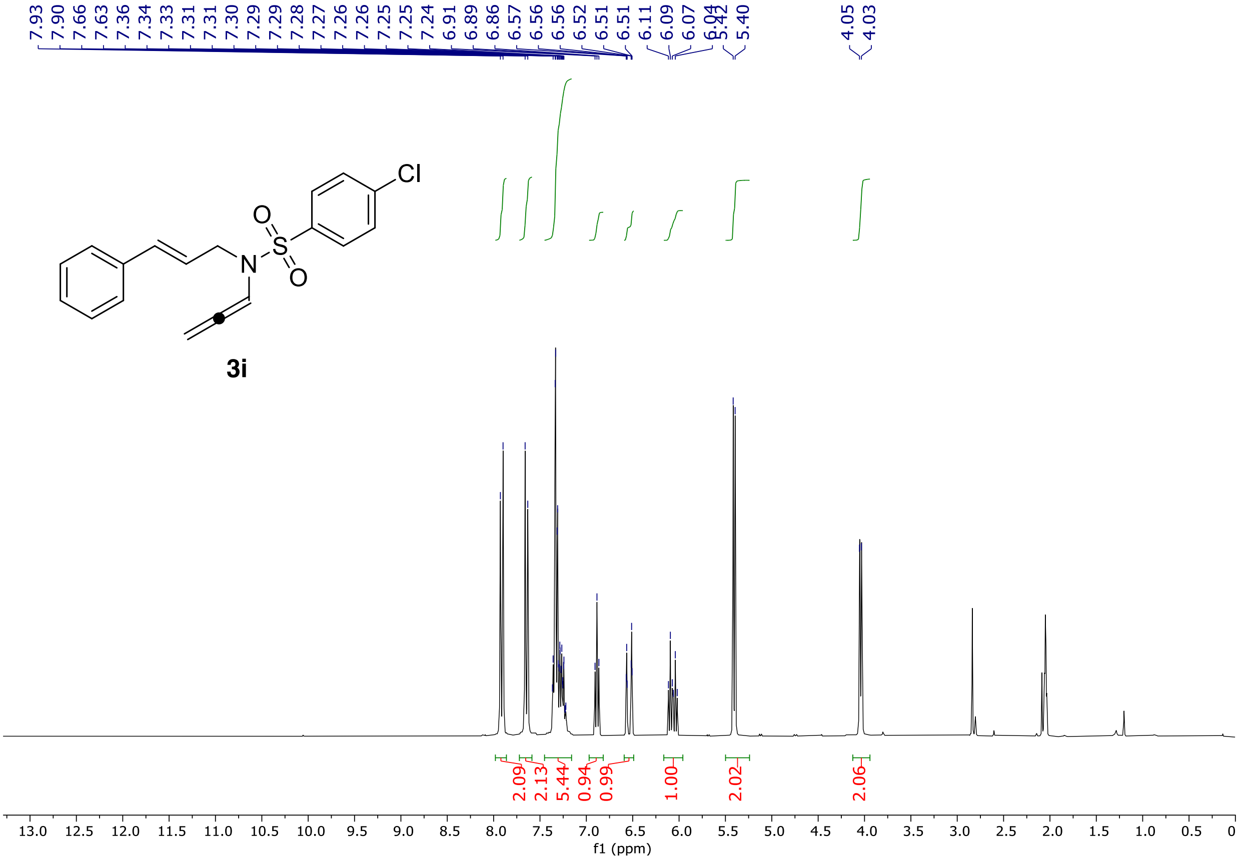

^{13}C NMR (75 MHz, acetone)

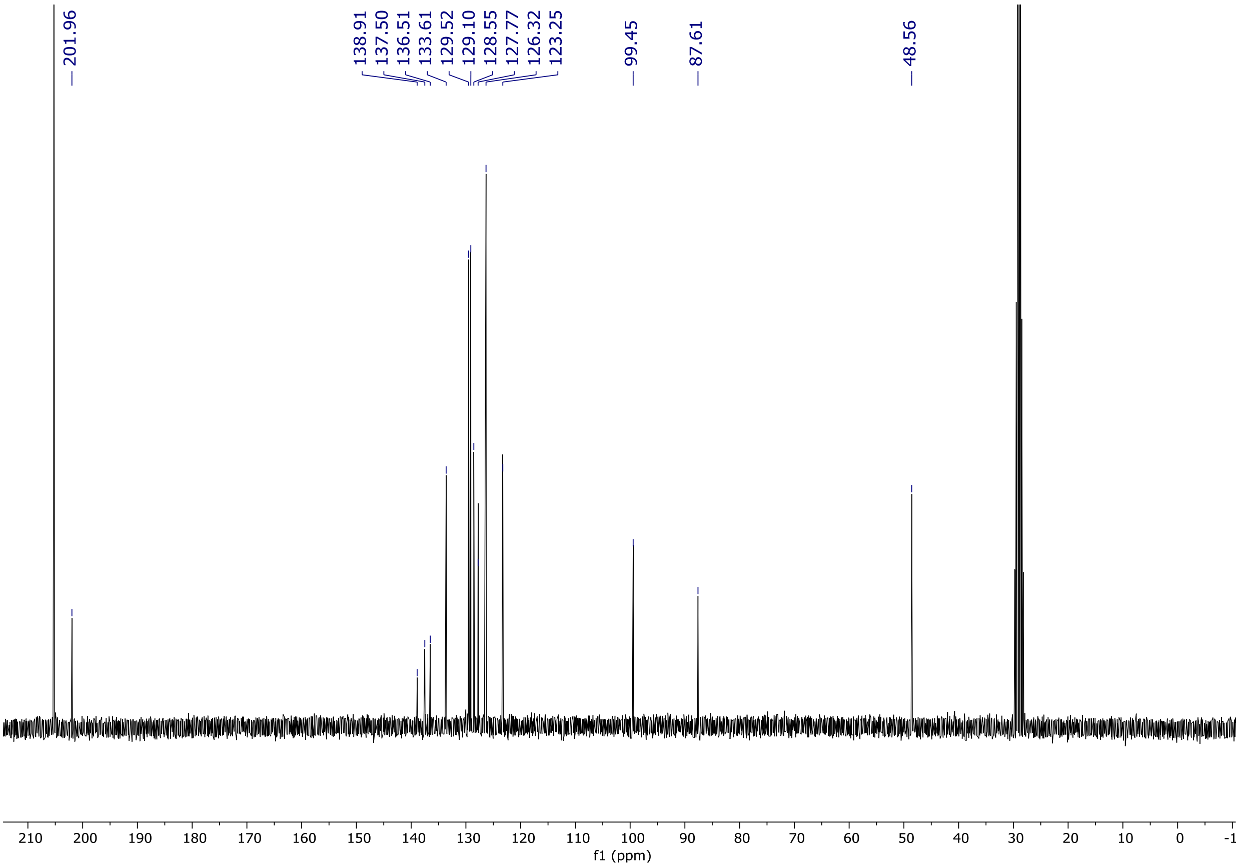

^{1}H NMR (300 MHz, acetone)

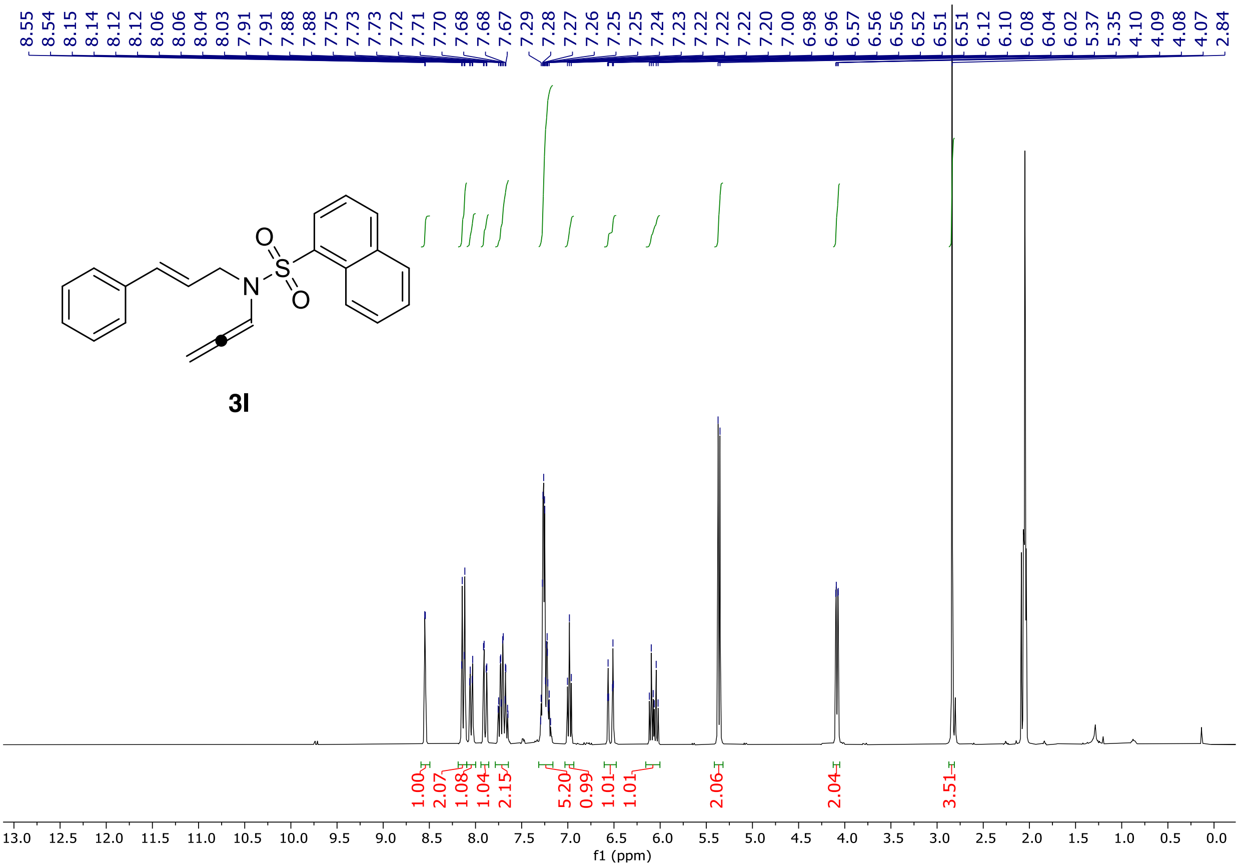

^{13}C NMR (75 MHz, acetone)

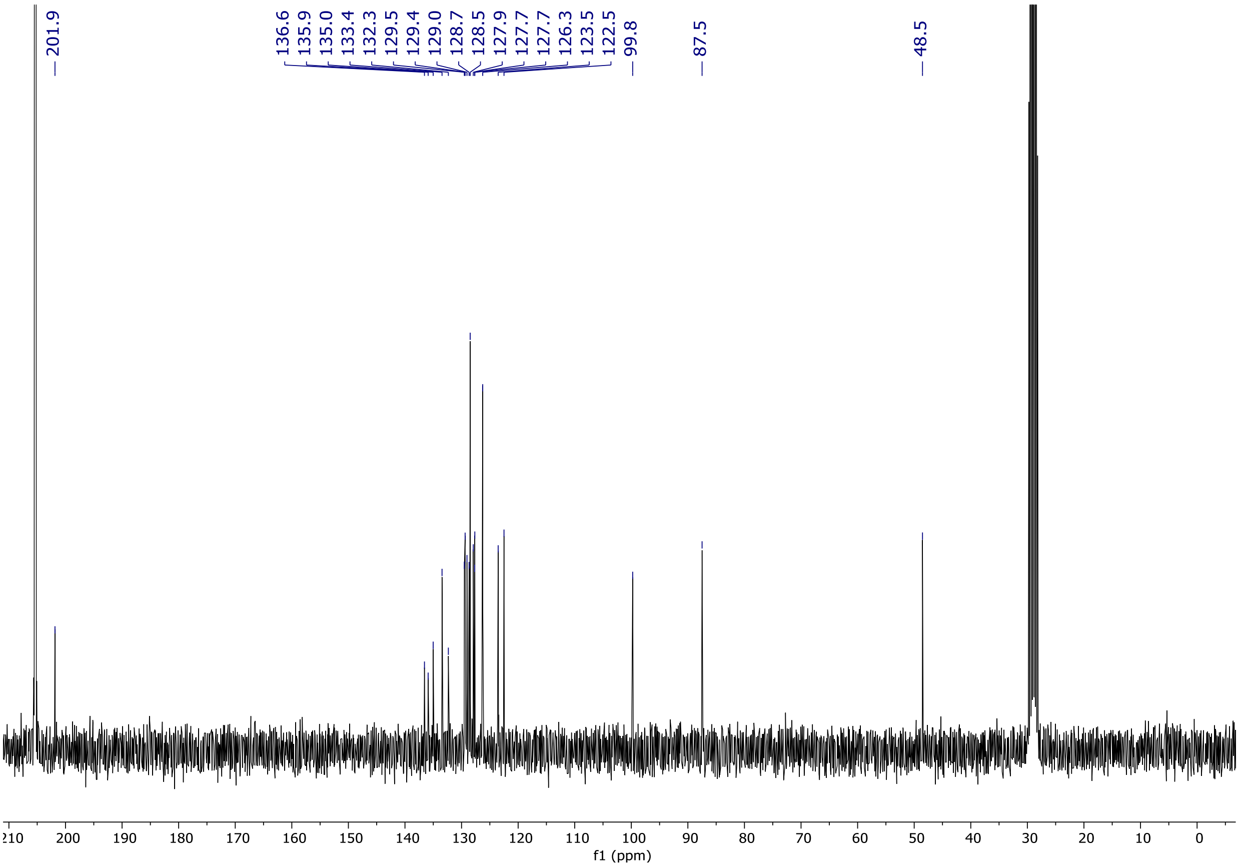

^{1}H NMR (400 MHz, acetone)

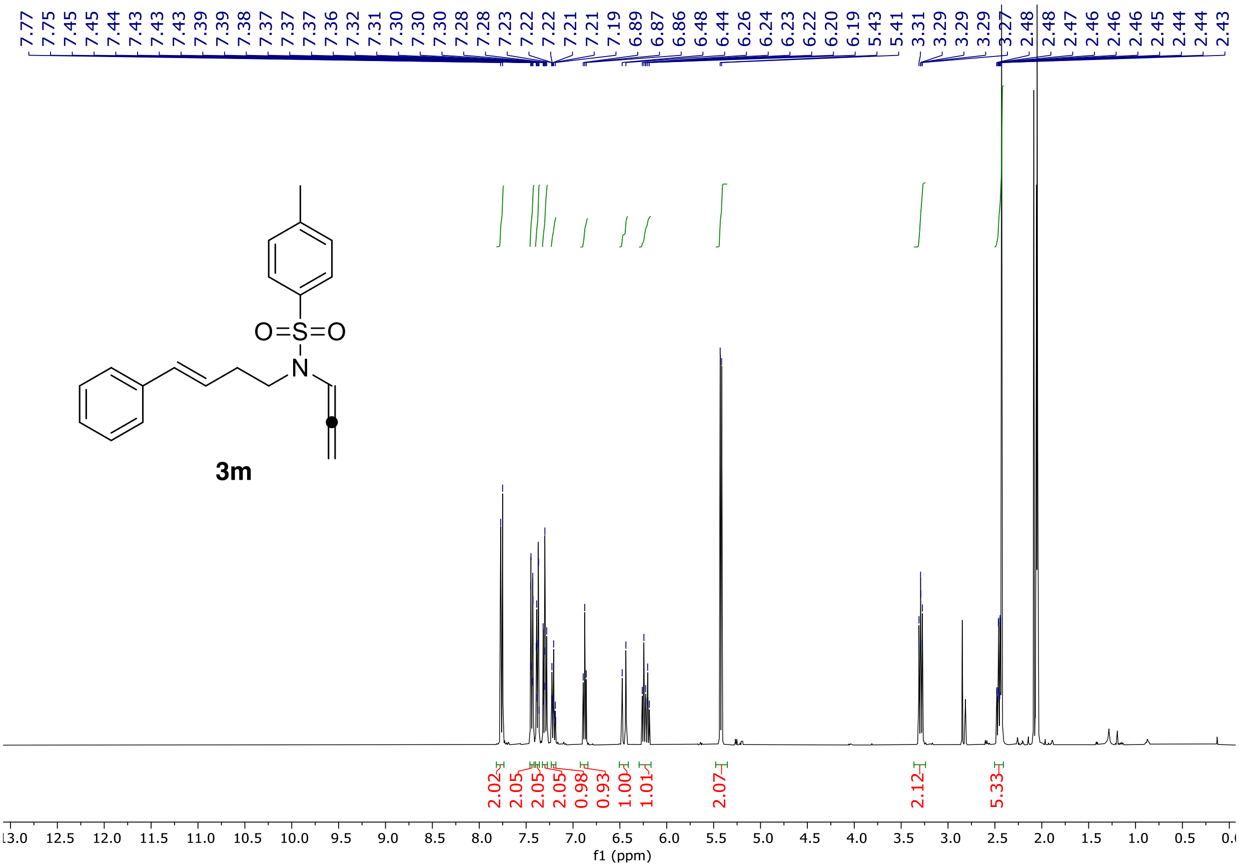

^{13}C NMR (101 MHz, acetone)

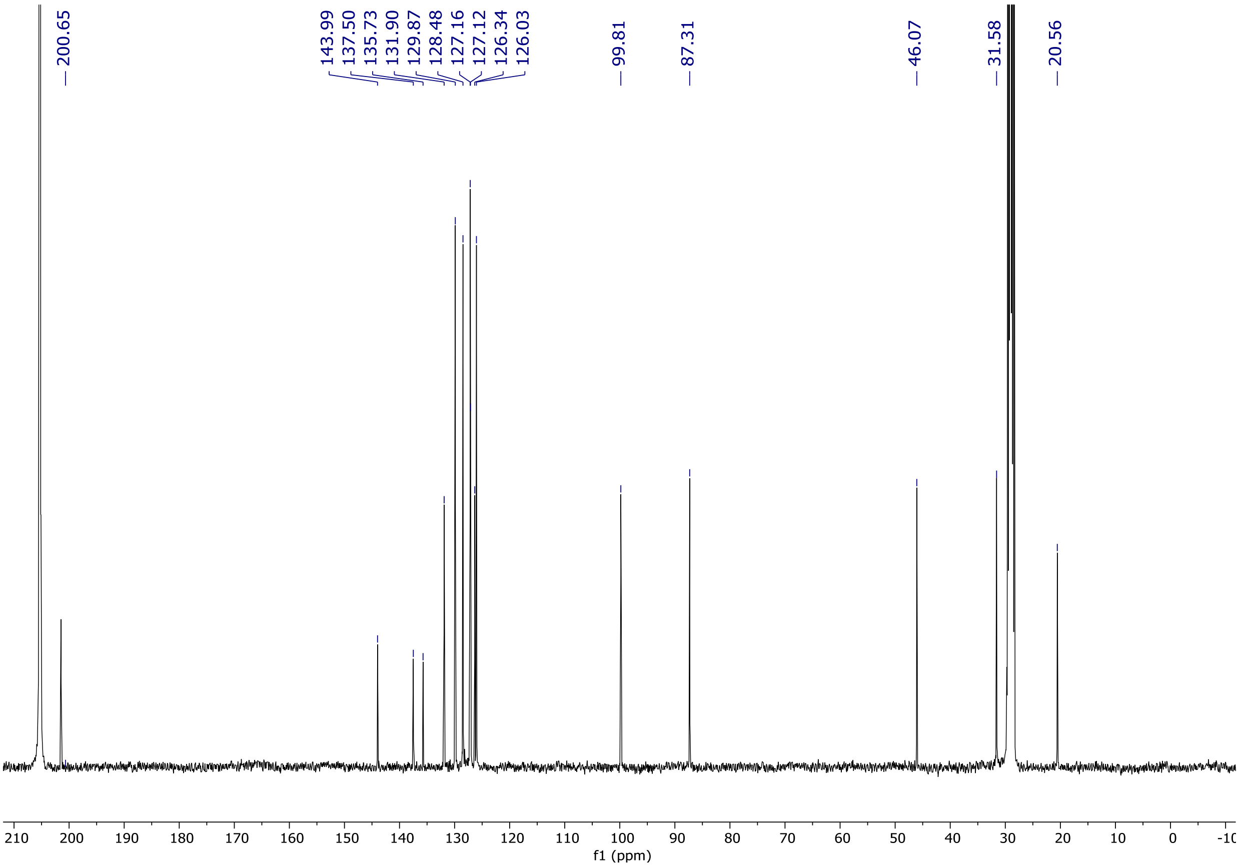

^{1}H NMR (400 MHz, $CDCl_3$)

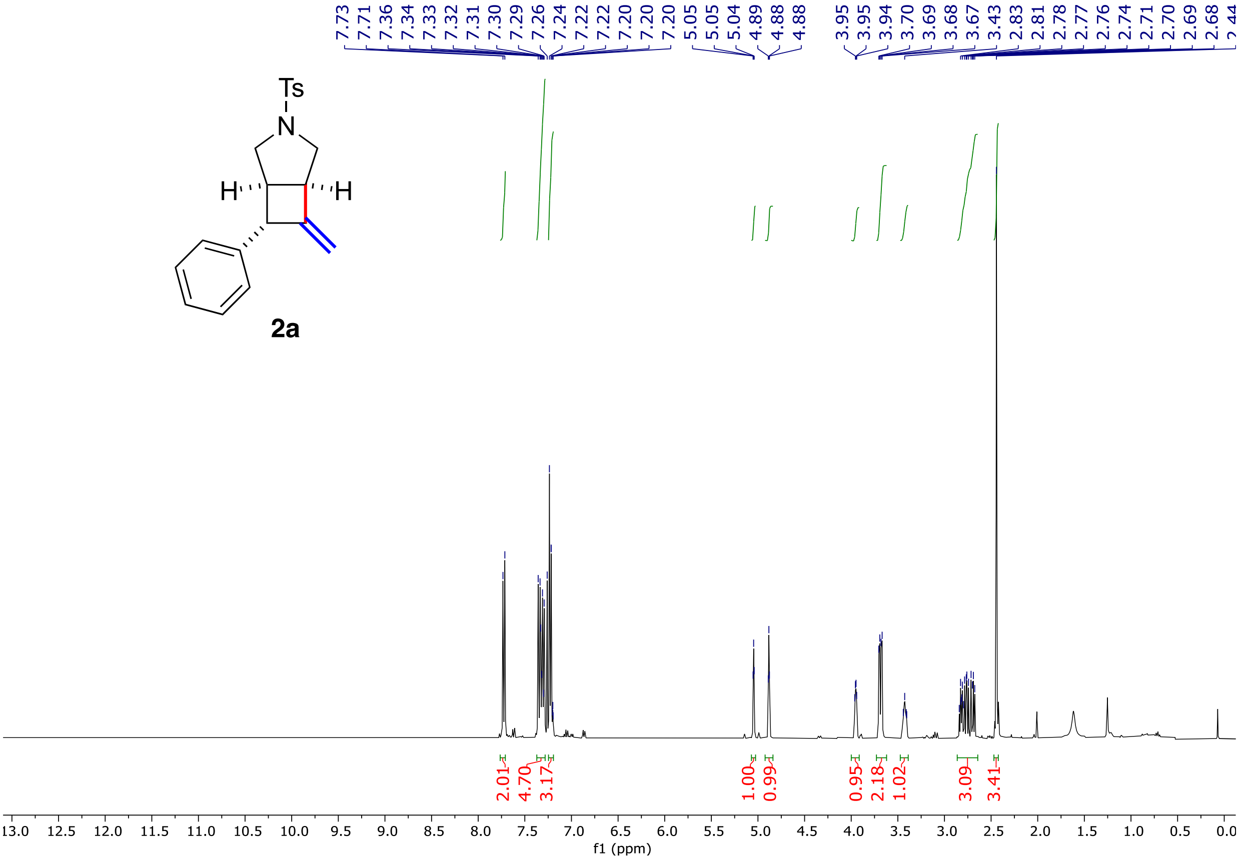

^{13}C NMR (101 MHz, $CDCl_3$)

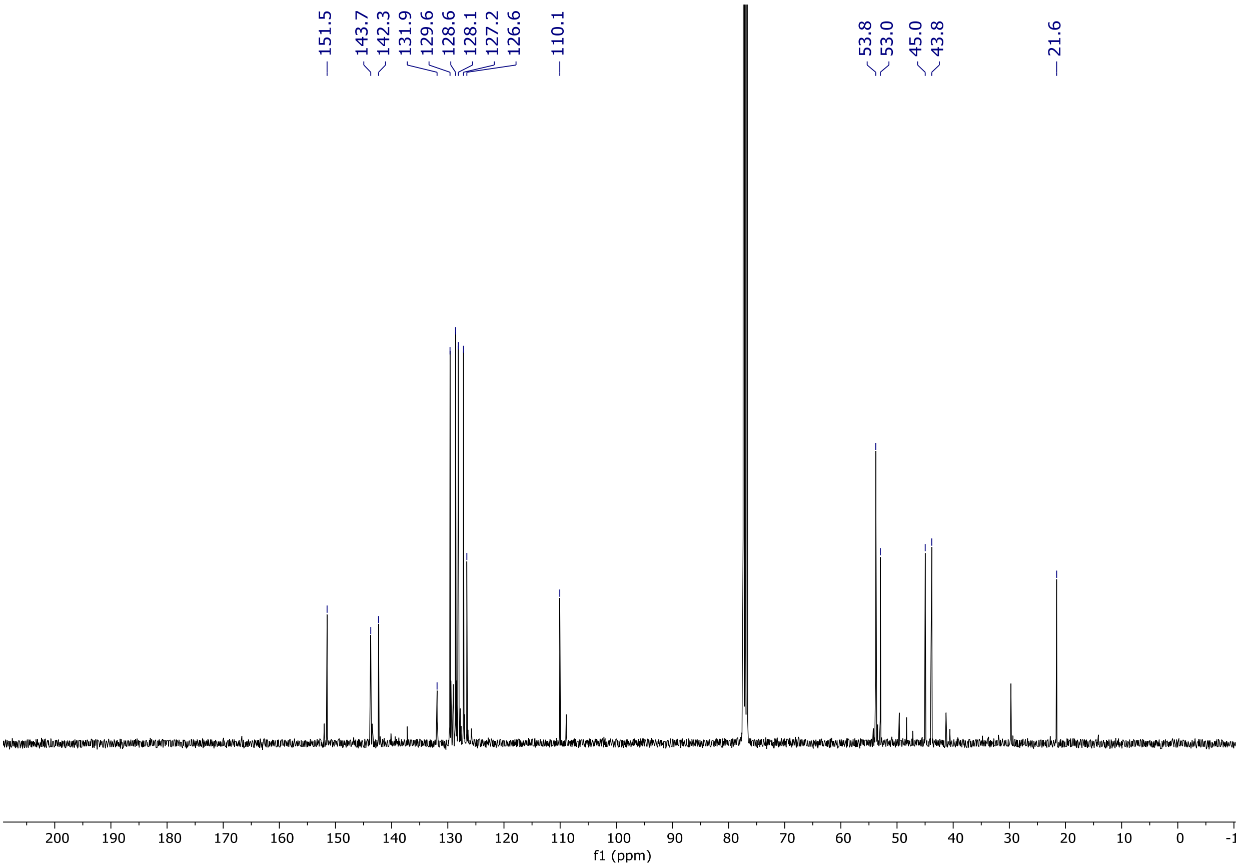

^{1}H NMR (300 MHz, $CDCl_3$)

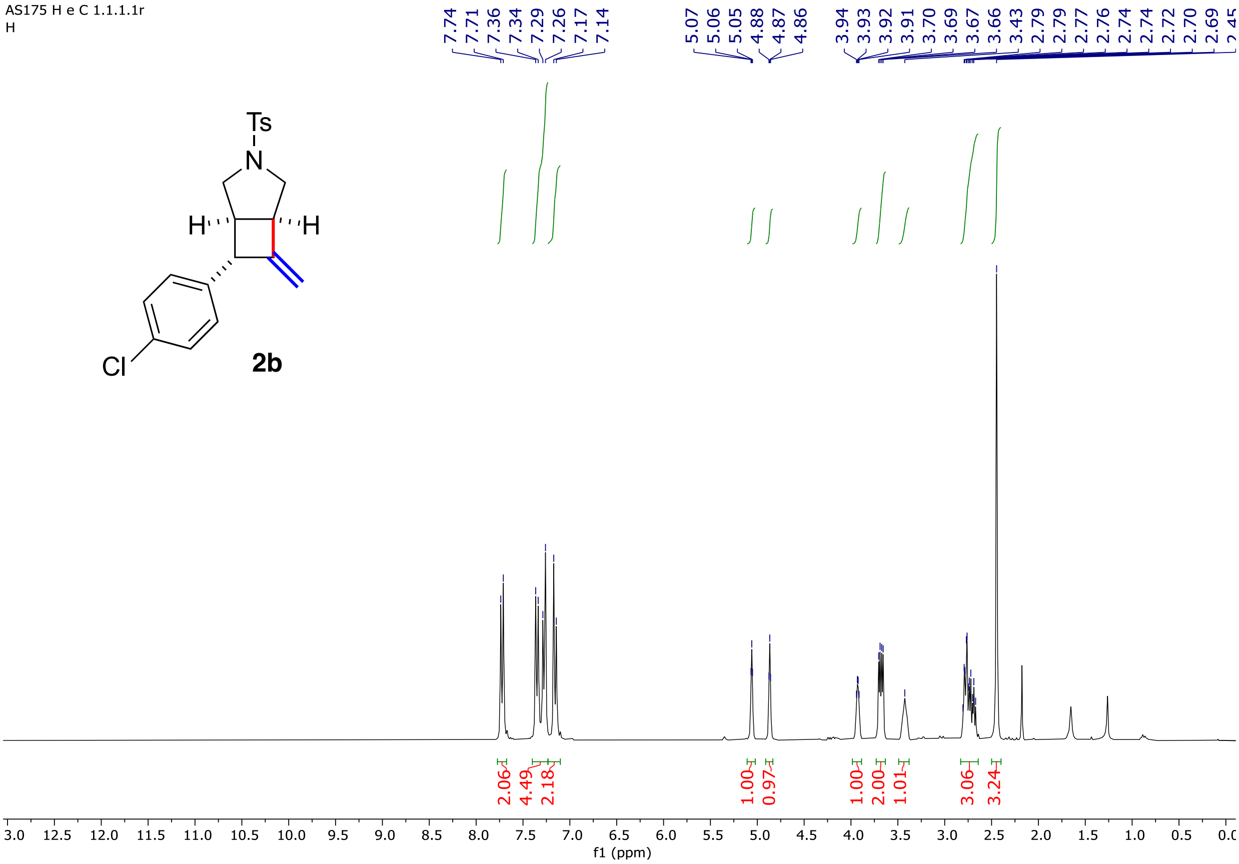

^{13}C NMR (75 MHz, $CDCl_3$)

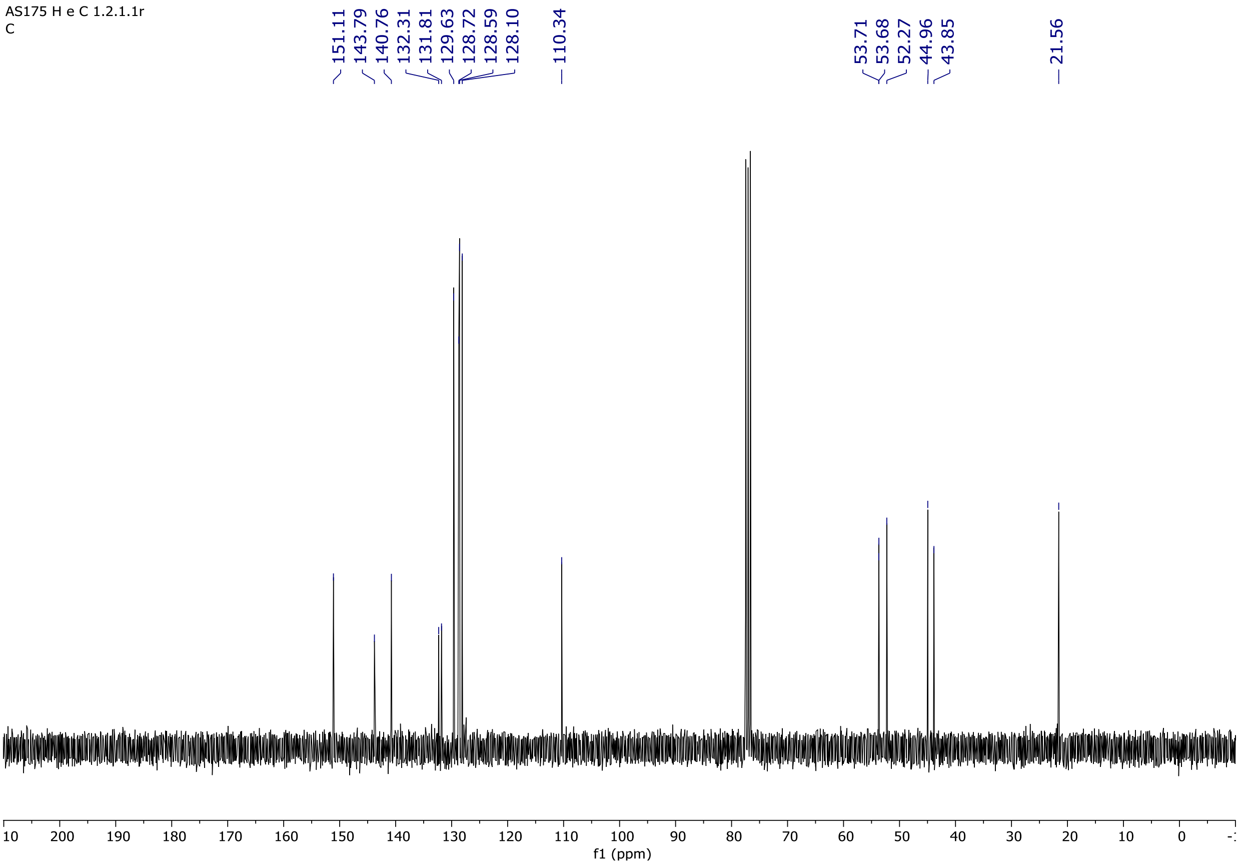

^{1}H NMR (400 MHz, $CDCl_3$)

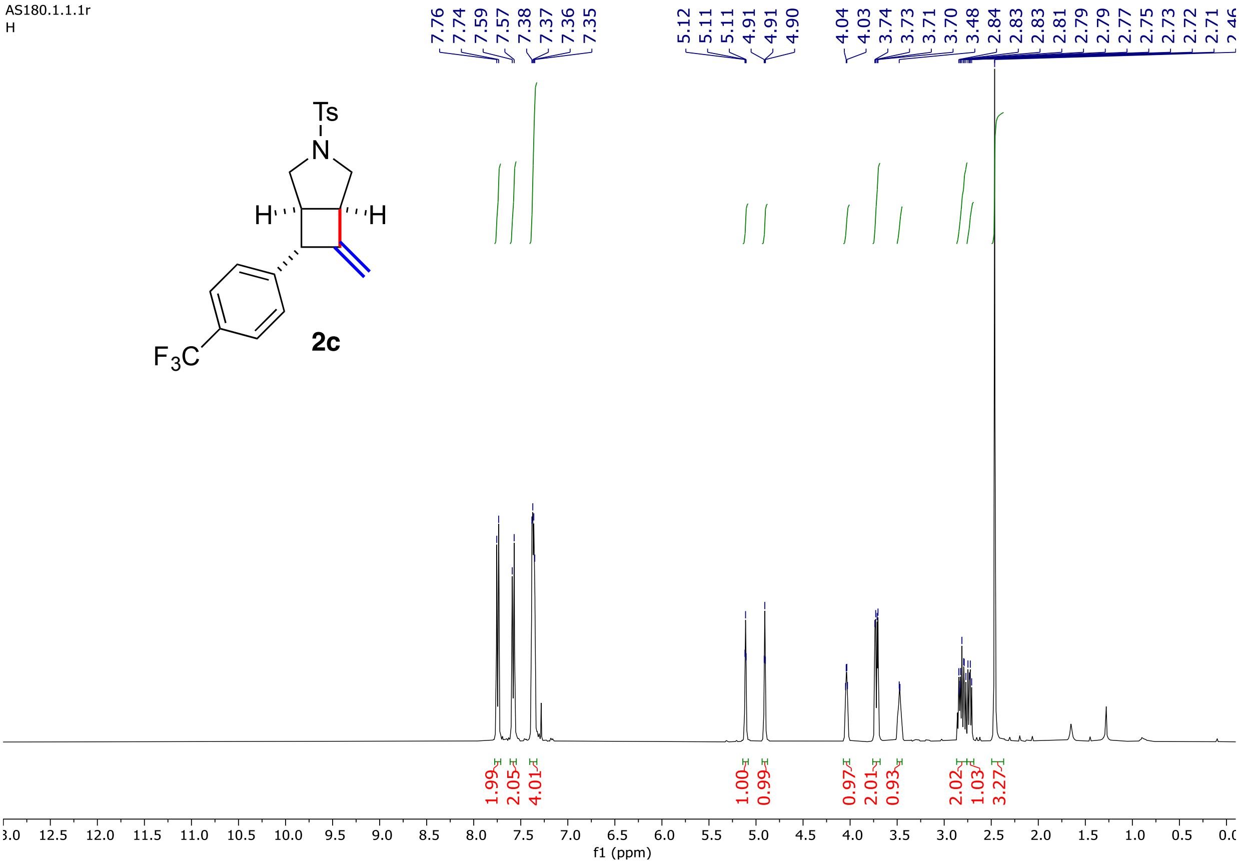

^{13}C NMR (101 MHz, $CDCl_3$)

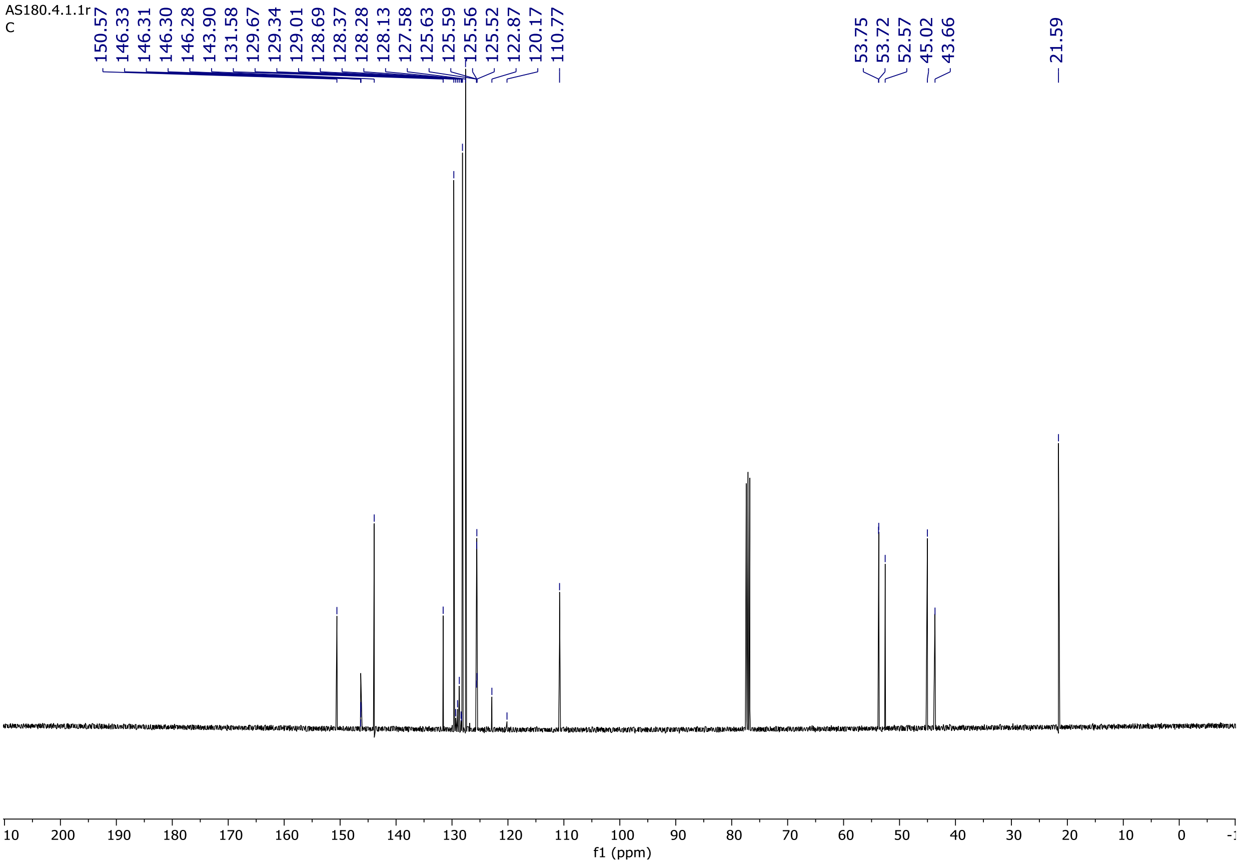

^{19}F NMR (564 MHz, $CDCl_3$)

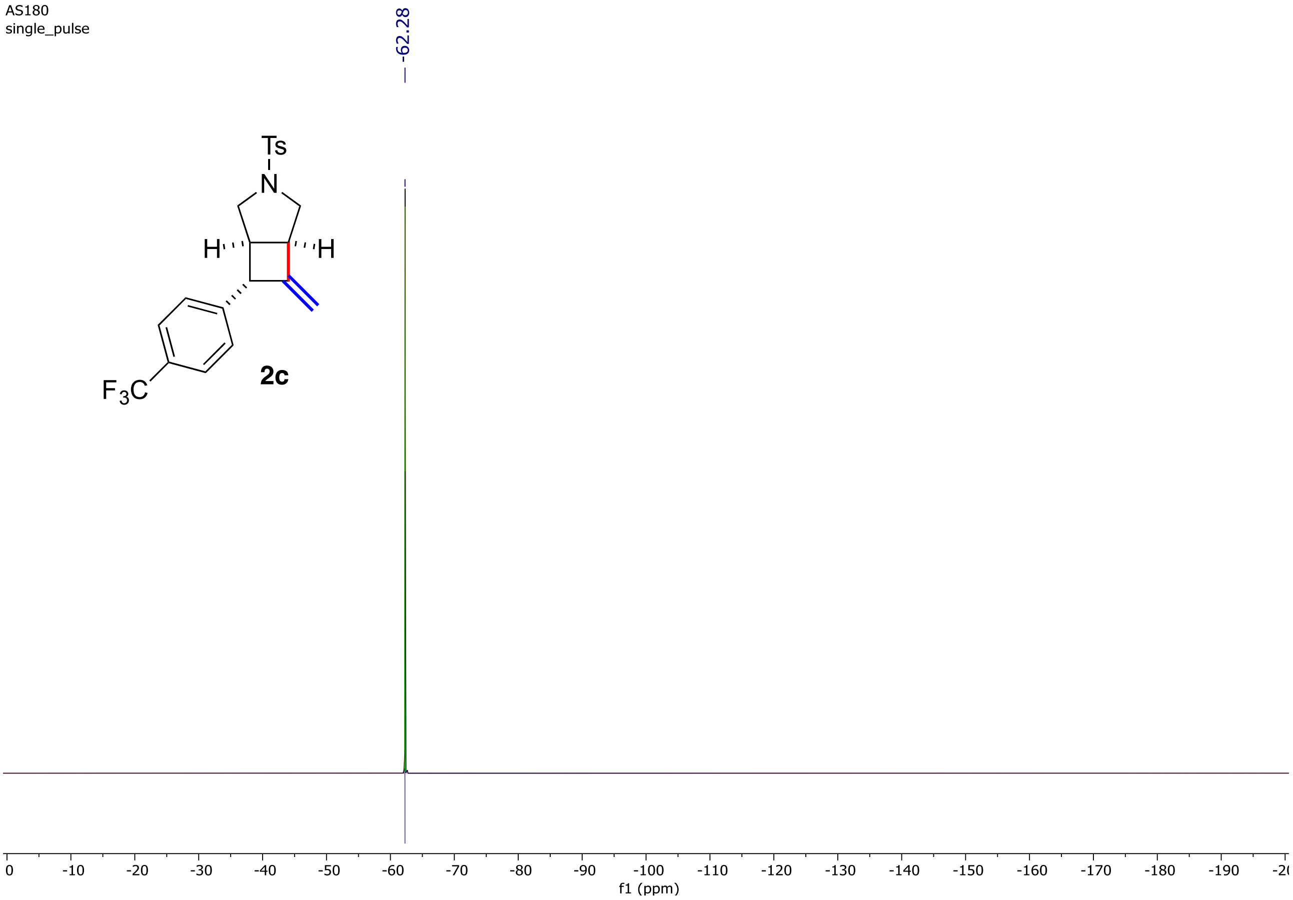

^{1}H NMR (600 MHz, $CDCl_3$)

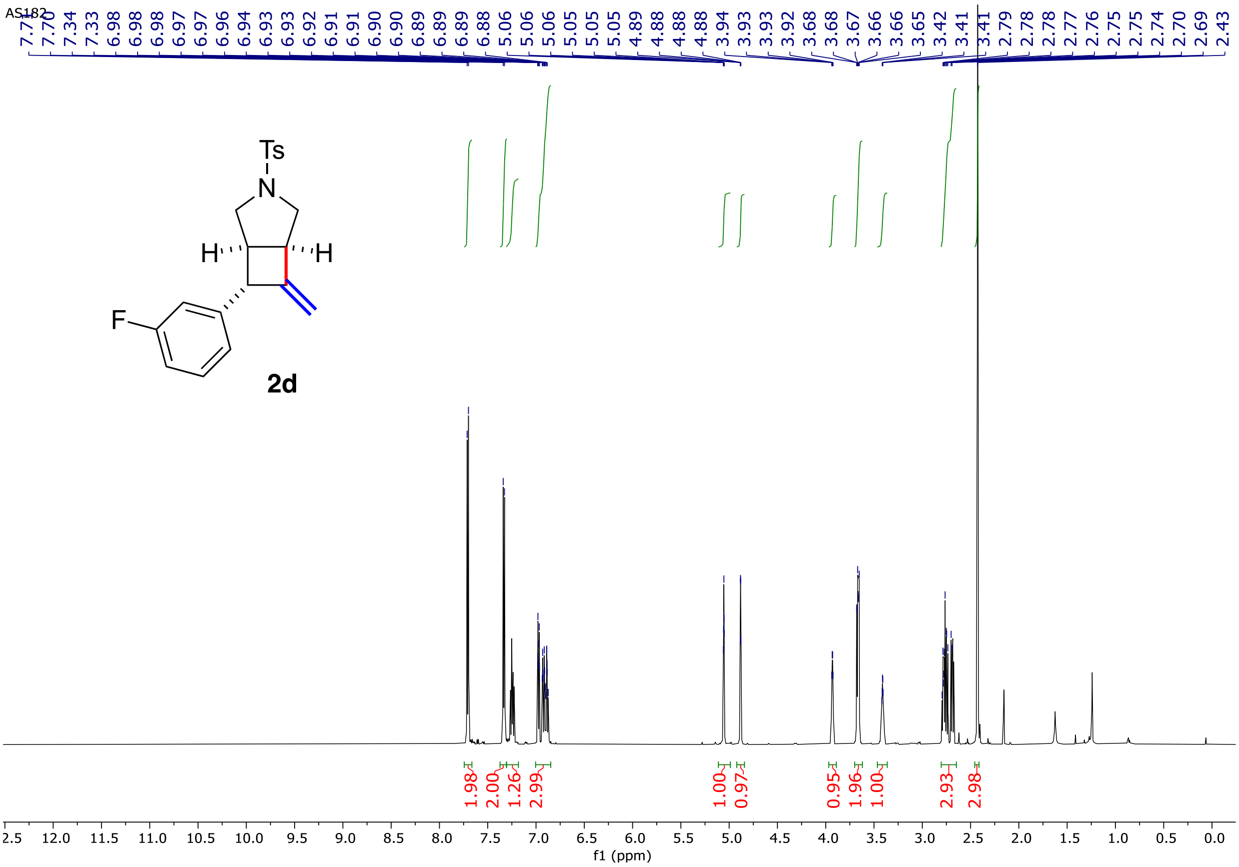

^{13}C NMR (151 MHz, $CDCl_3$)

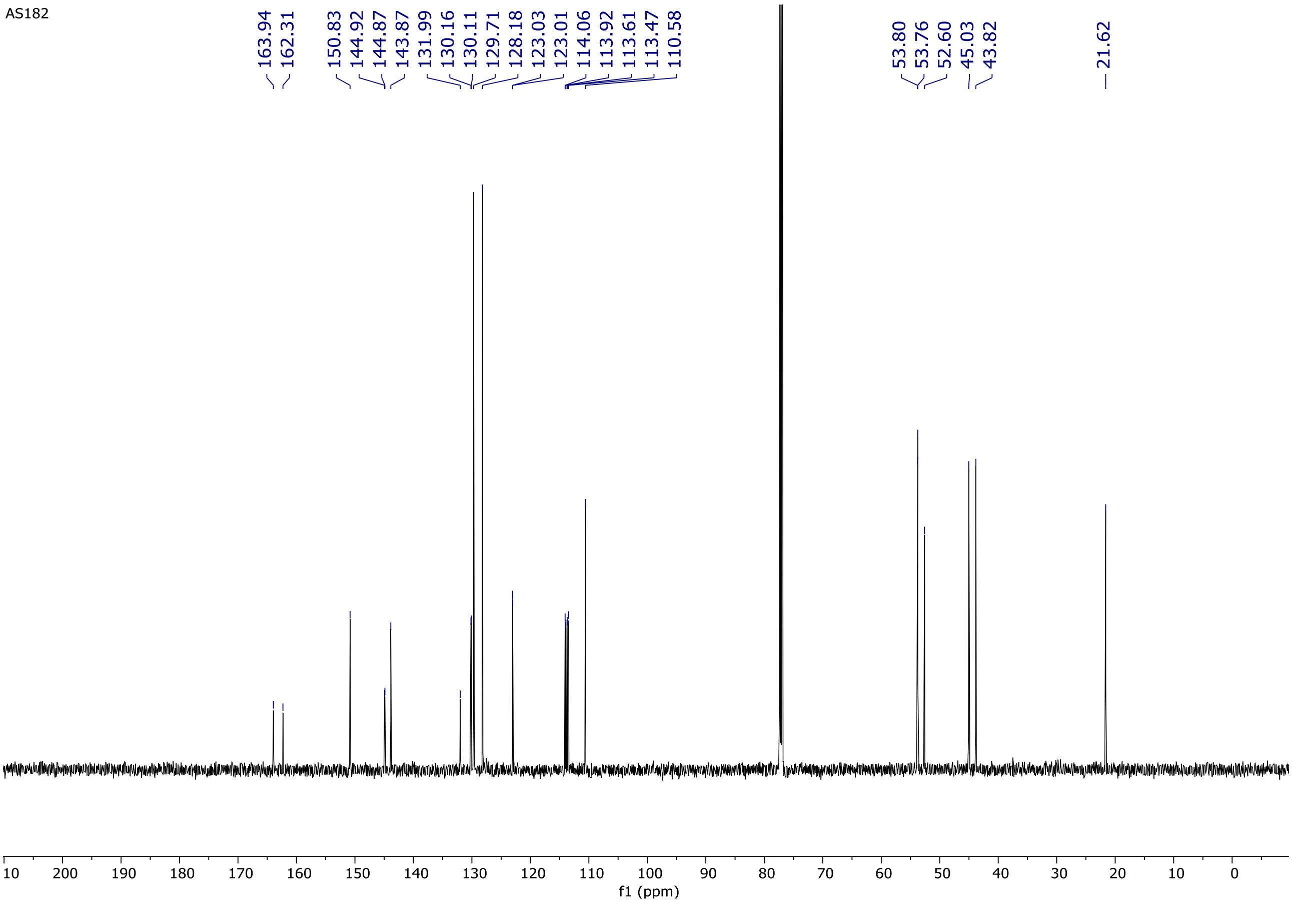

^{19}F NMR (564 MHz, $CDCl_3$)

AS182

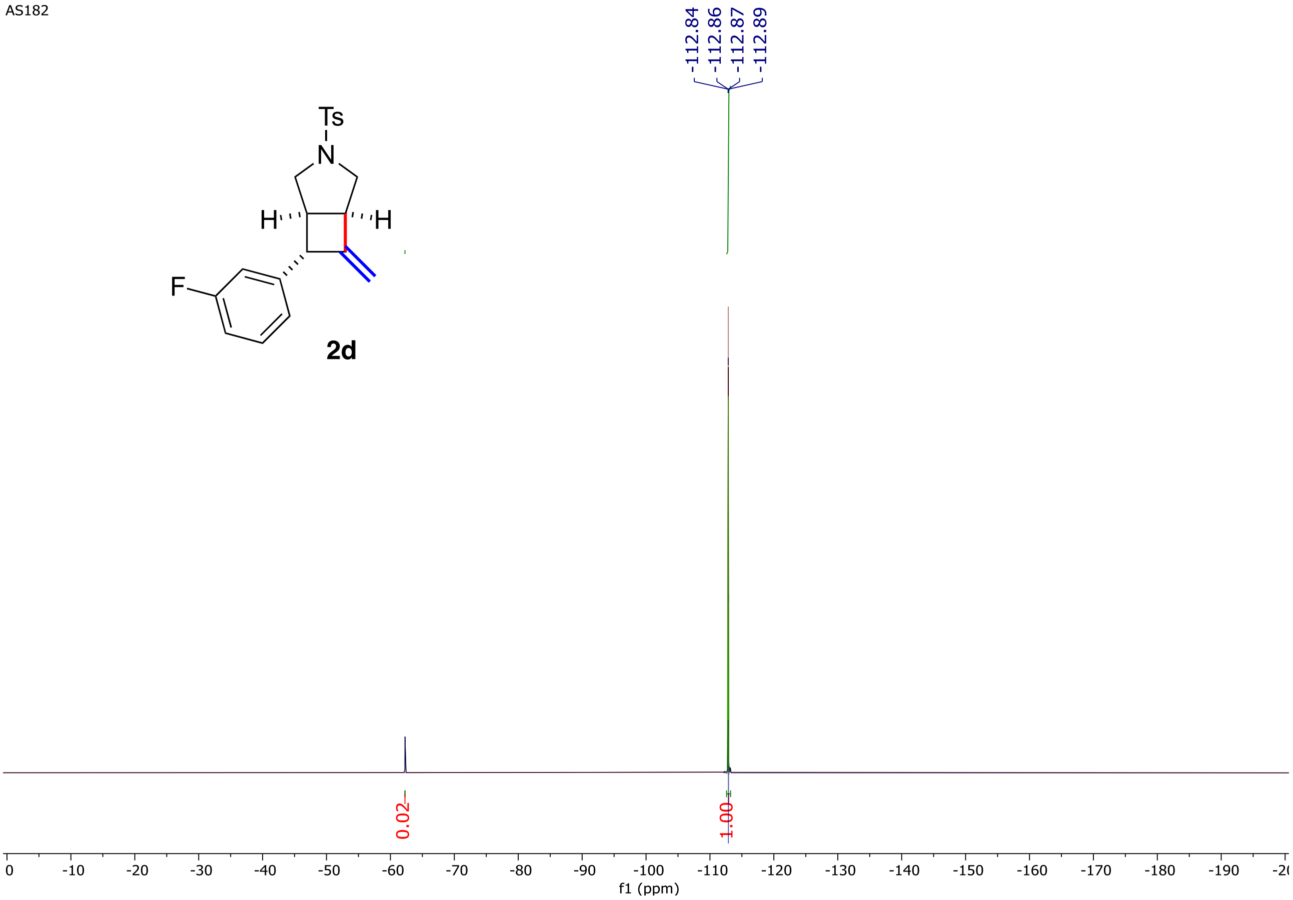

^{1}H NMR (400 MHz, $CDCl_3$)

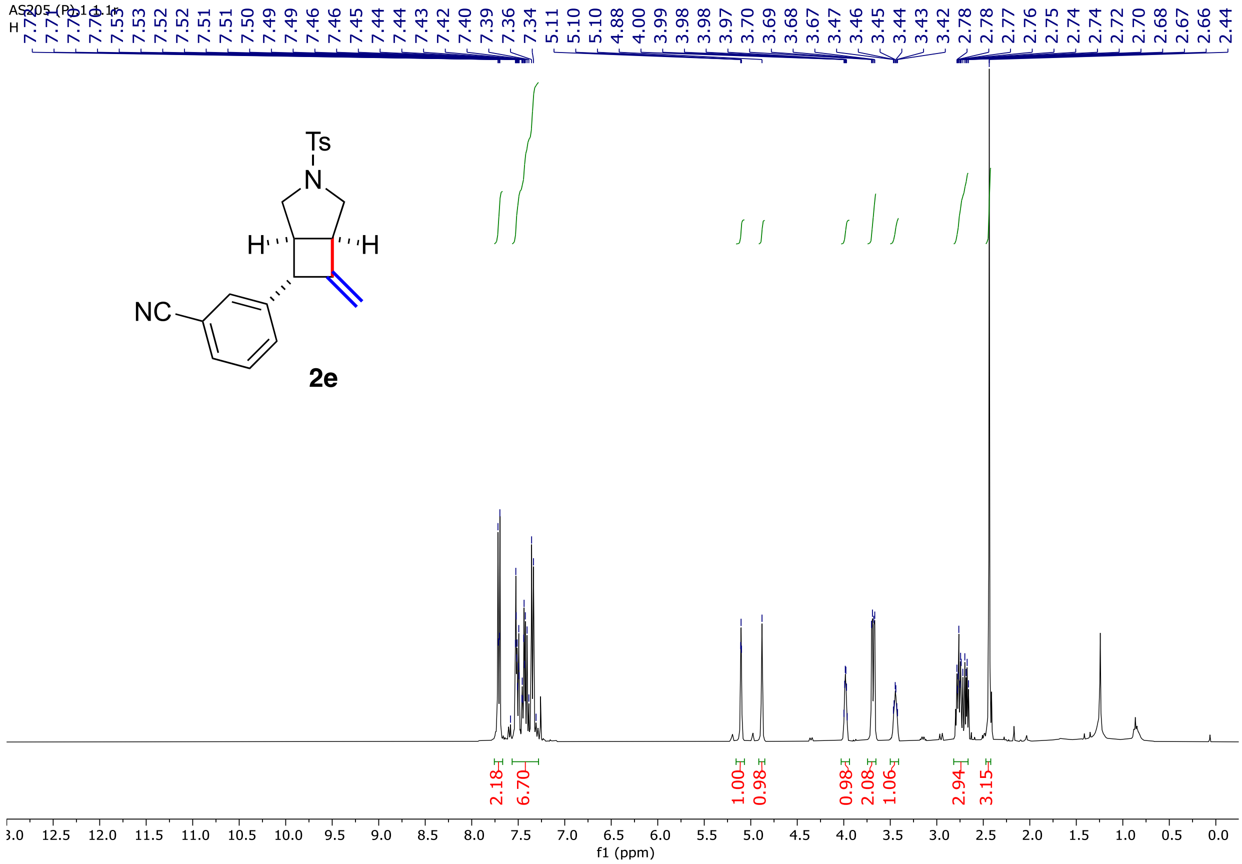

^{13}C NMR (101 MHz, $CDCl_3$)

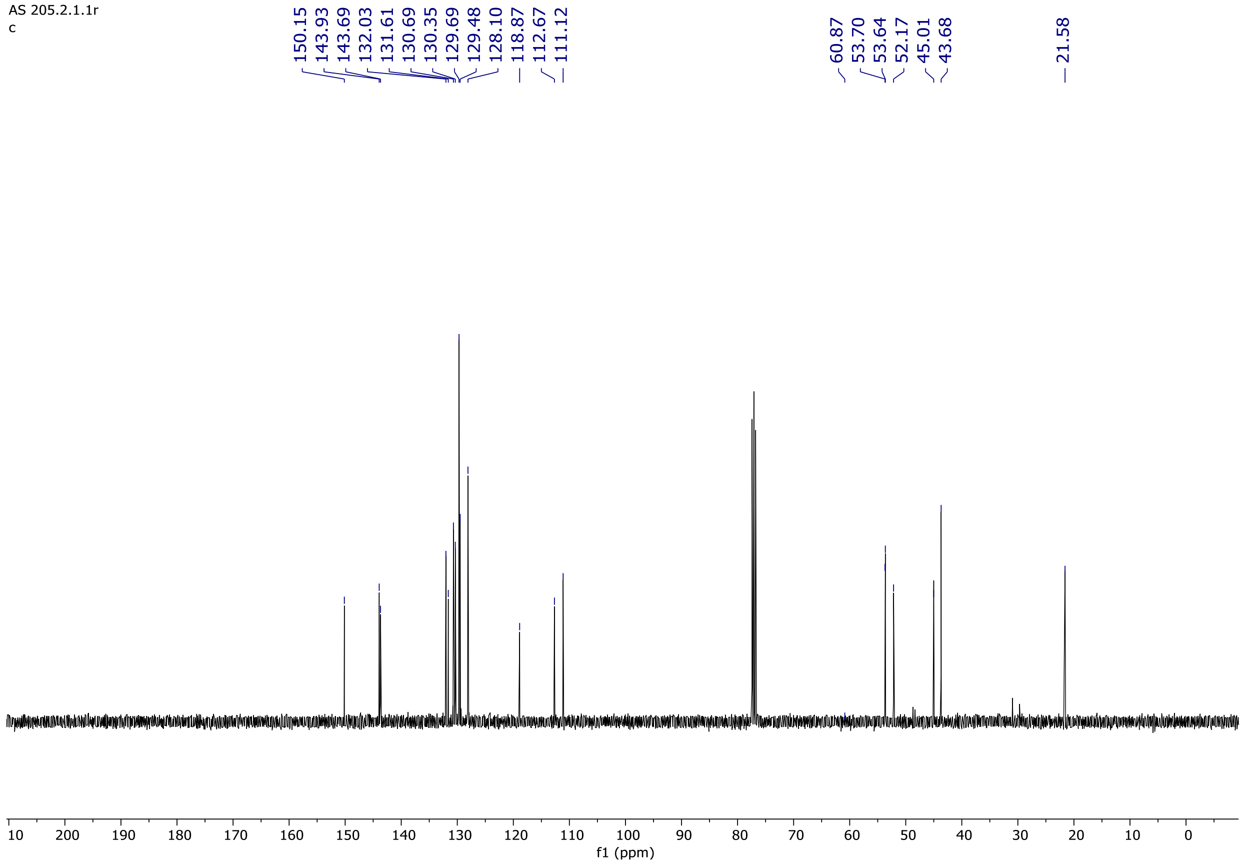

^{1}H NMR (400 MHz, $CDCl_3$)

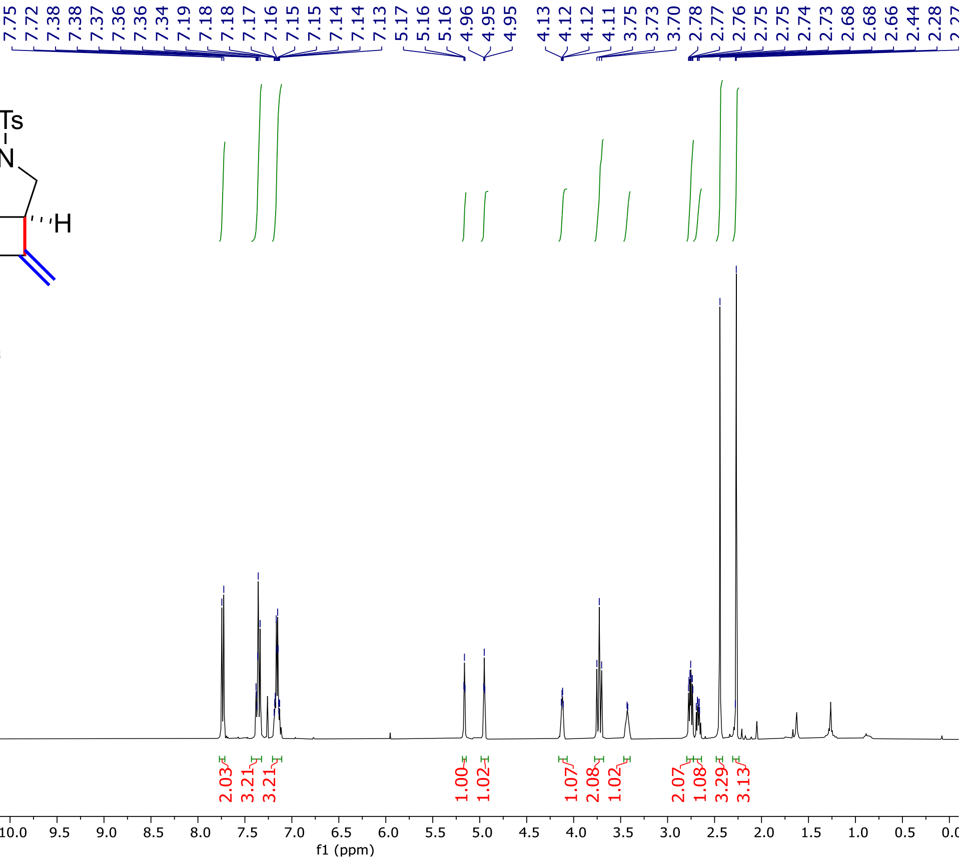

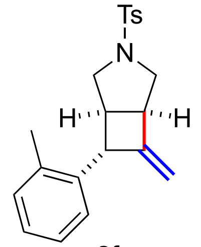

2f

^{13}C NMR (101 MHz, $CDCl_3$)

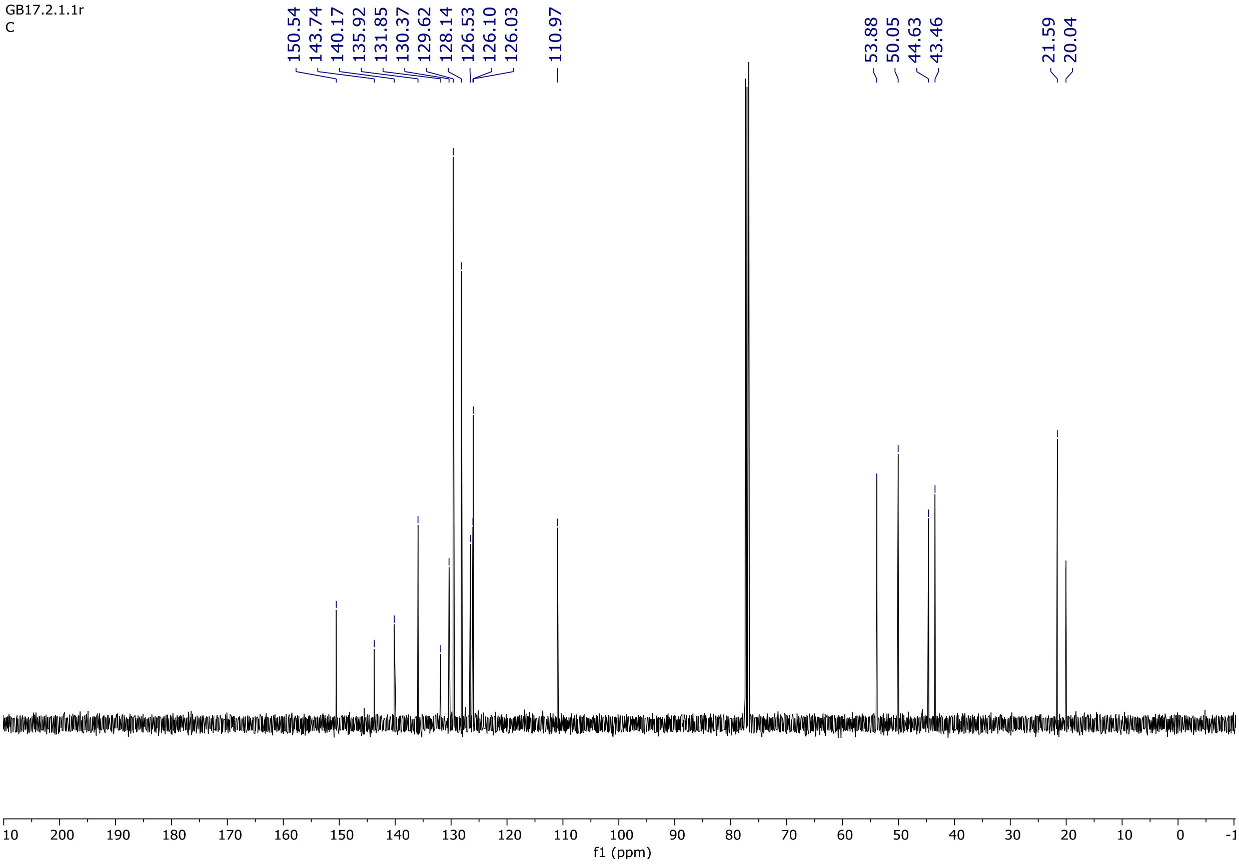

^{1}H NMR (400 MHz, $CDCl_3$)

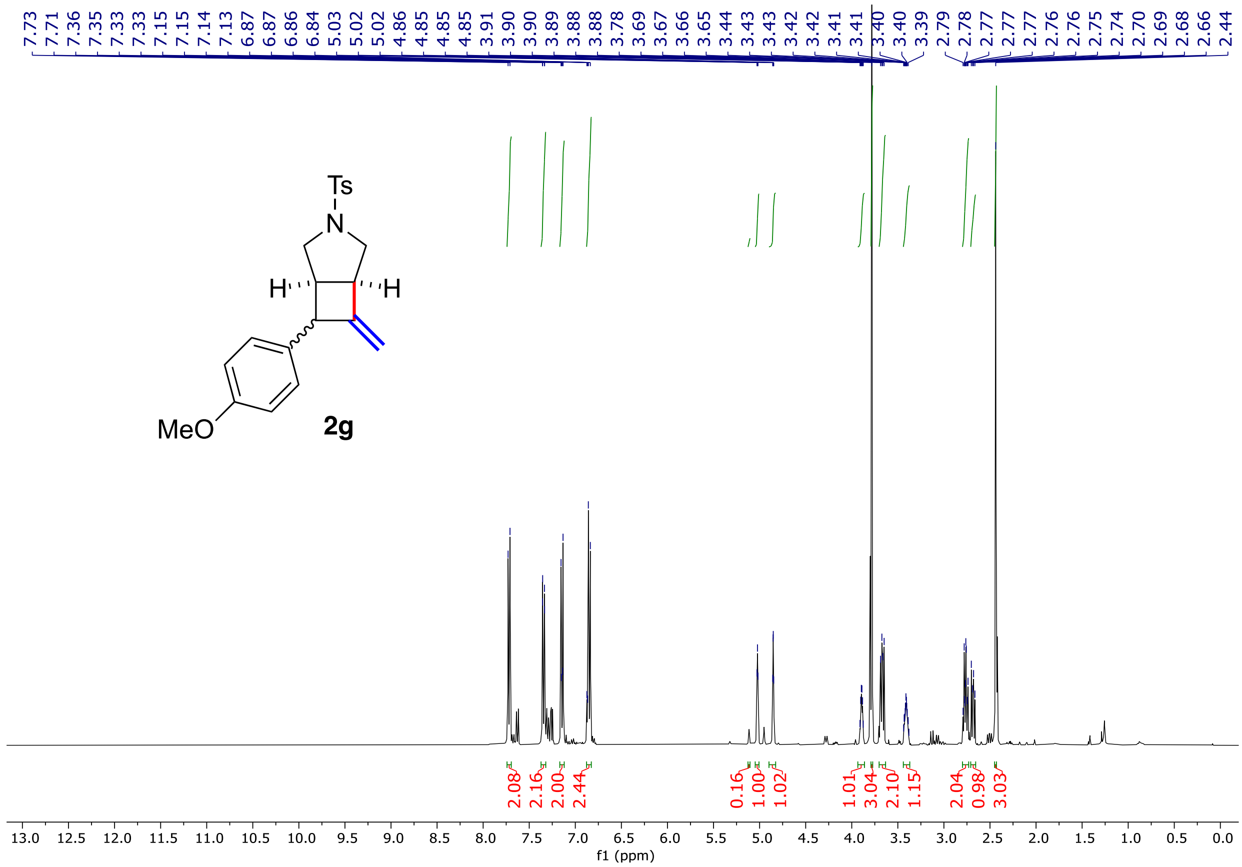

^{13}C NMR (101 MHz, $CDCl_3$)

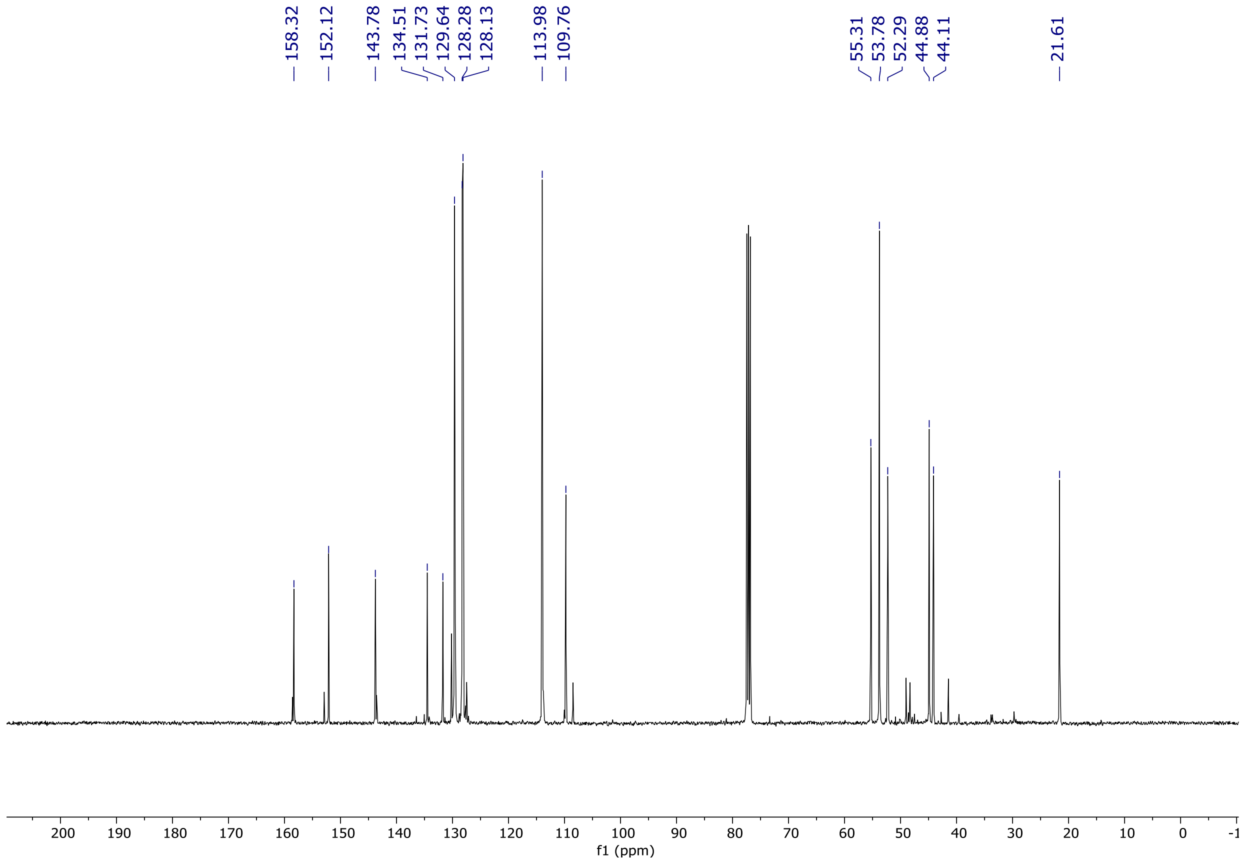

^{1}H NMR (400 MHz, $CDCl_3$)

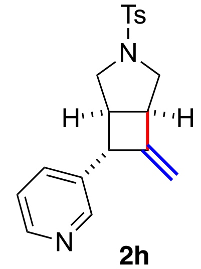

2h

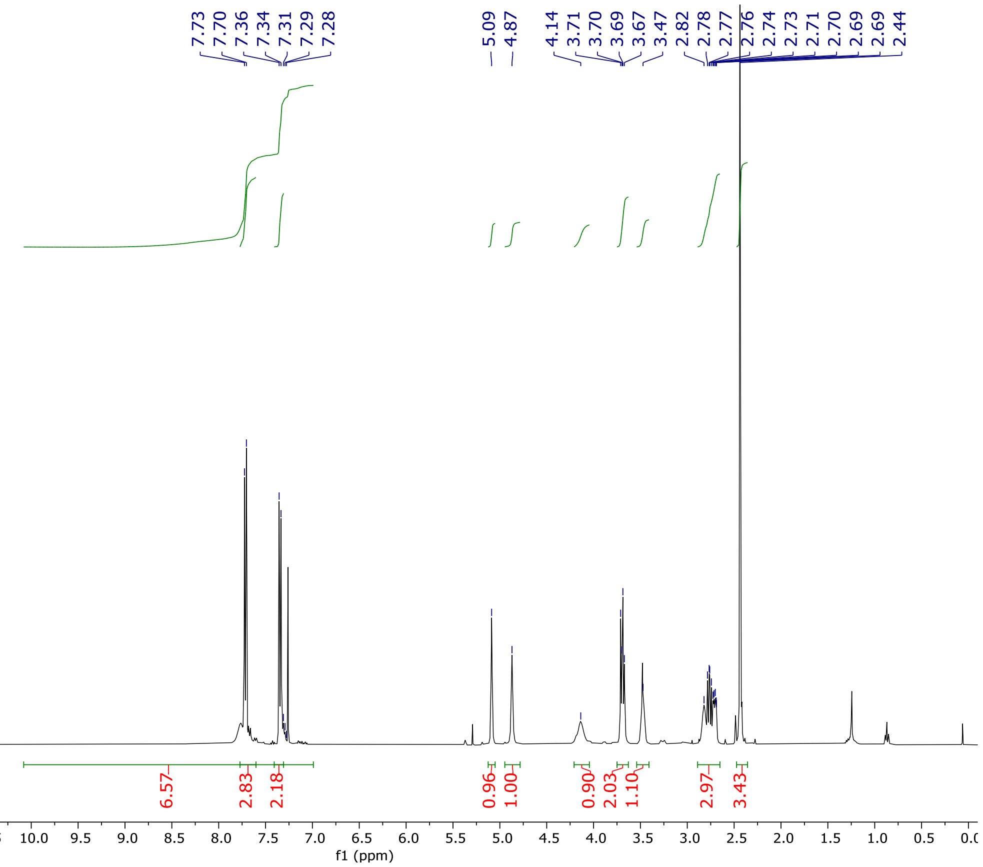

^{13}C NMR (101 MHz, $CDCl_3$)

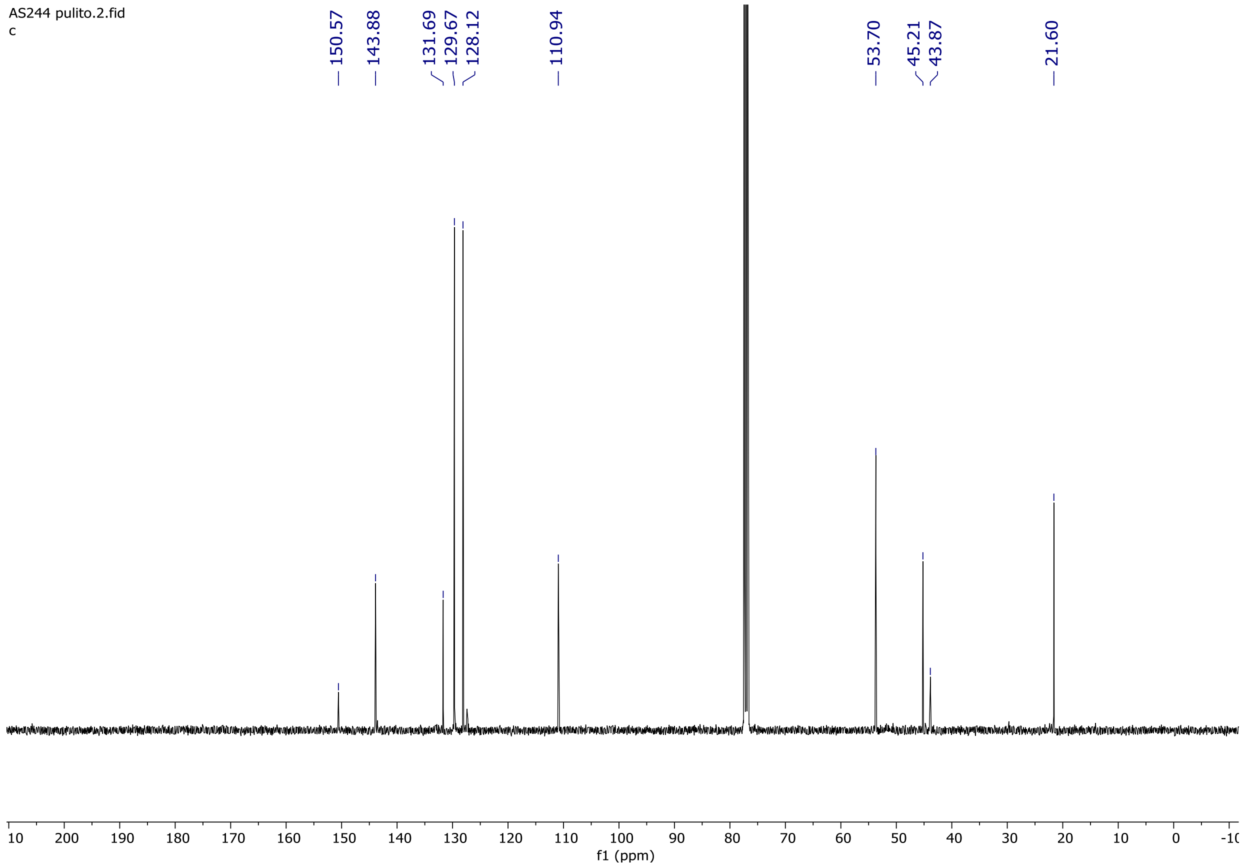

^{1}H NMR (400 MHz, $CDCl_3$)

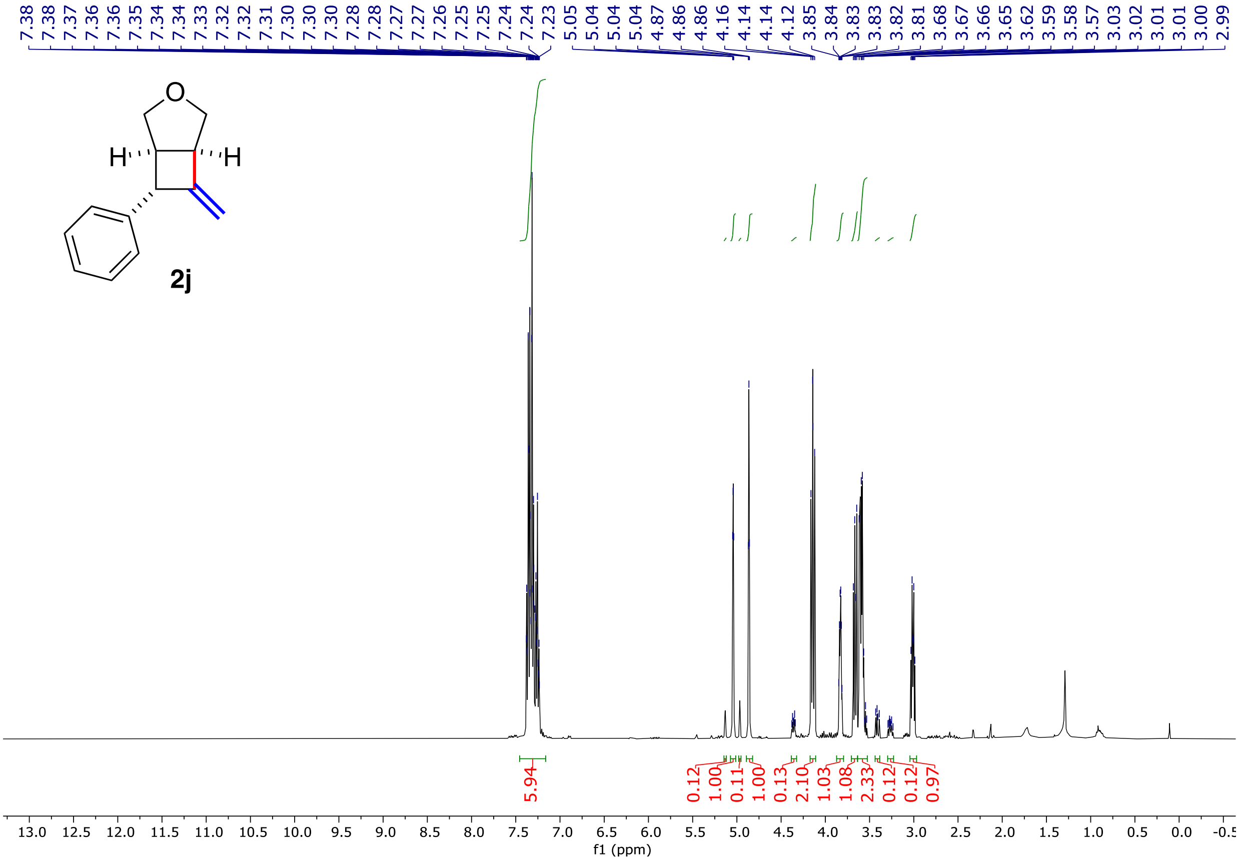

^{13}C NMR (101 MHz, $CDCl_3$)

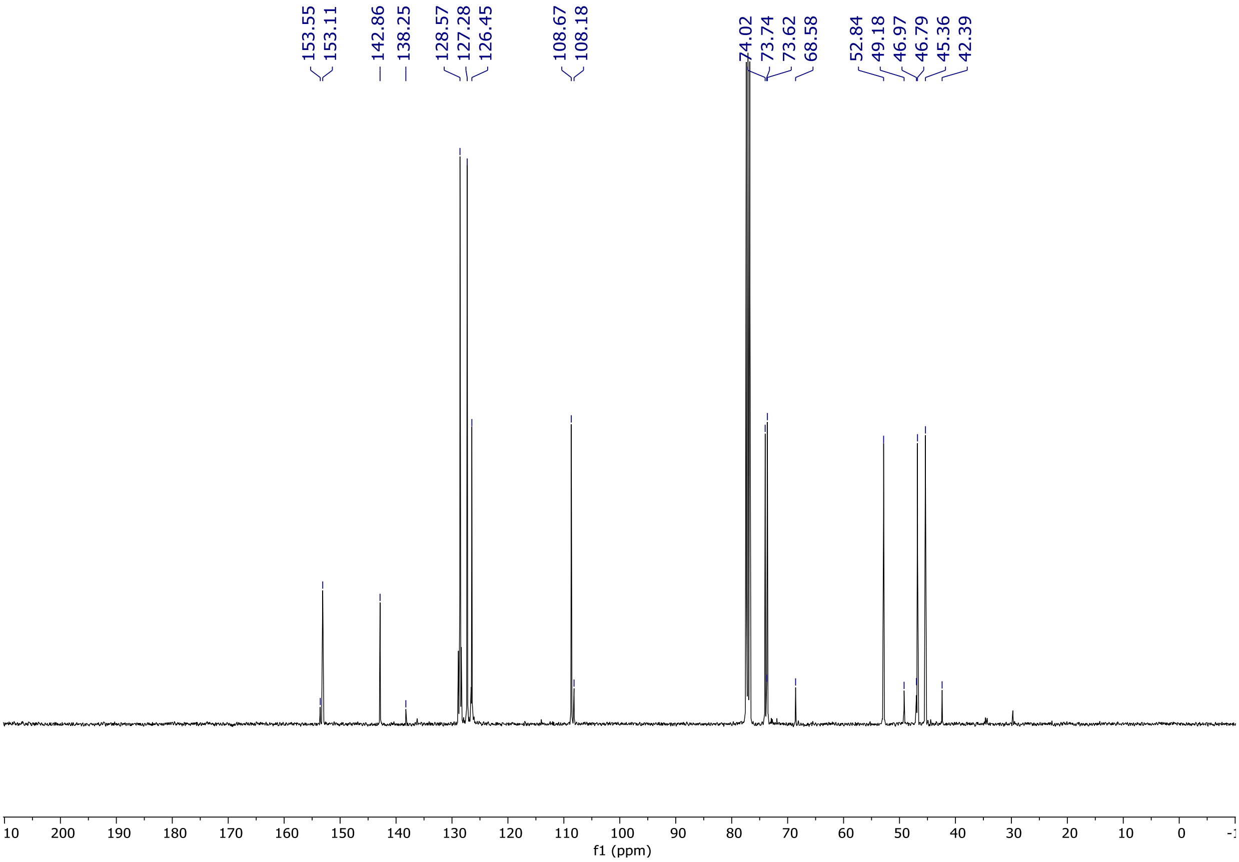

^{1}H NMR (400 MHz, $CDCl_3$)

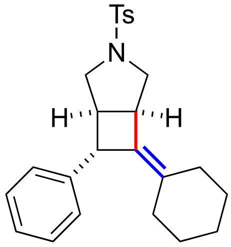

2k

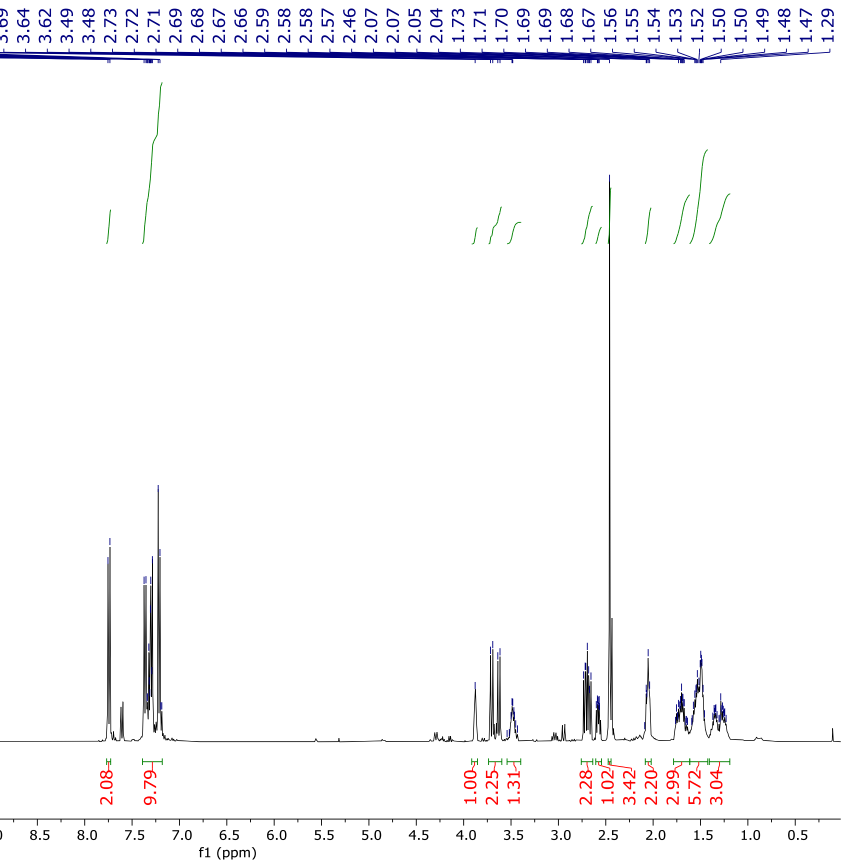

^{13}C NMR (101 MHz, $CDCl_3$)

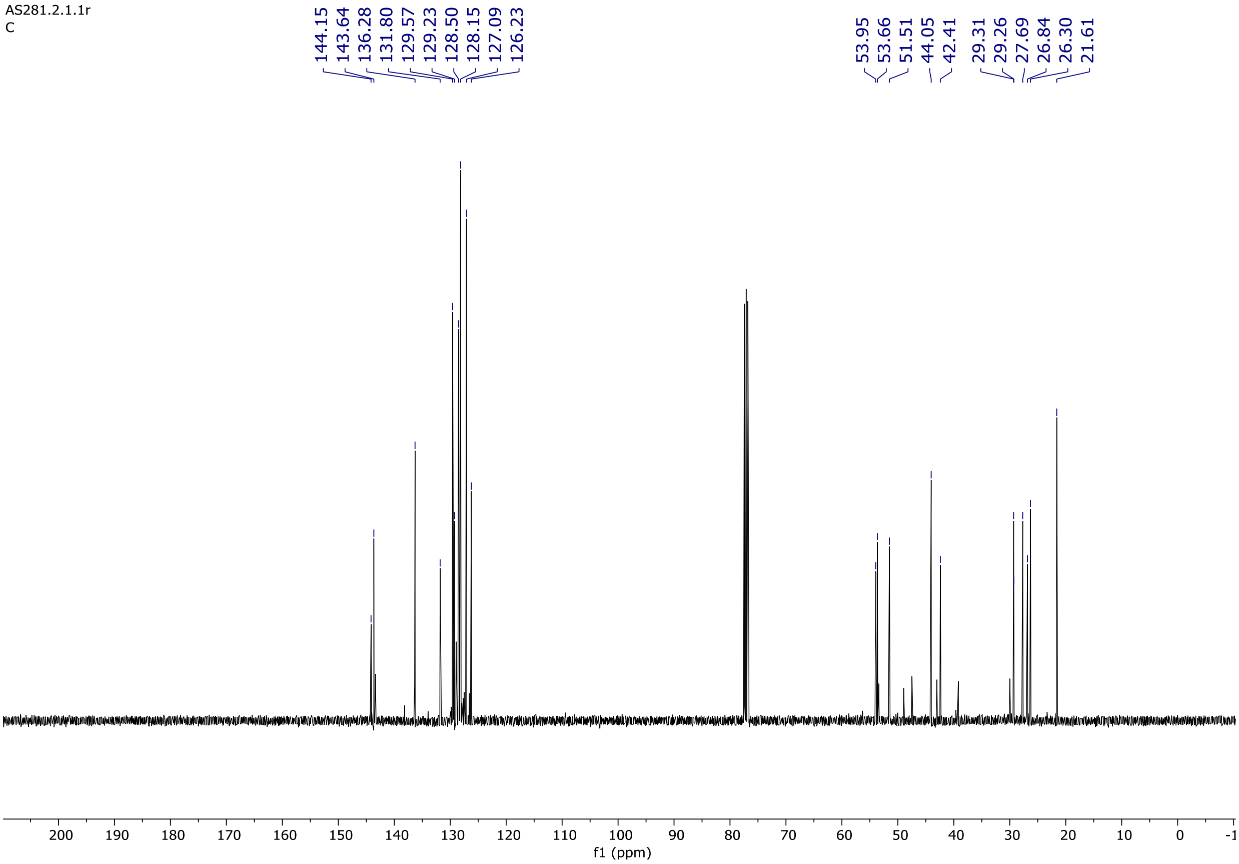

^{1}H NMR (400 MHz, acetone)

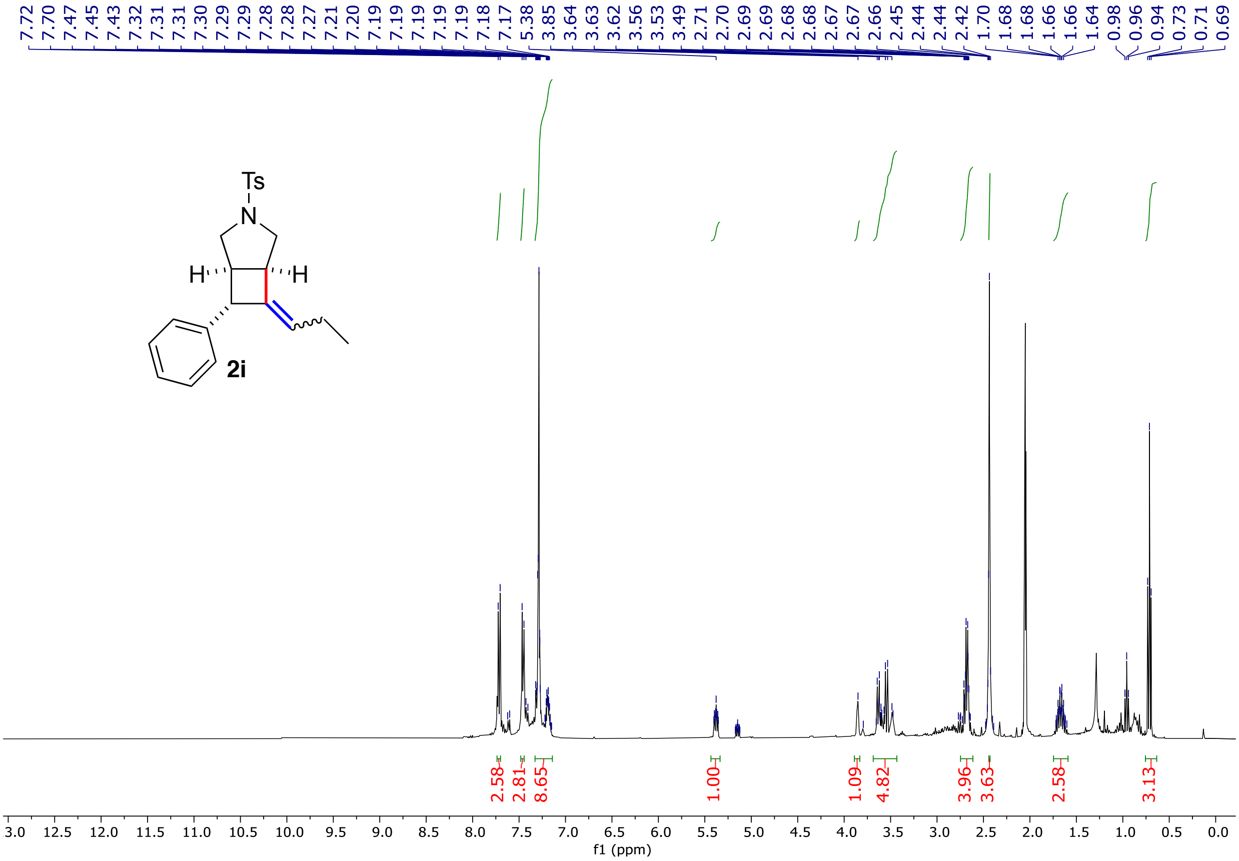

^{13}C NMR (101 MHz, acetone)

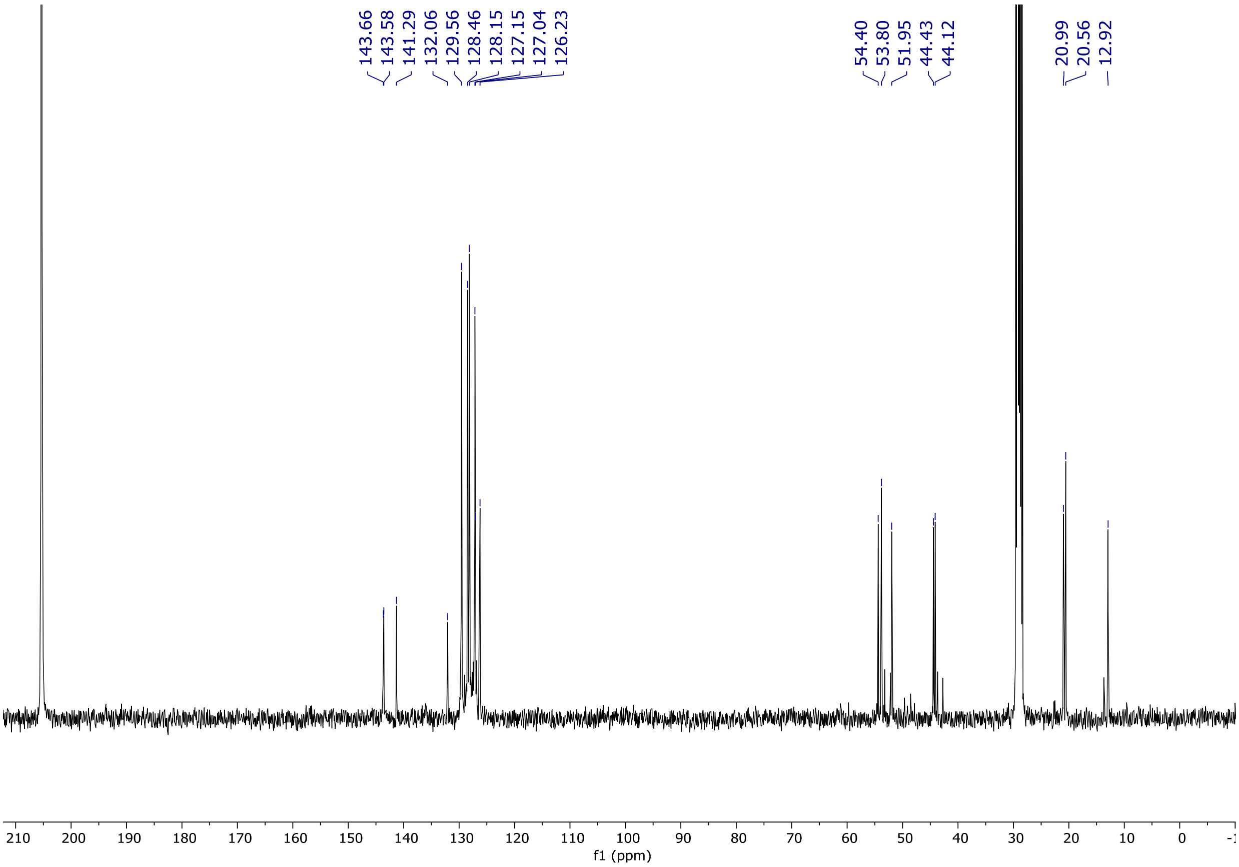

^{1}H NMR (400 MHz, $CDCl_3$)

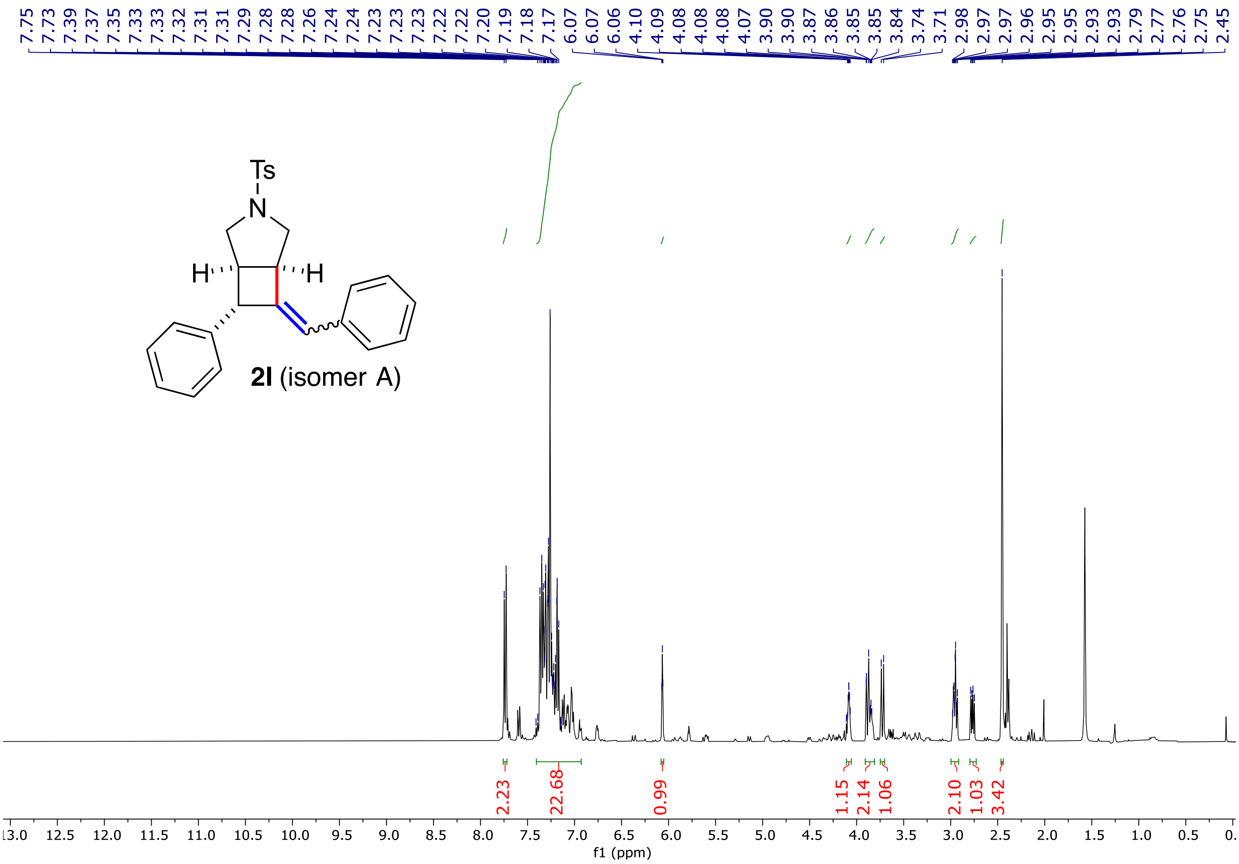

^{13}C NMR (101 MHz, $CDCl_3$)

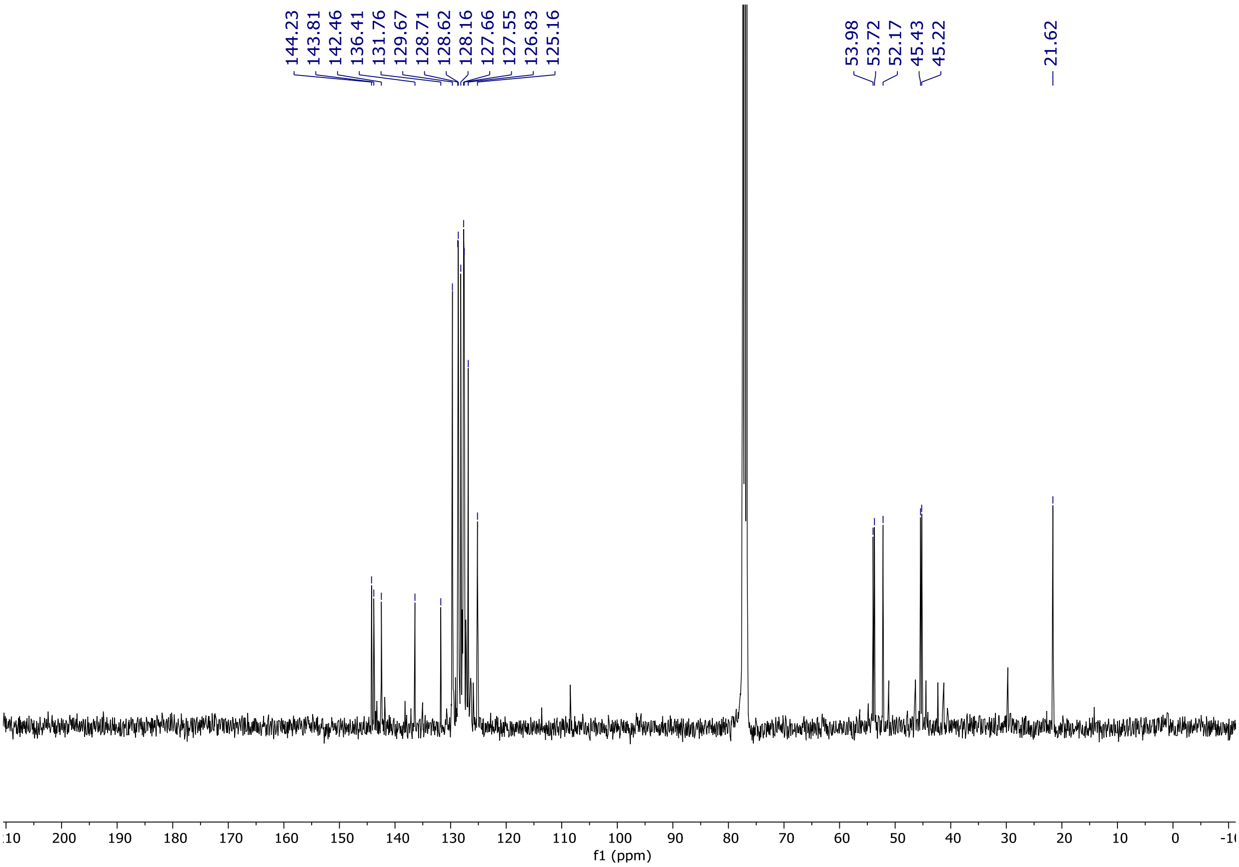

^{1}H NMR (400 MHz, $CDCl_3$)

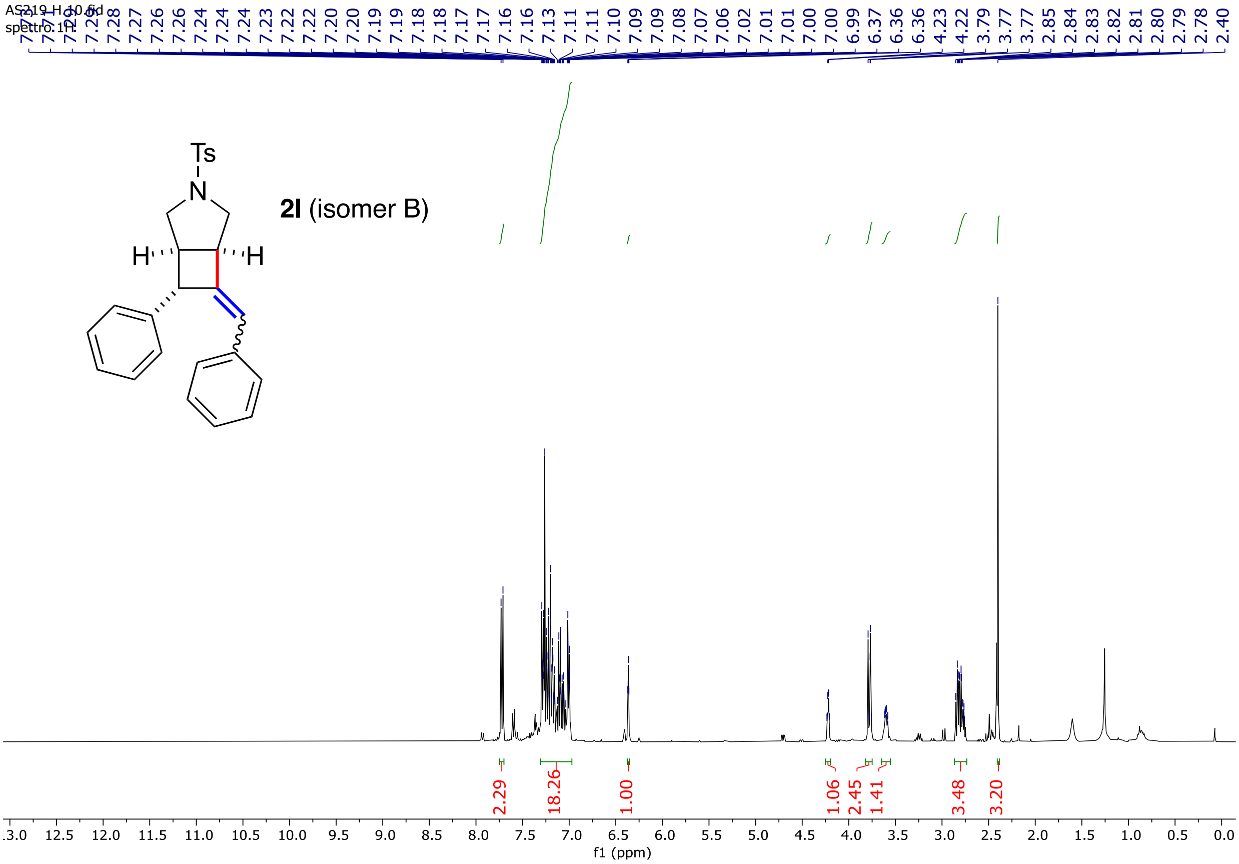

^{13}C NMR (101 MHz, $CDCl_3$)

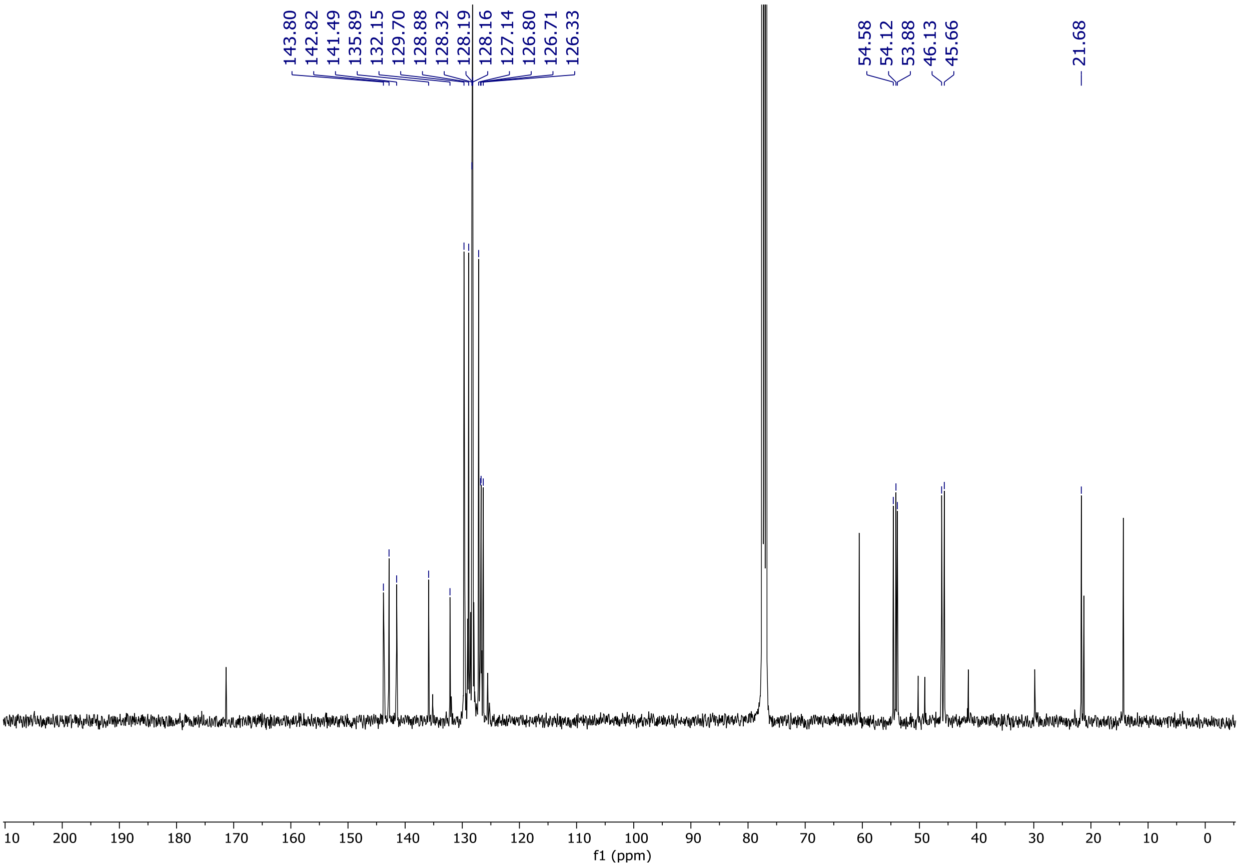

^{1}H NMR (600 MHz, $CDCl_3$)

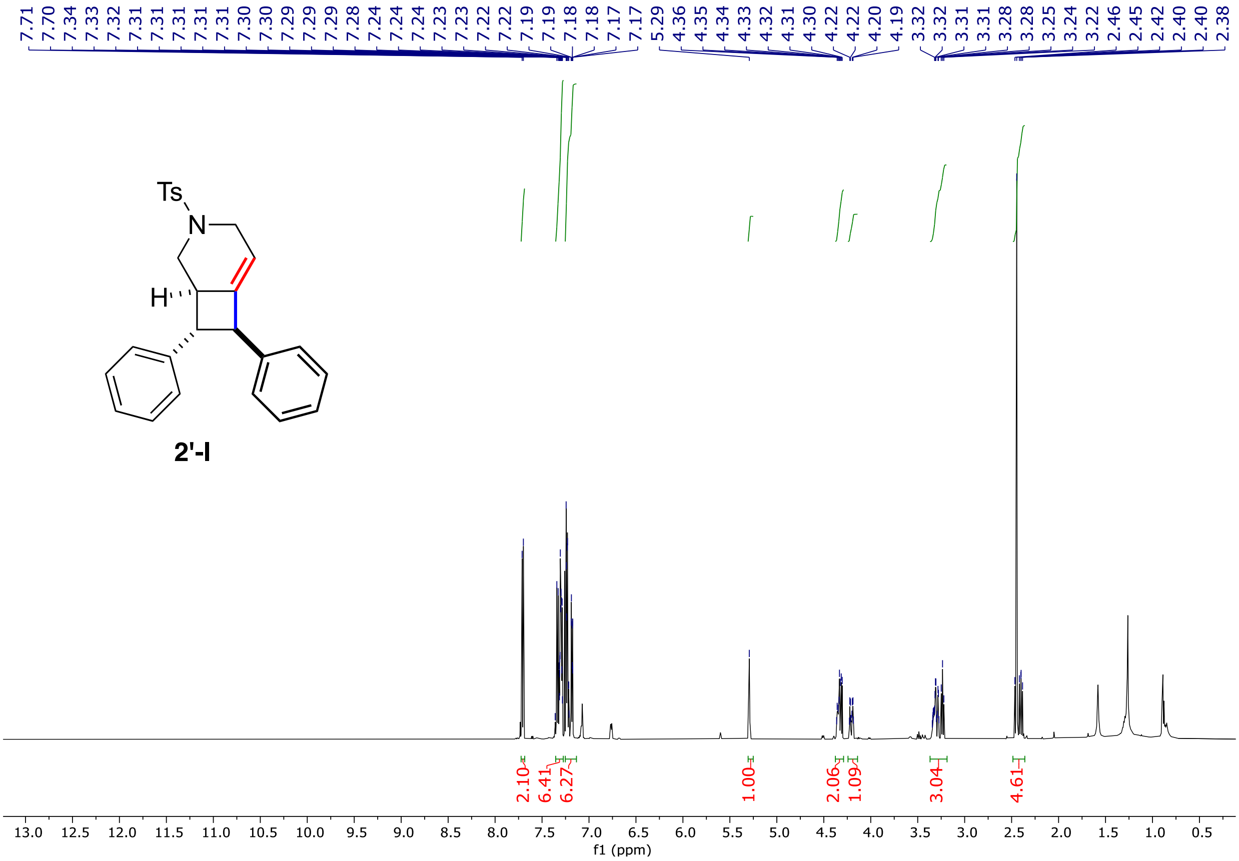

^{13}C NMR (151 MHz, $CDCl_3$)

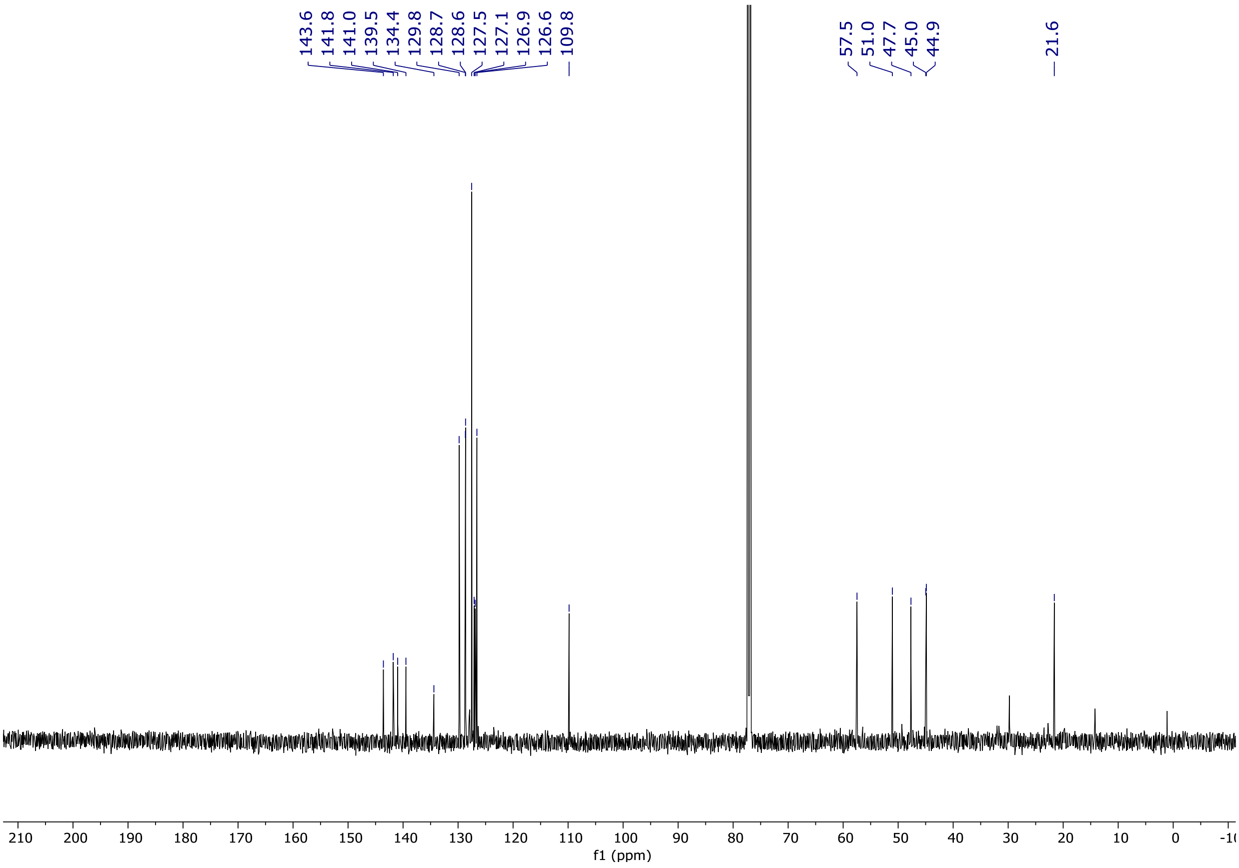

^{1}H NMR (400 MHz, $CDCl_3$)

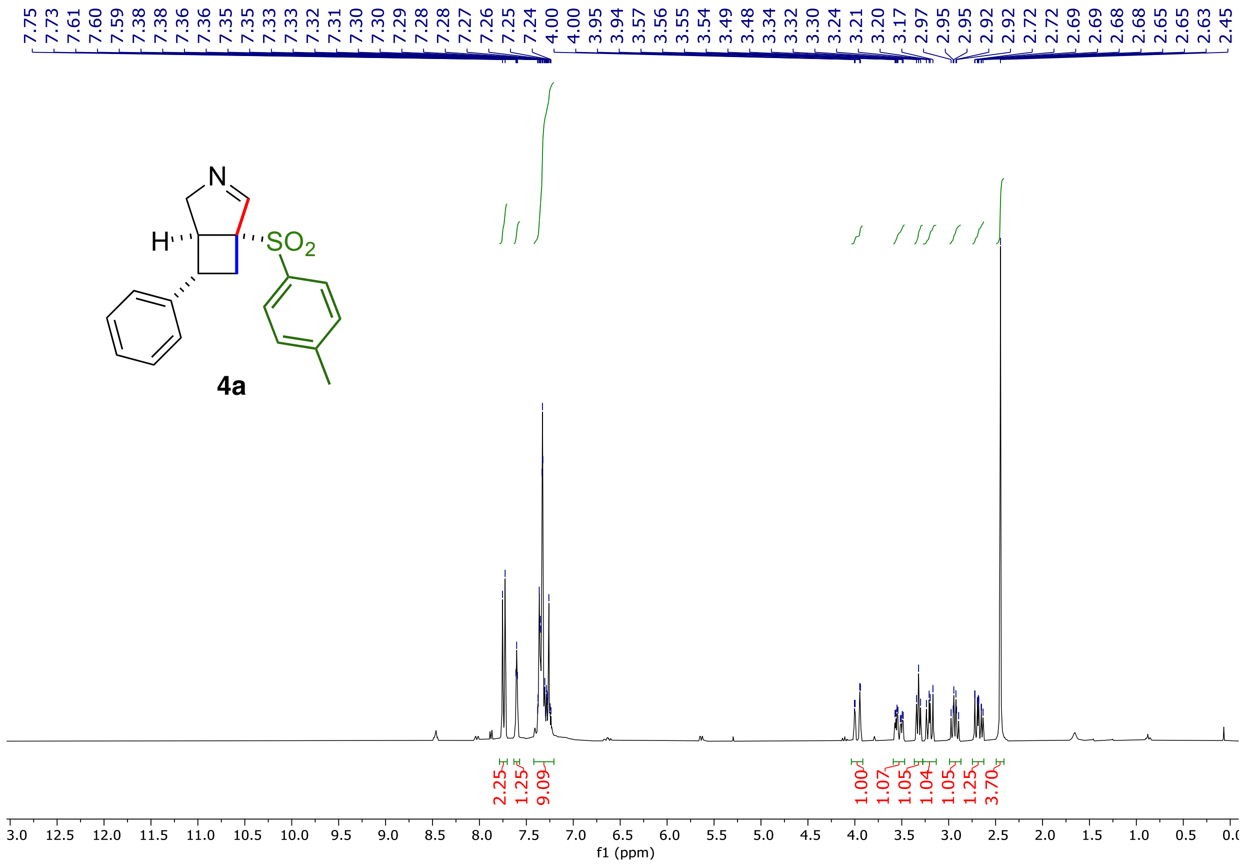

^{13}C NMR (101 MHz, $CDCl_3$)

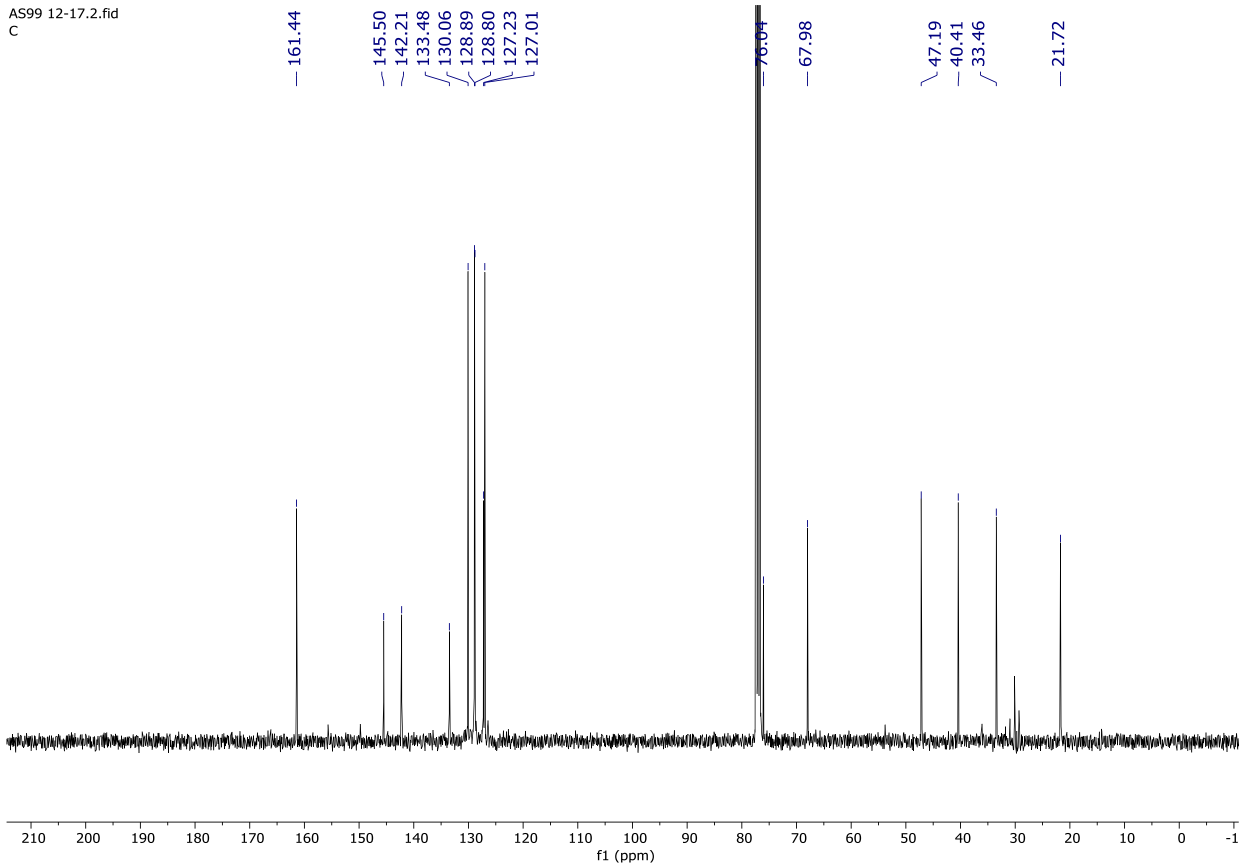

^{1}H NMR (400 MHz, $CDCl_3$)

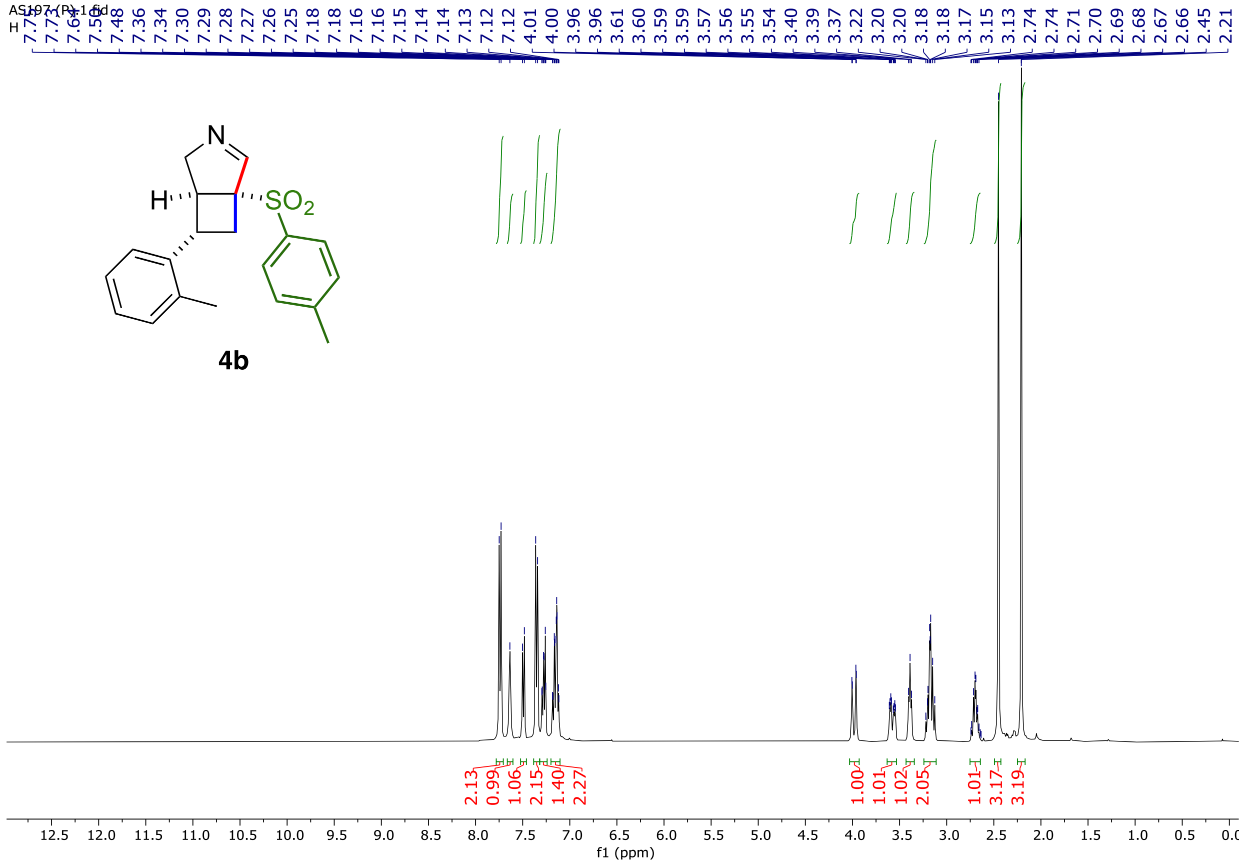

^{13}C NMR (101 MHz, $CDCl_3$)

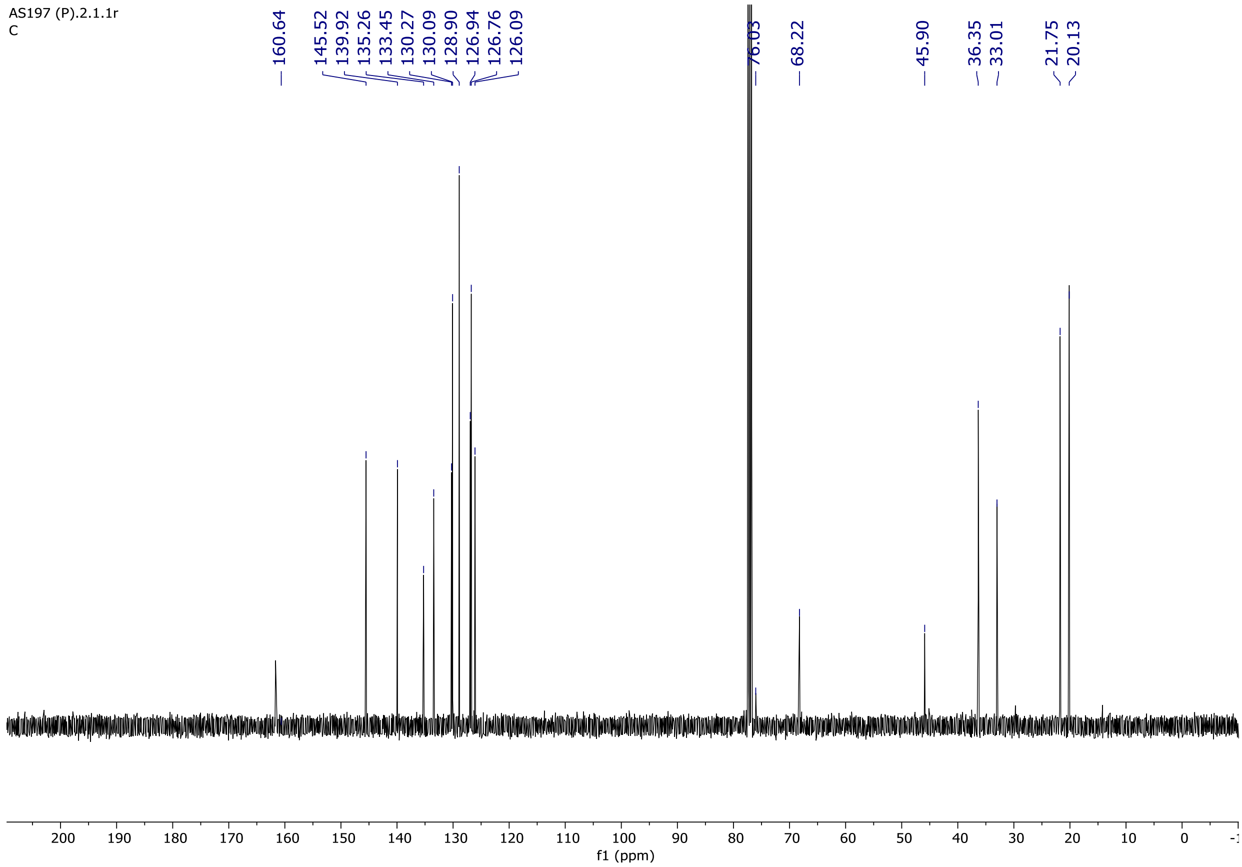

^{1}H NMR (300 MHz, $CDCl_3$)

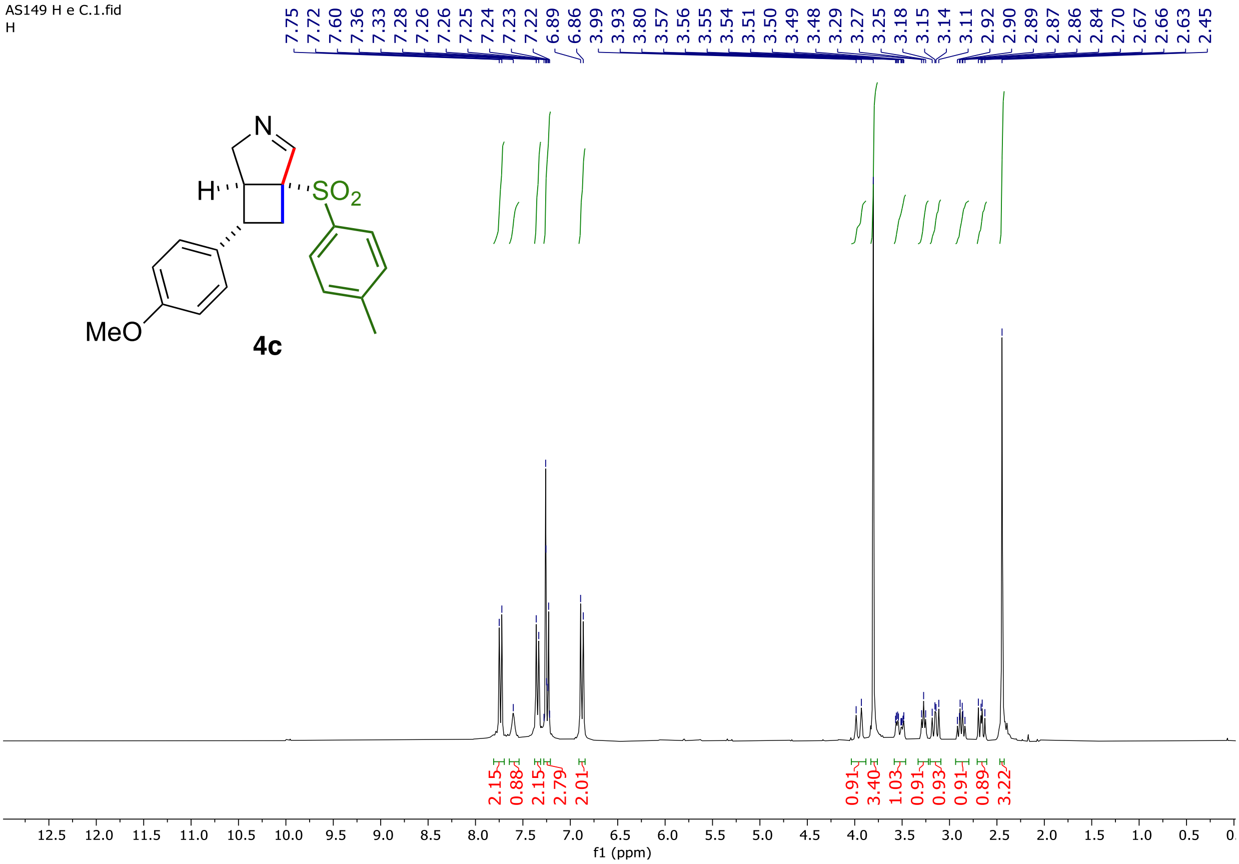

4c

^{13}C NMR (75 MHz, $CDCl_3$)

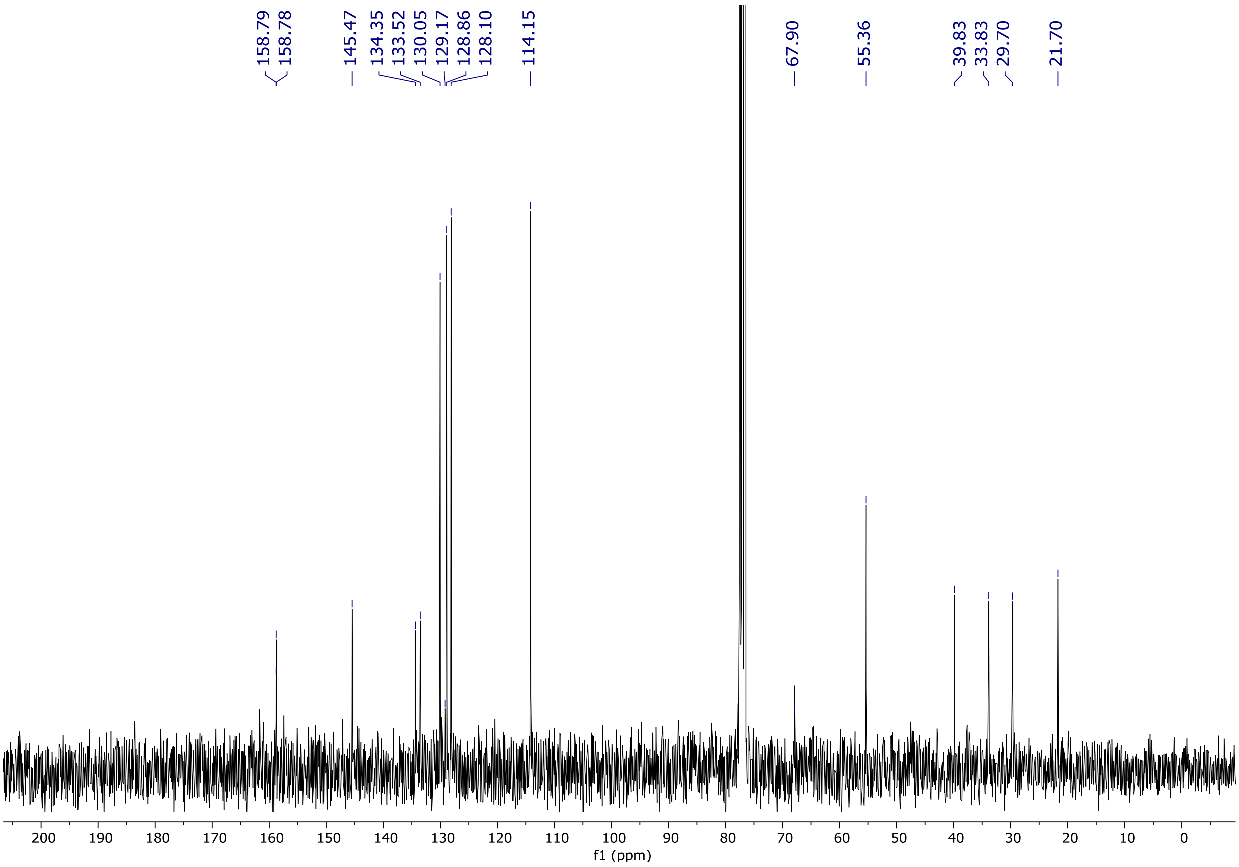

^{1}H NMR (400 MHz, $CDCl_3$)

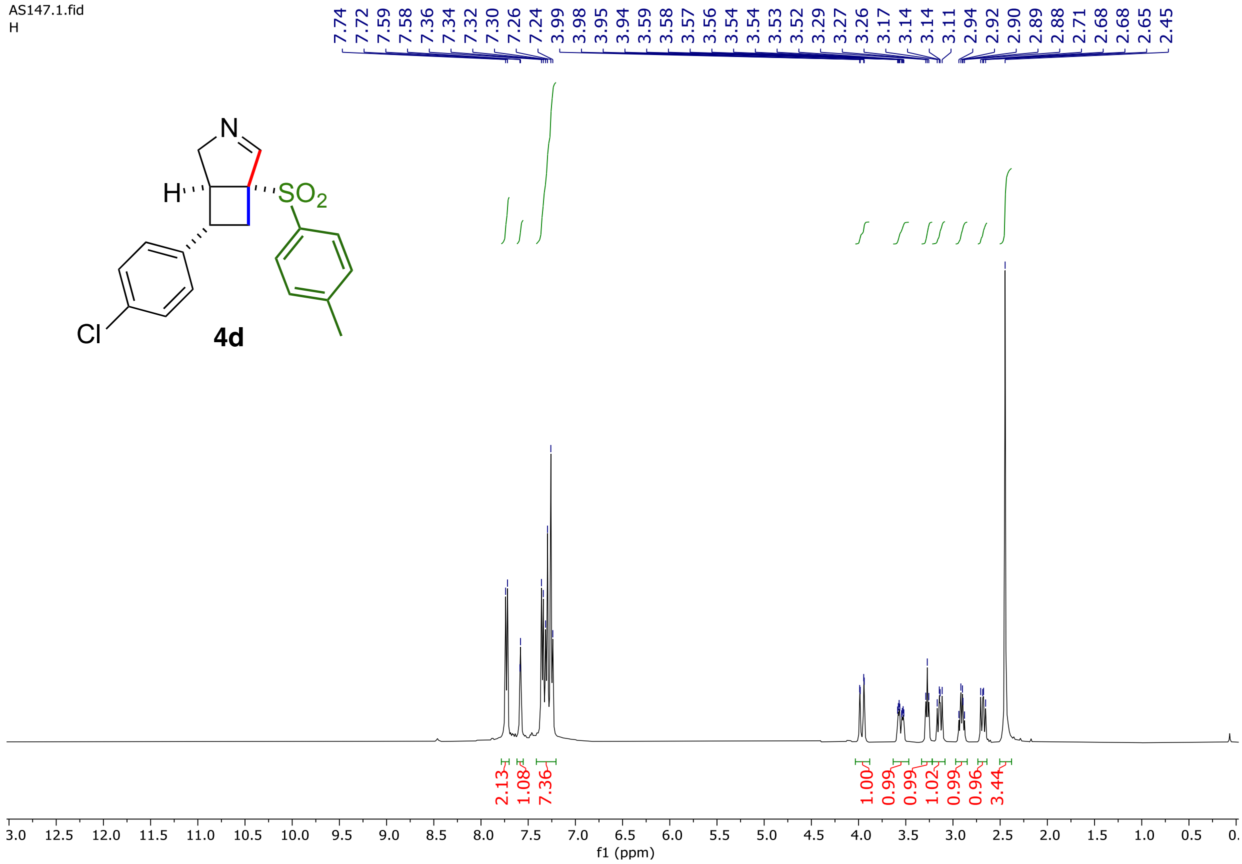

^{13}C NMR (101 MHz, $CDCl_3$)

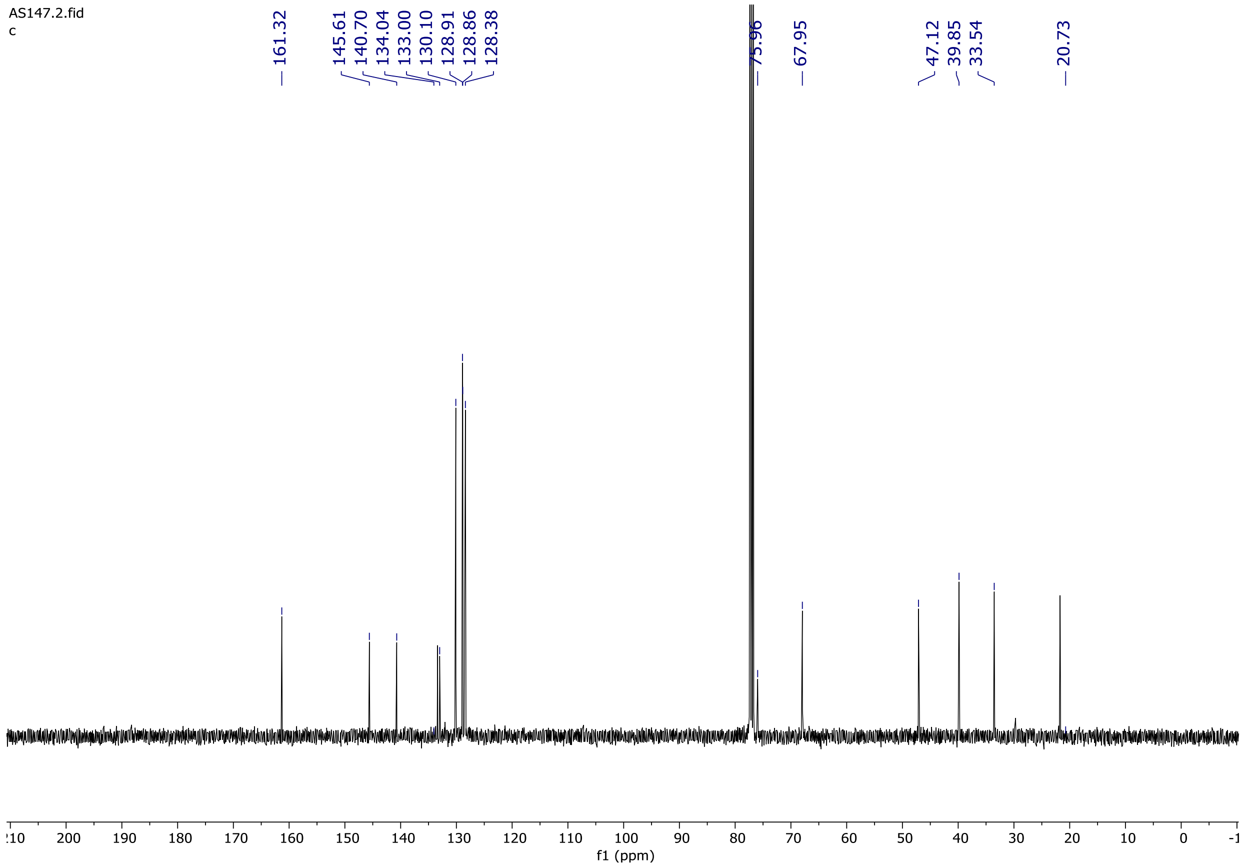

^{1}H NMR (400 MHz, $CDCl_3$)

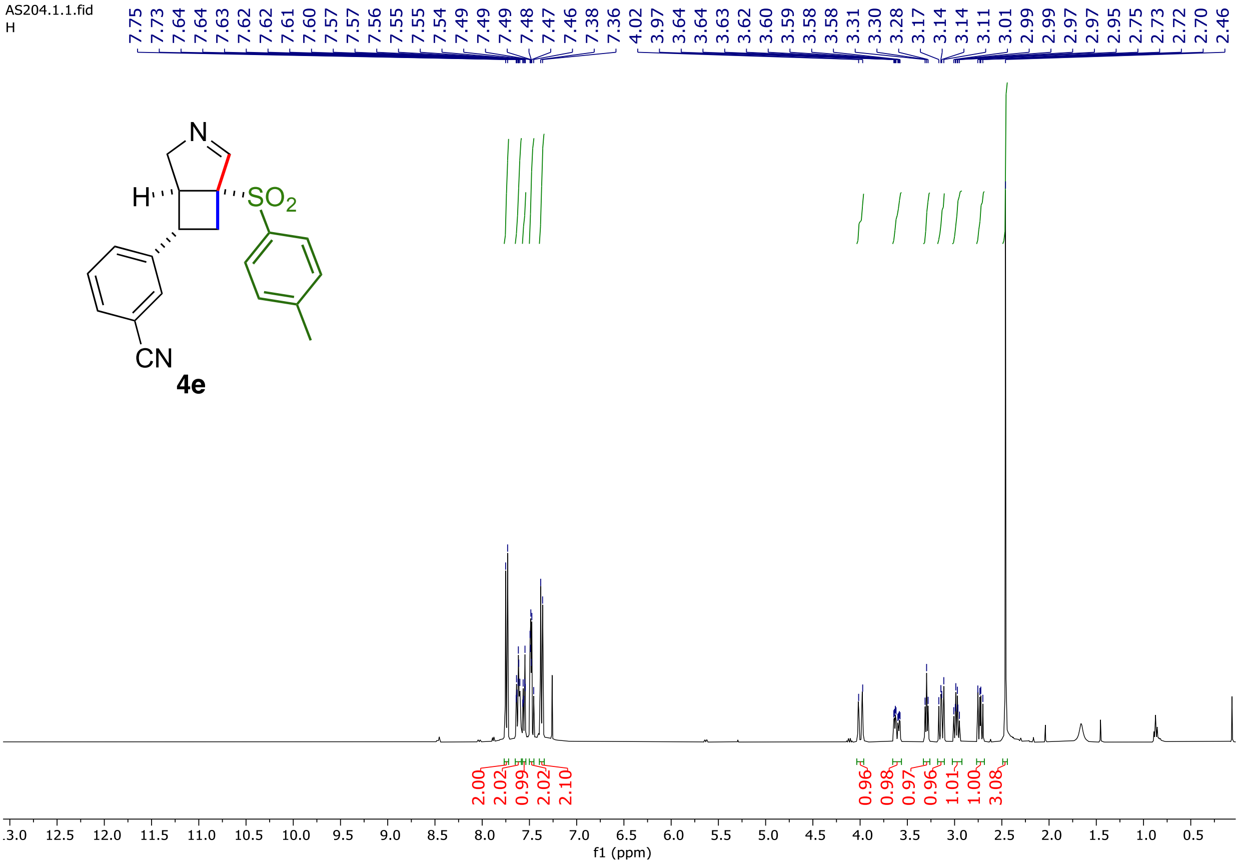

^{13}C NMR (101 MHz, $CDCl_3$)

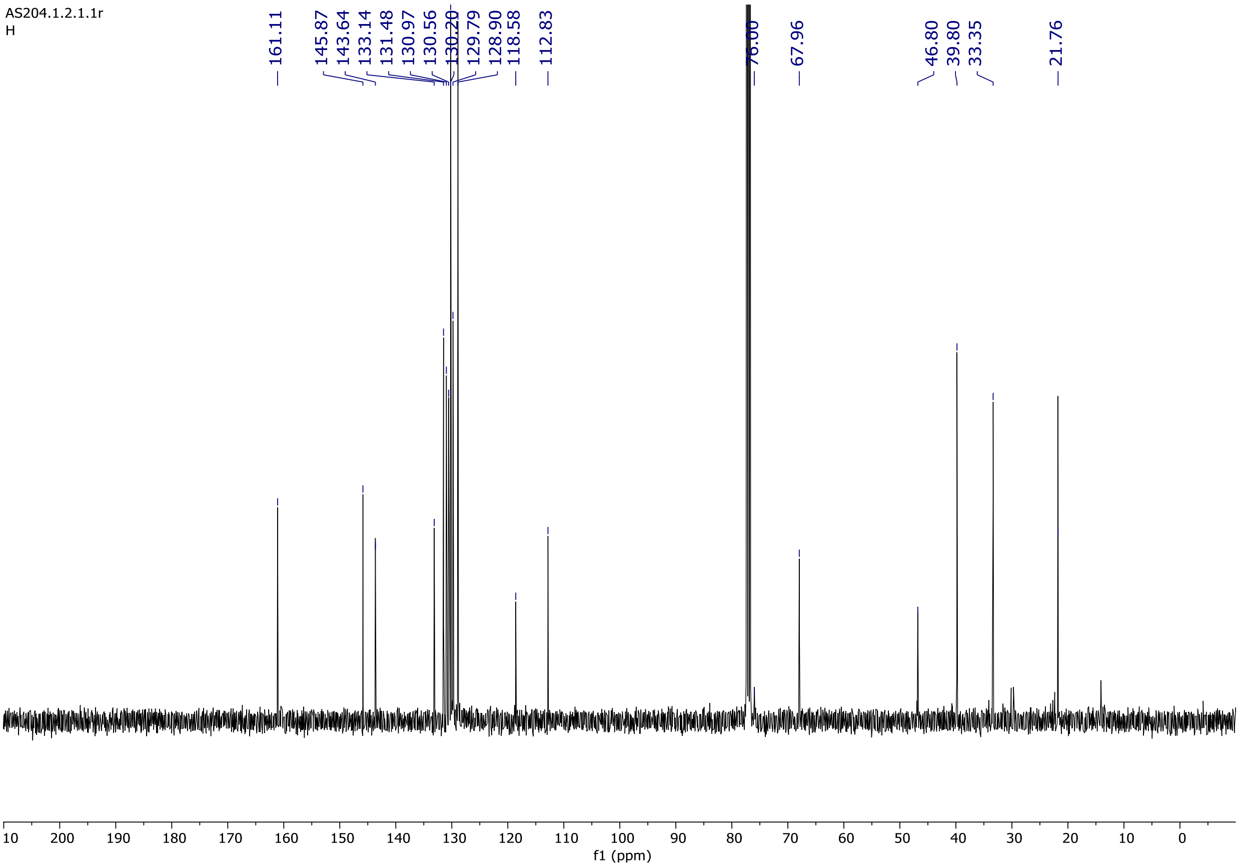

^{13}C NMR (101 MHz, CDCl$_3$)

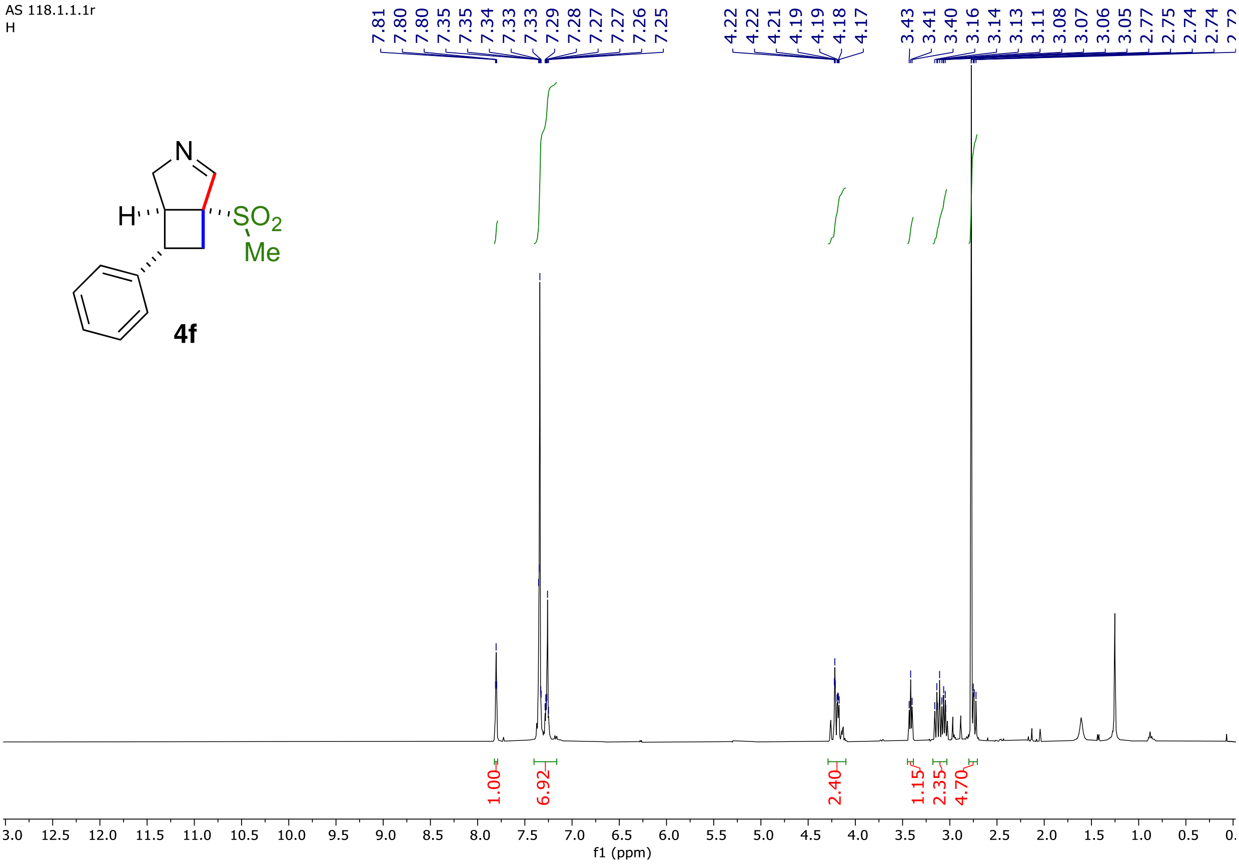

^{13}C NMR (101 MHz, CDCl$_3$)

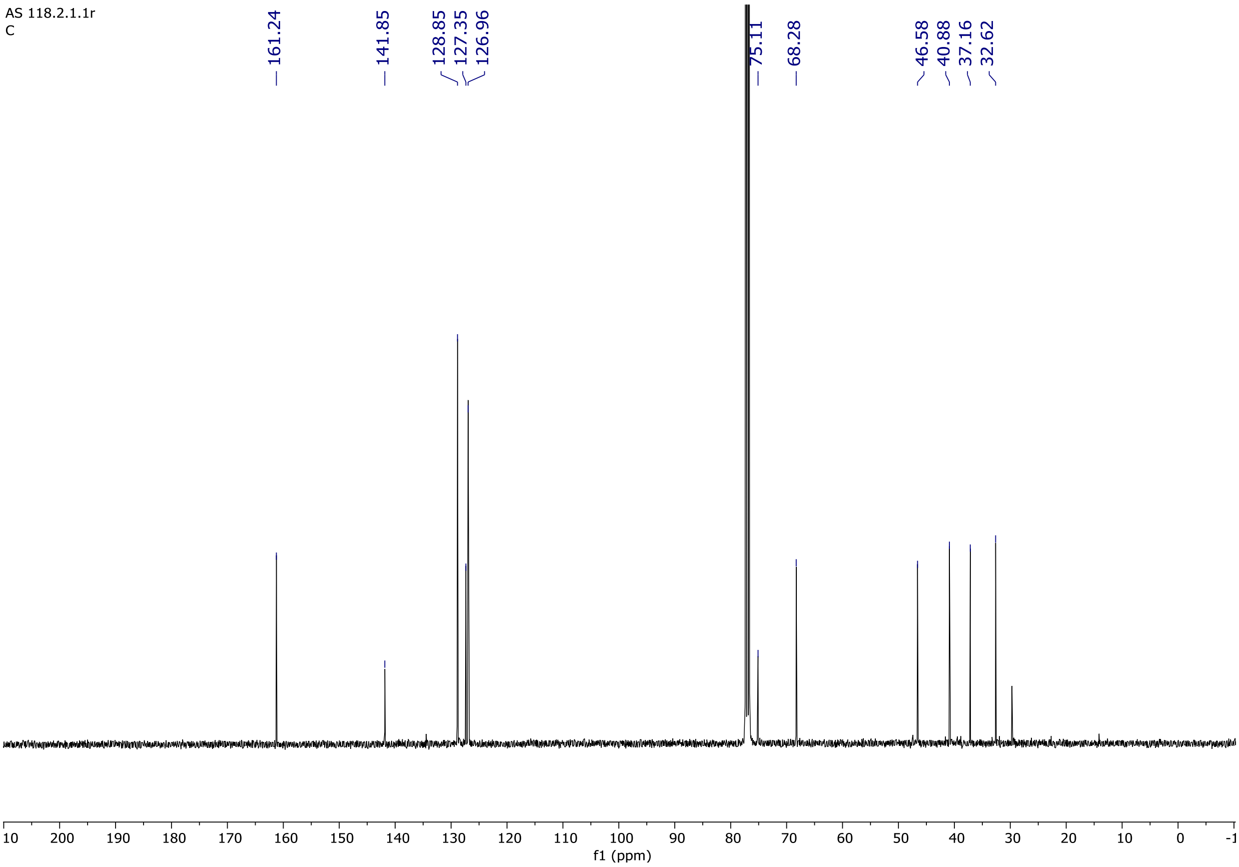

^{1}H NMR (400 MHz, $CDCl_3$)

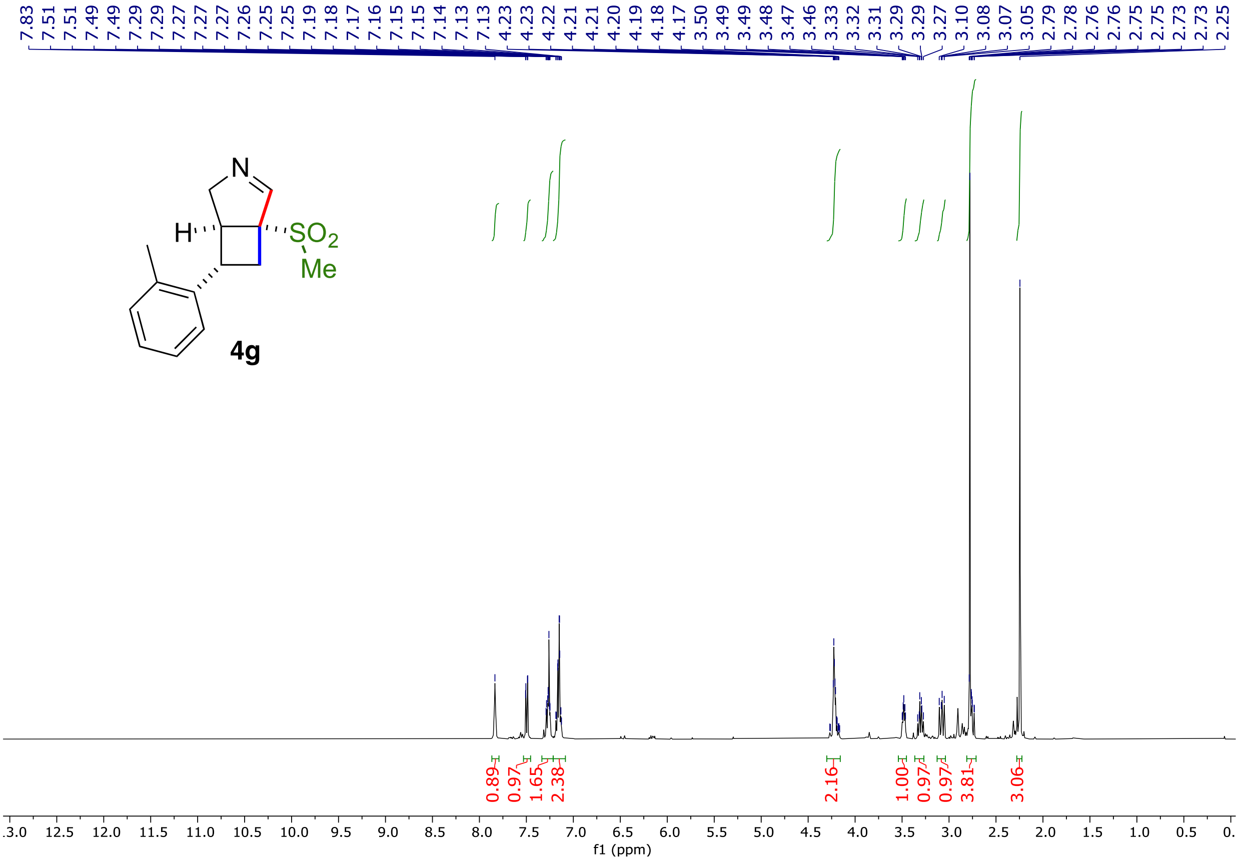

4g

^{13}C NMR (101 MHz, $CDCl_3$)

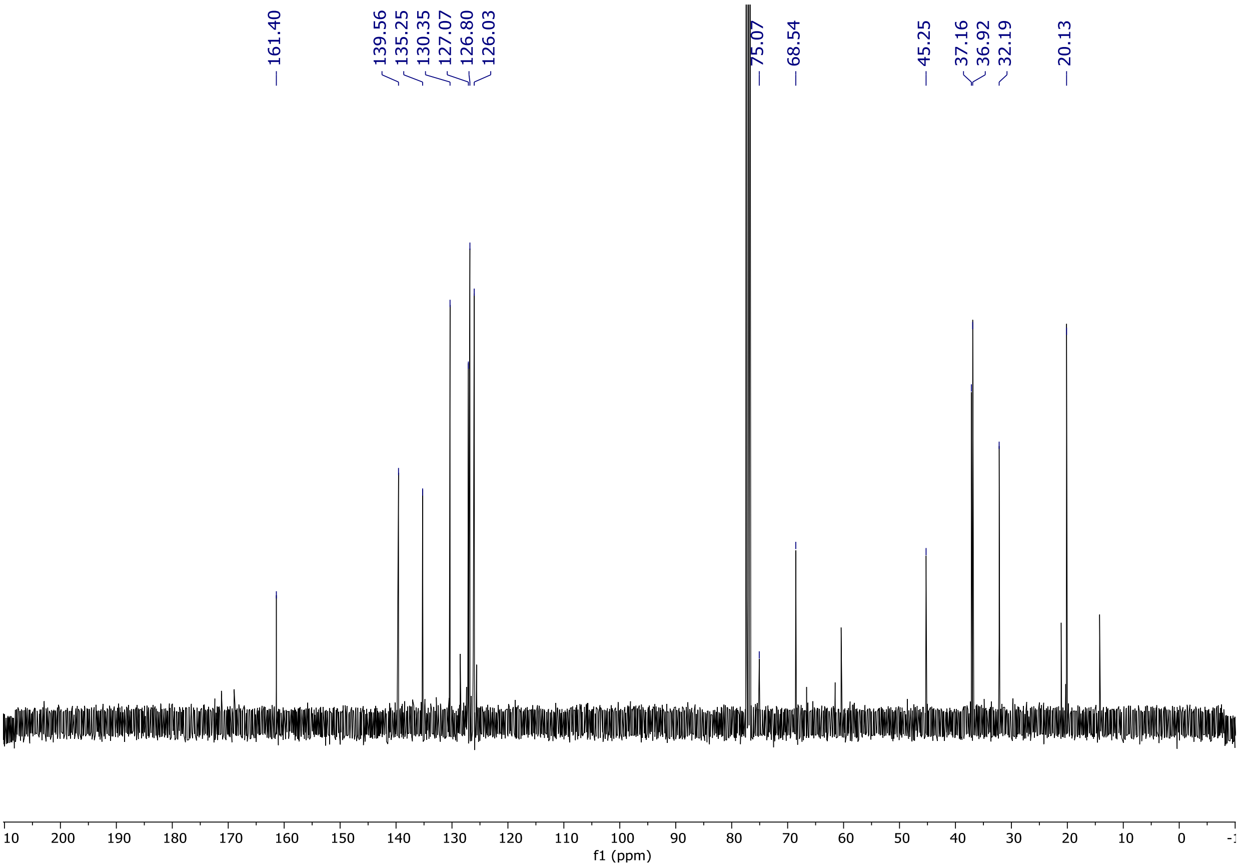

^{1}H NMR (300 MHz, $CDCl_3$)

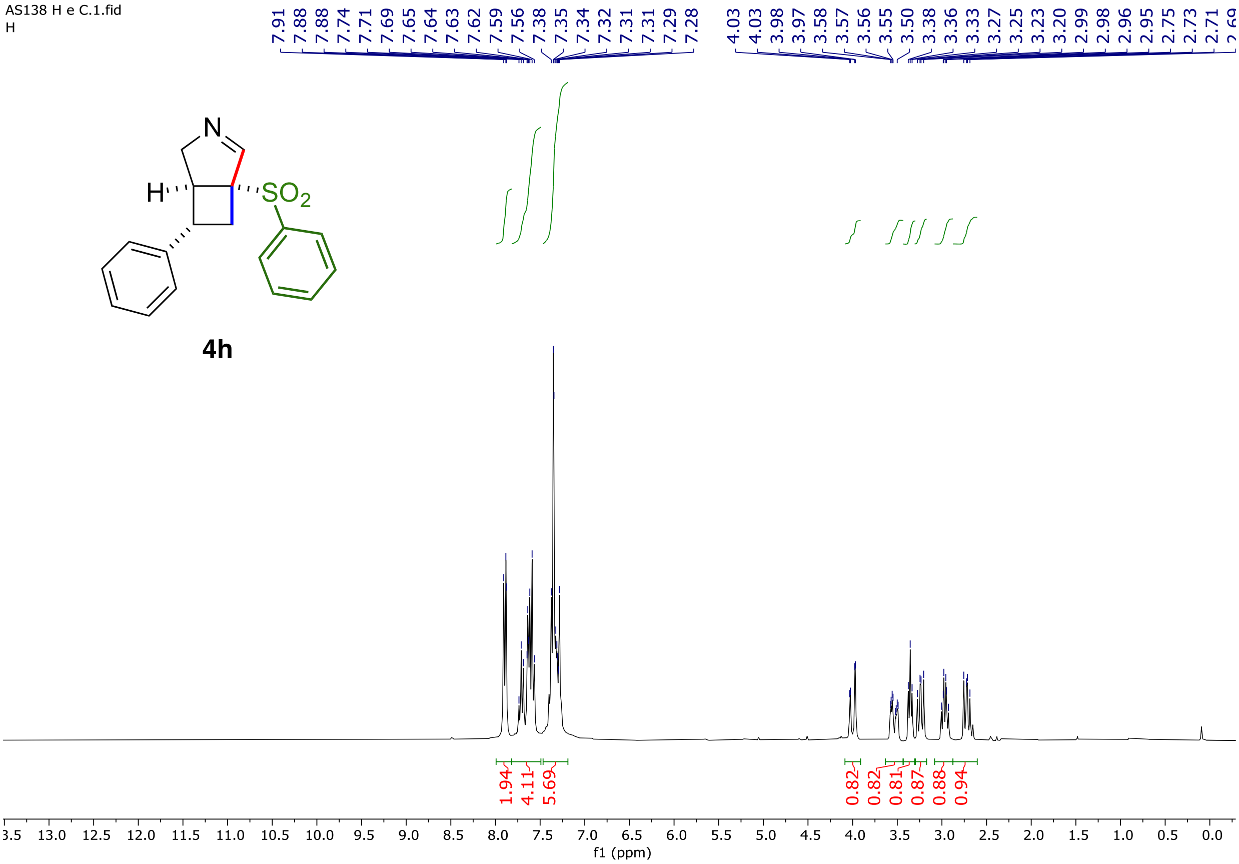

^{13}C NMR (75 MHz, $CDCl_3$)

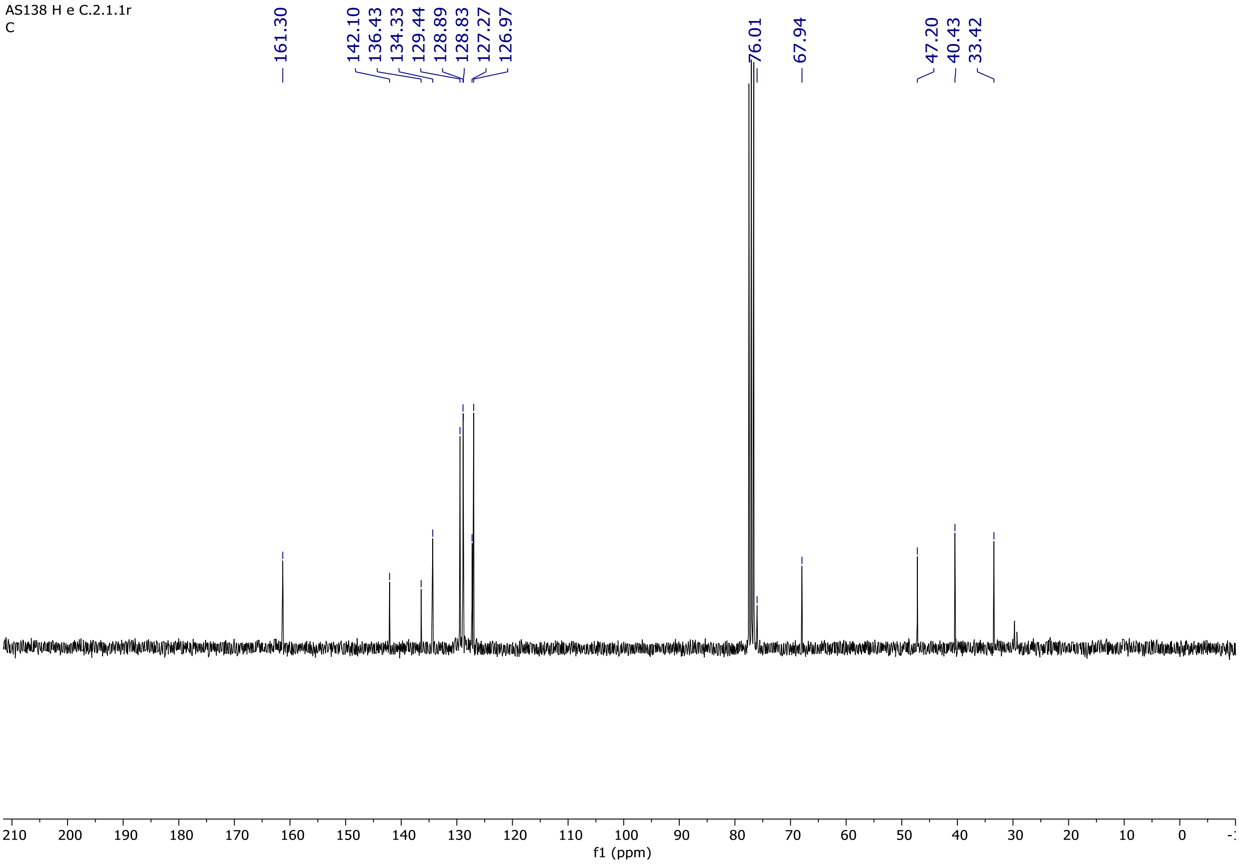

^{1}H NMR (400 MHz, $CDCl_3$)

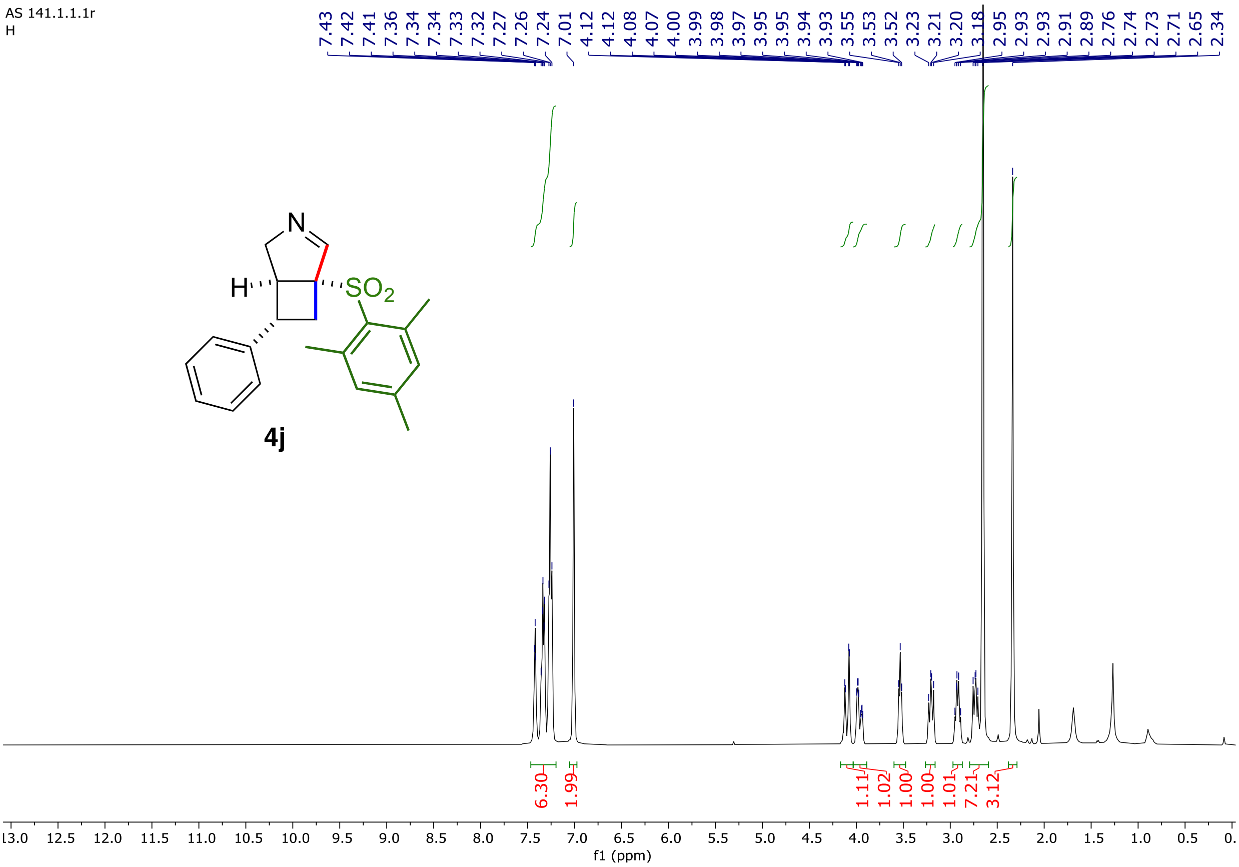

^{13}C NMR (101 MHz, $CDCl_3$)

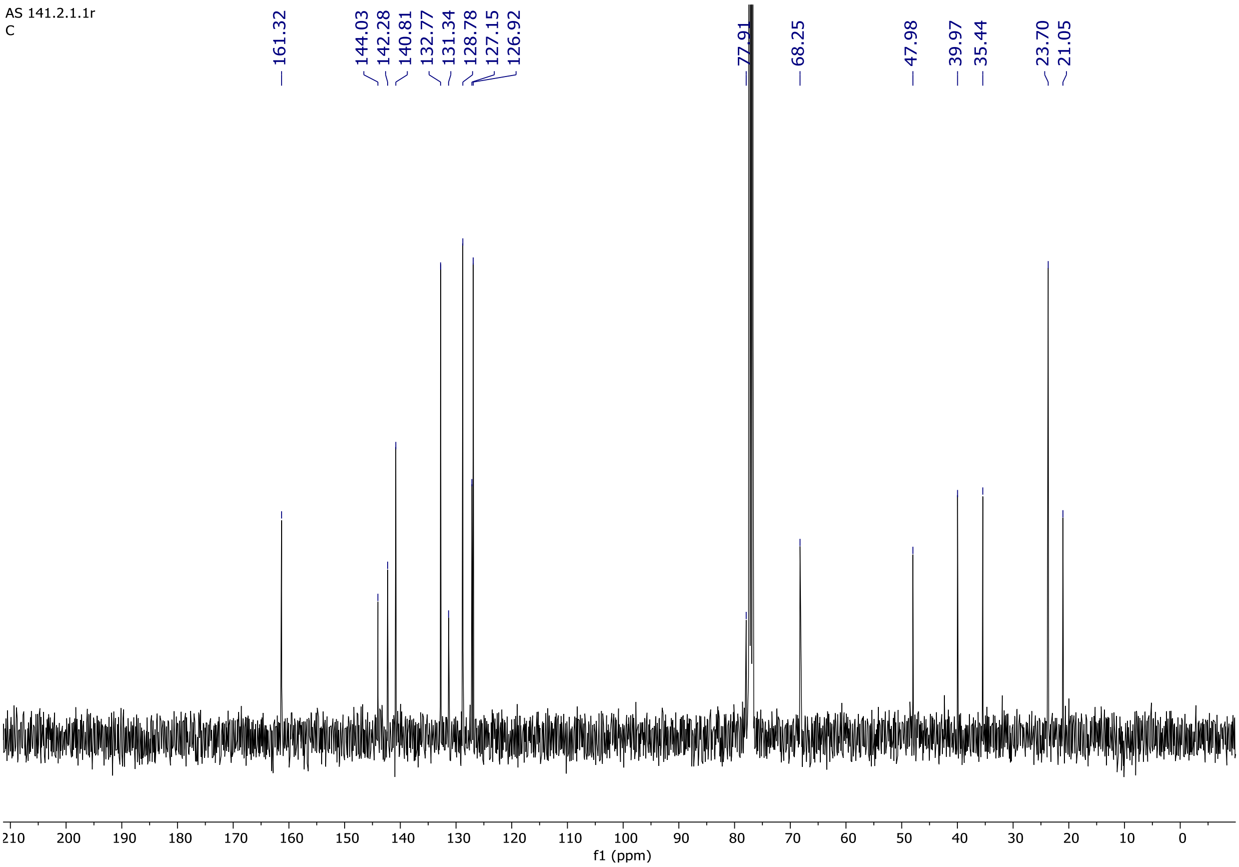

^{1}H NMR (400 MHz, $CDCl_3$)

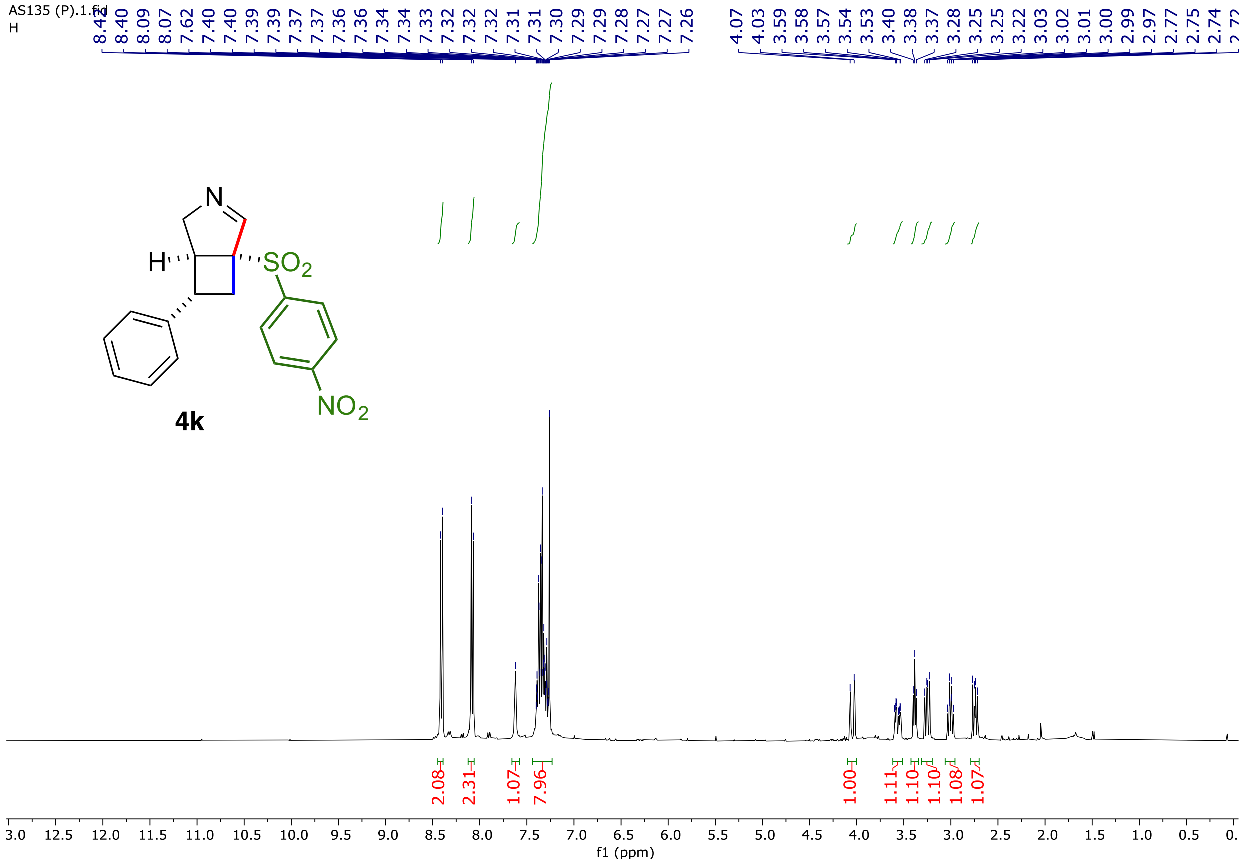

^{13}C NMR (75 MHz, acetone)

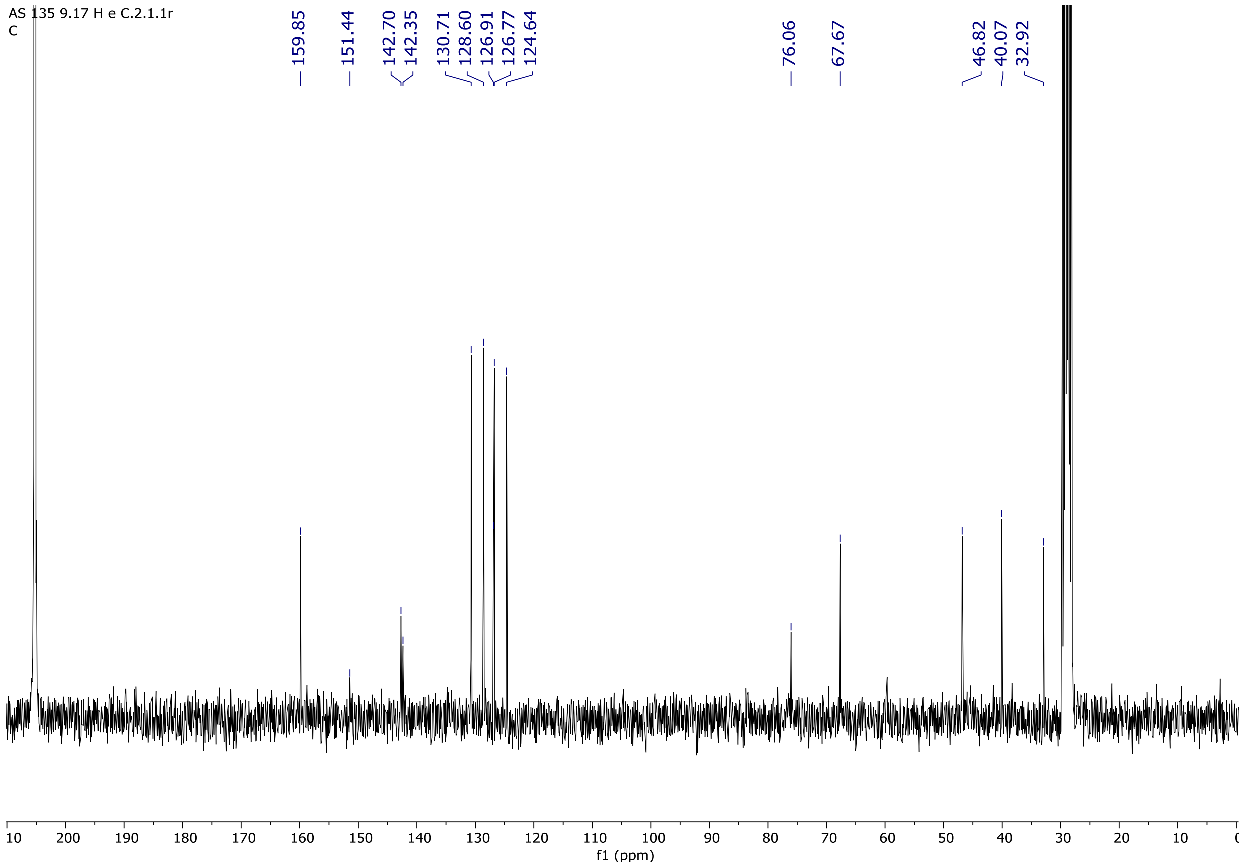

^{1}H NMR (400 MHz, $CDCl_3$)

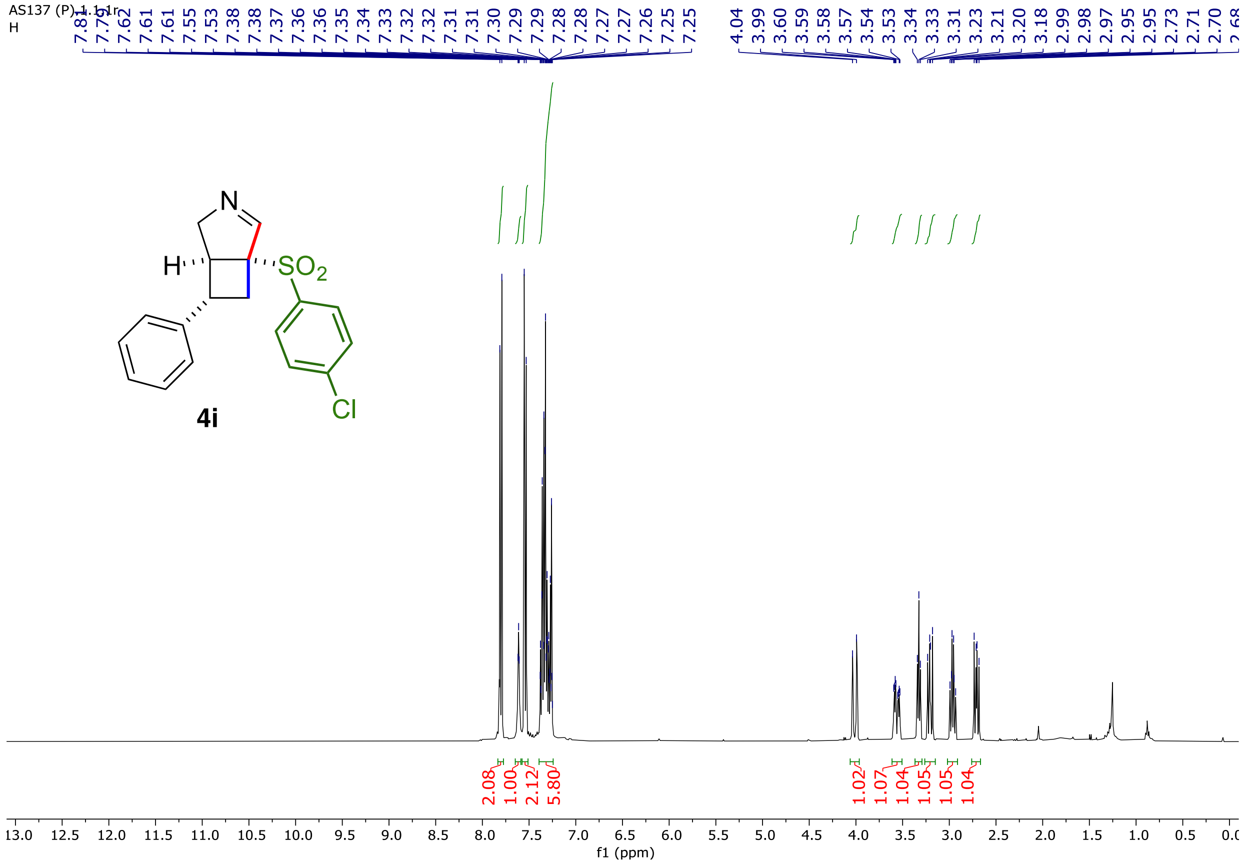

^{13}C NMR (75 MHz, $CDCl_3$)

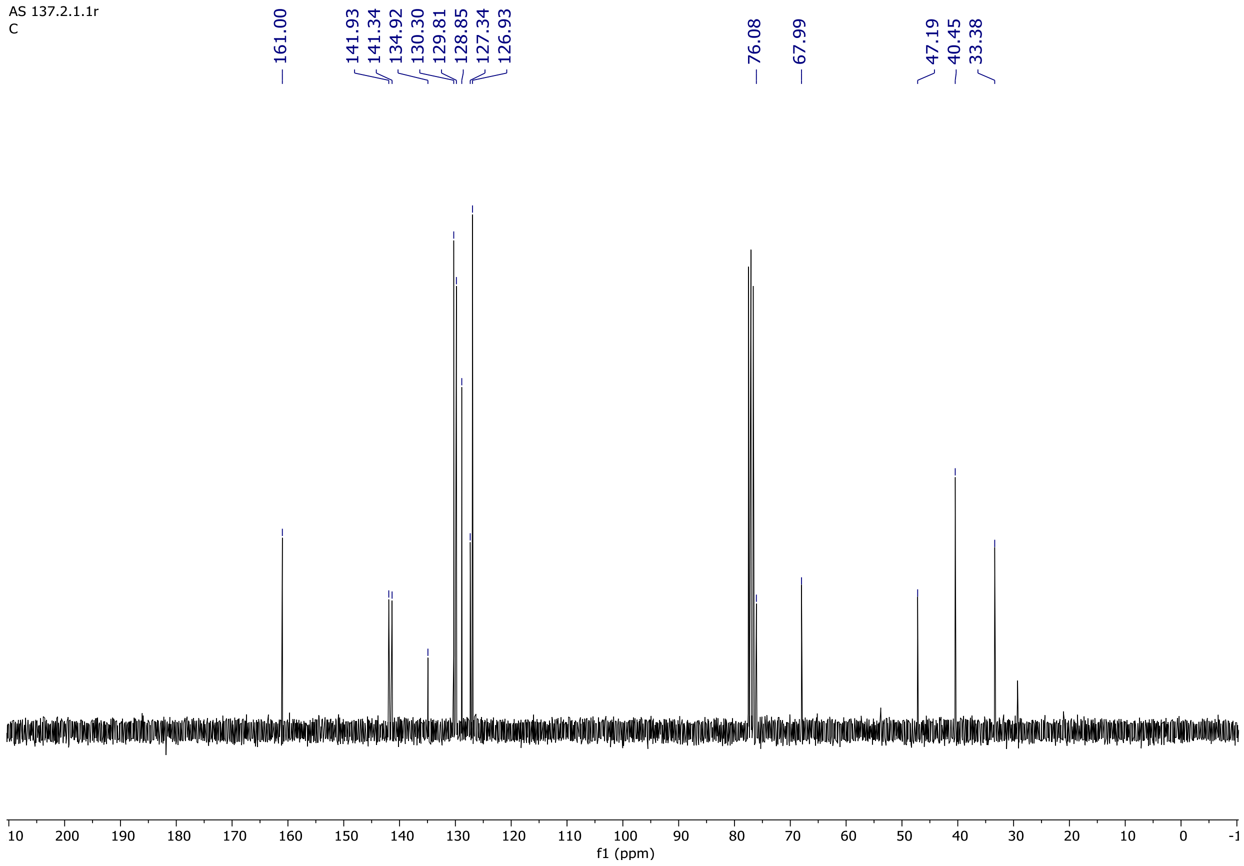

^{1}H NMR (300 MHz, $CDCl_3$)

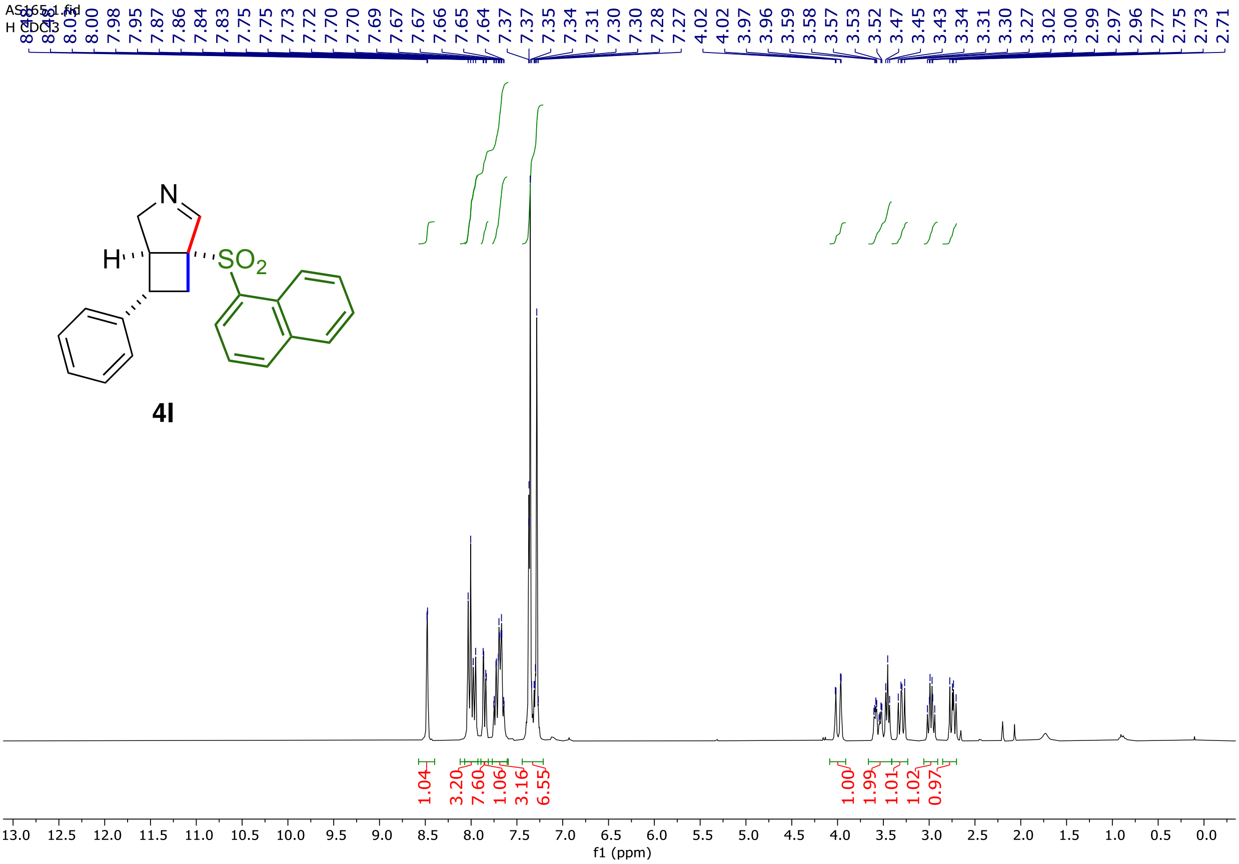

^{13}C NMR (75 MHz, $CDCl_3$)

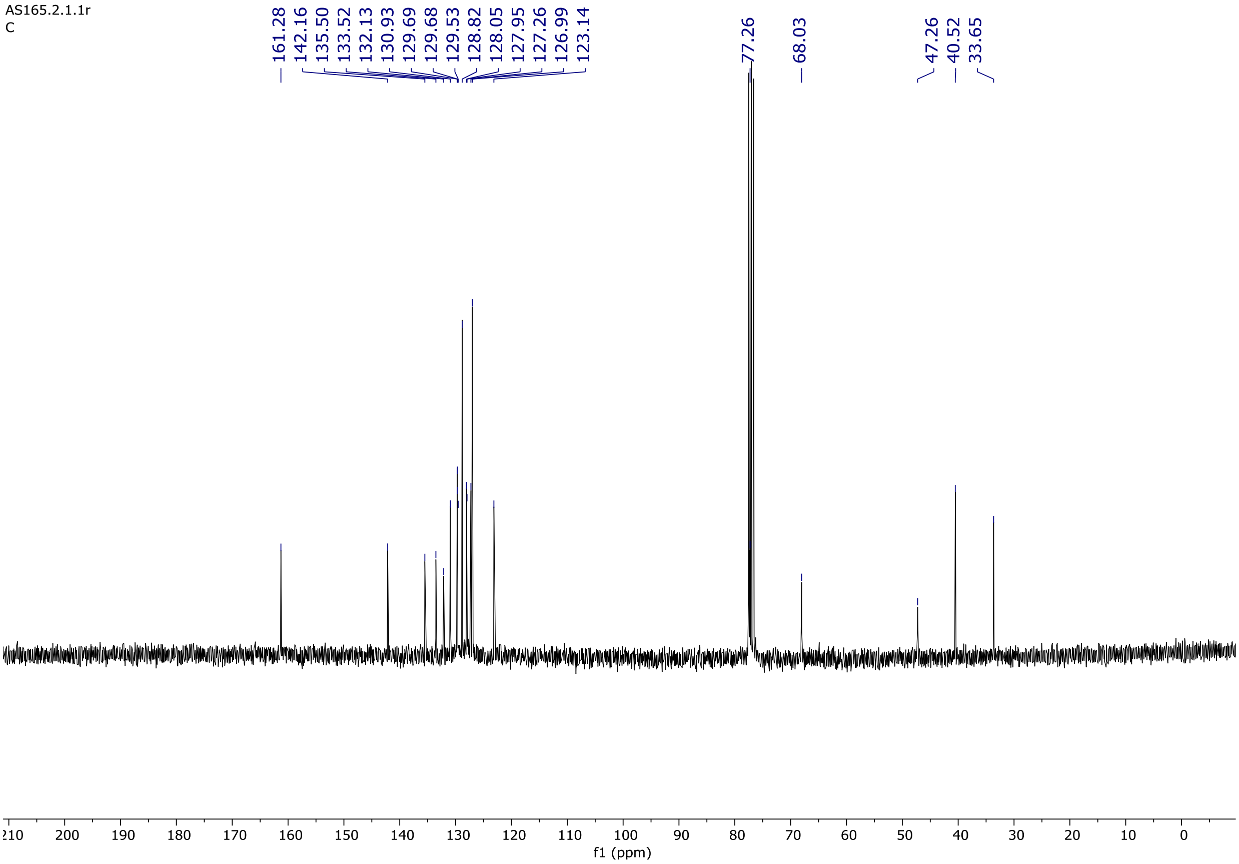

^{1}H NMR (400 MHz, $CDCl_3$)

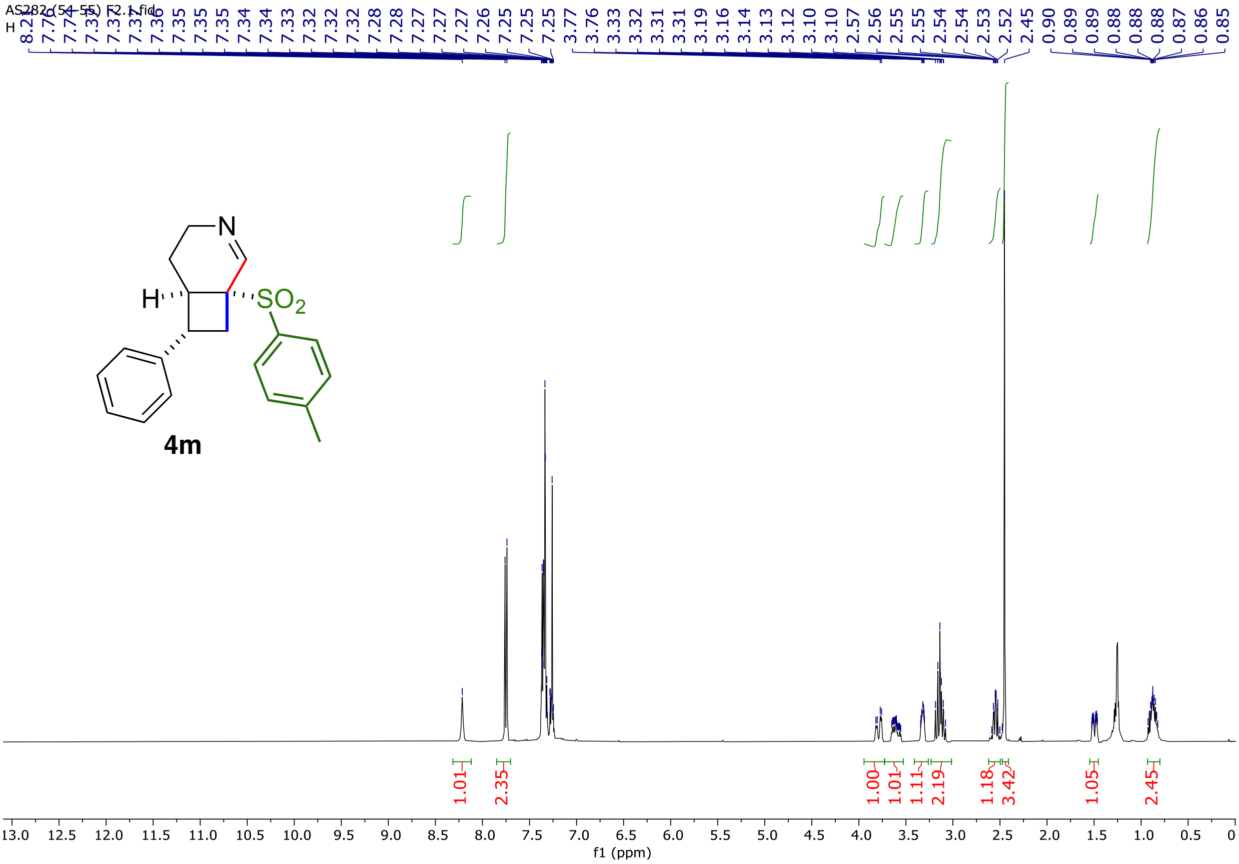

^{13}C NMR (101 MHz, $CDCl_3$)

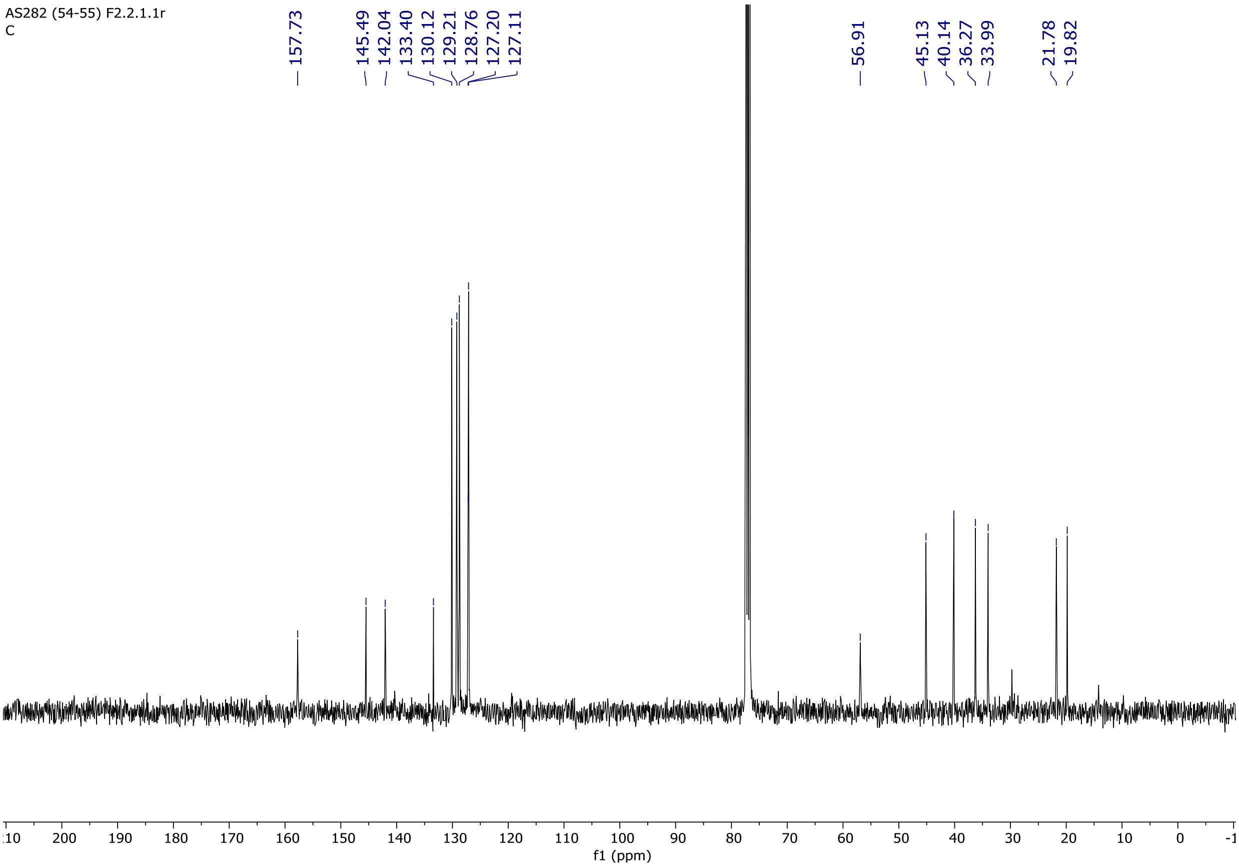

Xyz coordinates

optimized at the M06/Def2-tzvp level with DMF as implicit solvent

1

35

scf done: -1146.775960

C	8.646922	-4.891170	0.108052
C	8.872387	-3.781292	0.911007
C	7.965534	-2.740312	0.934692
C	6.809457	-2.780705	0.152718
C	6.596896	-3.903239	-0.646698
C	7.504404	-4.948094	-0.671270
C	5.820225	-1.706686	0.138601
C	5.871368	-0.552054	0.795402
C	4.794801	0.472816	0.728424
N	5.300042	1.770729	0.266302
S	4.930387	2.261069	-1.240015
O	5.695036	3.443515	-1.507423
C	5.756403	2.715200	1.285288
C	4.636625	3.374071	2.037801
C	4.318008	3.069756	3.261347
C	3.226193	2.713589	-1.218101
O	5.048949	1.125125	-2.106175
H	4.342688	0.635277	1.714684
H	3.991751	0.128284	0.072813
H	2.943782	2.977334	-2.236785
H	2.633488	1.865798	-0.876983
H	3.090074	3.566963	-0.556786
H	6.723965	-0.305540	1.425004
H	4.949727	-1.892269	-0.490726
H	5.701638	-3.949387	-1.258691
H	7.317176	-5.809676	-1.301561
H	9.359901	-5.707055	0.093443
H	9.763328	-3.730912	1.526339
H	8.156937	-1.884856	1.572945
H	6.383985	3.461102	0.798280
H	6.393669	2.157148	1.975867
H	4.069650	4.143559	1.516806
C	4.022311	2.761223	4.485600
H	4.473126	3.280904	5.324924
H	3.310502	1.974942	4.715211

^{3}1

35

scf done: -1146.695914

C	0.693938	-0.445558	1.065119
C	1.646817	0.020474	1.998919
C	1.169530	0.485066	3.245700
C	-0.175502	0.489330	3.533401
C	-1.097029	0.029934	2.596201
C	-0.650307	-0.438111	1.364792
C	3.024389	0.064770	1.700294
C	3.600237	-0.343081	0.433139
C	3.630727	0.551502	-0.758429
N	4.167706	1.890012	-0.482249
C	5.419396	1.965878	0.272168
S	3.211431	3.210568	-0.563383
O	4.067294	4.352315	-0.706388
C	2.407518	3.354649	0.997682
O	2.206697	2.957235	-1.553866
H	2.628491	0.693368	-1.178632

H	4.235109	0.093384	-1.546488
H	1.873976	4.304512	1.007939
H	1.712397	2.524328	1.117405
H	3.161139	3.336632	1.786083
H	3.687110	0.426440	2.489226
H	3.930409	-1.370324	0.292167
H	1.888113	0.848597	3.973887
H	-0.519579	0.853987	4.494541
H	-2.155957	0.036516	2.824950
H	-1.365082	-0.800438	0.634739
H	1.037014	-0.820088	0.105723
H	5.696559	3.018254	0.344785
H	5.284126	1.582453	1.290368
C	6.503765	1.194740	-0.414802
C	7.028755	0.117472	0.089662
H	6.833710	1.559425	-1.385159
C	7.536242	-0.954095	0.614935
H	8.401278	-0.912563	1.268913
H	7.114436	-1.933904	0.413342

TS (1-II)

35

scf done: -1146.690499

C	0.207718	0.207421	0.045621
C	0.227805	0.362878	1.412456
C	1.428893	0.283727	2.111594
C	2.610199	0.041784	1.416536
C	2.601844	-0.115298	0.048653
C	1.395220	-0.032838	-0.684962
C	1.334521	-0.209032	-2.078273
C	2.464976	-0.491535	-2.947029
C	2.211171	-0.374917	-4.430183
N	1.799636	-1.689472	-4.909082
S	0.397628	-1.971286	-5.671601
C	0.738733	-1.657369	-7.368876
C	2.675146	-2.708597	-4.358200
C	2.758946	-2.451430	-2.862914
C	1.754612	-3.064111	-2.091200
C	0.700574	-3.319107	-1.382169
O	-0.560913	-0.990215	-5.249559
O	0.092205	-3.364481	-5.526029
H	3.123914	-0.076704	-4.956463
H	1.432030	0.355115	-4.657623
H	-0.184283	-1.791377	-7.931682
H	1.092212	-0.630957	-7.463912
H	1.499997	-2.357147	-7.709023
H	3.417495	-0.074907	-2.624133
H	0.349644	-0.184250	-2.542114
H	-0.730269	0.269418	-0.497870
H	-0.697562	0.547651	1.946273
H	1.444061	0.405138	3.188009
H	3.547643	-0.027485	1.956896
H	3.533286	-0.312562	-0.470726
H	3.668497	-2.610637	-4.808526
H	2.285211	-3.698671	-4.588063
H	3.760069	-2.544180	-2.442046
H	0.425948	-4.337785	-1.102552
H	0.027601	-2.540592	-1.009330

^{3}II

35

scf done: -1146.721976

C	0.000000	0.000000	0.000000

C	0.000000	0.000000	1.552151
C	1.493934	0.000000	1.841920
N	2.044117	0.860602	0.780400
C	1.088770	1.025926	-0.322296
C	-0.681856	1.185584	2.135069
C	-2.042366	1.251295	2.482729
C	-2.558302	2.426394	3.079572
C	-3.881887	2.523519	3.441686
C	-4.750603	1.458622	3.223894
C	-4.270096	0.292918	2.634503
C	-2.947691	0.184359	2.268531
S	3.638987	0.824460	0.468057
O	3.920256	1.889050	-0.448340
C	-1.288492	0.276039	-0.620527
C	-1.819900	1.017705	-1.548901
C	3.938107	-0.698252	-0.368775
O	4.311241	0.769843	1.732058
H	1.896194	-1.018212	1.779023
H	1.734284	0.403290	2.825976
H	5.003891	-0.750560	-0.587141
H	3.651252	-1.526536	0.277966
H	3.368766	-0.717407	-1.297434
H	-0.457238	-0.923492	1.916615
H	-0.076342	2.071671	2.315687
H	-1.882349	3.257805	3.252245
H	-4.248751	3.435484	3.898863
H	-5.792995	1.536256	3.508772
H	-4.943810	-0.537884	2.458097
H	-2.598155	-0.728078	1.796698
H	1.550913	0.854231	-1.296332
H	0.678382	2.040833	-0.319920
H	0.354389	-0.990611	-0.317258
H	-2.885355	1.008935	-1.764616
H	-1.215908	1.687196	-2.168250

TS (1-II')

35

scf done: -1146.678467

C	0.045452	0.130642	0.336764
C	0.003330	0.049081	1.711334
C	1.192384	0.033683	2.474323
C	2.417896	0.121946	1.775102
C	2.448443	0.200451	0.401970
C	1.264105	0.201762	-0.329731
C	1.186235	-0.101633	3.878933
C	-0.018392	-0.298427	4.679857
C	-0.696525	-1.632891	4.701781
N	0.185213	-2.724679	5.130754
S	0.474568	-3.974251	4.126677
O	-0.749731	-4.288505	3.450032
C	1.163572	-2.444609	6.200156
C	0.606976	-1.414110	7.123926
C	0.677426	-0.124087	6.804375
C	0.945908	1.148677	6.959201
O	1.135187	-4.986569	4.897779
C	1.616139	-3.403692	2.910228
H	-1.086863	-1.892499	3.711230
H	-1.561581	-1.586690	5.370254
H	1.893870	-4.260619	2.297154
H	1.135294	-2.641282	2.297044
H	2.497560	-2.996190	3.406712
H	2.151681	-0.052984	4.382194
H	-0.708436	0.540443	4.774680

H	3.341688	0.113486	2.345360
H	3.400901	0.260548	-0.112260
H	1.291613	0.260630	-1.411048
H	-0.879425	0.139892	-0.228565
H	-0.953805	0.001522	2.220008
H	1.364720	-3.374684	6.728856
H	2.109640	-2.088839	5.769198
H	-0.033723	-1.738543	7.939399
H	1.665943	1.466589	7.707160
H	0.495519	1.921259	6.347109

^{3}II’

35

scf done: -1146.761413

C	0.164627	1.662493	0.688619
C	0.052808	0.957120	1.866063
C	1.042041	0.020660	2.251056
C	2.141792	-0.151695	1.373371
C	2.241367	0.556852	0.199932
C	1.253901	1.472486	-0.155124
C	0.988497	-0.731495	3.435609
C	-0.060557	-0.682791	4.488268
C	-0.668351	-2.052171	4.767496
N	0.320496	-2.950944	5.341301
S	0.587781	-4.433337	4.733507
O	-0.595611	-4.856425	4.044387
C	1.062181	-2.511616	6.518430
C	1.082213	-1.037623	6.663997
C	0.563273	-0.136154	5.756430
C	0.628104	1.227825	5.942944
O	1.122026	-5.239952	5.791557
C	1.854829	-4.234220	3.526712
H	-1.052204	-2.496288	3.849173
H	-1.511087	-1.939423	5.460389
H	2.084273	-5.218118	3.118901
H	1.489823	-3.574023	2.739417
H	2.737484	-3.811899	4.007298
H	1.843753	-1.368835	3.651249
H	-0.878765	-0.022346	4.189163
H	2.913093	-0.865281	1.647296
H	3.094269	0.401894	-0.450939
H	1.333688	2.032702	-1.078936
H	-0.606522	2.375303	0.419193
H	-0.806086	1.127400	2.504412
H	0.644363	-2.966771	7.427911
H	2.092412	-2.887467	6.455178
H	1.547822	-0.663793	7.570786
H	1.098103	1.650514	6.823506
H	0.216943	1.912242	5.209390

2

35

scf done: -1146.816233

C	1.634281	-4.107607	23.097796
C	1.468107	-3.164085	24.278012
C	2.816536	-2.391700	24.383750
C	3.657315	-2.875177	23.224925
N	3.093201	-4.206588	22.964190
C	1.744133	-3.750789	25.639009
C	1.038997	-4.500924	26.462531
C	3.104406	-3.089630	25.746157
C	3.368388	-2.218608	26.932889
C	2.440338	-1.258873	27.331832

C	2.689719	-0.439051	28.416912
C	3.874025	-0.565493	29.128654
C	4.802774	-1.517084	28.744689
C	4.549378	-2.335980	27.654944
S	3.731471	-5.136070	21.786649
O	5.153550	-5.118581	21.959031
C	3.360456	-4.326587	20.267091
O	3.028832	-6.384316	21.805957
H	2.755352	-1.306064	24.441860
H	3.528602	-2.215281	22.355129
H	4.720667	-2.952532	23.453026
H	0.552677	-2.576124	24.200705
H	3.916030	-3.819607	25.642956
H	1.198682	-5.092641	23.264250
H	1.184160	-3.667573	22.196543
H	0.049767	-4.858902	26.197171
H	1.423122	-4.780372	27.438427
H	5.280139	-3.081149	27.356699
H	1.507864	-1.157202	26.783590
H	5.730198	-1.625657	29.295377
H	4.069056	0.075132	29.980668
H	1.956079	0.302360	28.712158
H	3.773103	-4.936148	19.464486
H	2.279953	-4.247698	20.154879
H	3.824736	-3.341356	20.260769

2’

35

scf done: -1146.810608

C	0.038627	2.236763	1.028448
C	0.256857	1.577390	2.225225
C	1.008204	0.405942	2.268121
C	1.530131	-0.086326	1.077004
C	1.312503	0.569407	-0.123961
C	0.566004	1.734910	-0.151702
C	1.226132	-0.336088	3.543909
C	-0.007944	-0.909997	4.303442
C	-0.509292	-2.338264	4.141308
N	-0.033009	-3.224681	5.207292
S	0.981693	-4.456377	4.887998
O	0.716109	-4.893702	3.548449
C	-0.032620	-2.645748	6.560986
C	0.821939	-1.409715	6.603799
C	0.754325	-0.609803	5.555364
C	1.612360	0.402593	4.859843
O	0.876014	-5.395995	5.966514
C	2.623741	-3.816062	4.929722
H	-0.212092	-2.756145	3.178188
H	-1.602867	-2.353935	4.173597
H	3.304661	-4.642336	4.727880
H	2.726990	-3.046848	4.165677
H	2.818717	-3.400825	5.917762
H	1.941141	-1.148112	3.375537
H	-0.836360	-0.204303	4.175229
H	2.118670	-0.998574	1.095088
H	1.729155	0.168070	-1.040746
H	0.394989	2.252076	-1.088650
H	-0.547909	3.148355	1.015999
H	-0.165611	1.983088	3.139526
H	-1.077117	-2.437935	6.821144
H	0.316888	-3.403907	7.260443
H	1.523581	-1.264785	7.418133
H	2.667674	0.459673	5.126063

H	1.180494	1.407016	4.885308

3

32
scf done: -1107.482285

C	1.121650	-0.252960	2.153346
C	2.324264	-0.337037	1.328678
C	2.488491	0.150199	0.101951
C	3.753709	0.037124	-0.677784
N	4.508422	1.298437	-0.777752
S	3.936981	2.520775	-1.727053
C	2.900808	3.495707	-0.688324
C	5.395464	1.653561	0.258610
C	5.672042	0.927086	1.305585
O	3.132687	1.905201	-2.737348
O	5.053709	3.336239	-2.096301
H	3.557168	-0.302551	-1.694645
H	4.434317	-0.680111	-0.214029
H	2.555200	4.342262	-1.280573
H	2.056510	2.896789	-0.351598
H	3.483453	3.848046	0.162103
H	5.896013	2.606018	0.111081
H	3.157844	-0.873473	1.781373
C	6.005867	0.229016	2.346451
H	6.819552	-0.491022	2.317027
H	5.482938	0.329405	3.294366
H	1.666650	0.656340	-0.400420
C	1.051440	-1.007967	3.323021
C	-0.070531	-0.969554	4.132581
C	-1.144648	-0.166487	3.789801
C	-1.085490	0.600177	2.634213
C	0.033780	0.559027	1.826682
H	1.893719	-1.636745	3.593637
H	-0.104455	-1.568385	5.035265
H	-2.023920	-0.131296	4.422122
H	-1.918663	1.239019	2.364977
H	0.066188	1.175338	0.934604

^{3}3

32
scf done: -1107.401767

C	1.426010	-0.023837	2.038877
C	2.755814	-0.335832	1.694543
C	3.172139	-0.810063	0.390230
C	3.447824	0.094688	-0.762208
N	4.054792	1.369921	-0.385123
S	3.254003	2.802344	-0.548788
C	2.452572	3.092791	0.989607
C	5.274881	1.360953	0.314618
C	5.927483	0.286530	0.663841
O	2.253060	2.609845	-1.552969
O	4.245952	3.821499	-0.714562
H	2.535873	0.333128	-1.320704
H	4.122191	-0.402229	-1.468882
H	2.077420	4.115481	0.969019
H	1.631438	2.386201	1.104195
H	3.179686	2.971180	1.793363
H	5.679990	2.343182	0.534860
H	3.515652	-0.199516	2.467279
C	6.612791	-0.749462	1.038633
H	7.362304	-1.207592	0.398958
H	6.459293	-1.210448	2.011850
H	3.243932	-1.877806	0.195674

C	1.117297	0.486619	3.320739
C	-0.166038	0.861799	3.643714
C	-1.191837	0.739537	2.709880
C	-0.914674	0.226086	1.446431
C	0.366364	-0.152793	1.111733
H	1.919186	0.591155	4.044955
H	-0.379170	1.257577	4.630165
H	-2.200582	1.040374	2.966095
H	-1.712301	0.123350	0.719558
H	0.577816	-0.556481	0.126067

^{2}Ms

7
scf done: -588.481609

C	2.757171	3.451048	-0.312669
S	3.843311	2.571298	-1.440555
O	4.930289	3.466043	-1.793198
O	3.019126	1.995476	-2.487148
H	2.332735	4.277833	-0.882707
H	1.986252	2.759250	0.016321
H	3.358763	3.810305	0.518005

TS (3-III)

32
scf done: -1107.391966

C	-0.087498	0.252109	0.063188
C	0.034324	-0.074877	1.432691
C	1.337825	-0.129789	1.977065
C	2.444544	0.121024	1.199321
C	2.299855	0.439480	-0.148145
C	1.027386	0.505076	-0.705252
C	-1.085307	-0.352642	2.245532
C	-2.458826	-0.422676	1.759187
C	-2.920885	-1.611110	0.981093
N	-2.999776	-2.780598	1.870503
C	-3.368443	-2.450385	3.183534
C	-3.544147	-1.203708	3.561365
C	-3.968135	-0.206632	4.327283
S	-2.084700	-4.122612	1.594886
O	-2.536341	-5.122602	2.513625
C	-0.436249	-3.682258	2.028493
O	-2.126758	-4.371196	0.186910
H	-2.251667	-1.858938	0.150355
H	-3.914951	-1.429789	0.564338
H	0.172311	-4.583175	1.957539
H	-0.075825	-2.926045	1.331820
H	-0.431265	-3.297500	3.048950
H	-3.493207	-3.283618	3.864886
H	-0.898924	-0.521651	3.306156
H	-5.021422	0.046198	4.407676
H	-3.265478	0.469060	4.808483
H	-2.986616	0.507655	1.560626
H	1.450812	-0.381132	3.027319
H	3.434095	0.070150	1.639027
H	3.172771	0.638232	-0.758175
H	0.910268	0.760605	-1.752256
H	-1.075878	0.321458	-0.379561

^{3}III

32
scf done: -1107.471149

C	-3.462008	-2.370116	-1.108423

C	-3.878110	-3.223579	-2.307371
C	-4.293917	-2.172694	-3.338947
N	-3.520731	-0.983867	-2.940348
C	-3.234311	-1.084728	-1.576530
C	-4.940979	-4.212037	-2.017749
C	-4.753283	-5.575154	-1.738643
C	-3.482507	-6.199615	-1.715113
C	-3.357289	-7.541026	-1.431179
C	-4.478472	-8.317963	-1.158525
C	-5.739691	-7.728094	-1.174194
C	-5.877409	-6.389765	-1.456127
S	-3.831720	0.480787	-3.632970
O	-4.004141	0.236480	-5.030834
C	-3.306493	-2.837455	0.162304
C	-5.369817	0.996222	-2.951489
O	-2.815229	1.375447	-3.174166
H	-2.974028	-3.725621	-2.670856
H	-2.827226	-0.236377	-1.045055
H	-5.369474	-1.967390	-3.252968
H	-4.070466	-2.452511	-4.365922
H	-5.956911	-3.825228	-1.961497
H	-6.861632	-5.932411	-1.466540
H	-6.618509	-8.325954	-0.961299
H	-4.370635	-9.372324	-0.933843
H	-2.372444	-7.994121	-1.417970
H	-2.592047	-5.615148	-1.917854
H	-5.595166	1.978388	-3.365032
H	-6.145956	0.286390	-3.233443
H	-5.270581	1.055785	-1.868128
H	-3.523966	-3.869851	0.407475
H	-2.972045	-2.186029	0.960382

TS (III-IV)

32

scf done: -1107.437330

C	-0.718373	-0.106016	0.380737
C	-0.278107	-0.045284	1.725794
C	1.118535	-0.106509	1.950476
C	2.001621	-0.216036	0.899576
C	1.542265	-0.271166	-0.411912
C	0.173011	-0.215601	-0.659933
C	-1.220314	0.075248	2.759487
C	-0.925722	0.093823	4.216200
C	-1.134281	-1.295880	4.801174
C	-2.338361	-1.219553	5.580155
N	-2.827691	-0.019390	5.704433
C	-1.934875	0.928433	5.023535
C	-0.372772	-2.378525	4.627517
S	-4.917100	0.225169	5.089788
O	-5.525365	-0.914679	5.737206
O	-5.270986	1.555808	5.530546
C	-5.269549	0.100593	3.333034
H	0.093244	0.437757	4.404242
H	-2.832229	-2.072657	6.036779
H	-2.516136	1.615572	4.402881
H	-1.441733	1.529976	5.790465
H	-2.272702	0.077694	2.478527
H	-1.784876	-0.063005	0.183822
H	-0.193692	-0.259433	-1.679143
H	2.243725	-0.358246	-1.232916
H	3.066273	-0.259925	1.099370
H	1.503797	-0.065400	2.963107
H	-6.355297	0.096524	3.224283

H	-4.832037	0.961852	2.832431
H	-4.843689	-0.833677	2.970786
H	0.528917	-2.352412	4.026706
H	-0.637929	-3.324283	5.085723

^{3}IV

32

scf done: -1107.447628

C	0.365505	-0.290033	-0.152482
C	0.278789	-0.161691	1.255667
C	1.497417	-0.105028	1.974334
C	2.708766	-0.164373	1.321822
C	2.764852	-0.282903	-0.063144
C	1.580609	-0.346807	-0.792843
C	-0.982588	-0.101448	1.869524
C	-1.238816	0.037879	3.321486
C	-1.451518	-1.278092	4.048842
C	-2.607142	-1.052198	4.908347
N	-3.200744	0.058690	4.740571
C	-2.530751	0.800363	3.675588
C	-0.774676	-2.412121	3.929213
S	-5.386722	0.185493	6.728502
O	-5.358071	-1.261775	6.859030
O	-5.899493	0.978338	7.836233
C	-6.381078	0.579962	5.288665
H	-0.394230	0.542548	3.805550
H	-2.964853	-1.783819	5.632100
H	-3.212076	0.858286	2.819244
H	-2.344132	1.826690	3.996501
H	-1.855481	-0.207326	1.228568
H	-0.555880	-0.340231	-0.724024
H	1.615995	-0.441895	-1.872123
H	3.722146	-0.328462	-0.568501
H	3.627407	-0.120893	1.895996
H	1.478059	-0.024058	3.055763
H	-7.402838	0.283017	5.525995
H	-6.312100	1.651559	5.118340
H	-5.976018	0.016670	4.452726
H	0.050336	-2.516396	3.232950
H	-1.033094	-3.279170	4.526433

TS (3-V)

39

scf done: -1695.970008

C	1.612130	-1.072801	0.310633
C	1.080199	-0.893900	1.572518
C	1.631039	0.037845	2.453501
C	2.724408	0.787014	2.021203
C	3.259485	0.608478	0.757794
C	2.705465	-0.323919	-0.102767
C	1.128899	0.247399	3.806916
C	0.155826	-0.421044	4.418175
C	-0.304353	-0.113779	5.800329
N	-0.156978	-1.241531	6.735727
S	-1.404020	-2.331892	6.966062
O	-2.605440	-1.650316	6.603675
C	1.078248	-1.548775	7.267505
C	2.237008	-1.010356	6.855636
S	2.966107	-2.587380	5.065419
C	3.774205	-3.757576	6.153756
C	3.242986	-0.183710	6.889498
O	-1.246475	-2.867916	8.281608
C	-1.086312	-3.615580	5.811654

O	1.856493	-3.302424	4.448758
O	3.998183	-2.033844	4.196965
H	-1.358569	0.165361	5.813783
H	0.261832	0.719695	6.222786
H	-1.816604	-4.402484	5.998287
H	-1.193754	-3.224263	4.801717
H	-0.070705	-3.980325	5.965795
H	1.081985	-2.350776	8.000128
H	1.628935	1.039354	4.364293
H	-0.364104	-1.228230	3.906928
H	3.163073	1.513289	2.698387
H	4.112742	1.199073	0.445025
H	3.120709	-0.467428	-1.093383
H	1.170893	-1.801407	-0.359613
H	0.223395	-1.485563	1.875879
H	4.085470	-0.263554	6.208666
H	3.266496	0.631666	7.607390
H	4.162659	-4.564201	5.531501
H	4.578762	-3.241588	6.674535
H	3.028077	-4.131906	6.853421

^{2}V

39

scf done: -1696.007693

C	-0.265965	0.700926	1.627030
C	0.800168	-0.042937	2.137473
C	0.685987	-0.559570	3.427359
C	-0.455481	-0.352878	4.181829
C	-1.506172	0.381798	3.659444
C	-1.404984	0.908889	2.379285
C	2.025828	-0.292767	1.386074
C	2.260695	-0.006183	0.107310
C	3.580058	-0.250441	-0.539158
N	4.471880	0.941687	-0.482960
C	5.224808	1.222941	0.643655
C	5.978256	0.298437	1.351458
S	4.109771	2.241769	-1.468586
O	5.316940	2.992397	-1.614361
C	2.969737	3.232099	-0.562987
O	3.447113	1.700914	-2.612924
H	3.479962	-0.530981	-1.585659
H	4.104381	-1.054860	-0.027715
H	2.773481	4.113240	-1.173197
H	2.050573	2.674741	-0.393350
H	3.419598	3.531459	0.382217
H	5.249155	2.256202	0.971987
H	2.820121	-0.778863	1.953512
H	1.488032	0.444324	-0.510758
H	1.510667	-1.132932	3.838535
H	-0.523113	-0.765009	5.181865
H	-2.400658	0.549646	4.247530
H	-2.221261	1.491035	1.967694
H	-0.203041	1.128250	0.632009
C	6.333009	-0.985093	1.022183
S	6.652123	0.919526	2.883853
H	6.905732	-1.594127	1.709033
H	6.101936	-1.408739	0.054036
O	5.993979	2.154269	3.217349
O	6.645190	-0.149987	3.844338
C	8.325383	1.282697	2.470380
H	8.794903	1.667639	3.375575
H	8.818397	0.368912	2.143207
H	8.339393	2.035601	1.683738

TS (V-VI)

39

scf done: -1695.973466

C	-0.043280	0.488725	0.334585
C	0.237496	-0.844019	0.580499
C	1.397309	-1.194330	1.249599
C	2.299656	-0.224656	1.685437
C	2.000702	1.115465	1.431957
C	0.844303	1.466096	0.764123
C	3.503905	-0.657016	2.388959
C	4.465675	0.114208	2.887364
C	5.670037	-0.355556	3.632387
N	5.634071	-1.722247	4.096634
C	5.999094	-2.761646	3.421737
C	6.525201	-2.845259	2.076859
C	6.439729	-1.983324	1.058560
S	7.434133	-4.355461	1.773083
O	7.828909	-4.383916	0.392735
C	8.879437	-4.180429	2.761693
O	6.652389	-5.443954	2.296100
S	4.054602	-1.999549	5.528235
C	2.277015	-1.888317	5.235619
O	4.398934	-0.953622	6.463725
O	4.332633	-3.375629	5.872608
H	5.796893	0.276422	4.517480
H	6.573021	-0.212064	3.022225
H	1.798381	-2.161630	6.177566
H	2.039377	-0.865127	4.953290
H	2.012540	-2.591807	4.448653
H	5.940385	-3.702681	3.970651
H	3.586635	-1.737320	2.508887
H	4.418445	1.193580	2.772936
H	2.679499	1.894662	1.760446
H	0.630069	2.511812	0.575948
H	-0.949534	0.768635	-0.189373
H	-0.448108	-1.615497	0.249995
H	1.617558	-2.240495	1.439228
H	6.937961	-2.198276	0.121898
H	5.838139	-1.085905	1.110843
H	9.464693	-5.088795	2.618433
H	9.436931	-3.309106	2.422044
H	8.593335	-4.078516	3.807577

VI

32

scf done: -1107.494400

C	-1.567891	0.261787	1.905483
C	-0.265743	0.323464	1.450031
C	0.735549	-0.452210	2.038283
C	0.379372	-1.293526	3.091554
C	-0.925510	-1.357043	3.548967
C	-1.905305	-0.578100	2.957933
C	2.129248	-0.420985	1.601135
C	2.665193	0.371648	0.677780
C	4.098579	0.317526	0.271224
N	4.670075	1.651226	0.284580
C	5.523405	1.997056	1.147926
C	6.057218	1.201536	2.276342
S	7.825921	1.082984	2.348405
C	8.199701	0.058907	0.967072
C	5.343551	0.655980	3.247650
O	8.356471	2.396939	2.099263

O	8.205648	0.394155	3.549678
H	4.161264	-0.030121	-0.767373
H	4.642310	-0.405224	0.892883
H	5.942672	3.004130	1.067976
H	2.781562	-1.136443	2.102180
H	2.062893	1.115203	0.161303
H	1.145823	-1.905637	3.556496
H	-1.177148	-2.018058	4.370248
H	-2.928616	-0.625249	3.311179
H	-2.330088	0.871376	1.433813
H	-0.024782	0.979526	0.621028
H	5.820344	0.126724	4.063915
H	4.262636	0.747051	3.259565
H	9.284992	-0.033932	0.927769
H	7.737088	-0.916236	1.113647
H	7.830811	0.534616	0.058825

^{3}VI

32

scf done: -1107.411141

C	-0.335546	1.307530	2.055636
C	0.658388	0.689631	1.331131
C	1.190727	-0.553314	1.744561
C	0.666838	-1.130330	2.924460
C	-0.324284	-0.501668	3.642291
C	-0.835093	0.720814	3.214709
C	2.204769	-1.208109	1.017636
C	2.835613	-0.691476	-0.179455
C	4.138157	0.033749	-0.108588
N	3.976470	1.294495	0.598105
C	4.481702	1.480940	1.740131
C	5.295671	0.545343	2.554315
S	6.974836	1.058254	2.815728
C	7.709559	0.832270	1.232491
C	4.868265	-0.547744	3.163688
O	6.948319	2.466785	3.108524
O	7.605288	0.164646	3.746341
H	4.480655	0.274339	-1.117624
H	4.896812	-0.616218	0.357434
H	4.340230	2.464916	2.196384
H	2.520142	-2.187896	1.384510
H	2.425775	-0.927276	-1.158144
H	1.063412	-2.085000	3.257112
H	-0.710128	-0.961571	4.545019
H	-1.617633	1.212438	3.780287
H	-0.733046	2.258525	1.719252
H	1.049527	1.147160	0.427634
H	5.535537	-1.140279	3.778382
H	3.834190	-0.865183	3.078724
H	8.735653	1.192459	1.309507
H	7.694187	-0.227368	0.982109
H	7.162245	1.418214	0.494475

TS (VI-VII)

32

scf done: -1107.398933

C	-0.070044	-0.179135	0.127847
C	0.054283	0.109440	1.484454
C	1.319997	0.270603	2.038262
C	2.447157	0.142166	1.258280
C	2.347345	-0.157643	-0.120776
C	1.048397	-0.308890	-0.661379
C	3.477676	-0.297557	-0.947098

C	4.853449	-0.216829	-0.483266
C	5.449151	-1.275191	0.395069
N	6.838636	-1.568076	0.061889
C	7.146662	-1.350025	-1.140979
C	6.173273	-0.907978	-2.175981
S	6.627530	0.520224	-3.149144
O	7.864047	0.180686	-3.805003
C	5.427335	-1.816695	-2.856436
O	5.496429	0.896396	-3.951890
C	6.987110	1.832872	-2.032838
H	4.869178	-2.203308	0.329412
H	5.418547	-0.971297	1.447414
H	8.171346	-1.532044	-1.472003
H	3.301977	-0.494760	-2.004768
H	5.278049	0.775442	-0.326518
H	0.949731	-0.534334	-1.718403
H	-1.054125	-0.302254	-0.309955
H	-0.828800	0.210785	2.103679
H	1.421179	0.503299	3.092217
H	3.426909	0.286453	1.701626
H	4.887673	-1.545822	-3.755818
H	5.313027	-2.825993	-2.476812
H	7.476855	2.591577	-2.643426
H	6.071478	2.233821	-1.605567
H	7.673981	1.486673	-1.261040

4

32

scf done: -1107.530657

C	-0.293565	0.044279	0.611012
C	-0.101461	0.762616	1.784991
C	1.184453	1.204940	2.089598
C	2.244603	0.932946	1.243492
C	2.039465	0.213279	0.075813
C	0.766485	-0.230122	-0.238544
C	-1.244184	1.028668	2.708355
C	-1.118493	0.532205	4.176497
C	-1.638316	1.907911	4.640012
C	-3.044293	1.561513	5.042803
N	-3.338697	0.333002	5.043960
C	-2.164162	-0.455767	4.665786
C	-1.459434	2.472812	3.224557
O	0.651553	2.846865	5.466392
S	-0.692555	2.658054	5.946615
O	-0.892226	1.866965	7.133024
C	-1.424888	4.242656	6.167912
H	-1.836151	-1.022558	5.542241
H	-2.442720	-1.184294	3.899252
H	-3.779027	2.323545	5.301269
H	-2.307887	3.023341	2.817525
H	-0.559806	3.084563	3.141117
H	-0.094484	0.292823	4.464424
H	-2.167908	0.638333	2.270080
H	-1.290527	-0.305851	0.362350
H	0.595327	-0.792976	-1.148997
H	2.870677	0.000491	-0.586219
H	3.238496	1.284119	1.496463
H	1.360156	1.766672	3.003256
H	-0.818106	4.768924	6.904336
H	-1.404692	4.774425	5.216944
H	-2.443703	4.127748	6.534056

TS (V-VIII)

39
scf done: -1695.985472

C	0.694764	-0.037657	-0.411990
C	0.773102	-0.862791	0.700085
C	1.899369	-0.845529	1.501312
C	2.966352	0.002542	1.209896
C	2.882425	0.813828	0.081015
C	1.755158	0.798397	-0.721367
C	4.175920	0.030596	2.041747
C	4.160240	-0.233345	3.392121
C	5.420809	-0.350521	4.158259
N	5.671187	-1.784081	4.484485
C	5.351443	-2.804072	3.607742
C	5.202016	-2.723723	2.266452
C	5.421934	-1.600023	1.400245
S	5.845925	-2.185906	6.100363
C	4.206285	-2.122649	6.730382
O	6.614906	-1.143131	6.700495
O	6.304310	-3.538018	6.146262
S	4.648918	-4.210117	1.492674
O	3.637074	-3.869856	0.525872
C	6.066214	-4.774187	0.608018
O	4.333640	-5.192372	2.497749
H	5.395738	0.186787	5.107770
H	6.273190	0.043467	3.601289
H	4.249818	-2.408099	7.780927
H	3.823425	-1.107432	6.635888
H	3.590566	-2.826471	6.171073
H	5.220116	-3.772333	4.076987
H	4.969709	0.674021	1.667388
H	3.263574	-0.625896	3.865282
H	3.714008	1.467717	-0.162503
H	1.704788	1.440977	-1.592693
H	-0.188010	-0.053428	-1.040324
H	-0.046806	-1.530206	0.938936
H	1.964222	-1.517326	2.351223
H	5.116572	-1.698173	0.364175
H	6.339681	-1.042760	1.546346
H	5.766235	-5.674561	0.071941
H	6.379715	-4.003616	-0.094685
H	6.860343	-4.997594	1.318331

^{2}VIII

39
scf done: -1696.024923

C	0.655965	-0.570210	0.399152
C	0.972042	-0.966362	1.687521
C	2.257557	-0.790245	2.175066
C	3.245595	-0.218778	1.383621
C	2.913943	0.180860	0.093688
C	1.632587	0.007863	-0.397817
C	4.674437	-0.086468	1.858441
C	4.782795	0.135910	3.323841
C	5.803313	-0.534013	4.154673
N	5.458087	-1.957153	4.433815
C	5.145234	-2.824547	3.415441
C	5.163246	-2.570910	2.099362
C	5.512686	-1.289205	1.394385
S	5.109596	-2.397799	6.006132
C	3.474978	-1.800660	6.246509
O	5.995451	-1.659385	6.848160
O	5.097562	-3.825356	6.051362
S	4.742361	-3.935187	1.078446

O	3.886106	-3.472803	0.015787
C	6.278309	-4.406844	0.351153
O	4.283459	-5.030079	1.894350
H	5.920858	-0.041346	5.117928
H	6.791466	-0.537132	3.685607
H	3.203610	-1.989113	7.284750
H	3.458252	-0.730289	6.041363
H	2.805706	-2.338660	5.576121
H	4.864826	-3.817328	3.749435
H	5.095933	0.790696	1.340213
H	4.120356	0.860457	3.785407
H	3.678226	0.631587	-0.533383
H	1.393164	0.327528	-1.405535
H	-0.349178	-0.706381	0.017529
H	0.214476	-1.415981	2.319148
H	2.498461	-1.105014	3.186078
H	5.352409	-1.411561	0.321000
H	6.577264	-1.068385	1.514567
H	6.061337	-5.199027	-0.365547
H	6.712117	-3.549373	-0.161935
H	6.944284	-4.766836	1.133334

TS (VIII-IX)

39

scf done: -1695.982428

C	-0.244712	0.081370	-0.079057
C	0.010182	0.118206	1.290201
C	1.318222	-0.046472	1.727518
C	2.351049	-0.244206	0.824559
C	2.087342	-0.276815	-0.533859
C	0.785327	-0.113132	-0.982348
C	-1.096380	0.350533	2.276278
C	-2.290395	-0.605469	2.194937
C	-3.495859	0.334604	2.078427
C	-1.842101	1.653545	2.094813
C	-2.211815	2.380113	3.329597
N	-3.241672	1.551090	4.037079
C	-4.118848	0.930456	3.158005
S	-4.525673	0.092503	0.664320
O	-5.605743	1.042091	0.702412
S	-3.604153	1.802006	5.624899
C	-2.746471	0.495441	6.425021
C	-5.208062	-1.518139	0.895533
O	-3.680403	0.060220	-0.501829
O	-3.006841	3.044315	6.007129
O	-5.010005	1.596196	5.793313
H	-2.629359	3.368147	3.123126
H	-1.351599	2.499545	3.995016
H	-5.173341	1.181068	3.193511
H	-2.390696	-1.267059	3.059576
H	-2.223041	-1.232969	1.303634
H	-1.722087	2.219417	1.177231
H	-0.667740	0.328888	3.285415
H	1.529245	-0.019490	2.791984
H	3.365087	-0.371901	1.185716
H	2.892526	-0.429135	-1.243111
H	0.571062	-0.136643	-2.044811
H	-1.261990	0.205774	-0.439930
H	-5.823073	-1.726439	0.019873
H	-4.407515	-2.252161	0.971128
H	-5.817907	-1.512444	1.797854
H	-2.931816	0.579741	7.495427
H	-3.126350	-0.451580	6.043545

H	-1.682662	0.598699	6.212916

^{2}IX

39

scf done: -1696.028895

C	-2.033167	-0.424624	4.939095
C	-1.123625	0.632974	4.355379
C	-1.651800	2.006103	4.827667
C	-3.031196	1.707678	5.235001
N	-3.242454	0.334306	5.307907
C	-1.360516	1.107280	2.896675
C	-1.444771	2.564471	3.407794
S	-0.662725	2.732842	6.141075
C	-1.450470	4.273558	6.462686
C	-0.341183	0.749826	1.867152
C	-0.697895	0.011979	0.745117
C	0.245772	-0.333441	-0.209222
C	1.564740	0.056186	-0.052679
C	1.934296	0.790223	1.064417
C	0.989825	1.132368	2.015445
O	0.657319	2.993142	5.624151
O	-0.785767	1.882498	7.297634
H	-1.608600	-0.887424	5.832091
H	-2.271537	-1.216990	4.225423
H	-3.844454	2.398057	5.399468
H	-2.244627	3.190785	3.014607
H	-0.489046	3.084293	3.316381
H	-0.067103	0.458784	4.564457
H	-2.348819	0.774285	2.563096
H	-1.731958	-0.293857	0.618622
H	-0.052305	-0.908586	-1.078284
H	2.304724	-0.210402	-0.798094
H	2.965389	1.098241	1.194564
H	1.292265	1.706598	2.887050
H	-0.846656	4.789989	7.208184
H	-1.480352	4.849848	5.537968
H	-2.452728	4.091140	6.846956
S	-4.751460	-0.323860	5.197589
O	-4.617436	-1.702628	5.548072
O	-5.627668	0.533787	5.932129
C	-5.169908	-0.203710	3.492231
H	-6.194929	-0.554811	3.378672
H	-5.094551	0.841986	3.192367
H	-4.494754	-0.829378	2.909788

TS (IX-4)

39

scf done: -1696.003826

C	-0.099932	-0.031188	0.100321
C	-0.118658	0.067706	1.489541
C	1.091263	0.069249	2.172504
C	2.293155	-0.024827	1.488980
C	2.299550	-0.120539	0.108057
C	1.098142	-0.123189	-0.584960
C	-1.409590	0.178817	2.229977
C	-2.512724	-0.872449	1.958020
C	-3.539257	0.272654	1.890936
C	-2.394349	1.305927	1.820308
C	-2.740548	2.351491	2.861039
N	-3.740944	1.716338	3.736700
C	-4.201552	0.637578	3.173855
S	-4.643946	0.297096	0.486708
O	-5.358110	1.546122	0.520785

S	-3.096801	1.567534	5.743988
C	-1.607131	0.624383	6.084094
C	-5.760308	-1.026177	0.792664
O	-3.871158	-0.020045	-0.685475
O	-2.869605	2.939757	6.125475
O	-4.204263	0.843366	6.317261
H	-3.201143	3.239233	2.420639
H	-1.879858	2.684666	3.446829
H	-4.955670	0.032396	3.668231
H	-2.682409	-1.625267	2.728426
H	-2.386972	-1.362718	0.990601
H	-2.193565	1.713469	0.829378
H	-1.205951	0.244276	3.302940
H	1.088384	0.144381	3.255791
H	3.227445	-0.023062	2.038544
H	3.238063	-0.193685	-0.428929
H	1.095633	-0.197385	-1.666240
H	-1.035889	-0.034835	-0.452052
H	-6.406198	-1.101876	-0.081708
H	-5.189388	-1.945960	0.918207
H	-6.352282	-0.803768	1.678868
H	-1.510667	0.566844	7.169604
H	-1.734276	-0.370618	5.658867
H	-0.756758	1.144219	5.646273

TS (IV-X)

32

scf done: -1107.443806

C	-0.035018	-0.314541	-0.058359
C	-0.103259	-0.151249	1.435417
C	1.390434	0.063234	1.778283
N	2.089425	0.343099	0.530659
C	1.292096	0.113389	-0.440222
C	-0.766828	-1.265963	2.146963
C	-1.764157	-1.167728	3.130470
C	-2.294195	0.061058	3.593158
C	-3.270456	0.091714	4.563547
C	-3.762994	-1.086387	5.115630
C	-3.258366	-2.308453	4.678867
C	-2.282613	-2.351839	3.711681
C	-1.035178	-0.691391	-0.882936
S	-2.222110	1.580384	-1.006604
O	-1.169012	2.570088	-1.128999
C	-3.276164	1.716722	-2.449072
O	-3.072188	1.614933	0.170281
H	-0.633303	0.793119	1.623601
H	1.605767	0.216608	-1.477568
H	1.827491	-0.838163	2.223657
H	1.549087	0.873402	2.492475
H	-0.419101	-2.265112	1.889214
H	-1.890341	-3.306161	3.374777
H	-3.635544	-3.231886	5.103572
H	-4.531183	-1.052653	5.878760
H	-3.657828	1.046670	4.900374
H	-1.928730	0.994643	3.181183
H	-0.865949	-0.789637	-1.949531
H	-1.953993	-1.109528	-0.484225
H	-3.737300	2.704139	-2.403709
H	-2.652149	1.615235	-3.333807
H	-4.025550	0.930760	-2.395045

^{3}X

32

scf done: -1107.475888

C	1.579722	0.371556	1.605514
C	0.379325	-0.182802	1.098828
C	0.355480	-0.515840	-0.278592
C	1.451567	-0.317098	-1.083926
C	2.621307	0.226005	-0.559268
C	2.669909	0.568234	0.788354
C	-0.765099	-0.413241	1.880251
C	-0.927595	-0.156119	3.332997
C	-2.053266	0.845454	3.690504
N	-2.895274	0.241511	4.708735
C	-2.484535	-0.988037	4.877303
C	-1.381471	-1.358477	4.106254
C	-0.728233	-2.673754	4.072103
H	0.023259	0.185556	3.760700
H	-2.988937	-1.657730	5.570456
H	-2.673254	1.071989	2.814083
H	-1.665010	1.803900	4.044515
H	-1.639065	-0.821345	1.373676
H	-0.554844	-0.939473	-0.691072
H	1.403458	-0.586108	-2.132970
H	3.484867	0.382517	-1.194359
H	3.576670	0.994687	1.202481
H	1.646462	0.646511	2.651727
H	-1.384485	-3.475007	4.419861
H	-0.342104	-2.915197	3.075915
S	0.697208	-2.684320	5.172061
C	1.336351	-4.314054	4.993730
O	1.663546	-1.747321	4.662255
O	0.220659	-2.513305	6.518439
H	2.212603	-4.375777	5.638601
H	0.581775	-5.031281	5.312035
H	1.620213	-4.474711	3.954922

TS (IV-XI)

32

scf done: -1107.434856

C	-0.155016	-0.169951	0.404597
C	-0.069776	0.265772	1.749640
C	1.175759	0.780547	2.184313
C	2.254206	0.839552	1.329964
C	2.145732	0.395741	0.016326
C	0.929469	-0.107472	-0.437700
C	-1.196879	0.170324	2.581488
C	-1.261097	0.573377	4.009065
C	-1.891040	1.962308	4.156888
C	-3.211252	1.703847	4.763443
N	-3.426193	0.507187	5.116280
C	-2.242882	-0.295232	4.824580
C	-1.640468	3.043387	3.338251
O	0.647545	2.570216	5.745796
S	-0.757903	2.602171	6.097116
O	-1.202828	1.854015	7.256591
C	-1.231085	4.302677	6.321430
H	-1.811062	-0.626128	5.773035
H	-2.538463	-1.196637	4.281686
H	-3.951819	2.492307	4.896211
H	-2.304269	3.900735	3.341078
H	-0.751553	3.092830	2.720219
H	-0.260339	0.555898	4.448602
H	-2.124410	-0.187875	2.138006
H	-1.101648	-0.563409	0.048127
H	0.835623	-0.451939	-1.461307

H	2.999654	0.444240	-0.648610
H	3.196866	1.238285	1.687407
H	1.284368	1.141437	3.201690
H	-0.731945	4.649379	7.226400
H	-0.900364	4.867831	5.452440
H	-2.313007	4.346703	6.437322

^{3}XI

32

scf done: -1107.446096

C	-0.219978	-0.078377	0.488042
C	-0.068616	0.296415	1.845083
C	1.193465	0.799351	2.242009
C	2.226412	0.908618	1.337503
C	2.053216	0.527217	0.011145
C	0.819491	0.034060	-0.404672
C	-1.154771	0.163563	2.725633
C	-1.156459	0.505658	4.167642
C	-1.682332	1.969061	4.418665
C	-3.075797	1.691918	4.915793
N	-3.353960	0.493467	5.189608
C	-2.182943	-0.338033	4.947197
C	-1.612497	2.925487	3.307639
O	0.662745	2.855483	5.289965
S	-0.666756	2.647134	5.801815
O	-0.829490	1.806058	6.958589
C	-1.391840	4.218650	6.134787
H	-1.794096	-0.676181	5.911062
H	-2.477216	-1.232050	4.391728
H	-3.805803	2.494960	5.020683
H	-2.498519	3.461339	2.991035
H	-0.670716	3.131380	2.816474
H	-0.153130	0.393045	4.587364
H	-2.100206	-0.178661	2.307660
H	-1.181100	-0.462916	0.161906
H	0.676350	-0.262699	-1.437439
H	2.871171	0.616584	-0.693684
H	3.183304	1.299411	1.664590
H	1.346468	1.117306	3.267520
H	-0.803391	4.660595	6.938555
H	-1.336490	4.833814	5.237960
H	-2.422587	4.080472	6.458515

4^{III}

32

scf done: -1107.532875

C	-1.805971	-0.410722	5.245281
C	-0.836496	0.276897	4.312024
C	-1.605243	1.436182	3.728389
C	-2.957121	1.236668	4.304991
N	-3.105376	0.245406	5.079465
C	-1.176816	0.243077	2.865643
C	-0.158432	0.394650	1.805397
C	-0.502289	0.048279	0.500462
C	0.408231	0.166823	-0.533760
C	1.685869	0.642252	-0.284225
C	2.040702	0.994071	1.007138
C	1.129708	0.870618	2.043256
C	-1.104182	2.820914	3.478858
S	-0.376917	3.504067	4.958705
C	-0.170043	5.206564	4.566458
O	0.926174	2.918212	5.142788
O	-1.333888	3.384156	6.028078

H	-1.500987	-0.318921	6.292462
H	-1.904981	-1.479955	5.035965
H	-3.784008	1.905869	4.066033
H	-1.925867	3.485581	3.197549
H	-0.336864	2.867806	2.702231
H	0.200256	0.394129	4.598341
H	-2.004614	-0.404069	2.584604
H	-1.501507	-0.325600	0.301053
H	0.118941	-0.114190	-1.539849
H	2.401980	0.736445	-1.091968
H	3.037824	1.365214	1.214121
H	1.433789	1.148186	3.047359
H	0.316477	5.666934	5.425935
H	0.463222	5.294173	3.684864
H	-1.145634	5.658426	4.396336

4^{IV}

32
scf done: -1107.529897

C	-2.381814	-0.126222	5.058674
C	-1.204097	0.285203	4.150884
C	-1.611975	1.690984	3.750686
C	-2.705711	2.023573	4.658462
N	-3.127360	1.080002	5.395785
C	-0.896212	-0.662470	2.994143
C	0.183870	-0.178483	2.066678
C	-0.058953	-0.139123	0.700154
C	0.899077	0.345020	-0.173493
C	2.120295	0.782851	0.312029
C	2.378649	0.727110	1.672307
C	1.417529	0.248662	2.545842
C	-1.147436	2.534842	2.835427
S	-0.379662	-2.322629	3.561102
C	-1.838979	-3.179115	4.062714
O	0.478881	-2.167378	4.707292
O	0.126297	-3.021466	2.409510
H	-2.057362	-0.632165	5.971188
H	-3.075270	-0.802892	4.546030
H	-3.140762	3.021419	4.697101
H	-1.593024	3.520182	2.749560
H	-0.337142	2.304765	2.156497
H	-0.305233	0.362488	4.773166
H	-1.804810	-0.851205	2.409805
H	-1.017568	-0.478416	0.320867
H	0.689876	0.381632	-1.236033
H	2.870770	1.166395	-0.368963
H	3.333466	1.064520	2.057871
H	1.628724	0.224023	3.608985
H	-1.518781	-4.211593	4.205183
H	-2.224356	-2.779100	4.996455
H	-2.582112	-3.130060	3.268167

$^{3}4^{IV}$

32
scf done: -1107.447703

C	-2.472384	-0.141554	5.022873
C	-1.236085	0.231573	4.184221
C	-1.564884	1.659153	3.804166
C	-2.710671	2.010337	4.531563
N	-3.239939	1.066217	5.263319
C	-0.933137	-0.685831	2.996554
C	0.115416	-0.164108	2.053131
C	-0.158559	-0.113275	0.692826

C	0.770144	0.400409	-0.195420
C	1.993068	0.856309	0.268390
C	2.283228	0.789383	1.621731
C	1.351650	0.281817	2.510109
C	-0.767888	2.537739	2.984123
S	-0.376094	-2.345130	3.516652
C	-1.790790	-3.193151	4.142026
O	0.572666	-2.193819	4.589997
O	0.033596	-3.042560	2.326648
H	-2.217472	-0.618564	5.973789
H	-3.124599	-0.841897	4.486059
H	-3.144122	3.006810	4.514597
H	-0.932496	2.632348	1.916038
H	0.047854	3.111896	3.412176
H	-0.342936	0.249534	4.825127
H	-1.851522	-0.879757	2.429716
H	-1.117330	-0.469766	0.330152
H	0.537499	0.443841	-1.252834
H	2.721220	1.260989	-0.424579
H	3.240135	1.139882	1.990161
H	1.586832	0.247371	3.567808
H	-1.458735	-4.222158	4.282452
H	-2.101911	-2.771658	5.094038
H	-2.592937	-3.161029	3.406467

TS (IV-XII)

25

scf done: -518.907289

C	0.006197	-0.217950	-0.060600
C	-0.133847	0.216707	1.263844
C	1.034322	0.490701	1.990811
C	2.281142	0.329348	1.424389
C	2.401282	-0.105672	0.110573
C	1.257876	-0.376600	-0.625349
C	-1.418855	0.451222	1.877177
C	-2.754475	-0.029821	1.345025
C	-3.642233	-0.870344	2.268321
N	-4.520523	0.058247	2.983736
C	-4.304487	1.252095	2.532073
C	-3.347775	1.340212	1.483564
C	-2.321965	2.252781	1.246620
H	-2.684660	-0.426715	0.331491
H	-4.818396	2.110774	2.959126
H	-3.060390	-1.449151	2.994560
H	-4.251497	-1.590424	1.714404
H	-1.412818	0.601404	2.955165
H	0.943376	0.831873	3.017240
H	3.169096	0.541779	2.008719
H	3.380589	-0.232932	-0.335281
H	1.342300	-0.717071	-1.651051
H	-0.873782	-0.434875	-0.655602
H	-1.743167	2.177194	0.331227
H	-2.305632	3.234743	1.714411

^{2}XII

25

scf done: -518.939843

C	-3.463347	1.161048	1.341872
C	-2.690528	-0.109439	1.451504
C	-3.409026	-0.857489	2.564748
N	-4.464119	0.033956	3.062419
C	-4.419912	1.142814	2.380785
C	-1.463880	0.833387	1.726193

C	-0.130792	0.457005	1.168597
C	0.054718	0.257524	-0.196854
C	1.291761	-0.106086	-0.698904
C	2.369250	-0.278272	0.156757
C	2.197930	-0.084328	1.516787
C	0.957528	0.280499	2.015299
C	-2.262940	2.015894	1.089268
H	-2.539606	-0.677480	0.530119
H	-5.073135	1.972817	2.639322
H	-2.748136	-1.118907	3.399845
H	-3.857489	-1.795515	2.224502
H	-1.391982	0.995810	2.805865
H	0.827795	0.432536	3.082253
H	3.033243	-0.215924	2.194981
H	3.337986	-0.562568	-0.237203
H	1.416272	-0.255926	-1.765282
H	-0.778645	0.386687	-0.880975
H	-2.019052	2.124419	0.028996
H	-2.209604	2.991124	1.572671

Comprehensive Table in A.U.

Lacvp(d) values	H (Hartrees)	S (cal/K*mol)	Imaginary freq (1/cm)
3	-1106.880894	135.836	
^{3}3	-1106.804725	136.17	
TS (3-III)	-1106.798218	133.323	-492.4888
^{3}III	-1106.875924	134.063	
TS (III-IV)	-1106.856009	135.658	-286.159
^{3}IV	-1106.868117	144.636	
3 + ^{2}Ms	-1695.181824	209.7	
TS (3-V)	-1695.190841	157.936	-206.7432
^{2}V	-1695.220438	162.604	
TS (V-VI)	-1695.191993	157.227	-370.6295
VI	-1106.900501	135.884	
^{3}VI	-1106.823273	139.579	
TS (VI-7-endo)	-1106.805698	131.639	-339.0999
TS (VI-VII)	-1106.81301	133.343	-391.8719
^{3}VII	-1106.861394	127.706	
4	-1106.94178	125.232	
TS (V-VIII)	-1695.200220	152.807	-647.6618
^{2}VIII	-1695.238531	152.176	
TS (VIII-IX)	-1695.200259	151.173	-625.3631
^{2}IX	-1695.245717	151.476	
TS (IX-4)	-1695.232621	149.66	-320.918
4 + ^{2}Ms	-1695.242710	199.096	
TS (IV-XI)	-1106.854991	132.675	-276.3483
^{3}XI	-1106.861394	127.706	
TS (IV-X)	-1106.863467	136.151	-163.3347
^{3}X	-1106.888645	135.263	
4'''	-1106.94181	122.855	
4^{IV}	-1106.938154	125.583	
$^{3}4^{IV}$	-1106.862152	130.067	

Def2-tzvp values	H (Hartrees)	S (cal/K*mol)	Imaginary freq (1/cm)
1	-1146.493303	144.27	
^{3}1	-1146.417069	139.018	
TS (1-II)	-1146.411644	132.997	-285.928
^{3}II	-1146.440102	138.747	
2	-1146.529487	133.083	
TS (1-II')	-1146.399568	135.39	
^{3}II'	-1146.479156	138.084	-616.6099
2'	-1146.529369	132.194	
^{2}Ms	-588.30093	73.864	
3	-1107.228361	135.164	
^{3}3	-1107.151390	135.844	
TS (3-III)	-1107.14244	131.839	-544.8373
^{3}III	-1107.217777	133.285	
TS (III-IV)	-1107.185694	134.697	-280.4895
^{3}IV	-1107.195668	144.53	
3 + ^{2}Ms	-2293.008525	270.278	
TS (3-V)	-1695.668898	158.19	-277.8522
^{2}V	-1695.704138	164.403	
TS (V-VI)	-1695.672168	159.877	-324.0021
VI	-1107.239316	136.464	
^{3}VI	-1107.160336	136.864	
TS (VI-VII)	-1107.148978	132.446	-437.3805
^{3}VII	-1107.193643	130.303	
4	-1107.271870	122.944	
TS (V-VIII)	-1695.681673	152.53	-679.0901
^{2}VIII	-1695.717872	150.157	
TS (VIII-IX)	-1695.677585	149.915	-649.7728
^{2}IX	-1695.721683	149.626	
TS (IX-4)	-1695.698783	150.138	-322.5286
4 + ^{2}Ms	-1625.557631	234.246	
TS (IV-XI)	-1107.18351	131.509	-320.8584
^{3}XI	-1107.193643	130.303	
4	-1107.271870	122.944	
TS (IV-X)	-1107.192465	137.375	-237.0548
^{3}X	-1107.222111	134.163	
4'''	-1107.274438	123.202	
4^{IV}	-1107.271834	124.862	
$^{3}4^{IV}$	-1107.194543	130.161	
TS (IV-XII)	-518.70264	101.571	-812.992
^{2}XII	-518.73281	102.039	

References

[1] Gaussian 09, Revision **A.1**, Frisch, M. J.; Trucks, G. W.; Schlegel, H. B.; Scuseria, G. E.; Robb, M. A.; Cheeseman, J. R.; Scalmani, G.; Barone, V.; Mennucci, B.; Petersson, G. A.; Nakatsuji, H.; Caricato, M.; Li, X.; Hratchian, H. P.; Izmaylov, A. F.; Bloino, J.; Zheng, G.; Sonnenberg, J. L.; Hada, M.; Ehara, M.; Toyota, K.; Fukuda, R.; Hasegawa, J.; Ishida, M.; Nakajima, T.; Honda, Y.; Kitao, O.; Nakai, H.; Vreven, T.; Montgomery, Jr., J. A.; Peralta, J. E.; Ogliaro, F.; Bearpark, M.; Heyd, J. J.; Brothers, E.; Kudin, K. N.; Staroverov, V. N.; Kobayashi, R.; Normand, J.; Raghavachari, K.; Rendell, A.; Burant, J. C.; Iyengar, S. S.; Tomasi, J.; Cossi, M.; Rega, N.; Millam, N. J.; Klene, M.; Knox, J. E.; Cross, J. B.; Bakken, V.; Adamo, C.; Jaramillo, J.; Gomperts, R.; Stratmann, R. E.; Yazyev, O.; Austin, A. J.; Cammi, R.; Pomelli, C.; Ochterski, J. W.; Martin, R. L.; Morokuma, K.; Zakrzewski, V. G.; Voth, G. A.; Salvador, P.; Dannenberg, J. J.; Dapprich, S.; Daniels, A. D.; Farkas, Ö.; Foresman, J. B.; Ortiz, J. V.; Cioslowski, J.; Fox, D. J. Gaussian, Inc., Wallingford CT, 2009.

[2] Zhao, Y.; Truhlar, D. G. *Theor. Chem. Account* **2008**, *120*, 215.

[3] Hay, J. P.; Wadt, W. R. *J. Chem. Phys.* **1985**, *82*, 299. Friesner, R. A.; Murphy, R. B.; Beachy, M. D.; Ringlanda, M. N.; Pollard, W. T.; Dunietz, B. D.; Cao, Y. X. *J. Phys. Chem. A* **1999**, *103*, 1913; Weigen, F.; Ahlrichs, R. *Phys. Chem. Chem. Phys.* **2005**, *7*, 3297; Andrae, D.; Haeussermann, U.; Dolg, M.; Stoll, H.; Preuss, H. *Theor. Chim. Acta* **1990**, *77*, 123; Peterson, K. A.; Figgen, D.; Goll, E.; Stoll, H.; Dolg, M. *J. Chem. Phys.* **2003**, *119*, 11113.

[4] Barone. V.; Cossi, M. *J. Phys. Chem. A* **1998**, *102*, 1995; Cossi, M.; Rega, N.; Scalmani, G.; Barone, V. *J. Comput. Chem.* **2003**, *24*, 669; Klamt, A.; Schüürmann, G. *J. Chem. Soc., Perkin Trans 2* **1993**, 799; Schäfer, A.; Klamt, A.; Sattel, D.; Lohrenz, J. C. W.; Eckert, F. *Phys. Chem. Chem. Phys.* **2000**, *2*, 2187.

[5] Kuang, J.; Ma, S. *J. Am. Chem. Soc.* **2010**, 132, 1786.

[6] Lu, Z.; Yoon, T. P. *Angew. Chem. Int. Ed*. **2012**, 51, 10329.

[7] Kuang, J.; Tang, X.; Ma, S. *Org. Chem. Front*. **2015**, 2, 470.

[8] Krause, L.; Herbst-Irmer, R.; Sheldrick G.M.; Stalke D. *J. Appl. Cryst.* **2015**, 48, 3.

[9] Sheldrick, G. M. *Acta Crystallogr. Sect. A Found. Crystallogr.* **2015**, A71, 3.

[10] Sheldrick, G. M. *Acta Crystallogr. Sect. C Struct. Chem.* **2015**, C71, 3.

[11] Dolomanov, O. V.; Bourhis, L. J.; Gildea, R. J.; Howard, J. A. K.; Puschmann, H.; *J. Appl. Cryst.* **2009**, 42, 339.
